# *Coxiella burnetii* and Related Tick Endosymbionts Evolved from Pathogenic Ancestors

**DOI:** 10.1093/gbe/evab108

**Published:** 2021-05-19

**Authors:** Amanda E Brenner, Sebastián Muñoz-Leal, Madhur Sachan, Marcelo B Labruna, Rahul Raghavan

**Affiliations:** Department of Biology and Center for Life in Extreme Environments, Portland State University, Portland, OR, USA; Departamento de Patología y Medicina Preventiva, Facultad de Ciencias Veterinarias, Universidad de Concepción, Chillán, Ñuble, Chile; Department of Biology and Center for Life in Extreme Environments, Portland State University, Portland, OR, USA; Departamento de Medicina Veterinária Preventiva e Saúde Animal, Faculdade de Medicina Veterinária e Zootecnia, Universidade de São Paulo, São Paulo, Brazil; Department of Biology and Center for Life in Extreme Environments, Portland State University, Portland, OR, USA; Department of Biology and South Texas Center for Emerging Infectious Diseases, The University of Texas at San Antonio, San Antonio, TX, USA

**Keywords:** Coxiella, tick, endosymbiont, pathogen, heme

## Abstract

Both symbiotic and pathogenic bacteria in the family Coxiellaceae cause morbidity and mortality in humans and animals. For instance, *Coxiella*-like endosymbionts (CLEs) improve the reproductive success of ticks—a major disease vector, while *Coxiella burnetii* causes human Q fever, and uncharacterized coxiellae infect both animals and humans. To better understand the evolution of pathogenesis and symbiosis in this group of intracellular bacteria, we sequenced the genome of a CLE present in the soft tick *Ornithodoros amblus* (CLEOA) and compared it to the genomes of other bacteria in the order Legionellales. Our analyses confirmed that CLEOA is more closely related to *C. burnetii*, the human pathogen, than to CLEs in hard ticks, and showed that most clades of CLEs contain both endosymbionts and pathogens, indicating that several CLE lineages have evolved independently from pathogenic *Coxiella*. We also determined that the last common ancestorof CLEOA and *C. burnetii* was equipped to infect macrophages and that even though horizontal gene transfer (HGT) contributed significantly to the evolution of *C. burnetii*, most acquisition events occurred primarily in ancestors predating the CLEOA–*C. burnetii* divergence. These discoveries clarify the evolution of *C. burnetii*, which previously was assumed to have emerged when an avirulent tick endosymbiont recently gained virulence factors via HGT. Finally, we identified several metabolic pathways, including heme biosynthesis, that are likely critical to the intracellular growth of the human pathogen but not the tick symbiont, and show that the use of heme analog is a promising approach to controlling *C. burnetii* infections.

SIGNIFICANCE

Coxiellae are enigmatic intracellular bacteria that adversely affect human and animal health, but their evolutionary origins and intracellular biology are not clearly understood. Here, by sequencing the first genome of a soft-tick endosymbiont, and combining this information with phylogenetic and phylogenomic analyses, we show that endosymbiotic coxiellae evolved from pathogenic ancestors and that the human pathogen *Coxiella burnetii* evolved from a pre-existing pathogen—not from an avirulent tick endosymbiont as previously assumed. Additionally, having the genome of a closely related nonpathogen allowed us, for the first time, to perform in-depth comparative genomic analyses, which identified several metabolic processes that are likely critical to *C. burnetii’*s intracellular growth and virulence. Knowledge gained from this study, in addition to helping us better understand the evolution of coxiellae, should hasten the development of novel therapies to control Q fever and could be applied to controlling the spread of ticks.

## Introduction

A bacterium’s genome size and gene content signal both the degree of its dependence on the host and the length of the bacterium–host relationship. For example, a bacterium that has established a long-term, obligate symbiosis would have a tiny genome filled with protein-coding genes (Wernegreen et al. 2000). Conversely, the genome of a bacterium that is in the early stages of symbiosis is usually large and contains numerous pseudogenized genes, which, as the relationship progresses, would eventually be lost, resulting in a tiny genome (Moran 2002; McCutcheon and Moran 2011). The genomes of *Coxiella*-like endosymbionts (CLEs) found in ticks (Acari: Ixodida) fall into both categories: Some ticks, for example, *Amblyomma americanum* and *A. sculptum*, contain small-genomed CLEs (∼0.6 Mbp) that have few pseudogenes, indicating that they represent an ancient lineage of tick endosymbionts (Smith et al. 2015). In contrast, CLEs in *Rhipicephalus turanicus* (CRt), and *R. sanguineus* have large genomes (∼1.7 Mbp) filled with pseudogenes, denoting that the bacteria are in the early stages of symbioses (Gottlieb et al. 2015; Tsementzi et al. 2018). While most ticks contain CLEs, a few have *Francisella*-like endosymbionts (FLEs) (Gerhart et al. 2016, 2018; Duron et al. 2017). All FLEs studied to date have large genomes (∼1.5 Mbp) with hundreds of pseudogenes, including inactivated virulence genes, indicating that FLEs evolved recently from pathogenic ancestors (Gerhart et al. 2016, 2018; Duron et al. 2018). Irrespective of their age, CLEs and FLEs improve the reproductive fitness of their hosts by likely providing metabolites missing in vertebrate blood, ticks’ sole nutritional source (Gottlieb et al. 2015; Smith et al. 2015; Gerhart et al. 2016, 2018; Duron et al. 2017, 2018; Tsementzi et al. 2018).

*Coxiella burnetii*, the causative agent of human Q fever, has also been detected in ticks; in fact, the intracellular pathogen was first isolated from hard ticks *Dermacentor andersoni* and *Haemaphysalis humerosa* (Cox 1938; Smith and Derrick 1940). In addition, transstadial transmission and fecal excretion of *C. burnetii* occur in laboratory-raised ticks (Eldin et al. 2017; Körner et al. 2020). However, it is not clear whether ticks play any meaningful role in the natural spread of *C. burnetii* (Duron et al. 2015b); instead, Q fever generally occurs following inhalation of *C. burnetii*-contaminated aerosols originating from infected farm animals (Maurin and Raoult 1999; Eldin et al. 2017). Within the human lungs, *C. burnetii* infects alveolar macrophages and generates a large replicative vacuole, termed the *Coxiella*-containing vacuole (CCV), by subverting host responses through a Dot/Icm Type IVB secretion system (T4BSS). This secretion system is essential to the pathogenicity of both *C. burnetii* and *Legionella pneumophila*, the two established pathogens in the order Legionellales (Segal et al. 1999; Chen et al. 2010; Beare et al. 2011; Newton et al. 2014; Burstein et al. 2016). Genes for T4BSS, which evolved from conjugation machinery, have spread across the bacterial kingdom via horizontal gene transfer (HGT), a process through which organisms gain foreign genes, allowing them to quickly adapt to a new environment (Ochman et al. 2000; Lerat et al. 2005).

The closest relatives of *C. burnetii* are CLEs present in ticks (Almeida et al. 2012; Duron et al. 2015a; Smith et al. 2015), leading to the notion that the human pathogen emerged when an avirulent tick endosymbiont gained pathogenicity genes, probably via HGT (Duron et al. 2015a; Gerhart et al. 2016). Contrary to this hypothesis, by sequencing the genome of a CLE in *Ornithodoros amblus* (henceforth referred to as CLEOA), we show that a common virulent ancestor gave rise to both *C. burnetii* and CLEOA. The potentially pathogenic ancestor contained genes for most virulence factors, including T4BSS, indicating that the erstwhile bacterium was likely capable of infecting mammalian macrophages. In CLEOA, homologs of most virulence-associated genes have been rendered nonfunctional, but genes for B vitamin and cofactor biosynthesis have been retained, suggesting that a virulent bacterium has morphed into a nutrient-provisioning tick endosymbiont. In a similar fashion, we found that several other tick endosymbionts likely evolved from pathogenic ancestors, indicating that pathogen-to-endosymbiont transformation is widespread across ticks. Finally, by inhibiting *C. burnetii* growth using a synthetic analog of heme, a metabolite produced by *C. burnetii* but not by CLEOA, we demonstrate how knowledge gained through comparative genomics could be applied to developing novel strategies to control Q fever, which is difficult to treat with currently available antibiotics.

## Results

### CLEOA Arose from a Pathogenic Ancestor

**Fig. 1.**
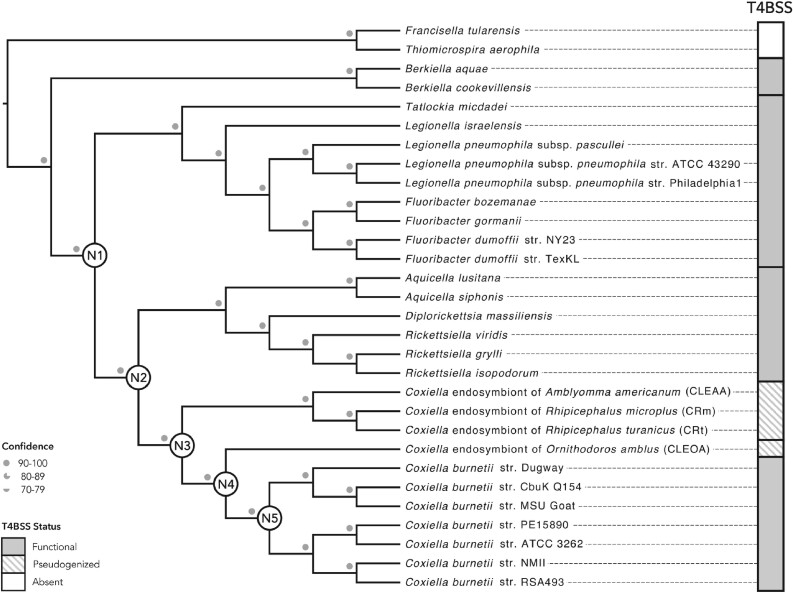
CLEOA is the closet relative of *Coxiella burnetii*. Maximum likelihood and Bayesian trees built using 117 single-copy protein-coding genes were combined to generate the shown phylogenomic tree. Bootstrap support and posterior probabilities agreed at all branchpoints and are depicted as a single confidence value. The Dot/Icm Type IVB secretion system (T4BSS), which is critical to pathogenesis, is found in all members of the order Legionellales, but has been pseudogenized in CLEs. Nodes N1–N5 mark major branching points in the evolution of *C. burnetii*.

**Fig. 2.**
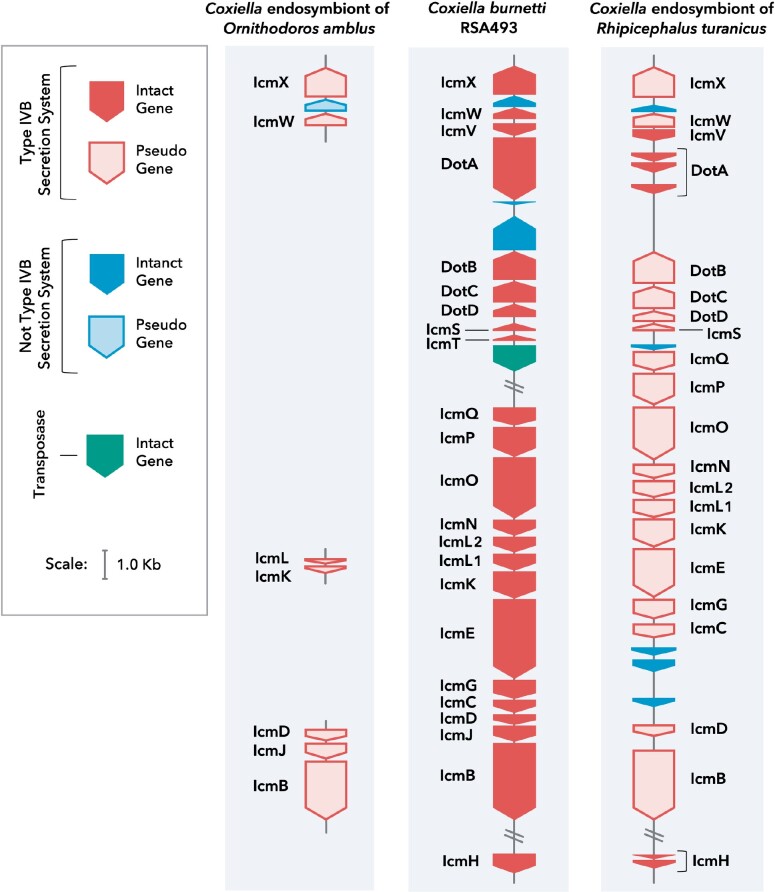
CLEs contain nonfunctional T4BSS. Comparison of T4BSS genes in CLEOA, *Coxiella burnetii* RSA493 and CRt indicate that the secretion system has been rendered nonfunctional in tick endosymbionts. Filled blocks represent intact genes. Outlined blocks represent pseudogenized genes.

Phylogenetic trees based mainly on 16S rDNA have previously indicated that the closest relatives of *C. burnetii* are CLEs present in *Ornithodoros* and *Argas* soft ticks (family Argasidae) (Almeida et al. 2012; Duron et al. 2015a; Smith et al. 2015); however, all CLE genomes available to date are from CLEs in hard ticks (family Ixodidae) (Gottlieb et al. 2015; Smith et al. 2015; Guizzo et al. 2017; Tsementzi et al. 2018), stymieing earlier efforts to understand *C. burnetii* evolution. Here, by sequencing the first soft-tick CLE genome, we were able to build a more definitive phylogenomic tree, which confirmed that CLEOA is a sister taxon of *C. burnetii* (fig. 1; supplementary table S1, Supplementary Material online). In addition, the presence of pseudogenized T4BSS genes in CLEOA indicates that the tick-symbiont evolved from a pathogenic ancestor with a functional T4BSS (fig. 2; supplementary table S2, Supplementary Material online).

To resolve the ancestry of pathogenicity in Legionellales, we determined the prevalence of T4BSS, which is an essential virulence factor in this group of bacteria that includes human pathogens (*C. burnetii* and *L. pneumophila*), opportunistic pathogens (*Rickettsiella*), and symbionts (CLEs). Our analyses revealed that the secretion system is intact in all members of this order with the exception of CLEs, which only contained remnants of the T4BSS (figs. 1 and 2). The most parsimonious explanation for this phyletic pattern is that T4BSS was present in the common ancestor of all Legionellales and was later lost in lineages that gave rise to CLEs, including CLEOA.

### Multiple CLEs Have Evolved from Pathogens

**Fig. 3.**
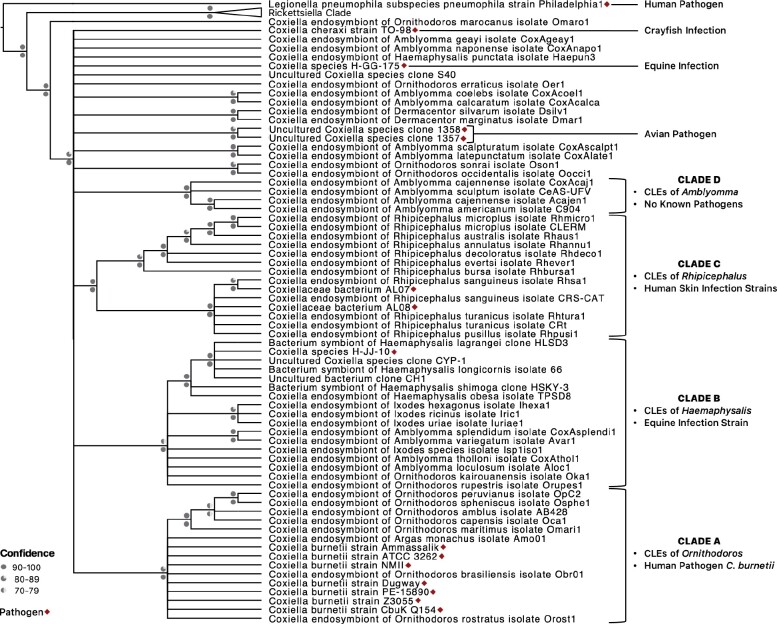
CLE clades contain both tick endosymbionts and pathogens. A 16S rDNA-based phylogenetic tree is shown. Bootstrap support and posterior probabilities are labeled above and below branchpoints, respectively. Nodes with ≤70% bootstrap support were collapsed to polytomies. Taxa colors represent the continent from which the host was derived. Established pathogens are marked with asterisks. Clades A–D were originally defined by Duron et al. (2015a).

*Coxiella* detected in ticks are classified into four clades, three of which contain intermingled pathogens and endosymbionts (fig. 3; supplementary table S3, Supplementary Material online; Duron et al. 2015a). Clade A includes *C. burnetii*—the human pathogen, and CLEOA, which arose from a pathogenic ancestor, as discussed above. In Clade B, CLEs of *Haemaphysalis* ticks are present along with a presumably pathogenic *Coxiella* that caused horse infection (Seo et al. 2016). Clade C has CRt, a pathogen-derived endosymbiont, along with strains that caused opportunistic human skin infections (Gottlieb et al. 2015; Angelakis et al. 2016; Guimard et al. 2017; Tsementzi et al. 2018; Ben-Yosef et al. 2020). Only Clade D, which contains small-genomed CLEs (e.g., CLEAA), has no known pathogenic representatives. This phylogenetic pattern of endosymbionts clustering with pathogens indicate that, similar to the pathogenic ancestry of CLEOA and CRt, CLEs of several other ticks have also evolved from pathogenic coxiellae. Thus, based on phylogenetic and T4BSS distribution patterns, we surmise that *Coxiella* strains that infect vertebrates (e.g., humans, horses, and birds) and invertebrates (e.g., crayfish) are widespread across the globe (fig. 3), and many of them have evolved into tick endosymbionts.

### HGT Was a Major Contributor to Gene Accumulation in *C. burnetii’*s Ancestors

**Fig. 4.**
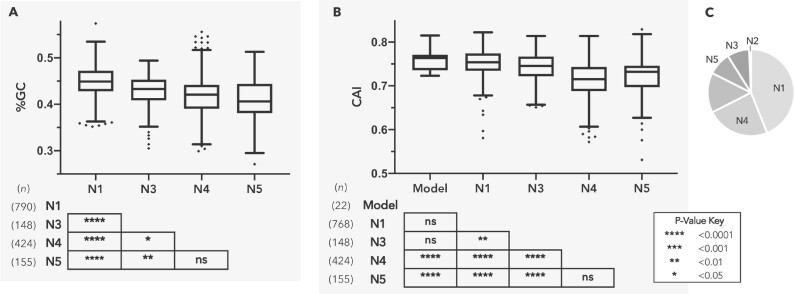
HGT was a major contributor to gene accumulation in *C. burnetii’*s ancestors. Nodes N1–N5 (as labeled in fig. 1) represent major branchpoints in the evolution of *C. burnetii*. (A and B) depict %GC and Codon Adaptation Index (CAI) distributions, respectively, for genes originating at each node. Boxes illustrate each distribution’s interquartile range while the black line dividing the box represents the median. Whiskers represent minimum and maximum values, excluding outliers (black diamonds) which were determined using the Tukey method. *P*-values shown in tables are for pairwise T-tests (pooled SD, BKY adjusted). Genes gained at N2 were excluded due to small sample size (*n *=* *13). (C) *Coxiella burnetii* genome composition based on nodes of gene origin: N1: 43.9%, N2: 0.7%, N3: 8.2%, N4: 22.6%, N5: 8.6%. The unlabeled portion represents potentially spurious genes (*n *=* *224) as well as genes with undefined nodes of origin (*n *=* *44).

In order to better understand the evolution of *C. burnetii*, we traced the ancestry of its protein-coding genes by determining whether their orthologs—either functional or pseudogenized—were present in other Legionellales members. Out of 1,530 protein-coding genes whose ancestries we could trace, 790 were deemed to be ancestral, meaning it was present in the ancestor that diverged from *Legionella* (Node 1), and an additional 585 genes originated in Nodes 2–4 (Fig. 4; supplementary table S4, Supplementary Material online). These data demonstrate that the common ancestor of *C. burnetii* and CLEOA contained most of the genes, including virulence factors, present in *C. burnetii*, and was hence well equipped to infect mammals.

A major impediment to unspooling the evolutionary history of *C. burnetii* is the sparse availability of Coxiellaceae genomes, which makes it difficult to ascertain whether genes were gained by *C. burnetii’*s ancestors at Nodes 2–5 or were instead lost in other bacteria represented at each node. To overcome this difficulty, we calculated each *C. burnetii* gene’s nucleotide composition (%GC) and Codon Adaptation Index (CAI), two measures known to distinguish foreign-origin genes from ancestral ones (fig. 4; supplementary table S5, Supplementary Material online; Sharp and Li 1987; Lawrence and Ochman 1997; Jansen et al. 2003; Raghavan et al. 2012). Both %GC and CAI values for genes that originated in Nodes 3–5 were significantly different from those of ancestral (Node 1) genes, indicating that a considerable portion of these genes were likely acquired horizontally. [Node 2 genes were excluded from this analysis due to the small sample size (*n *=* *13).] Interestingly, %GC and CAI values for Node 5 genes were not significantly different from those gained at Node 4, suggesting that many of the genes currently found only in the human pathogen were present in the common ancestor of *C. burnetii* and CLEOA, and were later lost in the tick endosymbiont. However, it is clear that HGT has contributed to the accumulation of genes at Node 5 as well because 88 out of 155 genes in this category showed phylogenetic patterns consistent with HGT (supplementary fig. S1, Supplementary Material online; supplementary table S6, Supplementary Material online). Cumulatively, our results validate the important role HGT has played in assembling *C. burnetii’*s protein repertoire (Moses et al. 2017), and show that this process occurred principally in ancestors that preceded the *C. burnetii*-CLEOA split.

### CLEOA Potentially Provides *O. amblus* with Nutrients Missing in Vertebrate Blood

**Fig. 5.**
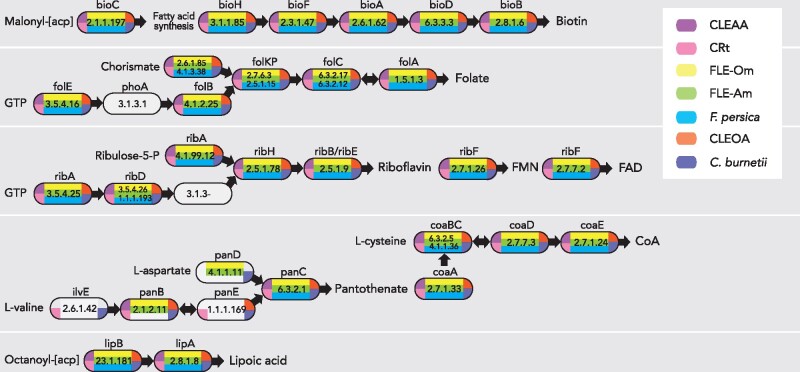
CLEs and FLEs encode B vitamin and cofactor biosynthetic pathways. CLEOA, CRt, CLEAA, FLE-Om (*Francisella* endosymbiont of *Ornithodoros moubata*; LVCE00000000), FLE-Am (*Francisella* endosymbiont of *Amblyomma maculatum*; LNCT00000000), and *Francisella persica* (FLE in *Argas arboreus*; NZ_CP013022) contain intact pathways for the synthesis of B vitamins and cofactors. Enzymes catalyzing each step are labeled with gene names and EC numbers. Blocks with no color indicate that a functional copy of a gene was not detected in that genome. Depictions of metabolic pathways modified from Gerhart et al. (2018).

Similar to other hematophagic organisms (Duron and Gottlieb 2020), ticks likely obtain nutrients missing in blood from endosymbiotic bacteria such as CLEs and FLEs (Gottlieb et al. 2015; Smith et al. 2015; Gerhart et al. 2016, 2018; Duron et al. 2018; Tsementzi et al. 2018). In accordance with this idea, although CLEOA has lost a large number of genes (table 1), it has retained complete pathways for the synthesis of several B-vitamins and cofactors (fig. 5). Interestingly, these pathways are also present in *C. burnetii*, indicating that the genes are of ancestral origin and could be critical to the intracellular growth of both the endosymbiont and the human pathogen.

**Table 1:** Genome Characteristics of CLEOA, *Coxiella burnetii*, and CRt

	CLEOA	*Coxiella burnetii* RSA493	CRt
NCBI accession	VFIV00000000	AE016828.3	NZ_CP011126.1
Length (bp)	1,561,173	1,995,488	1,733,840
%GC	40.6	42.7	38.2
rRNA (5S, 16S, 23S)	1,1,1	1,1,1	1,1,1
tRNA	42	42	48
Functional genes	889	1,798	926
Pseudogenes	660	197	383
Single copy genes^a^	106/111	106/111	105/111

a Albertsen et al. (2013).

CLEOA also contains 91 genes that are absent or have been deactivated in *C. burnetii* (supplementary table S7, Supplementary Material online). It is likely that many of these CLEOA-specific genes have functions in the tick ecosystem but are not useful during mammalian infections. Collectively, based on its genomic features (table 1), recent loss of T4BSS (fig. 2), and presence of intact pathways for synthesizing B vitamins and cofactors (fig. 5), we conclude that a pathogenic *Coxiella* was recruited to function as a nutrient-provisioning endosymbiont in *O. amblus*.

### CLEOA Has Not Retained Genes that Allow *C. burnetii* to Withstand the Harsh Environs of CCV

#### Loss of T4BSS and Associated Genes

Because *C. burnetii* diverged from CLEOA recently, it provided us an opportunity to perform a thorough comparison of the human pathogen’s genome to that of a closely related nonpathogen. Our analysis identified 980 functional genes in *C. burnetii* whose orthologs have been either pseudogenized or deleted in CLEOA (supplementary table S8, Supplementary Material online). Among them are genes for T4BSS and for effectors secreted through T4BSS (fig. 2; supplementary table S2, Supplementary Material online) that enable *C. burnetii* to generate its intracellular replicative niche, termed *Coxiella*-containing vacuole (CCV) (Chen et al. 2010; Beare et al. 2011; Martinez et al. 2014, 2020; Newton et al. 2014). In addition, genes for PmrAB and EirA that control T4BSS activity, and for eight T4BSS effectors present on *C. burnetii’*s QpH1 plasmid have been inactivated in CLEOA (Maturana et al. 2013; Beare et al. 2014; Kuba et al. 2020). Therefore, the secretion system, which is critical to the intramacrophage growth of *C. burnetii*, is clearly not required for CLEOA to grow within tick cells.

#### Loss of Transporters of Antibacterial Molecules

To protect itself from noxious molecules produced by the host, *C. burnetii* likely depends on transport proteins that efflux harmful substances out of its cytoplasm. For instance, macrophages increase Cu^2+^ concentration within phagosomes to kill intracellular bacteria (Neyrolles et al. 2015), and *C. burnetii* probably utilizes a P-1B type ATPase to export copper from its cytoplasm to sustain intracellular growth (Rowland and Niederweis 2012). This ATPase, along with 18 others of unknown function, has been pseudogenized or deleted in CLEOA (supplementary table S9, Supplementary Material online). In addition, *C. burnetii* encodes 25 putative transporters that could facilitate the pathogen’s growth within CCV, out of which, 19 have become nonfunctional in CLEOA (supplementary table S9, Supplementary Material online), suggestive of the mild nature of the tick endosymbiont’s intracellular compartment where antibacterial molecules are probably not a major threat.

#### Diminished pH Regulation

A defining feature of CCV is its acidity (pH ∼4.75) (Vallejo Esquerra et al. 2017; Samanta et al. 2019). To compensate, *C. burnetii* utilizes several mechanisms to maintain its cytoplasmic pH close to neutral (Hackstadt 1983), many of which have been rendered nonfunctional in CLEOA. For example, the enzyme carbonic anhydrase that catalyzes the production of bicarbonate (HCO^3−^) buffer from CO_2_ (Bury-Moné et al. 2008; Vallejo Esquerra et al. 2017) has been pseudogenized in CLEOA (supplementary table S10, Supplementary Material online). Another pH-regulating strategy used by *C. burnetii* is to remove excess protons in its cytoplasm via proton-antiporters such as the multiprotein Mrp antiporter, a pair of Na^+^/H^+^ antiporters, and a K^+^/H^+^ antiporter. *C. burnetii* also encodes a pair of glutamate/gamma-aminobutyrate (GABA) antiporters that export GABA in exchange for glutamate, thereby reducing the cytoplasmic proton content. The Mrp antiporter, one of the two Na^+^/H^+^ antiporters, and both glutamate/GABA antiporters have been pseudogenized in CLEOA (supplementary table S10, Supplementary Material online). A hallmark of *C. burnetii* is its unusually high number of basic proteins (∼46% of proteins have pI values ≥9; average pI 8.22) that could function as a “proton sink,” which allows the pathogen to maintain its cytoplasmic pH close to neutral (Seshadri et al. 2003). In contrast, only ∼39% of proteins in CLEOA have pI values ≥9 (average pI 8.0), again illustrating a lack of acidic stress within CLEOA’s intracellular vacuole. Collectively, our data suggest that the endosymbiont does not face the constant threat of excess protons entering its cytoplasm, probably because its intracellular niche, unlike *C. burnetii’*s, has a pH closer to neutral.

#### Loss of Cell Membrane and Cell Wall Genes

In gram-negative bacteria, inner and outer membranes along with peptidoglycan play important roles in stress response (Rowlett et al. 2017). In CLEOA, the gene that encodes PlsC, which converts lysophosphatidic acid into phosphatidic acid (PA), a universal intermediate in the biosynthesis of membrane phospholipids, has been pseudogenized. The *plsC* gene is essential in *Escherichia coli*, and a transposon insertion in this gene in *C. burnetii* caused severe intracellular growth defect (Coleman 1990; Martinez et al. 2014); hence, it is not clear how CLEOA is able to build its membranes without a functional *plsC*, but one possibility is that the endosymbiont utilizes PA obtained from its host. Another membrane-associated loss of function in CLEOA is the pseudogenization of the *pldA* gene that encodes phospholipase A (PldA), which is critical to *C. burnetii’*s outer membrane function and for optimal growth within macrophages (Stead et al. 2018). As for its peptidoglycan, CLEOA contains intact genes for D,D-transpeptidases (also known as penicillin-binding proteins) that catalyze 4-3 peptide cross-links between D-alanine and diaminopimelate; however, all L,D-transpeptidase genes (annotated as ‘enhanced entry proteins’) have been pseudogenized, indicating that the tick symbiont does not have the ability to generate 3–3 cross-links between diaminopimelate molecules in its peptidoglycan. These nonclassical cross-links contribute to *C. burnetii’*s environmental stability (Sandoz et al. 2016) and are probably not critical to CLEOA because the endosymbiont is passed vertically from one generation to next. Collectively, as observed in other endosymbionts (Nakabachi et al. 2006; McCutcheon and Moran 2010; Chong and Moran 2018), CLEOA lacks numerous proteins that are typically considered integral to the optimal functioning of bacterial cell membrane and cell wall.

#### Loss of Antioxidant Genes

An intricate network of antioxidants allows *C. burnetii* to thrives in a phagolysosome-derived intracellular vacuole (Mertens and Samuel 2012). In contrast to CCV, oxidative stress appears to be minimal in CLEOA’s intracellular vacuole because the endosymbiont contains only a streamlined version of *C. burnetii’*s antioxidant defense system. For instance, *C. burnetii* contains two superoxide dismutases (SODs), but CLEOA has retained the cytoplasmic Fe-containing SodB, but not SodC, the periplasmic Cu/Zn-SOD. OxyR, the master regulator of peroxide stress, along with a catalase, a peroxidase (AhpC2), a methionine sulfoxide reductase, a hemerythrin-like protein, and a glutathione transferase that together help mitigate oxidative stress have also been deactivated in CLEOA (supplementary table S11, Supplementary Material online). In addition, *C. burnetii*, but not CLEOA, has the ability to synthesize queuine, a guanine analog, found in the first anticodon position of several post-transcriptionally modified tRNAs (Iwata-Reuyl 2003). The precise functions of queuine is not understood, but it is thought to promote the activity of antioxidant enzymes, including catalase, superoxide dismutase, and glutathione transferase, most of which, as mentioned above, have lost their functionality in CLEOA (Koh and Sarin 2018).

*Coxiella burnetii* utilizes both cytochrome bd (encoded by genes *cydABX*) and cytochrome o (encoded by genes *cyoABCD*) as terminal oxidases, but CLEOA has only retained cytochrome o genes. Cytochrome bd, which also functions as a quinol peroxidase that prevents the buildup of oxidative free radicals (Endley et al. 2001; Omsland and Heinzen 2011), has become nonfunctional in the tick endosymbiont. In addition, CLEOA does not encode genes for an acid phosphatase and two sterol reductases that likely modify host proteins and cholesterol, respectively, to protect *C. burnetii* from host-induced oxidative stress (Seshadri et al. 2003; Gilk et al. 2010; Hill and Samuel 2011; Gilk 2012). Finally, *C. burnetii* is thought to compensate for the lack of the oxidative branch of pentose phosphate pathway (PPP)—a major source of NADPH, by utilizing alternative NADPH-regenerating enzymes such as short-chain dehydrogenases and sterol reductases, and by salvaging NAD^+^ from the host (Bitew et al. 2018, 2020). In CLEOA, all four short-chain dehydrogenases, the two eukaryote-like sterol reductases, and the nicotinate-salvaging protein have become nonfunctional. In total, while the human pathogen contains several mechanisms to defend against oxidative stress, most of these antioxidant systems have been lost in CLEOA, most likely due to minimal oxidative stress experienced by the bacterium within tick cells. Collectively, the loss of T4BSS, transporters, pH regulation, cell wall modification, and antioxidant defense in CLEOA show that its intracellular vacuole is a less stressful place to live than the phagolysosome-derived CCV occupied by *C. burnetii*.

### Heme Analog Inhibits *C. burnetii* Growth

Cytochromes require heme as a cofactor, but CLEOA does not contain a functional heme biosynthesis pathway, which is present in *C. burnetii* (supplementary table S12, Supplementary Material online). The only intact heme biosynthesis gene in CLEOA is *ctaB*, which encodes an enzyme that converts heme b to heme o, a component of cytochrome o—the sole terminal cytochrome oxidase present in CLEOA (Saiki et al. 1992). Based on this evidence, the endosymbiont appears to import heme b from the tick hemocoel (vertebrate hemoglobin contains heme b) and converts it to heme o using the *ctaB*-encoded protoheme IX farnesyltransferase. Additionally, while *C. burnetii* can import ferrous iron released from iron-containing host molecules such as ferritin and transferrin (Sanchez and Omsland 2020), free Fe^2+^ does not seem to be important for CLEOA’s intracellular growth because the iron transporter FeoB has been pseudogenized, suggesting that host-derived heme b serves as the tick endosymbiont’s heme and iron source.

**Fig. 6.**
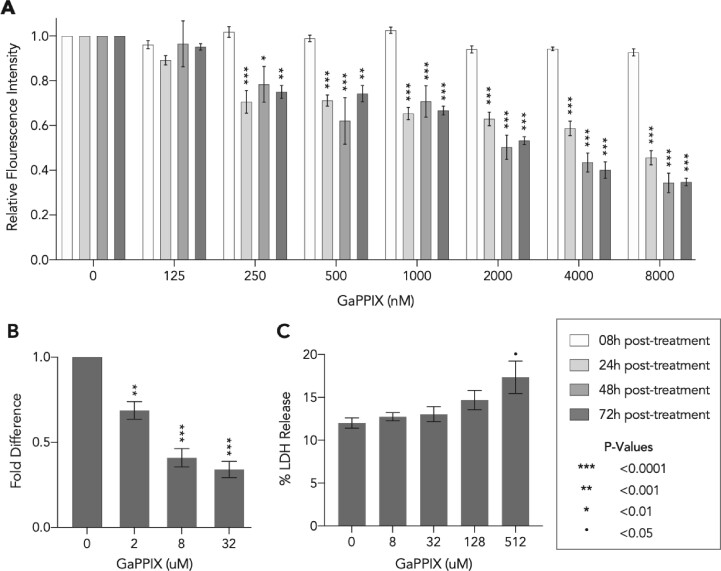
A heme analog reduces *Coxiella burnetii* growth. (A) Bacteria growing in ACCM-2 were exposed to concentrations of GaPPIX shown in x-axis and were quantified using PicoGreen at 8 h, 24 h, 48 h, and 72 h post-treatment. Data shown are mean fluorescence intensity (± SE) compared to the vehicle control (0 nM). Statistical significance was analyzed using two-way repeated measures ANOVA followed by Dunnett's test (*n* = 5). (B) At 72 h post-treatment, bacterial growth within THP-1 cells was quantified using qPCR. Data shown as mean fold difference (±SE) compared to control. (C) At 24 h post-GaPPIX treatment of THP-1 cells, lactate dehydrogenase (LDH) activity was determined by measuring the level of resorufin formation using an LDH cytotoxicity assay. The cytotoxicity was reported as the percentage LDH released compared to the maximum LDH activity. Data shown as mean percentage LDH released (±SEM). For both (B and C), statistical significance was analyzed using one-way ANOVA followed by Dunnett's test (*n* = 3).

The heme biosynthesis pathway, while absent in CLEOA, is conserved in all strains of *C. burnetii*, probably because the iron-protoporphyrin molecule is critical to the pathogen’s ability to grow within human macrophages (Moses et al. 2017). We tested *C. burnetii’*s dependence on heme by treating both axenically grown and intracellular *C. burnetii* with gallium protoporphyrin IX (GaPPIX), which can replace heme in cytochromes and other heme-containing enzymes (Hijazi et al. 2017, 2018). As shown in figure 6, ≥250 nM of GaPPIX caused significant inhibition of *C. burnetii* growth in ACCM-2, and treatment with ≥2 µM of GaPPIX resulted in significant growth impairment of *C. burnetii* within THP-1 cells. Reassuringly, only GaPPIX concentrations of ≥512 µM caused cytotoxicity in THP-1 cells (fig. 6*C*), indicating that gallium compounds could potentially be used to treat *C. burnetii* infections.

## Discussion

Although symbiotic and pathogenic coxiellae associated with ticks are found across the globe, it is not clear how pathogenesis and symbiosis evolved in this group of bacteria. Here, we show that CLEOA, a soft-tick symbiont, and *C. burnetii*, a human pathogen, evolved recently from a common ancestor that contained genes necessary to infect macrophages. Additionally, while HGT contributed significantly to the evolution of *C. burnetii*, it occurred in ancestors prior to the divergence of CLEOA and *C. burnetii* lineages. These discoveries clarify the evolution of *C. burnetii*, which previously was thought to have evolved from an avirulent tick endosymbiont by gaining virulence factors via HGT. We further show that the evolution of *C. burnetii* and CLEOA fits into a general pattern of tick-associated coxiellae originating from pathogens, thereby revealing that CLEs, as described previously for FLEs, originated from pathogenic ancestors. Lastly, by comparing the genomes of *C. burnetii* and CLEOA, we were able to gain new insights into the intracellular biology of both bacteria and show that metabolic pathways retained only in the human pathogen are promising targets for the development of new treatments against Q fever.

### Emergence of Tick-Symbionts from Virulent Ancestors

*Coxiella* species related to CLEs infect a wide range of animals (Shivaprasad et al. 2008; Woc-Colburn et al. 2008; Angelakis et al. 2016; Seo et al. 2016; Guimard et al. 2017; Elliman and Owens 2020; Needle et al. 2020), but these infectious strains are not the closest relatives of *C. burnetii*; instead, the human pathogen’s closest relative is the soft-tick symbiont CLEOA. Akin to the CLEOA–*C. burnetii* relationship, CRt, the endosymbiont in *R*. *turanicus*, is closely related to pathogenic *Coxiella* (termed “*Candidatus* Coxiella massiliensis”) isolated from human skin infections, and a strain of *Coxiella* isolated from horse blood is closely related to CLEs present in *Haemaphysalis* ticks (Angelakis et al. 2016; Seo et al. 2016; Guimard et al. 2017). In addition to these pathogens, bacteria related to CLEs have repeatedly caused fatal bird and crayfish infections (fig. 3; Shivaprasad et al. 2008; Woc-Colburn et al. 2008; Elliman and Owens 2020; Needle et al. 2020). Microscopic and histological data from avian infections demonstrated that the bacteria have the ability to generate CCV-like compartments within macrophages, and both avian and human skin infection strains have “small-cell” and “large-cell” morphologies—two distinct characteristics of *C. burnetii*—suggesting that the bacteria are genuine vertebrate pathogens (Shivaprasad et al. 2008; Woc-Colburn et al. 2008; Angelakis et al. 2016; Guimard et al. 2017; Needle et al. 2020). Further research, including sequencing their genomes, is required to elucidate the biology and pathogenicity of these infective strains and to understand why only one, that is, *C. burnetii*, among several virulent lineages have evolved into a bona fide human pathogen.

### Tick Endosymbionts are Ephemeral

Phylogenies of only a few CLEs are congruent with those of their hosts (Duron et al. 2017; Binetruy et al. 2020), probably because older CLEs get replaced by newer CLEs derived from distantly related coxiellae. In a similar fashion, FLEs seem to have replaced older CLEs in several tick lineages (Gerhart et al. 2016, 2018; Duron et al. 2017, 2018). This ephemeral nature of CLEs is surprising because hematophagic arthropods typically need a reliable partner to gain nutrients that are in short supply in vertebrate blood (Duarte et al. 1999; Sterkel et al. 2017; Duron and Gottlieb 2020). Insects such as bedbugs and body lice that face similar nutrient scarcity have evolved stable long-term relationships with endosymbionts (Perotti et al. 2007; Hosokawa et al. 2010). It is not clear why that is not the case in ticks, but one possibility is that ticks do not need to establish long-term relationships because they frequently encounter pathogenic bacteria that are predisposed to becoming nutrient-provisioning endosymbionts. Another reason for the unstable nature of CLE-tick relationships could be that the constant turnover of endosymbionts protects ticks from being dependent on an endosymbiont with reduced nutrient-provisioning capability (Russell et al. 2017; Bennett and Moran 2015). Gaining new symbionts via horizontal transmission could also protect ticks from becoming dependent on a degraded endosymbiont. While the mechanistic details of this process are not understood, phylogenetic patterns of CLE and FLE distribution strongly indicate the occurrence of horizontal transmission of bacteria between ticks (Gerhart et al. 2016, 2018; Duron et al. 2017; Binetruy et al. 2020). It should be noted however that not all tick endosymbionts are short-lived. Ticks that carry CLEs belonging to Clade D (fig. 3) appear to have established long-term relationships with their endosymbionts. For instance, CLEs in *A. americanum* and *A. sculptum* have highly reduced (∼0.60 Mbp) genomes that are similar in size to *Buchnera*, which established its symbiosis with aphids more than 200 mya (Moran et al. 1993). Putting all this information together, it appears that a combination of vertical inheritance, horizontal transmission, and periodic replacement of old symbionts with new pathogen-derived symbionts underlies the complex distribution pattern of endosymbionts observed in ticks (Gottlieb et al. 2015; Smith et al. 2015; Gerhart et al. 2016, 2018; Duron et al. 2017; Tsementzi et al. 2018; Binetruy et al. 2020).

### Functions of CLEs and FLEs

While the exact functions of FLEs and CLEs have not been fully characterized, previous studies have shown that they infect tick ovaries and are often the predominant bacterium present in long-term laboratory tick colonies, an indication that the bacteria are vertically transmitted and are essential to ticks’ wellbeing (Reinhardt et al. 1972; Klyachko et al. 2007; Smith et al. 2015; Gerhart et al. 2016, 2018; Buysse et al. 2019). In addition, removal of the resident endosymbiont via antibiotic treatment reduced tick fitness, which was reversed when ticks were provided with B vitamins (Zhong et al. 2007; Smith et al. 2015; Gerhart et al. 2016; Guizzo et al. 2017; Zhang et al. 2017; Duron et al. 2018; Li et al. 2018; Ben-Yosef et al. 2020). Our genome analyses support a nutrient-provisioning role for tick endosymbionts because the genes required to synthesize several B vitamins and cofactors are conserved in all CLEs and FLEs. Future experiments should clarify whether any or all of these nutrients form the basis for the CLE-tick and FLE-tick symbioses.

### Pathogen-Specific Metabolic Processes are Potential Targets to Control Q Fever

Genetic and physiological capabilities accumulated by a bacterium are critical to its ability to adapt to new environments, especially ones such as intramacrophage vacuoles that do not facilitate the gain of new genes via HGT. In accordance with this idea, our analyses showed that virulence factors and metabolic genes utilized by *C. burnetii* to grow within CCV were present in the common ancestor of *C. burnetii* and CLEOA. Befitting its obligate endosymbiotic lifestyle, many of these genes have become nonfunctional in CLEOA, allowing us to identify metabolic processes that are likely critical to *C. burnetii’*s intracellular growth. One metabolite that is exclusively produced by the pathogen is heme, the iron-protoporphyrin required for oxidative phosphorylation, among other functions. To test the importance of heme to *C. burnetii*, we exposed the bacterium to GaPPIX, a Ga(III) complex of protoporphyrin IX. Ga(III) inhibits bacterial growth because it binds to biological complexes that normally binds to Fe(III), but under physiological conditions Ga(III) is not reduced to Ga(II), thereby disrupting essential redox-driven biological processes (Bernstein 1998). We chose GaPPIX over other gallium-based formulations because it could replace heme in cytochromes, is known to be bactericidal, and is not toxic to primary human fibroblasts and established cell lines (Stojiljkovic et al. 1999; Arivett et al. 2015; Hijazi et al. 2018). *Coxiella burnetii* lacks homologs of known heme transporters (Moses et al. 2017), hence, it is not clear how GaPPIX enters into the pathogen, but our growth assays clearly demonstrated that the heme analog is very effective at inhibiting both axenic and intracellular growth of *C. burnetii* (fig. 6). Encouragingly, a recent human trial showed that Ga could improve lung function in people with cystic fibrosis and chronic *Pseudomonas aeruginosa* lung infections, and that the molecule worked synergistically with other antibiotics to inhibit bacterial growth (Goss et al. 2018). Although further work is required to gauge its impact on human microbiome, Ga, which has been approved by FDA for intravenous administration (Bonchi et al. 2014), and its derivatives such as GaPPIX, hold great promise as new therapeutic tools.

## Materials and Methods

### Genome Sequencing and Assembly

An *O. amblus* female, collected from soil underneath rocks near a *Spheniscus humbolti* (Humboldt penguin) nesting area in Isla Grande de Atacama, Chile, was identified as described in Clifford *et al.* (1980). DNA was extracted from the tick using DNeasy Blood & Tissue kit (Qiagen) and was submitted to Yale Center for Genome Analysis for Illumina (NovaSeq) sequencing. The resulting 150 bp paired-end reads were trimmed using Trimmomatic resulting in approximately 220 million read pairs of suitable quality (Bolger et al. 2014). The reads were assembled into contigs using metaSPAdes (Nurk et al. 2017), and open reading frames (ORFs) were identified using Prodigal (Hyatt et al. 2010). RNammer (Lagesen et al. 2007) was used to identify ribosomal RNA in all contigs and sequencing coverage values were used to determine the relative abundance of bacteria: 88.5% *Coxiella*, 4.6% *Alkalihalobacillus*, 3.8% *Sporosarcina*, and 3.1% *Oceanobacillus*.

Contigs containing *Coxiella* genes were tentatively identified using BLASTn and BLASTp by comparing to a database of all publicly available sequences from Coxiellacea members. CONCOCT (Alneberg et al. 2014) was used for binning contigs based on coverage and k-mer composition, and these findings were merged with BLAST-based binning results. Approximately 20 million paired reads that mapped to contigs identified as containing *Coxiella* genes were used for a final metaSPAdes assembly resulting in a total of 101 contigs. The final collection of contigs was verified using hmmsearch (Potter et al. 2018) to identify essential single-copy genes (Albertsen et al. 2013), as well as RNammer (Lagesen et al. 2007) and tRNAscan-SE (Chan and Lowe 2019) to identify ribosomal and transfer RNAs, respectively. We were unable to stitch the contigs together into a closed chromosome because 49 out of the 101 CLEOA contigs contained the insertion sequence IS1111 at one or both ends. Similar to the CLEOA genome, multiple copies of IS1111 is present in the genomes of other CLEs and *C. burnetii* and is known to have an impact on genome evolution and gene content (Beare et al. 2009; Duron 2015). Although we couldn’t close the genome, the presence of 106 out of 111 highly conserved single-copy genes in both CLEOA and *C. burnetii* indicate that most of the CLEOA genome is represented in the assembled contigs. The final sets of 101 contigs were submitted to NCBI (accession VFIV00000000) and annotated using the Prokaryotic Genome Pipeline.

### Phylogenetic Analysis

Orthofinder (Emms and Kelly 2015) was utilized to identify 205 single-copy genes present in 52 representative species from the order Legionellales (supplementary tables S1 and S13, Supplementary Material online) in order to build the comprehensive phylogenomic tree supplementary figure S2, Supplementary Material online. A subset of 117 genes conserved in 30 species (supplementary tables S1 and S13, Supplementary Material online) were used to generate figure 1. For both trees, nucleotide sequences were aligned individually using global MAFFT (Katoh and Standley 2013) and were then concatenated. GBlocks (Talavera and Castresana 2007) was used to cull ambiguously aligned regions and jModelTest2 (Darriba et al. 2012) was used to select the appropriate model (GTR+I+G). Maximum likelihood trees were generated using RaxML and Bayesian trees were produced using MrBayes (Ronquist et al. 2012; Stamatakis 2014). The 16S rDNA trees were built using the same process as above, with the final tree based on 1203 nucleotide positions, and nodes with less than 70% support collapsed. To confirm the HGT origin of Node 5 genes, homologs were identified via BLASTp (NCBI nr database, e-value ≤10e−5, identity ≥30% identity, coverage ≥70%). The nucleotide sequences of the homologs were collected into a database, and the Phylomizer pipeline (https://github.com/Gabaldonlab/phylomizer) was used to generate individual maximum likelihood trees using the 75 most closely related homolog sequences. Each tree was then compared to an NCBI Taxonomy-based tree to validate HGT (supplementary fig. S1, Supplementary Material online).

### Determination of Nodes of Gene Origin

The presence of functional homologs of *C. burnetii* RSA493 (AE016828.3) genes in other members of the order Legionellales was determined using BLASTp (identity ≥30%, coverage ≥70%, e-value ≤10e−5), and pseudogenized homologs were detected using tBLASTn (identity ≥30%, coverage ≥50%, e-value ≤10e−5). The presence/absence profile was utilized in the Gain and Loss Mapping Engine (GLOOME) (Cohen et al. 2010) to determine the posterior probability of each gene’s presence at nodes N1–N5. For each *C. burnetii* gene, the node of origin was marked as the oldest node at which posterior probability was ≥0.7, with all subsequent nodes also having posterior probability of ≥0.7, as described previously (Peer and Margalit 2014). We also identified 409 genes that are conserved in all CLEs (supplementary table S14, Supplementary Material online) using BLASTp (identity ≥30%, coverage ≥70%, e-value ≤10e−5).

### Calculation of CAI and pI

We identified 22 highly conserved single-copy protein-coding genes in *C. burnetii* that were highly expressed in both ACCM-2 and within human macrophages based on previous RNA-seq data (supplementary table S5, Supplementary Material online; Warrier et al. 2014; Wachter et al. 2019). CodonW (http://codonw.sourceforge.net) was used to generate CAI values for the 22 genes in order to generate a model for optimal codon usage in *C. burnetii*, which was then compared to CAI values of sets of genes acquired at each node. All 22 genes used to build the model belonged to Node 1, and were not included in this analysis. Potentially spurious genes (*n* = 224) that did not have any detectable homologs outside of *C. burnetii*, as well as genes with undetermined nodes of origin (*n* = 44) were excluded from this analysis (supplementary table S15, Supplementary Material online). Isoelectric points (pI) for all proteins in CLEOA and *C. burnetii* RSA493 (AE016828.3) were calculated using IPC (Kozlowski 2016).

### GaPPIX Susceptibility Assay

A 10 mM GaPPIX (Frontier Scientific) solution was prepared in dimethyl sulfoxide (DMSO) and was stored at 4°C under dark conditions until further use. *C. burnetii* was cultured in ACCM-2 for 2 days at 37°C, 5% CO_2_ and 2.5% O_2_, and ∼2 × 10^7^ genome equivalents were resuspended in fresh ACCM-2 containing 125 nM, 250 nM, 500 nM, 1 mM, 2 mM, 4 mM, or 8 mM GaPPIX in 96-well black-bottom microplates (Greiner Bio-One). Bacterial growth was measured using PicoGreen (Invitrogen) as described previously (Moses et al. 2017).

THP-1 human monocytes (ATCC, TIB-202) were cultured in sterile RPMI-1640 medium (Gibco) supplemented with 1 mM sodium pyruvate, 0.05 mM beta-mercaptoethanol, 1% Pen-Strep, and 4500 mg/L glucose with 10% heat-inactivated fetal bovine serum at 37°C, 5% CO_2_ in 6-well tissue culture plates. Prior to infection, cells were differentiated into macrophages by treating with 30 nM phorbol 12-myristate 13-acetate (PMA) for 24 h, followed by resting in PMA-free RPMI for 24 h. Infection of THP-1 cells with *C. burnetii* was carried out using a 7d bacterial culture at a multiplicity of infection of 25. After briefly washing the cells with PBS, a bacteria-containing medium was added to each well and gently centrifuged for 10 min followed by incubation at 37°C, 5% CO_2_ for 2 h. To remove extracellular bacteria, cells were washed three times with PBS, and replaced with antibiotic-free RPMI and were incubated for 48 h before treating with GaPPIX- (2 μM, 8 μM, and 32 μM) or DMSO- (as control) containing media. After 72 h, cells were washed three times with PBS and intracellular bacterial load was measure using qPCR, as we described previously (Moses et al. 2017). Potential cytotoxicity of GaPPIX was determined by measuring the levels of released lactate dehydrogenase (LDH) in cell supernatants using an LDH Cytotoxicity Assay Kit (Invitrogen).

## Data Availability

The CLEOA genome generated in this study has been submitted to the NCBI Assembly database (https://www.ncbi.nlm.nih.gov/assembly/) under Whole Genome Shotgun (WGS) accession prefix VFIV, BioSample SAMN12040594, and BioProject PRJNA548565.

## Supplementary Material

Supplementary data are available at *Genome Biology and Evolution* online.

## Supplementary Material

evab108_Supplementary_Data
