# Supplementary material for: Coxiella burnetii and Related Tick Endosymbionts Evolved from Pathogenic Ancestors
Source: Genome Biol Evol. 2021 May 19;13(7):evab108. doi: 10.1093/gbe/evab108 (PMC8290121; doi:10.1093/gbe/evab108)
Supplement: evab108_Supplementary_Data [file evab108_supplementary_data.zip › CLEOA-supl-figure-legends.docx]

**SUPPLEMENTAL FIGURE LEGENDS**

**Supplemental** **Figure S1. HGT contributed to *C. burnetii* evolution.** 88 Node 5 genes have phylogenies (left) inconsistent with established taxonomic relationships (right), indicating that they were acquired via HGT. Branches and species titles are colored by taxonomy as specified on Page 1. (**A**) Maximum likelihood gene trees with bootstrap values labeled at branchpoints. *C. burnetii* RSA493 genes are indicated by their locus tags and are highlighted in blue. Duplicate genes are shown together in single trees. (**B**) Taxonomy-based trees depict the established relationships among the taxa featured in A. Taxa sharing the same NCBI taxonomic identifier number are displayed only once even if multiple representatives are present in A.

**Supplemental** **Figure S2**. **Legionellales phylogenomic tree**. Maximum likelihood and Bayesian trees were generated using 205 single-copy protein-coding genes conserved in all represented taxa (Supplemental Tables S1, S13). Bootstrap support and posterior probabilities are labeled above and below each node respectively. This tree was used in GLOOME (Cohen et al. 2010) to determine the nodes of gene origin.
