# Supplementary material for: Coxiella burnetii and Related Tick Endosymbionts Evolved from Pathogenic Ancestors
Source: Genome Biol Evol. 2021 May 19;13(7):evab108. doi: 10.1093/gbe/evab108 (PMC8290121; doi:10.1093/gbe/evab108)

Acidobacteria

Aquificae

Deferribacteres

Elusimicrobia

FCB Group

Fusobacteria

Nitrospinae Tectomicrobia Group

Nitrospirae

PVC Group

Spirochaetes

Synergistetes

Terrabacteria Group

Thermodesulfobacteria

Thermotogae

Proteobacteria: Acidithiobacillia

Proteobacteria: Alphaproteobacteria

Proteobacteria: Betaproteobacteria

Proteobacteria: Candidatus Muproteobacteria

Proteobacteria: Delta Epsilon Subdivisions

Proteobacteria: Gammaproteobacteria

Proteobacteria: Hydrogenophilalia

Proteobacteria: Oligoflexia

Proteobacteria: Zetaproteobacteria

Proteobacteria: Unclassified & Environmental Samples

Bacteria: Unclassified & Environmental Samples

Viruses & Constructs

Eukaryota

Archaea

CBU_2085

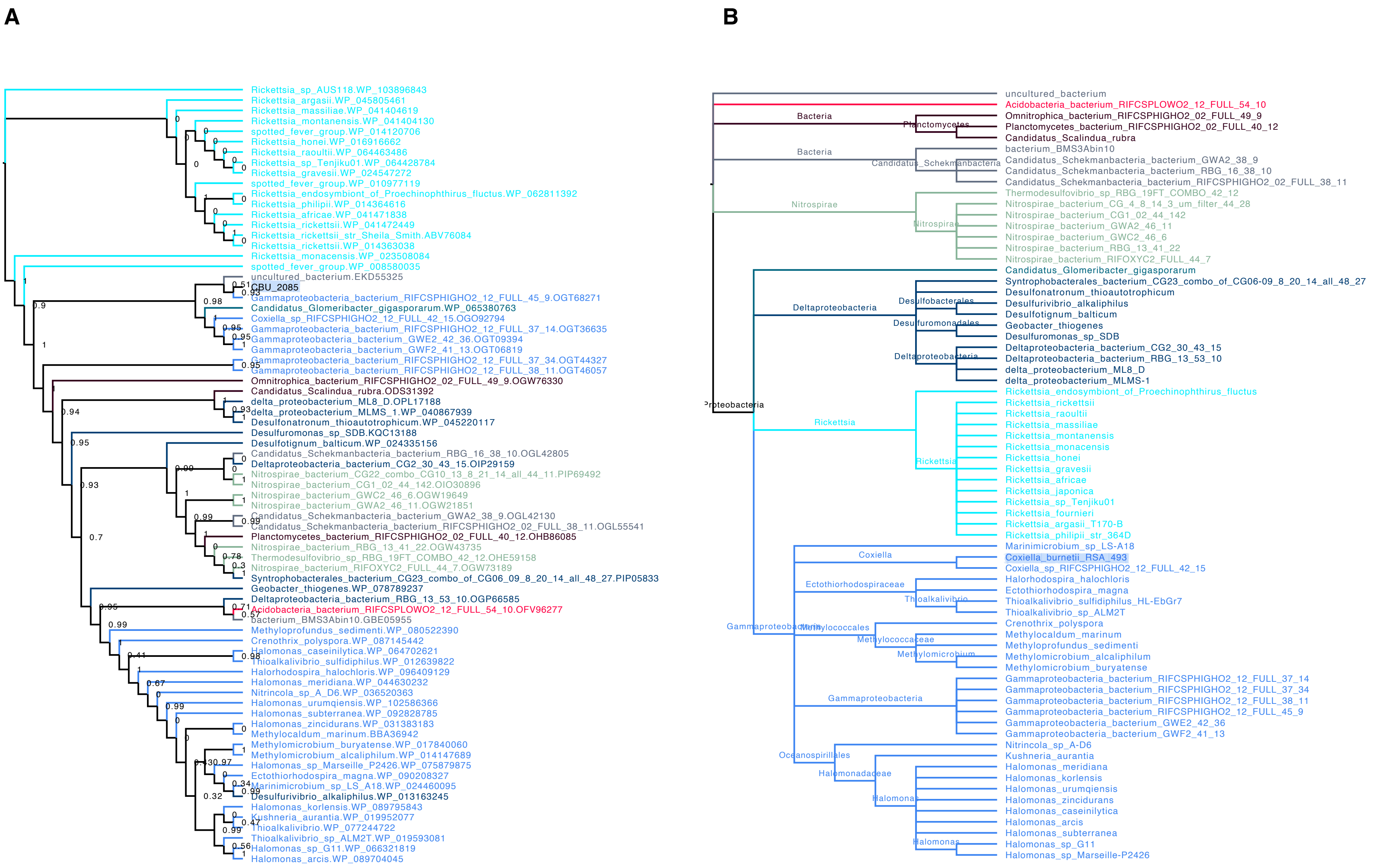

A
Rickettsia_sp_AUS118.WP_103896843
Rickettsia_argasii.WP_045805461
Rickettsia_massiliae.WP_041404619
Rickettsia_montanensis.WP_041404130
spotted_fever_group.WP_014120706
Rickettsia_honei.WP_016916662
Rickettsia_raoultii.WP_064463486
Rickettsia_sp_Tenjiku01.WP_064428784
Rickettsia_gravesii.WP_024547272
spotted_fever_group.WP_010977119
Rickettsia_endosymbiont_of_Proechinophthirus_fluctus.WP_062811392
Rickettsia_philipii.WP_014364616
Rickettsia_africae.WP_041471838
Rickettsia_rickettsii.WP_041472449
Rickettsia_rickettsii_str_Sheila_Smith.ABV76084
Rickettsia_rickettsii.WP_014363038
Rickettsia_monacensis.WP_023508084
spotted_fever_group.WP_008580035
uncultured_bacterium.EKD55325
CBU_2085
Gammaproteobacteria_bacterium_RIFCSPHIGHO2_12_FULL_45_9.OGT68271
Candidatus_Glomeribacter_gigasporarum.WP_065380763
Coxiella_sp_RIFCSPHIGHO2_12_FULL_42_15.OGO92794
Gammaproteobacteria_bacterium_RIFCSPHIGHO2_12_FULL_37_14.OGT36635
Gammaproteobacteria_bacterium_GWE2_42_36.OGT09394
Gammaproteobacteria_bacterium_GWF2_41_13.OGT06819
Gammaproteobacteria_bacterium_RIFCSPHIGHO2_12_FULL_37_34.OGT44327
Gammaproteobacteria_bacterium_RIFCSPHIGHO2_12_FULL_38_11.OGT46057
Omnitrophica_bacterium_RIFCSPHIGHO2_02_FULL_49_9.OGW76330
Candidatus_Scalindua_rubra.ODS31392
delta_proteobacterium_ML8_D.OPL17188
delta_proteobacterium_MLMS_1.WP_040867939
Desulfonatronum_thioautotrophicum.WP_045220117
Desulfuromonas_sp_SDB.KQC13188
Desulfotignum_balticum.WP_024335156
Candidatus_Schekmanbacteria_bacterium_RBG_16_38_10.OGL42805
Deltaproteobacteria_bacterium_CG2_30_43_15.OIP29159
Nitrospirae_bacterium_CG22_combo_CG10_13_8_21_14_all_44_11.PIP69492
Nitrospirae_bacterium_CG1_02_44_142.OIO30896
Nitrospirae_bacterium_GWC2_46_6.OGW19649
Nitrospirae_bacterium_GWA2_46_11.OGW21851
Candidatus_Schekmanbacteria_bacterium_GWA2_38_9.OGL42130
Candidatus_Schekmanbacteria_bacterium_RIFCSPHIGHO2_02_FULL_38_11.OGL55541
Planctomycetes_bacterium_RIFCSPHIGHO2_02_FULL_40_12.OHB86085
Nitrospirae_bacterium_RBG_13_41_22.OGW43735
Thermodesulfovibrio_sp_RBG_19FT_COMBO_42_12.OHE59158
Nitrospirae_bacterium_RIFOXYC2_FULL_44_7.OGW73189
Syntrophobacterales_bacterium_CG23_combo_of_CG06_09_8_20_14_all_48_27.PIP05833
Geobacter_thiogenes.WP_078789237
Deltaproteobacteria_bacterium_RBG_13_53_10.OGP66585
Acidobacteria_bacterium_RIFCSPLOWO2_12_FULL_54_10.OFV96277
bacterium_BMS3Abin10.GBE05955
Methyloprofundus_sedimenti.WP_080522390
Crenothrix_polyspora.WP_087145442
Halomonas_caseinilytica.WP_064702621
Thioalkalivibrio_sulfidiphilus.WP_012639822
Halorhodospira_halochloris.WP_096409129
Halomonas_meridiana.WP_044630232
Nitrincola_sp_A_D6.WP_036520363
Halomonas_urumqiensis.WP_102586366
Halomonas_subterranea.WP_092828785
Halomonas_zincidurans.WP_031383183
Methylocaldum_marinum.BBA36942
Methylomicrobium_buryatense.WP_017840060
Methylomicrobium_alcaliphilum.WP_014147689
Halomonas_sp_Marseille_P2426.WP_075879875
Ectothiorhodospira_magna.WP_090208327
Marinimicrobium_sp_LS_A18.WP_024460095
Desulfurivibrio_alkaliphilus.WP_013163245
Halomonas_korlensis.WP_089795843
Kushneria_aurantia.WP_019952077
Thioalkalivibrio.WP_077244722
Thioalkalivibrio_sp_ALM2T.WP_019593081
Halomonas_sp_G11.WP_066321819
Halomonas_arcis.WP_089704045
B
Bacteria
Planctomycetes
Bacteria
Candidatus_Schekmanbacteria
Nitrospirae
Nitrospirae
Deltaproteobacteria
Desulfobacterales
Desulfuromonadales
Deltaproteobacteria
Proteobacteria
Rickettsia
Rickettsia
Coxiella
Ectothiorhodospiraceae
Thioalkalivibrio
Gammaproteobacteria
Methylococcales
Methylococcaceae
Methylomicrobium
Gammaproteobacteria
Oceanospirillales
Halomonadaceae
Halomonas
Halomonas
uncultured_bacterium
Acidobacteria_bacterium_RIFCSPLOWO2_12_FULL_54_10
Omnitrophica_bacterium_RIFCSPHIGHO2_02_FULL_49_9
Planctomycetes_bacterium_RIFCSPHIGHO2_02_FULL_40_12
Candidatus_Scalindua_rubra
bacterium_BMS3Abin10
Candidatus_Schekmanbacteria_bacterium_GWA2_38_9
Candidatus_Schekmanbacteria_bacterium_RBG_16_38_10
Candidatus_Schekmanbacteria_bacterium_RIFCSPHIGHO2_02_FULL_38_11
Thermodesulfovibrio_sp_RBG_19FT_COMBO_42_12
Nitrospirae_bacterium_CG_4_8_14_3_um_filter_44_28
Nitrospirae_bacterium_CG1_02_44_142
Nitrospirae_bacterium_GWA2_46_11
Nitrospirae_bacterium_GWC2_46_6
Nitrospirae_bacterium_RBG_13_41_22
Nitrospirae_bacterium_RIFOXYC2_FULL_44_7
Candidatus_Glomeribacter_gigasporarum
Syntrophobacterales_bacterium_CG23_combo_of_CG06-09_8_20_14_all_48_27
Desulfonatronum_thioautotrophicum
Desulfurivibrio_alkaliphilus
Desulfotignum_balticum
Geobacter_thiogenes
Desulfuromonas_sp_SDB
Deltaproteobacteria_bacterium_CG2_30_43_15
Deltaproteobacteria_bacterium_RBG_13_53_10
delta_proteobacterium_ML8_D
delta_proteobacterium_MLMS-1
Rickettsia_endosymbiont_of_Proechinophthirus_fluctus
Rickettsia_rickettsii
Rickettsia_raoultii
Rickettsia_massiliae
Rickettsia_montanensis
Rickettsia_monacensis
Rickettsia_honei
Rickettsia_gravesii
Rickettsia_africae
Rickettsia_japonica
Rickettsia_sp_Tenjiku01
Rickettsia_fournieri
Rickettsia_argasii_T170-B
Rickettsia_philipii_str_364D
Marinimicrobium_sp_LS-A18
Coxiella_burnetii_RSA_493
Coxiella_sp_RIFCSPHIGHO2_12_FULL_42_15
Halorhodospira_halochloris
Ectothiorhodospira_magna
Thioalkalivibrio_sulfidiphilus_HL-EbGr7
Thioalkalivibrio_sp_ALM2T
Crenothrix_polyspora
Methylocaldum_marinum
Methyloprofundus_sedimenti
Methylomicrobium_alcaliphilum
Methylomicrobium_buryatense
Gammaproteobacteria_bacterium_RIFCSPHIGHO2_12_FULL_37_14
Gammaproteobacteria_bacterium_RIFCSPHIGHO2_12_FULL_37_34
Gammaproteobacteria_bacterium_RIFCSPHIGHO2_12_FULL_38_11
Gammaproteobacteria_bacterium_RIFCSPHIGHO2_12_FULL_45_9
Gammaproteobacteria_bacterium_GWE2_42_36
Gammaproteobacteria_bacterium_GWF2_41_13
Nitrincola_sp_A-D6
Kushneria_aurantia
Halomonas_meridiana
Halomonas_korlensis
Halomonas_urumqiensis
Halomonas_zincidurans
Halomonas_caseinilytica
Halomonas_arcis
Halomonas_subterranea
Halomonas_sp_G11
Halomonas_sp_Marseille-P2426

CBU_2084

A **B**

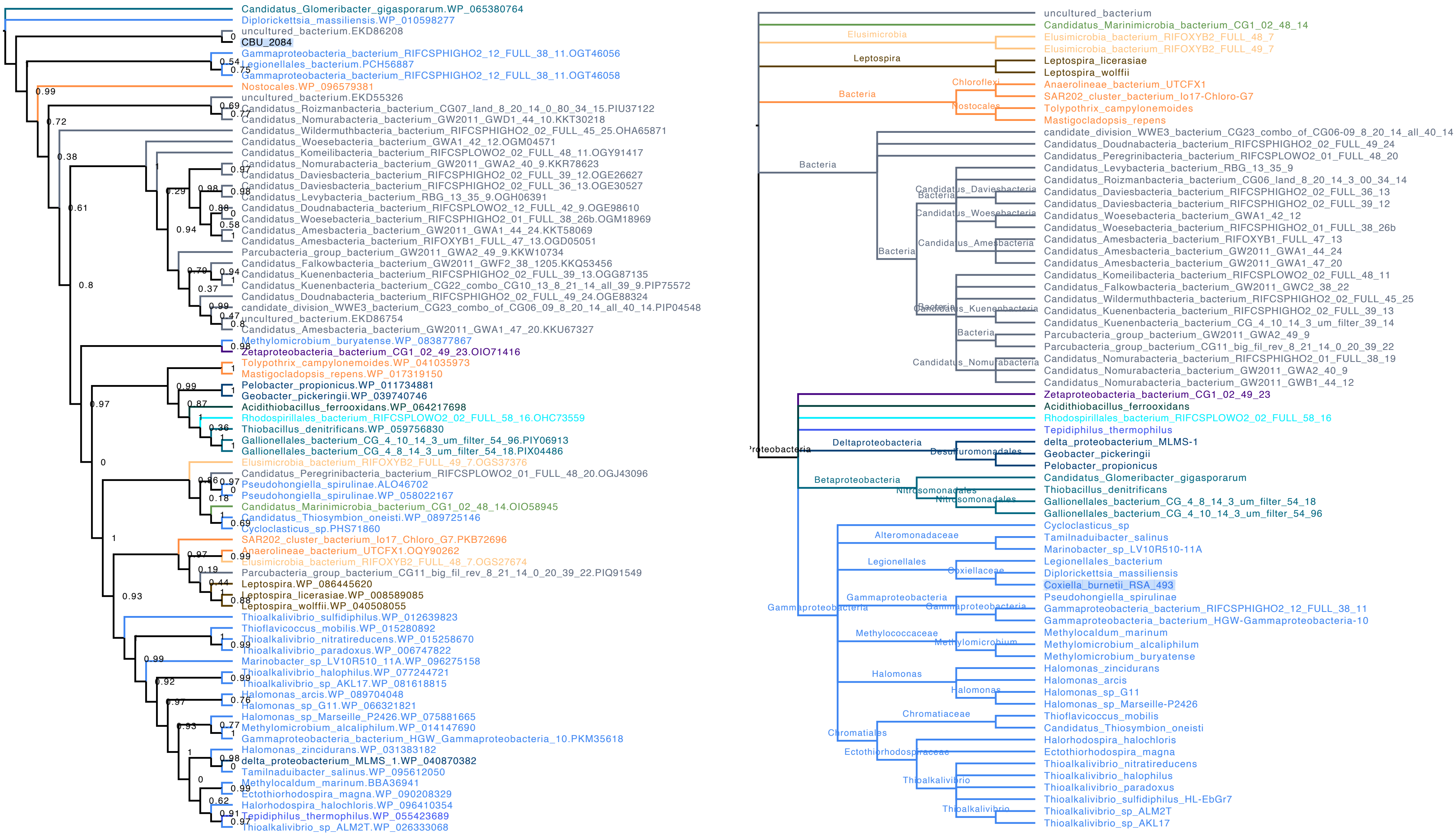

Candidatus_Glomeribacter_gigasporarum.WP_065380764
Diplorickettsia_massiliensis.WP_010598277
uncultured_bacterium.EKD86208
CBU_2084
Gammaproteobacteria_bacterium_RIFCSPHIGHO2_12_FULL_38_11.OGT46056
Legionellales_bacterium.PCH56887
Gammaproteobacteria_bacterium_RIFCSPHIGHO2_12_FULL_38_11.OGT46058
Nostocales.WP_096579381
uncultured_bacterium.EKD55326
Candidatus_Roizmanbacteria_bacterium_CG07_land_8_20_14_0_80_34_15.PIU37122
Candidatus_Nomurabacteria_bacterium_GW2011_GWD1_44_10.KKT30218
Candidatus_Wildermuthbacteria_bacterium_RIFCSPHIGHO2_02_FULL_45_25.OHA65871
Candidatus_Woesebacteria_bacterium_GWA1_42_12.OGM04571
Candidatus_Komeilibacteria_bacterium_RIFCSPLOWO2_02_FULL_48_11.OGY91417
Candidatus_Nomurabacteria_bacterium_GW2011_GWA2_40_9.KKR78623
Candidatus_Daviesbacteria_bacterium_RIFCSPHIGHO2_02_FULL_39_12.OGE26627
Candidatus_Daviesbacteria_bacterium_RIFCSPHIGHO2_02_FULL_36_13.OGE30527
Candidatus_Levybacteria_bacterium_RBG_13_35_9.OGH06391
Candidatus_Doudnabacteria_bacterium_RIFCSPLOWO2_12_FULL_42_9.OGE98610
Candidatus_Woesebacteria_bacterium_RIFCSPHIGHO2_01_FULL_38_26b.OGM18969
Candidatus_Amesbacteria_bacterium_GW2011_GWA1_44_24.KKT58069
Candidatus_Amesbacteria_bacterium_RIFOXYB1_FULL_47_13.OGD05051
Parcubacteria_group_bacterium_GW2011_GWA2_49_9.KKW10734
Candidatus_Falkowbacteria_bacterium_GW2011_GWF2_38_1205.KKQ53456
Candidatus_Kuenenbacteria_bacterium_RIFCSPHIGHO2_02_FULL_39_13.OGG87135
Candidatus_Kuenenbacteria_bacterium_CG22_combo_CG10_13_8_21_14_all_39_9.PIP75572
Candidatus_Doudnabacteria_bacterium_RIFCSPHIGHO2_02_FULL_49_24.OGE88324
candidate_division_WWE3_bacterium_CG23_combo_of_CG06_09_8_20_14_all_40_14.PIP04548
uncultured_bacterium.EKD86754
Candidatus_Amesbacteria_bacterium_GW2011_GWA1_47_20.KKU67327
Methylomicrobium_buryatense.WP_083877867
Zetaproteobacteria_bacterium_CG1_02_49_23.OIO71416
Tolypothrix_campylonemoides.WP_041035973
Mastigocladopsis_repens.WP_017319150
Pelobacter_propionicus.WP_011734881
Geobacter_pickeringii.WP_039740746
Acidithiobacillus_ferrooxidans.WP_064217698
Rhodospirillales_bacterium_RIFCSPLOWO2_02_FULL_58_16.OHC73559
Thiobacillus_denitrificans.WP_059756830
Gallionellales_bacterium_CG_4_10_14_3_um_filter_54_96.PIY06913
Gallionellales_bacterium_CG_4_8_14_3_um_filter_54_18.PIX04486
Elusimicrobia_bacterium_RIFOXYB2_FULL_49_7.OGS37376
Candidatus_Peregrinibacteria_bacterium_RIFCSPLOWO2_01_FULL_48_20.OGJ43096
Pseudohongiella_spirulinae.ALO46702
Pseudohongiella_spirulinae.WP_058022167
Candidatus_Marinimicrobia_bacterium_CG1_02_48_14.OIO58945
Candidatus_Thiosymbion_oneisti.WP_089725146
Cycloclasticus_sp.PHS71860
SAR202_cluster_bacterium_Io17_Chloro_G7.PKB72696
Anaerolineae_bacterium_UTCFX1.OQY90262
Elusimicrobia_bacterium_RIFOXYB2_FULL_48_7.OGS27674
Parcubacteria_group_bacterium_CG11_big_fil_rev_8_21_14_0_20_39_22.PIQ91549
Leptospira.WP_086445620
Leptospira_licerasiae.WP_008589085
Leptospira_wolffii.WP_040508055
Thioalkalivibrio_sulfidiphilus.WP_012639823
Thioflavicoccus_mobilis.WP_015280892
Thioalkalivibrio_nitratireducens.WP_015258670
Thioalkalivibrio_paradoxus.WP_006747822
Marinobacter_sp_LV10R510_11A.WP_096275158
Thioalkalivibrio_halophilus.WP_077244721
Thioalkalivibrio_sp_AKL17.WP_081618815
Halomonas_arcis.WP_089704048
Halomonas_sp_G11.WP_066321821
Halomonas_sp_Marseille_P2426.WP_075881665
Methylomicrobium_alcaliphilum.WP_014147690
Gammaproteobacteria_bacterium_HGW_Gammaproteobacteria_10.PKM35618
Halomonas_zincidurans.WP_031383182
delta_proteobacterium_MLMS_1.WP_040870382
Tamilnaduibacter_salinus.WP_095612050
Methylocaldum_marinum.BBA36941
Ectothiorhodospira_magna.WP_090208329
Halorhodospira_halochloris.WP_096410354
Tepidiphilus_thermophilus.WP_055423689
Thioalkalivibrio_sp_ALM2T.WP_026333068
uncultured_bacterium
Candidatus_Marinimicrobia_bacterium_CG1_02_48_14
Elusimicrobia
Elusimicrobia_bacterium_RIFOXYB2_FULL_48_7
Elusimicrobia_bacterium_RIFOXYB2_FULL_49_7
Leptospira
Leptospira_licerasiae
Leptospira_wolffii
Chloroflexi
Bacteria
Nostocales
Anaerolineae_bacterium_UTCFX1
SAR202_cluster_bacterium_Io17-Chloro-G7
Tolypothrix_campylonemoides
Mastigocladopsis_repens
candidate_division_WWE3_bacterium_CG23_combo_of_CG06-09_8_20_14_all_40_14
Candidatus_Doudnabacteria_bacterium_RIFCSPHIGHO2_02_FULL_49_24
Candidatus_Peregrinibacteria_bacterium_RIFCSPLOWO2_01_FULL_48_20
Candidatus_Levybacteria_bacterium_RBG_13_35_9
Candidatus_Roizmanbacteria_bacterium_CG06_land_8_20_14_3_00_34_14
Candidatus_Daviesbacteria
Candidatus_Daviesbacteria_bacterium_RIFCSPHIGHO2_02_FULL_36_13
Candidatus_Daviesbacteria_bacterium_RIFCSPHIGHO2_02_FULL_39_12
Candidatus_Woesebacteria
Candidatus_Woesebacteria_bacterium_GWA1_42_12
Candidatus_Woesebacteria_bacterium_RIFCSPHIGHO2_01_FULL_38_26b
Candidatus_Amesbacteria
Candidatus_Amesbacteria_bacterium_RIFOXYB1_FULL_47_13
Candidatus_Amesbacteria_bacterium_GW2011_GWA1_44_24
Candidatus_Amesbacteria_bacterium_GW2011_GWA1_47_20
Candidatus_Komeilibacteria_bacterium_RIFCSPLOWO2_02_FULL_48_11
Candidatus_Falkowbacteria_bacterium_GW2011_GWC2_38_22
Candidatus_Wildermuthbacteria_bacterium_RIFCSPHIGHO2_02_FULL_45_25
Candidatus_Kuenenbacteria
Candidatus_Kuenenbacteria_bacterium_RIFCSPHIGHO2_02_FULL_39_13
Candidatus_Kuenenbacteria_bacterium_CG_4_10_14_3_um_filter_39_14
Parcubacteria_group_bacterium_GW2011_GWA2_49_9
Parcubacteria_group_bacterium_CG11_big_fil_rev_8_21_14_0_20_39_22
Candidatus_Nomurabacteria
Candidatus_Nomurabacteria_bacterium_RIFCSPHIGHO2_01_FULL_39_19
Candidatus_Nomurabacteria_bacterium_GW2011_GWA2_40_9
Candidatus_Nomurabacteria_bacterium_GW2011_GWB1_44_12
Zetaproteobacteria_bacterium_CG1_02_49_23
Acidithiobacillus_ferrooxidans
Rhodospirillales_bacterium_RIFCSPLOWO2_02_FULL_58_16
Tepidiphilus_thermophilus
Proteobacteria
Deltaproteobacteria
Desulfuromonadales
delta_proteobacterium_MLMS-1
Geobacter_pickeringii
Pelobacter_propionicus
Betaproteobacteria
Nitrosomonadales
Candidatus_Glomeribacter_gigasporarum
Thiobacillus_denitrificans
Gallionellales_bacterium_CG_4_8_14_3_um_filter_54_18
Gallionellales_bacterium_CG_4_10_14_3_um_filter_54_96
Alteromonadaceae
Cycloclasticus_sp
Tamilnaduibacter_salinus
Marinobacter_sp_LV10R510-11A
Legionellales
Coxiellaceae
Legionellales_bacterium
Diplorickettsia_massiliensis
Coxiella_burnetii_RSA_493
Gammaproteobacteria
Pseudohongiella_spirulinae
Gammaproteobacteria_bacterium_RIFCSPHIGHO2_12_FULL_38_11
Gammaproteobacteria_bacterium_HGW-Gammaproteobacteria-10
Methylococcaceae
Methylomicrobium
Methylocaldum_marinum
Methylomicrobium_alcaliphilum
Methylomicrobium_buryatense
Halomonas
Halomonas_zincidurans
Halomonas_arcis
Halomonas_sp_G11
Halomonas_sp_Marseille-P2426
Chromatiales
Chromatiaceae
Thioflavicoccus_mobilis
Candidatus_Thiosymbion_oneisti
Ectothiorhodospiraceae
Halorhodospira_halochloris
Ectothiorhodospira_magna
Thioalkalivibrio
Thioalkalivibrio_nitratireducens
Thioalkalivibrio_halophilus
Thioalkalivibrio_paradoxus
Thioalkalivibrio_sulfidiphilus_HL-EbGr7
Thioalkalivibrio_sp_ALM2T
Thioalkalivibrio_sp_AKL17

CBU_2078

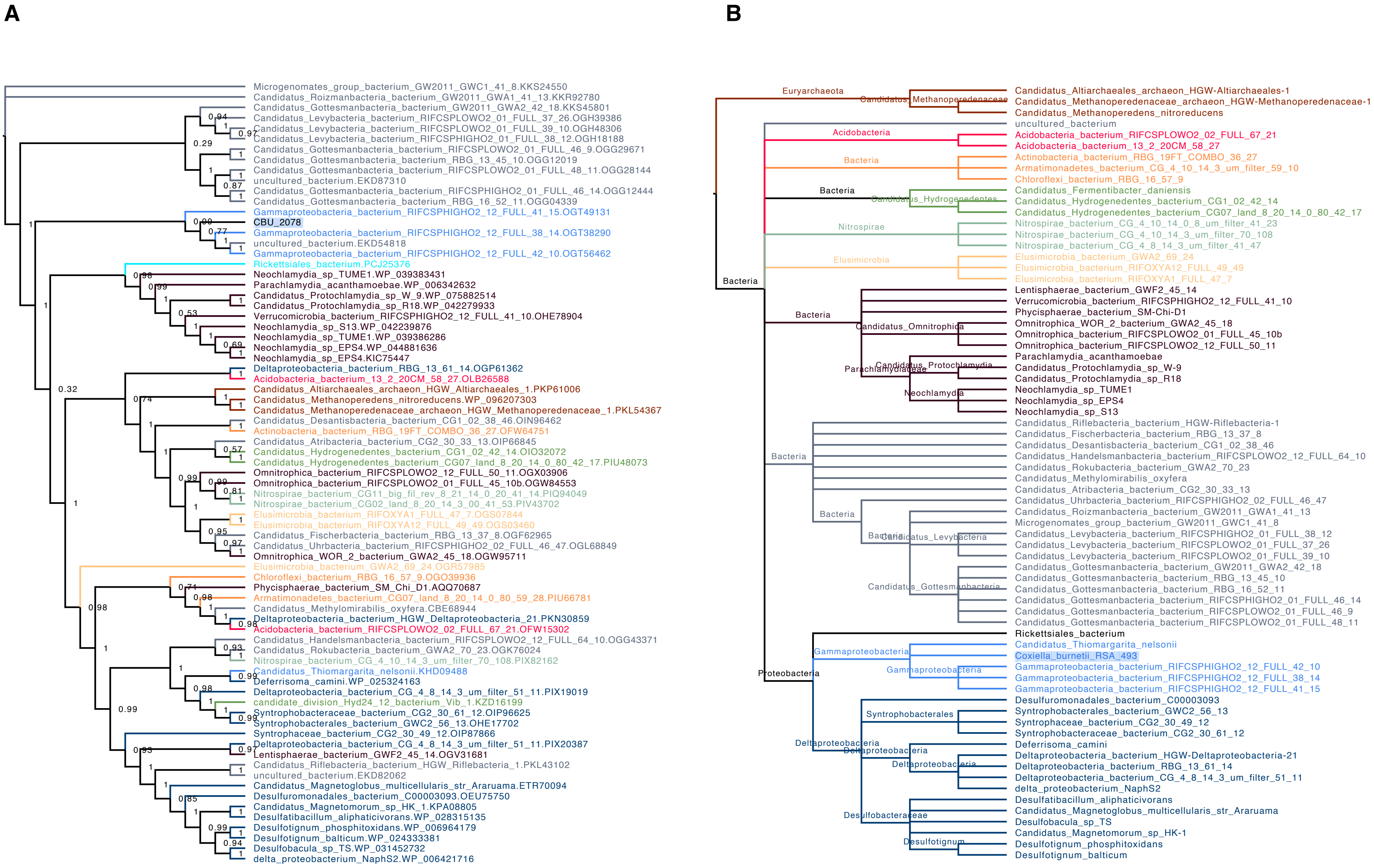
A
Microgenomates_group_bacterium_GW2011_GWC1_41_8.KKS24550
Candidatus_Roizmanbacteria_bacterium_GW2011_GWA1_41_13.KKR92780
Candidatus_Gottesmanbacteria_bacterium_GW2011_GWA2_42_18.KKS45801
Candidatus_Levybacteria_bacterium_RIFCSPLOWO2_01_FULL_37_26.OGH39386
Candidatus_Levybacteria_bacterium_RIFCSPLOWO2_01_FULL_39_10.OGH48306
Candidatus_Levybacteria_bacterium_RIFCSPHIGHO2_01_FULL_38_12.OGH18188
Candidatus_Gottesmanbacteria_bacterium_RIFCSPLOWO2_01_FULL_46_9.OGG29671
Candidatus_Gottesmanbacteria_bacterium_RBG_13_45_10.OGG12019
Candidatus_Gottesmanbacteria_bacterium_RIFCSPLOWO2_01_FULL_48_11.OGG28144
uncultured_bacterium.EKD87310
Candidatus_Gottesmanbacteria_bacterium_RIFCSPHIGHO2_01_FULL_46_14.OGG12444
Candidatus_Gottesmanbacteria_bacterium_RBG_16_52_11.OGG04339
Gammaproteobacteria_bacterium_RIFCSPHIGHO2_12_FULL_41_15.OGT49131
CBU_2078
Gammaproteobacteria_bacterium_RIFCSPHIGHO2_12_FULL_38_14.OGT38290
uncultured_bacterium.EKD54818
Gammaproteobacteria_bacterium_RIFCSPHIGHO2_12_FULL_42_10.OGT56462
Rickettsiales_bacterium.PCJ25376
Neochlamydia_sp_TUME1.WP_039383431
Parachlamydia_acanthamoebae.WP_006342632
Candidatus_Protochlamydia_sp_W_9.WP_075882514
Candidatus_Protochlamydia_sp_R18.WP_042279933
Verrucomicrobia_bacterium_RIFCSPHIGHO2_12_FULL_41_10.OHE78904
Neochlamydia_sp_S13.WP_042239876
Neochlamydia_sp_TUME1.WP_039386286
Neochlamydia_sp_EPS4.WP_044881636
Neochlamydia_sp_EPS4.KIC75447
Deltaproteobacteria_bacterium_RBG_13_61_14.OGP61362
Acidobacteria_bacterium_13_2_20CM_58_27.OLB26588
Candidatus_Altiarchaeales_archaeon_HGW_Altiarchaeales_1.PKP61006
Candidatus_Methanoperedens_nitroreducens.WP_096207303
Candidatus_Methanoperedenaceae_archaeon_HGW_Methanoperedenaceae_1.PKL54367
Candidatus_Desantisbacteria_bacterium_CG1_02_38_46.OIN96462
Actinobacteria_bacterium_RBG_19FT_COMBO_36_27.OFW64751
Candidatus_Atribacteria_bacterium_CG2_30_33_13.OIP66845
Candidatus_Hydrogenedentes_bacterium_CG1_02_42_14.OIO32072
Candidatus_Hydrogenedentes_bacterium_CG07_land_8_20_14_0_80_42_17.PIU48073
Omnitrophica_bacterium_RIFCSPLOWO2_12_FULL_50_11.OGX03906
Omnitrophica_bacterium_RIFCSPLOWO2_01_FULL_45_10b.OGW84553
Nitrospirae_bacterium_CG11_big_fil_rev_8_21_14_0_20_41_14.PIQ94049
Nitrospirae_bacterium_CG02_land_8_20_14_3_00_41_53.PIV43702
Elusimicrobia_bacterium_RIFOXYA1_FULL_47_7.OGS07844
Elusimicrobia_bacterium_RIFOXYA12_FULL_49_49.OGS03460
Candidatus_Fischerbacteria_bacterium_RBG_13_37_8.OGF62965
Candidatus_Uhrbacteria_bacterium_RIFCSPHIGHO2_02_FULL_46_47.OGL68849
Omnitrophica_WOR_2_bacterium_GWA2_45_18.OGW95711
Elusimicrobia_bacterium_GWA2_69_24.OGR57985
Chloroflexi_bacterium_RBG_16_57_9.OGO39936
Phycisphaerae_bacterium_SM_Chi_D1.AQQ70687
Armatimonadetes_bacterium_CG07_land_8_20_14_0_80_59_28.PIU66781
Candidatus_Methylomirabilis_oxyfera.CBE68944
Deltaproteobacteria_bacterium_HGW_Deltaproteobacteria_21.PKN30859
Acidobacteria_bacterium_RIFCSPLOWO2_02_FULL_67_21.OFW15302
Candidatus_Handelsmanbacteria_bacterium_RIFCSPLOWO2_12_FULL_64_10.OGG43371
Candidatus_Rokubacteria_bacterium_GWA2_70_23.OGK76024
Nitrospirae_bacterium_CG_4_10_14_3_um_filter_70_108.PIX82162
Candidatus_Thiomargarita_nelsonii.KHD09488
Deferrisoma_camini.WP_025324163
Deltaproteobacteria_bacterium_CG_4_8_14_3_um_filter_51_11.PIX19019
candidate_division_Hyd24_12_bacterium_Vib_1.KZD16199
Syntrophobacteraceae_bacterium_CG2_30_61_12.OIP96625
Syntrophobacterales_bacterium_GWC2_56_13.OHE17702
Syntrophaceae_bacterium_CG2_30_49_12.OIP87866
Deltaproteobacteria_bacterium_CG_4_8_14_3_um_filter_51_11.PIX20387
Lentisphaerae_bacterium_GWF2_45_14.OGV31681
Candidatus_Riflebacteria_bacterium_HGW_Riflebacteria_1.PKL43102
uncultured_bacterium.EKD82062
Candidatus_Magnetoglobus_multicellularis_str_Araruama.ETR70094
Desulfuromonadales_bacterium_C00003093.OEU75750
Candidatus_Magnetomorum_sp_HK_1.KPA08805
Desulfatibacillum_aliphaticivorans.WP_028315135
Desulfotignum_phosphitoxidans.WP_006964179
Desulfotignum_balticum.WP_024333381
Desulfobacula_sp_TS.WP_031452732
delta_proteobacterium_NaphS2.WP_006421716
B
Euryarchaeota
Candidatus_Methanoperedenaceae
Candidatus_Altiarchaeales_archaeon_HGW-Altiarchaeales-1
Candidatus_Methanoperedenaceae_archaeon_HGW-Methanoperedenaceae-1
Candidatus_Methanoperedens_nitroreducens
uncultured_bacterium
Acidobacteria
Acidobacteria_bacterium_RIFCSPLOWO2_02_FULL_67_21
Acidobacteria_bacterium_13_2_20CM_58_27
Bacteria
Actinobacteria_bacterium_RBG_19FT_COMBO_36_27
Armatimonadetes_bacterium_CG_4_10_14_3_um_filter_59_10
Chloroflexi_bacterium_RBG_16_57_9
Bacteria
Candidatus_Hydrogenedentes
Candidatus_Fermentibacter_daniensis
Candidatus_Hydrogenedentes_bacterium_CG1_02_42_14
Candidatus_Hydrogenedentes_bacterium_CG07_land_8_20_14_0_80_42_17
Nitrospirae
Nitrospirae_bacterium_CG_4_10_14_0_8_um_filter_41_23
Nitrospirae_bacterium_CG_4_10_14_3_um_filter_70_108
Nitrospirae_bacterium_CG_4_8_14_3_um_filter_41_47
Elusimicrobia
Elusimicrobia_bacterium_GWA2_69_24
Elusimicrobia_bacterium_RIFOXYA12_FULL_49_49
Elusimicrobia_bacterium_RIFOXYA1_FULL_47_7
Bacteria
Bacteria
Lentisphaerae_bacterium_GWF2_45_14
Verrucomicrobia_bacterium_RIFCSPHIGHO2_12_FULL_41_10
Phycisphaerae_bacterium_SM-Chi-D1
Candidatus_Omnitrophica
Omnitrophica_WOR_2_bacterium_GWA2_45_18
Omnitrophica_bacterium_RIFCSPLOWO2_01_FULL_45_10b
Omnitrophica_bacterium_RIFCSPLOWO2_12_FULL_50_11
Parachlamydiaceae
Candidatus_Protochlamydia
Parachlamydia_acanthamoebae
Candidatus_Protochlamydia_sp_W-9
Candidatus_Protochlamydia_sp_R18
Neochlamydia
Neochlamydia_sp_TUME1
Neochlamydia_sp_EPS4
Neochlamydia_sp_S13
Bacteria
Candidatus_Riflebacteria_bacterium_HGW-Riflebacteria-1
Candidatus_Fischerbacteria_bacterium_RBG_13_37_8
Candidatus_Desantisbacteria_bacterium_CG1_02_38_46
Candidatus_Handelsmanbacteria_bacterium_RIFCSPLOWO2_12_FULL_64_10
Candidatus_Rokubacteria_bacterium_GWA2_70_23
Candidatus_Methylomirabilis_oxyfera
Candidatus_Atribacteria_bacterium_CG2_30_33_13
Bacteria
Candidatus_Uhrbacteria_bacterium_RIFCSPHIGHO2_02_FULL_46_47
Candidatus_Roizmanbacteria_bacterium_GW2011_GWA1_41_13
Bacteria
Microgenomates_group_bacterium_GW2011_GWC1_41_8
Candidatus_Levybacteria
Candidatus_Levybacteria_bacterium_RIFCSPHIGHO2_01_FULL_38_12
Candidatus_Levybacteria_bacterium_RIFCSPLOWO2_01_FULL_37_26
Candidatus_Levybacteria_bacterium_RIFCSPLOWO2_01_FULL_39_10
Candidatus_Gottesmanbacteria
Candidatus_Gottesmanbacteria_bacterium_GW2011_GWA2_42_18
Candidatus_Gottesmanbacteria_bacterium_RBG_13_45_10
Candidatus_Gottesmanbacteria_bacterium_RBG_16_52_11
Candidatus_Gottesmanbacteria_bacterium_RIFCSPHIGHO2_01_FULL_46_14
Candidatus_Gottesmanbacteria_bacterium_RIFCSPLOWO2_01_FULL_46_9
Candidatus_Gottesmanbacteria_bacterium_RIFCSPLOWO2_01_FULL_48_11
Proteobacteria
Rickettsiales_bacterium
Gammaproteobacteria
Candidatus_Thiomargarita_nelsonii
Coxiella_burnetii_RSA_493
Gammaproteobacteria
Gammaproteobacteria_bacterium_RIFCSPHIGHO2_12_FULL_42_10
Gammaproteobacteria_bacterium_RIFCSPHIGHO2_12_FULL_38_14
Gammaproteobacteria_bacterium_RIFCSPHIGHO2_12_FULL_41_15
Deltaproteobacteria
Desulfuromonadales_bacterium_C00003093
Syntrophobacterales
Syntrophobacterales_bacterium_GWC2_56_13
Syntrophaceae_bacterium_CG2_30_49_12
Syntrophobacteraceae_bacterium_CG2_30_61_12
Deltaproteobacteria
Deferrisoma_camini
Deltaproteobacteria
Deltaproteobacteria_bacterium_HGW-Deltaproteobacteria-21
Deltaproteobacteria_bacterium_RBG_13_61_14
Deltaproteobacteria_bacterium_CG_4_8_14_3_um_filter_51_11
delta_proteobacterium_NaphS2
Desulfobacteraceae
Desulfatibacillum_aliphaticivorans
Candidatus_Magnetoglobus_multicellularis_str_Araruama
Desulfobacula_sp_TS
Candidatus_Magnetomorum_sp_HK-1
Desulfotignum
Desulfotignum_phosphitoxidans
Desulfotignum_balticum

CBU_1907

A **B**

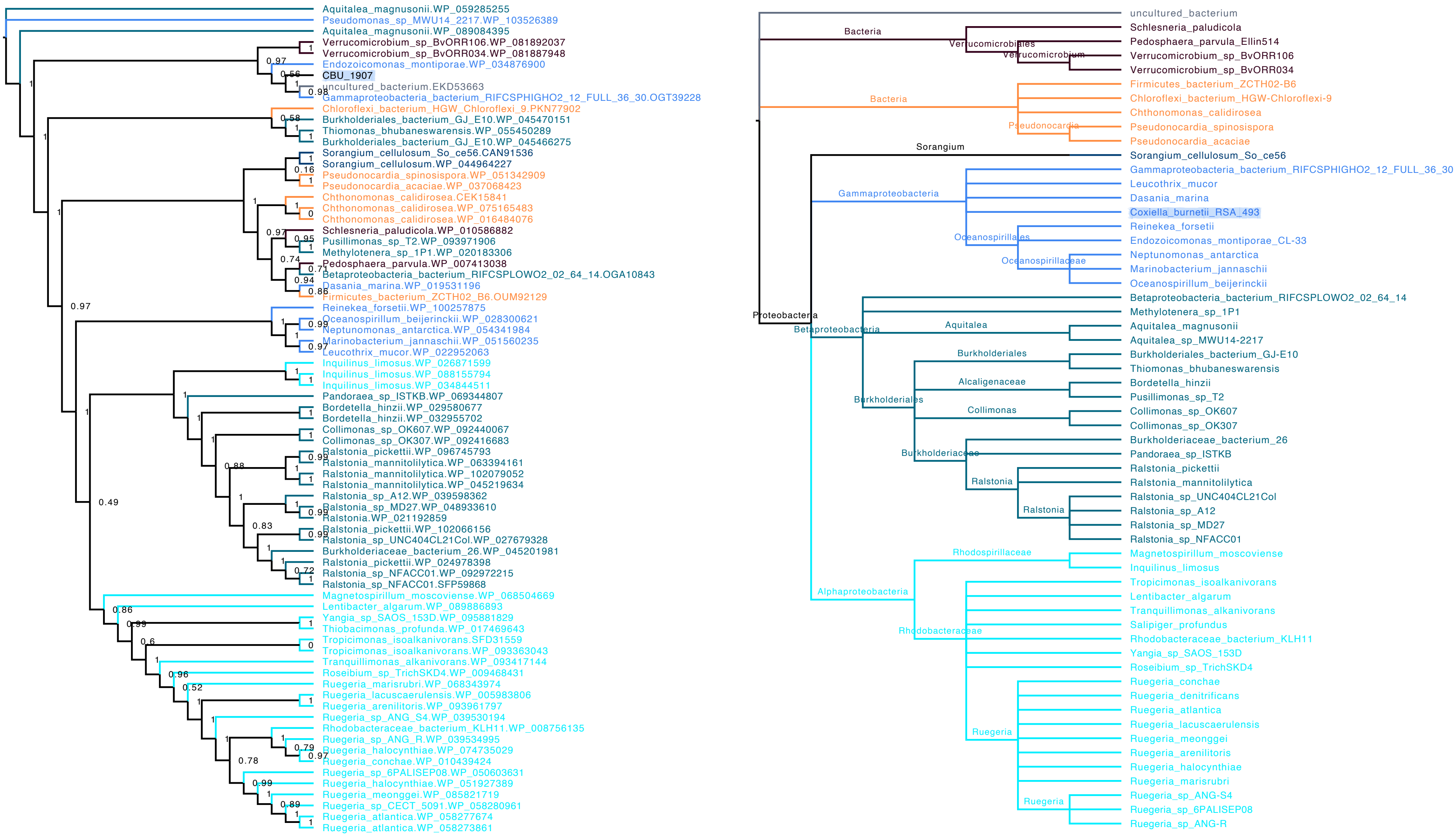

Aquitalea_magnusonii.WP_059285255
Pseudomonas_sp_MWU14_2217.WP_103526389
Aquitalea_magnusonii.WP_089084395
Verrucomicrobium_sp_BvORR106.WP_081892037
Verrucomicrobium_sp_BvORR034.WP_081887948
Endozoicomonas_montiporae.WP_034876900
CBU_1907
uncultured_bacterium.EKD53663
Gammaproteobacteria_bacterium_RIFCSPHIGHO2_12_FULL_36_30.OGT39228
Chloroflexi_bacterium_HGW_Chloroflexi_9.PKN77902
Burkholderiales_bacterium_GJ_E10.WP_045470151
Thiomonas_bhubaneswarensis.WP_055450289
Burkholderiales_bacterium_GJ_E10.WP_045466275
Sorangium_cellulosum_So_ce56.CAN91536
Sorangium_cellulosum.WP_044964227
Pseudonocardia_spinosispora.WP_051342909
Pseudonocardia_acaciae.WP_037068423
Chthonomonas_calidirosea.CEK15841
Chthonomonas_calidirosea.WP_075165483
Chthonomonas_calidirosea.WP_016484076
Schlesneria_paludicola.WP_010586882
Pusillimonas_sp_T2.WP_093971906
Methylotenera_sp_1P1.WP_020183306
Pedosphaera_parvula.WP_007413038
Betaproteobacteria_bacterium_RIFCSPLOWO2_02_64_14.OGA10843
Dasania_marina.WP_019531196
Firmicutes_bacterium_ZCTH02_B6.OUM92129
Reinekea_forsetii.WP_100257875
Oceanospirillum_beijerinckii.WP_028300621
Neptunomonas_antarctica.WP_054341984
Marinobacterium_jannaschii.WP_051560235
Leucothrix_mucor.WP_022952063
Inquilinus_limosus.WP_026871599
Inquilinus_limosus.WP_088155794
Inquilinus_limosus.WP_034844511
Pandoraea_sp_ISTKB.WP_069344807
Bordetella_hinzii.WP_029580677
Bordetella_hinzii.WP_032955702
Collimonas_sp_OK607.WP_092440067
Collimonas_sp_OK307.WP_092416683
Ralstonia_pickettii.WP_096745793
Ralstonia_mannitolilytica.WP_063394161
Ralstonia_mannitolilytica.WP_102079052
Ralstonia_mannitolilytica.WP_045219634
Ralstonia_sp_A12.WP_039598362
Ralstonia_sp_MD27.WP_048933610
Ralstonia.WP_021192859
Ralstonia_pickettii.WP_102066156
Ralstonia_sp_UNC404CL21Col.WP_027679328
Burkholderiaceae_bacterium_26.WP_045201981
Ralstonia_pickettii.WP_024978398
Ralstonia_sp_NFACC01.WP_092972215
Ralstonia_sp_NFACC01.SFP59868
Magnetospirillum_moscoviense.WP_068504669
Lentibacter_algarum.WP_089886893
Yangia_sp_SAOS_153D.WP_095881829
Thiobacimonas_profunda.WP_017469643
Tropicimonas_isoalkanivorans.SFD31559
Tropicimonas_isoalkanivorans.WP_093363043
Tranquillimonas_alkanivorans.WP_093417144
Roseibium_sp_TrichSKD4.WP_009468431
Ruegeria_marisrubri.WP_068343974
Ruegeria_lacuscaerulensis.WP_005983806
Ruegeria_arenilitoris.WP_093961797
Ruegeria_sp_ANG_S4.WP_039530194
Rhodobacteraceae_bacterium_KLH11.WP_008756135
Ruegeria_sp_ANG_R.WP_039534995
Ruegeria_halocynthiae.WP_074735029
Ruegeria_conchae.WP_010439424
Ruegeria_sp_6PALISEP08.WP_050603631
Ruegeria_halocynthiae.WP_051927389
Ruegeria_meonggei.WP_085821719
Ruegeria_sp_CECT_5091.WP_058280961
Ruegeria_atlantica.WP_058277674
Ruegeria_atlantica.WP_058273861
uncultured_bacterium
Bacteria
Schlesneria_paludicola
Verrucomicrobiales
Pedosphaera_parvula_Ellin514
Verrucomicrobium
Verrucomicrobium_sp_BvORR106
Verrucomicrobium_sp_BvORR034
Firmicutes_bacterium_ZCTH02-B6
Bacteria
Chloroflexi_bacterium_HGW-Chloroflexi-9
Chthonomonas_calidirosea
Pseudonocardia
Pseudonocardia_spinosispora
Pseudonocardia_acaciae
Sorangium
Sorangium_cellulosum_So_ce56
Gammaproteobacteria_bacterium_RIFCSPHIGHO2_12_FULL_36_30
Gammaproteobacteria
Leucothrix_mucor
Dasania_marina
Coxiella_burnetii_RSA_493
Reinekea_forsetii
Oceanospirillales
Endozoicomonas_montiporae_CL-33
Neptunomonas_antarctica
Oceanospirillaceae
Marinobacterium_jannaschii
Oceanospirillum_beijerinckii
Betaproteobacteria_bacterium_RIFCSPLOWO2_02_64_14
Methylotenera_sp_1P1
Proteobacteria
Aquitalea
Aquitalea_magnusonii
Betaproteobacteria
Aquitalea_sp_MWU14-2217
Burkholderiales
Burkholderiales_bacterium_GJ-E10
Thiomonas_bhubaneswarensis
Alcaligenaceae
Bordetella_hinzii
Burkholderiales
Pusillimonas_sp_T2
Collimonas
Collimonas_sp_OK607
Collimonas_sp_OK307
Burkholderiaceae_bacterium_26
Burkholderiaceae
Pandoraea_sp_ISTKB
Ralstonia_pickettii
Ralstonia
Ralstonia_mannitolilytica
Ralstonia_sp_UNC404CL21Col
Ralstonia
Ralstonia_sp_A12
Ralstonia_sp_MD27
Ralstonia_sp_NFACC01
Rhodospirillaceae
Magnetospirillum_moscoviense
Inquilinus_limosus
Tropicimonas_isoalkanivorans
Alphaproteobacteria
Lentibacter_algarum
Tranquillimonas_alkanivorans
Salipiger_profundus
Rhodobacteraceae
Rhodobacteraceae_bacterium_KLH11
Yangia_sp_SAOS_153D
Roseibium_sp_TrichSKD4
Ruegeria_conchae
Ruegeria_denitrificans
Ruegeria_atlantica
Ruegeria_lacuscaerulensis
Ruegeria
Ruegeria_meonggei
Ruegeria_arenilitoris
Ruegeria_halocynthiae
Ruegeria_marisrubri
Ruegeria_sp_ANG-S4
Ruegeria
Ruegeria_sp_6PALISEP08
Ruegeria_sp_ANG-R

CBU_1850

A

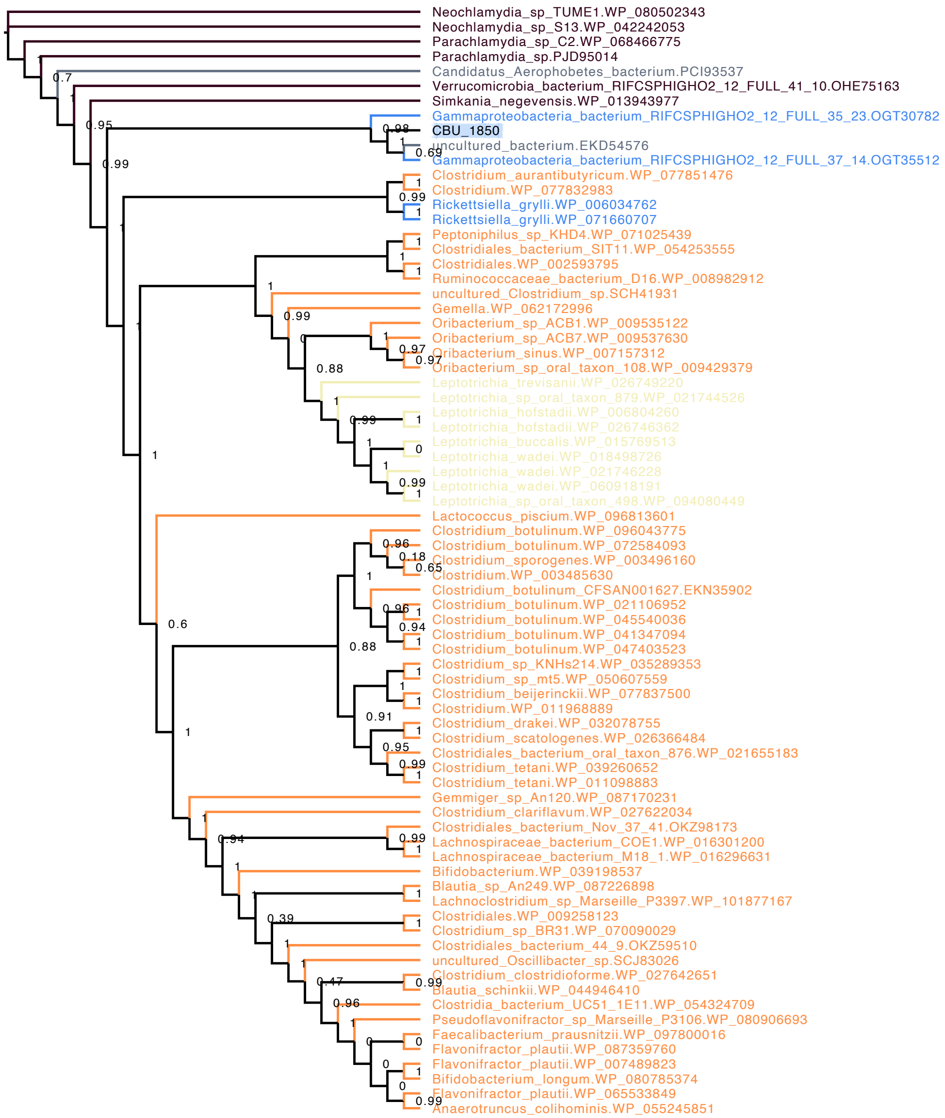

Neochlamydia_sp_TUME1.WP_080502343
Neochlamydia_sp_S13.WP_042242053
Parachlamydia_sp_C2.WP_068466775
Parachlamydia_sp.PJD95014
Candidatus_Aerophobetes_bacterium.PCI93537
Verrucomicrobia_bacterium_RIFCSPHIGHO2_12_FULL_41_10.OHE75163
Simkania_negevensis.WP_013943977
Gammaproteobacteria_bacterium_RIFCSPHIGHO2_12_FULL_35_23.OGT30782
CBU_1850
uncultured_bacterium.EKD54576
Gammaproteobacteria_bacterium_RIFCSPHIGHO2_12_FULL_37_14.OGT35512
Clostridium_aurantibutyricum.WP_077851476
Clostridium.WP_077832983
Rickettsiella_grylli.WP_006034762
Rickettsiella_grylli.WP_071660707
Peptoniphilus_sp_KHD4.WP_071025439
Clostridiales_bacterium_SIT11.WP_054253555
Clostridiales.WP_002593795
Ruminococcaceae_bacterium_D16.WP_008982912
uncultured_Clostridium_sp.SCH41931
Gemella.WP_062172996
Oribacterium_sp_ACB1.WP_009535122
Oribacterium_sp_ACB7.WP_009537630
Oribacterium_sinus.WP_007157312
Oribacterium_sp_oral_taxon_108.WP_009429379
Leptotrichia_trevisanii.WP_026749220
Leptotrichia_sp_oral_taxon_879.WP_021744526
Leptotrichia_hofstadii.WP_006804260
Leptotrichia_hofstadii.WP_026746362
Leptotrichia_buccalis.WP_015769513
Leptotrichia_wadei.WP_018498726
Leptotrichia_wadei.WP_021746228
Leptotrichia_wadei.WP_060918191
Leptotrichia_sp_oral_taxon_498.WP_094080449
Lactococcus_piscium.WP_096813601
Clostridium_botulinum.WP_096043775
Clostridium_botulinum.WP_072584093
Clostridium_sporogenes.WP_003496160
Clostridium.WP_003485630
Clostridium_botulinum_CFSAN001627.EKN35902
Clostridium_botulinum.WP_021106952
Clostridium_botulinum.WP_045540036
Clostridium_botulinum.WP_041347094
Clostridium_botulinum.WP_047403523
Clostridium_sp_KNHs214.WP_035289353
Clostridium_sp_mt5.WP_050607559
Clostridium_beijerinckii.WP_077837500
Clostridium.WP_011968889
Clostridium_drakei.WP_032078755
Clostridium_scatologenes.WP_026366484
Clostridiales_bacterium_oral_taxon_876.WP_021655183
Clostridium_tetani.WP_039260652
Clostridium_tetani.WP_011098883
Gemmiger_sp_An120.WP_087170231
Clostridium_clariflavum.WP_027622034
Clostridiales_bacterium_Nov_37_41.OKZ98173
Lachnospiraceae_bacterium_COE1.WP_016301200
Lachnospiraceae_bacterium_M18_1.WP_016296631
Bifidobacterium.WP_039198537
Blautia_sp_An249.WP_087226898
Lachnoclostridium_sp_Marseille_P3397.WP_101877167
Clostridiales.WP_009258123
Clostridium_sp_BR31.WP_070090029
Clostridiales_bacterium_44_9.OKZ59510
uncultured_Oscillibacter_sp.SCJ83026
Clostridium_clostridioforme.WP_027642651
Blautia_schinkii.WP_044946410
Clostridia_bacterium_UC51_1E11.WP_054324709
Pseudoflavonifractor_sp_Marseille_P3106.WP_080906693
Faecalibacterium_prausnitzii.WP_097800016
Flavonifractor_plautii.WP_087359760
Flavonifractor_plautii.WP_007489823
Bifidobacterium_longum.WP_080785374
Flavonifractor_plautii.WP_065533849
Anaerotruncus_colihominis.WP_055245851

B

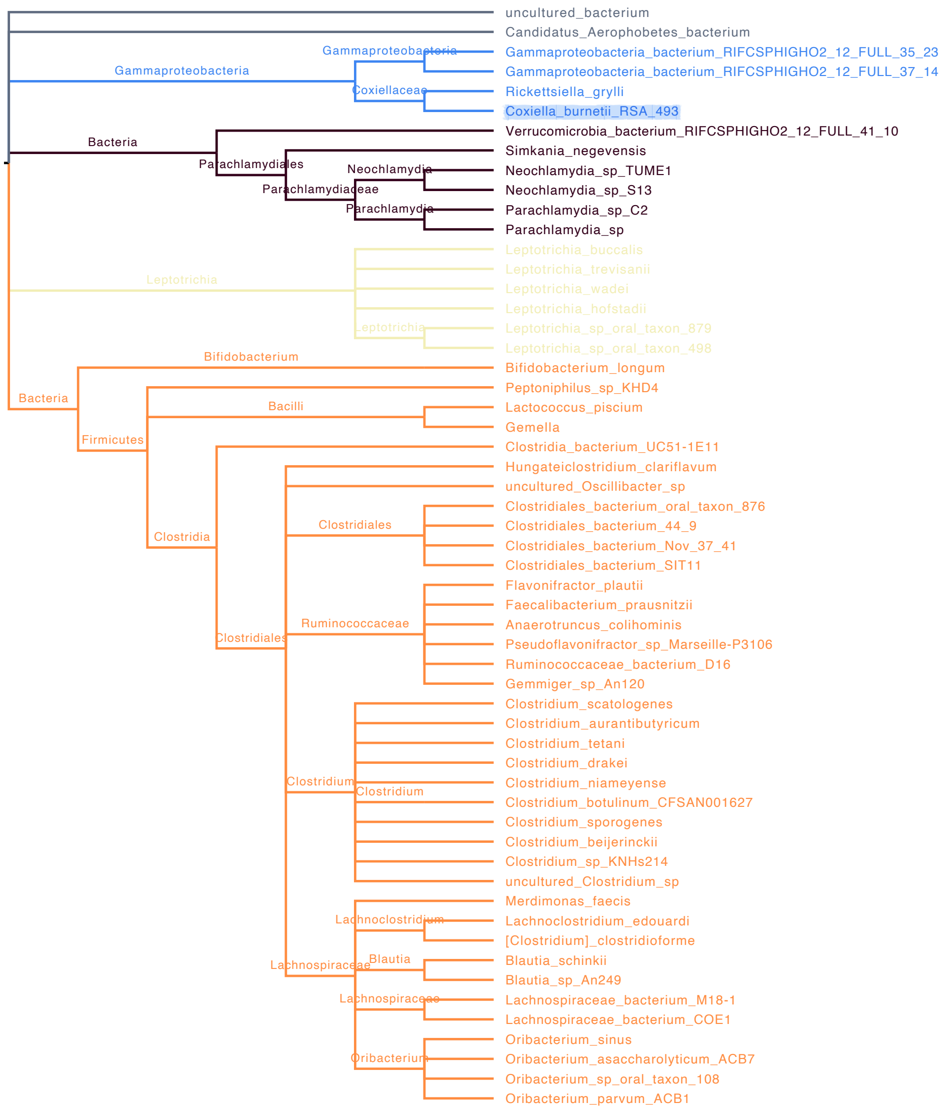

uncultured_bacterium
Candidatus_Aerophobetes_bacterium
Gammaproteobacteria
Gammaproteobacteria
Coxiellaceae
Gammaproteobacteria_bacterium_RIFCSPHIGHO2_12_FULL_35_23
Gammaproteobacteria_bacterium_RIFCSPHIGHO2_12_FULL_37_14
Rickettsiella_grylli
Coxiella_burnetii_RSA_493
Bacteria
Parachlamydiales
Neochlamydia
Parachlamydiaceae
Parachlamydia
Verrucomicrobia_bacterium_RIFCSPHIGHO2_12_FULL_41_10
Simkania_negevensis
Neochlamydia_sp_TUME1
Neochlamydia_sp_S13
Parachlamydia_sp_C2
Parachlamydia_sp
Leptotrichia
Leptotrichia
Leptotrichia_buccalis
Leptotrichia_trevisanii
Leptotrichia_wadei
Leptotrichia_hofstadii
Leptotrichia_sp_oral_taxon_879
Leptotrichia_sp_oral_taxon_498
Bacteria
Bifidobacterium
Firmicutes
Bacilli
Clostridia
Bifidobacterium_longum
Peptoniphilus_sp_KHD4
Lactococcus_piscium
Gemella
Clostridia_bacterium_UC51-1E11
Hungateiclostridium_clariflavum
uncultured_Oscillibacter_sp
Clostridiales
Clostridiales_bacterium_oral_taxon_876
Clostridiales_bacterium_44_9
Clostridiales_bacterium_Nov_37_41
Clostridiales_bacterium_SIT11
Ruminococcaceae
Clostridiales
Flavonifractor_plautii
Faecalibacterium_prausnitzii
Anaerotruncus_colihominis
Pseudoflavonifractor_sp_Marseille-P3106
Ruminococcaceae_bacterium_D16
Gemmiger_sp_An120
Clostridium
Clostridium
Clostridium_scatologenes
Clostridium_aurantibutyricum
Clostridium_tetani
Clostridium_drakei
Clostridium_niameyense
Clostridium_botulinum_CFSAN001627
Clostridium_sporogenes
Clostridium_beijerinckii
Clostridium_sp_KNHs214
uncultured_Clostridium_sp
Lachnoclostridium
Lachnospiraceae
Blautia
Lachnospiraceae
Oribacterium
Merdimonas_faecis
Lachnoclostridium_edouardi
[Clostridium]_clostridioforme
Blautia_schinkii
Blautia_sp_An249
Lachnospiraceae_bacterium_M18-1
Lachnospiraceae_bacterium_COE1
Oribacterium_sinus
Oribacterium_asaccharolyticum_ACB7
Oribacterium_sp_oral_taxon_108
Oribacterium_parvum_ACB1

CBU_1799

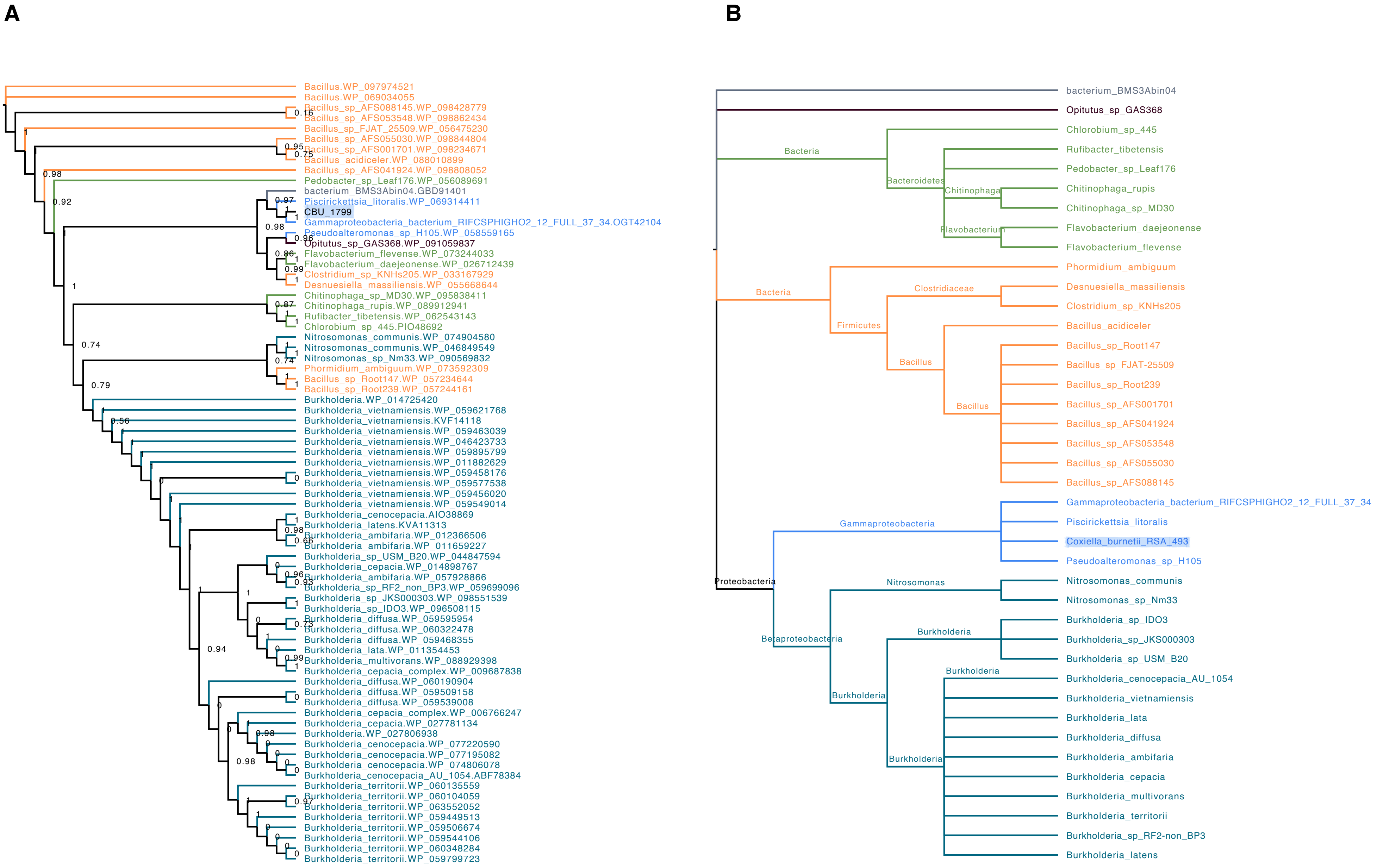

A
Bacillus.WP_097974521
Bacillus.WP_069034055
Bacillus_sp_AFS088145.WP_098428779
Bacillus_sp_AFS053548.WP_098862434
Bacillus_sp_FJAT_25509.WP_056475230
Bacillus_sp_AFS055030.WP_098844804
Bacillus_sp_AFS001701.WP_098234671
Bacillus_acidiceler.WP_088010899
Bacillus_sp_AFS041924.WP_098808052
Pedobacter_sp_Leaf176.WP_056089691
bacterium_BMS3Abin04.GBD91401
Piscirickettsia_litoralis.WP_069314411
CBU_1799
Gammaproteobacteria_bacterium_RIFCSPHIGHO2_12_FULL_37_34.OGT42104
Pseudoalteromonas_sp_H105.WP_058559165
Opitutus_sp_GAS368.WP_091059837
Flavobacterium_flevense.WP_073244033
Flavobacterium_daejeonense.WP_026712439
Clostridium_sp_KNHs205.WP_033167929
Desnuesiella_massiliensis.WP_055668644
Chitinophaga_sp_MD30.WP_095838411
Chitinophaga_rupis.WP_089912941
Rufibacter_tibetensis.WP_062543143
Chlorobium_sp_445.PIO48692
Nitrosomonas_communis.WP_074904580
Nitrosomonas_communis.WP_046849549
Nitrosomonas_sp_Nm33.WP_090569832
Phormidium_ambiguum.WP_073592309
Bacillus_sp_Root147.WP_057234644
Bacillus_sp_Root239.WP_057244161
Burkholderia.WP_014725420
Burkholderia_vietnamiensis.WP_059621768
Burkholderia_vietnamiensis.KVF14118
Burkholderia_vietnamiensis.WP_059463039
Burkholderia_vietnamiensis.WP_046423733
Burkholderia_vietnamiensis.WP_059895799
Burkholderia_vietnamiensis.WP_011882629
Burkholderia_vietnamiensis.WP_059458176
Burkholderia_vietnamiensis.WP_059577538
Burkholderia_vietnamiensis.WP_059456020
Burkholderia_vietnamiensis.WP_059549014
Burkholderia_cenocepacia.AIO38869
Burkholderia_latens.KVA11313
Burkholderia_ambifaria.WP_012366506
Burkholderia_ambifaria.WP_011659227
Burkholderia_sp_USM_B20.WP_044847594
Burkholderia_cepacia.WP_014898767
Burkholderia_ambifaria.WP_057928866
Burkholderia_sp_RF2_non_BP3.WP_059699096
Burkholderia_sp_JKS000303.WP_098551539
Burkholderia_sp_IDO3.WP_096508115
Burkholderia_diffusa.WP_059595954
Burkholderia_diffusa.WP_060322478
Burkholderia_diffusa.WP_059468355
Burkholderia_lata.WP_011354453
Burkholderia_multivorans.WP_088929398
Burkholderia_cepacia_complex.WP_009687838
Burkholderia_diffusa.WP_060190904
Burkholderia_diffusa.WP_059509158
Burkholderia_diffusa.WP_059539008
Burkholderia_cepacia_complex.WP_006766247
Burkholderia_cepacia.WP_027781134
Burkholderia.WP_027806938
Burkholderia_cenocepacia.WP_077220590
Burkholderia_cenocepacia.WP_077195082
Burkholderia_cenocepacia.WP_074806078
Burkholderia_cenocepacia_AU_1054.ABF78384
Burkholderia_territorii.WP_060135559
Burkholderia_territorii.WP_060104059
Burkholderia_territorii.WP_063552052
Burkholderia_territorii.WP_059449513
Burkholderia_territorii.WP_059506674
Burkholderia_territorii.WP_059544106
Burkholderia_territorii.WP_060348284
Burkholderia_territorii.WP_059799723
B
bacterium_BMS3Abin04
Opitutus_sp_GAS368
Bacteria
Bacteroidetes
Chitinophaga
Flavobacterium
Chlorobium_sp_445
Rufibacter_tibetensis
Pedobacter_sp_Leaf176
Chitinophaga_rupis
Chitinophaga_sp_MD30
Flavobacterium_daejeonense
Flavobacterium_flevense
Bacteria
Firmicutes
Clostridiaceae
Bacillus
Bacillus
Phormidium_ambiguum
Desnuesiella_massiliensis
Clostridium_sp_KNHs205
Bacillus_acidiceler
Bacillus_sp_Root147
Bacillus_sp_FJAT-25509
Bacillus_sp_Root239
Bacillus_sp_AFS001701
Bacillus_sp_AFS041924
Bacillus_sp_AFS053548
Bacillus_sp_AFS055030
Bacillus_sp_AFS088145
Proteobacteria
Gammaproteobacteria
Gammaproteobacteria_bacterium_RIFCSPHIGHO2_12_FULL_37_34
Piscirickettsia_litoralis
Coxiella_burnetii_RSA_493
Pseudoalteromonas_sp_H105
Betaproteobacteria
Nitrosomonas
Nitrosomonas_communis
Nitrosomonas_sp_Nm33
Burkholderia
Burkholderia
Burkholderia
Burkholderia
Burkholderia_sp_IDO3
Burkholderia_sp_JKS000303
Burkholderia_sp_USM_B20
Burkholderia_cenocepacia_AU_1054
Burkholderia_vietnamiensis
Burkholderia_lata
Burkholderia_diffusa
Burkholderia_ambifaria
Burkholderia_cepacia
Burkholderia_multivorans
Burkholderia_territorii
Burkholderia_sp_RF2-non_BP3
Burkholderia_latens

CBU_1788

A

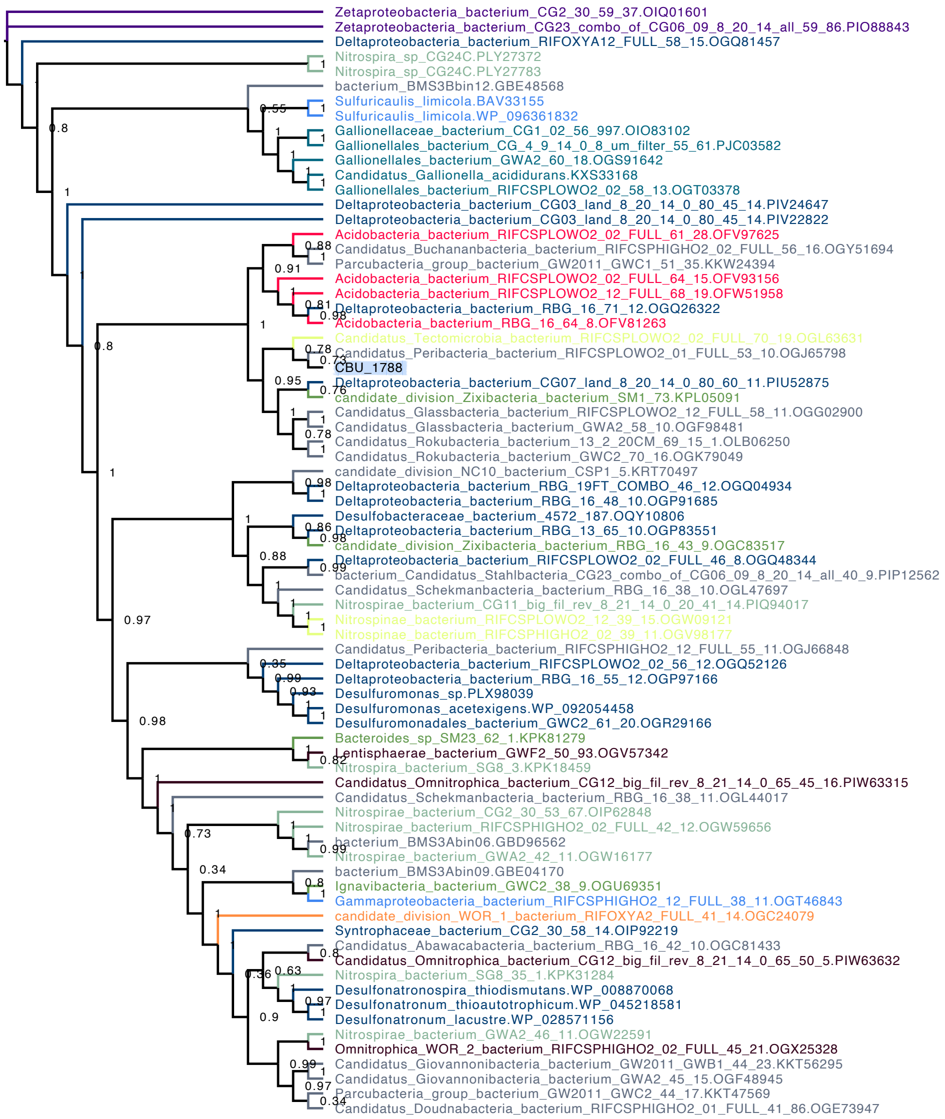

Zetaproteobacteria_bacterium_CG2_30_59_37.OIQ01601
Zetaproteobacteria_bacterium_CG23_combo_of_CG06_09_8_20_14_all_59_86.PIO88843
Deltaproteobacteria_bacterium_RIFOXYA12_FULL_58_15.OGQ81457
Nitrospira_sp_CG24C.PLY27372
Nitrospira_sp_CG24C.PLY27783
bacterium_BMS3Bbin12.GBE48568
Sulfuricaulis_limicola.BAV33155
Sulfuricaulis_limicola.WP_096361832
Gallionellaceae_bacterium_CG1_02_56_997.OIO83102
Gallionellales_bacterium_CG_4_9_14_0_8_um_filter_55_61.PJC03582
Gallionellales_bacterium_GWA2_60_18.OGS91642
Candidatus_Gallionella_acididurans.KXS33168
Gallionellales_bacterium_RIFCSPLOWO2_02_58_13.OGT03378
Deltaproteobacteria_bacterium_CG03_land_8_20_14_0_80_45_14.PIV24647
Deltaproteobacteria_bacterium_CG03_land_8_20_14_0_80_45_14.PIV22822
Acidobacteria_bacterium_RIFCSPLOWO2_02_FULL_61_28.OFV97625
Candidatus_Buchananbacteria_bacterium_RIFCSPHIGHO2_02_FULL_56_16.OGY51694
Parcubacteria_group_bacterium_GW2011_GWC1_51_35.KKW24394
Acidobacteria_bacterium_RIFCSPLOWO2_02_FULL_64_15.OFV93156
Acidobacteria_bacterium_RIFCSPLOWO2_12_FULL_68_19.OFW51958
Deltaproteobacteria_bacterium_RBG_16_71_12.OGQ26322
Acidobacteria_bacterium_RBG_16_64_8.OFV81263
Candidatus_Tectomicrobia_bacterium_RIFCSPLOWO2_02_FULL_70_19.OGL63631
Candidatus_Peribacteria_bacterium_RIFCSPLOWO2_01_FULL_53_10.OGJ65798
CBU_1788
Deltaproteobacteria_bacterium_CG07_land_8_20_14_0_80_60_11.PIU52875
candidate_division_Zixibacteria_bacterium_SM1_73.KPL05091
Candidatus_Glassbacteria_bacterium_RIFCSPLOWO2_12_FULL_58_11.OGG02900
Candidatus_Glassbacteria_bacterium_GWA2_58_10.OGF98481
Candidatus_Rokubacteria_bacterium_13_2_20CM_69_15_1.OLB06250
Candidatus_Rokubacteria_bacterium_GWC2_70_16.OGK79049
candidate_division_NC10_bacterium_CSP1_5.KRT70497
Deltaproteobacteria_bacterium_RBG_19FT_COMBO_46_12.OGQ04934
Deltaproteobacteria_bacterium_RBG_16_48_10.OGP91685
Desulfobacteraceae_bacterium_4572_187.OQY10806
Deltaproteobacteria_bacterium_RBG_13_65_10.OGP83551
candidate_division_Zixibacteria_bacterium_RBG_16_43_9.OGC83517
Deltaproteobacteria_bacterium_RIFCSPLOWO2_02_FULL_46_8.OGQ48344
bacterium_Candidatus_Stahlbacteria_CG23_combo_of_CG06_09_8_20_14_all_40_9.PIP12562
Candidatus_Schekmanbacteria_bacterium_RBG_16_38_10.OGL47697
Nitrospirae_bacterium_CG11_big_fil_rev_8_21_14_0_20_41_14.PIQ94017
Nitrospinae_bacterium_RIFCSPLOWO2_12_39_15.OGW09121
Nitrospinae_bacterium_RIFCSPHIGHO2_02_39_11.OGV98177
Candidatus_Peribacteria_bacterium_RIFCSPHIGHO2_12_FULL_55_11.OGJ66848
Deltaproteobacteria_bacterium_RIFCSPLOWO2_02_56_12.OGQ52126
Deltaproteobacteria_bacterium_RBG_16_55_12.OGP97166
Desulfuromonas_sp.PLX98039
Desulfuromonas_acetexigens.WP_092054458
Desulfuromonadales_bacterium_GWC2_61_20.OGR29166
Bacteroides_sp_SM23_62_1.KPK81279
Lentisphaerae_bacterium_GWF2_50_93.OGV57342
Nitrospira_bacterium_SG8_3.KPK18459
Candidatus_Omnitrophica_bacterium_CG12_big_fil_rev_8_21_14_0_65_45_16.PIW63315
Candidatus_Schekmanbacteria_bacterium_RBG_16_38_11.OGL44017
Nitrospirae_bacterium_CG2_30_53_67.OIP62848
Nitrospirae_bacterium_RIFCSPHIGHO2_02_FULL_42_12.OGW59656
bacterium_BMS3Abin06.GBD96562
Nitrospirae_bacterium_GWA2_42_11.OGW16177
bacterium_BMS3Abin09.GBE04170
Ignavibacteria_bacterium_GWC2_38_9.OGU69351
Gammaproteobacteria_bacterium_RIFCSPHIGHO2_12_FULL_38_11.OGT46843
candidate_division_WOR_1_bacterium_RIFOXYA2_FULL_41_14.OGC24079
Syntrophaceae_bacterium_CG2_30_58_14.OIP92219
Candidatus_Abawacabacteria_bacterium_RBG_16_42_10.OGC81433
Candidatus_Omnitrophica_bacterium_CG12_big_fil_rev_8_21_14_0_65_50_5.PIW63632
Nitrospira_bacterium_SG8_35_1.KPK31284
Desulfonatronospira_thiodismutans.WP_008870068
Desulfonatronum_thioautotrophicum.WP_045218581
Desulfonatronum_lacustre.WP_028571156
Nitrospirae_bacterium_GWA2_46_11.OGW22591
Omnitrophica_WOR_2_bacterium_RIFCSPHIGHO2_02_FULL_45_21.OGX25328
Candidatus_Giovannonibacteria_bacterium_GW2011_GWB1_44_23.KKT56295
Candidatus_Giovannonibacteria_bacterium_GWA2_45_15.OGF48945
Parcubacteria_group_bacterium_GW2011_GWC2_44_17.KKT47569
Candidatus_Doudnabacteria_bacterium_RIFCSPHIGHO2_01_FULL_41_86.OGE73947

B

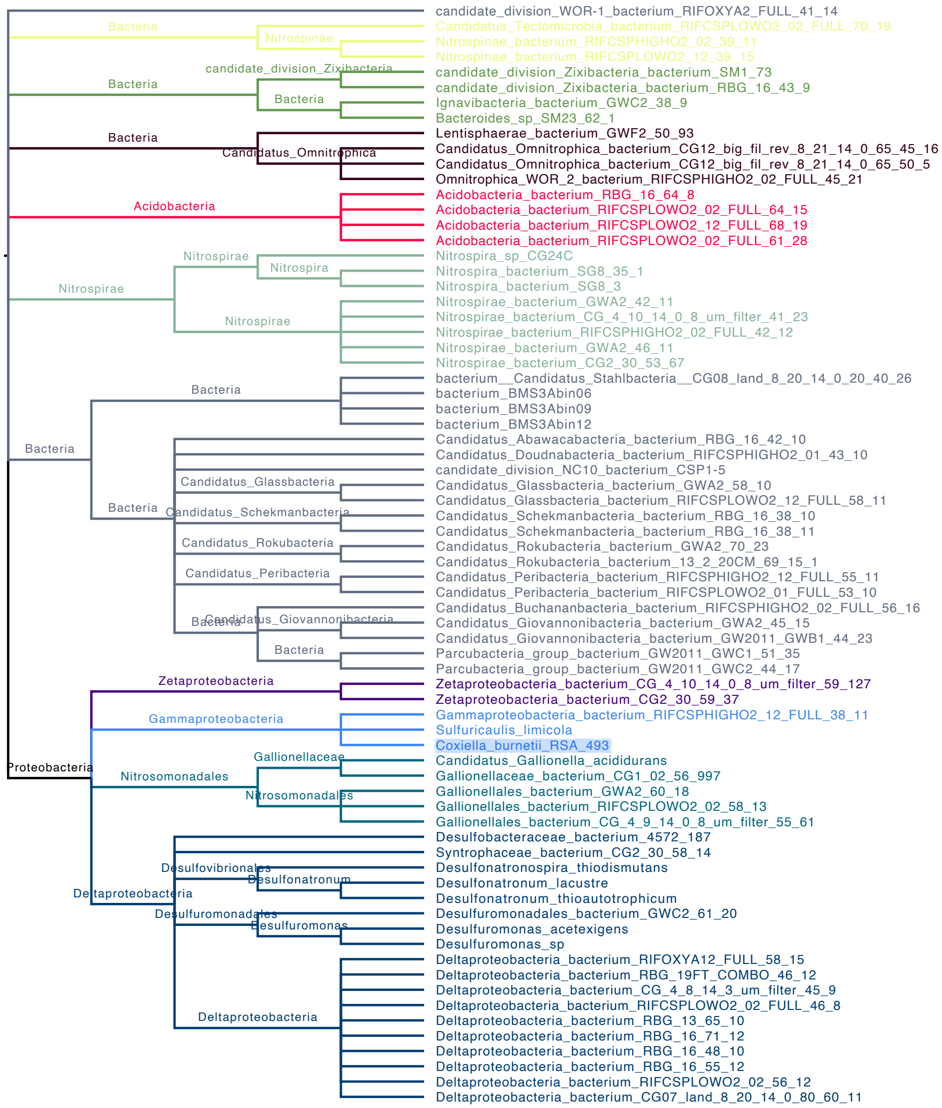

candidate_division_WOR-1_bacterium_RIFOXYA2_FULL_41_14
Bacteria
Nitrospinae
Candidatus_Tectomicrobia_bacterium_RIFCSPLOWO2_02_FULL_70_19
Nitrospinae_bacterium_RIFCSPHIGHO2_02_39_11
Nitrospinae_bacterium_RIFCSPLOWO2_12_39_15
candidate_division_Zixibacteria
candidate_division_Zixibacteria_bacterium_SM1_73
candidate_division_Zixibacteria_bacterium_RBG_16_43_9
Ignavibacteria_bacterium_GWC2_38_9
Bacteroides_sp_SM23_62_1
Candidatus_Omnitrophica
Lentisphaerae_bacterium_GWF2_50_93
Candidatus_Omnitrophica_bacterium_CG12_big_fil_rev_8_21_14_0_65_45_16
Candidatus_Omnitrophica_bacterium_CG12_big_fil_rev_8_21_14_0_65_50_5
Omnitrophica_WOR_2_bacterium_RIFCSPHIGHO2_02_FULL_45_21
Acidobacteria
Acidobacteria_bacterium_RBG_16_64_8
Acidobacteria_bacterium_RIFCSPLOWO2_02_FULL_64_15
Acidobacteria_bacterium_RIFCSPLOWO2_12_FULL_68_19
Acidobacteria_bacterium_RIFCSPLOWO2_02_FULL_61_28
Nitrospirae
Nitrospira
Nitrospira_sp_CG24C
Nitrospira_bacterium_SG8_35_1
Nitrospira_bacterium_SG8_3
Nitrospirae_bacterium_GWA2_42_11
Nitrospirae_bacterium_CG_4_10_14_0_8_um_filter_41_23
Nitrospirae_bacterium_RIFCSPHIGHO2_02_FULL_42_12
Nitrospirae_bacterium_GWA2_46_11
Nitrospirae_bacterium_CG2_30_53_67
bacterium__Candidatus_Stahlbacteria__CG08_land_8_20_14_0_20_40_26
bacterium_BMS3Abin06
bacterium_BMS3Abin09
bacterium_BMS3Abin12
Candidatus_Abawacabacteria_bacterium_RBG_16_42_10
Candidatus_Doudnabacteria_bacterium_RIFCSPHIGHO2_01_43_10
candidate_division_NC10_bacterium_CSP1-5
Candidatus_Glassbacteria
Candidatus_Glassbacteria_bacterium_GWA2_58_10
Candidatus_Glassbacteria_bacterium_RIFCSPLOWO2_12_FULL_58_11
Candidatus_Schekmanbacteria
Candidatus_Schekmanbacteria_bacterium_RBG_16_38_10
Candidatus_Schekmanbacteria_bacterium_RBG_16_38_11
Candidatus_Rokubacteria
Candidatus_Rokubacteria_bacterium_GWA2_70_23
Candidatus_Rokubacteria_bacterium_13_2_20CM_69_15_1
Candidatus_Peribacteria
Candidatus_Peribacteria_bacterium_RIFCSPHIGHO2_12_FULL_55_11
Candidatus_Peribacteria_bacterium_RIFCSPLOWO2_01_FULL_53_10
Candidatus_Buchananbacteria_bacterium_RIFCSPHIGHO2_02_FULL_56_16
Candidatus_Giovannonibacteria
Candidatus_Giovannonibacteria_bacterium_GWA2_45_15
Candidatus_Giovannonibacteria_bacterium_GW2011_GWB1_44_23
Parcubacteria_group_bacterium_GW2011_GWC1_51_35
Parcubacteria_group_bacterium_GW2011_GWC2_44_17
Proteobacteria
Zetaproteobacteria
Zetaproteobacteria_bacterium_CG_4_10_14_0_8_um_filter_59_127
Zetaproteobacteria_bacterium_CG2_30_59_37
Gammaproteobacteria
Gammaproteobacteria_bacterium_RIFCSPHIGHO2_12_FULL_38_11
Sulfuricaulis_limicola
Coxiella_burnetii_RSA_493
Nitrosomonadales
Gallionellaceae
Candidatus_Gallionella_acididurans
Gallionellaceae_bacterium_CG1_02_56_997
Gallionellales_bacterium_GWA2_60_18
Gallionellales_bacterium_RIFCSPLOWO2_02_58_13
Gallionellales_bacterium_CG_4_9_14_0_8_um_filter_55_61
Deltaproteobacteria
Desulfovibrionales
Desulfonatronum
Desulfuromonadales
Desulfuromonas
Desulfobacteraceae_bacterium_4572_187
Syntrophaceae_bacterium_CG2_30_58_14
Desulfonatronospira_thiodismutans
Desulfonatronum_lacustre
Desulfonatronum_thioautotrophicum
Desulfuromonadales_bacterium_GWC2_61_20
Desulfuromonas_acetexigens
Desulfuromonas_sp
Deltaproteobacteria_bacterium_RIFOXYA12_FULL_58_15
Deltaproteobacteria_bacterium_RBG_19FT_COMBO_46_12
Deltaproteobacteria_bacterium_CG_4_8_14_3_um_filter_45_9
Deltaproteobacteria_bacterium_RIFCSPLOWO2_02_FULL_46_8
Deltaproteobacteria_bacterium_RBG_13_65_10
Deltaproteobacteria_bacterium_RBG_16_71_12
Deltaproteobacteria_bacterium_RBG_16_48_10
Deltaproteobacteria_bacterium_RBG_16_55_12
Deltaproteobacteria_bacterium_RIFCSPLOWO2_02_56_12
Deltaproteobacteria_bacterium_CG07_land_8_20_14_0_80_60_11

CBU_1786

A **B**

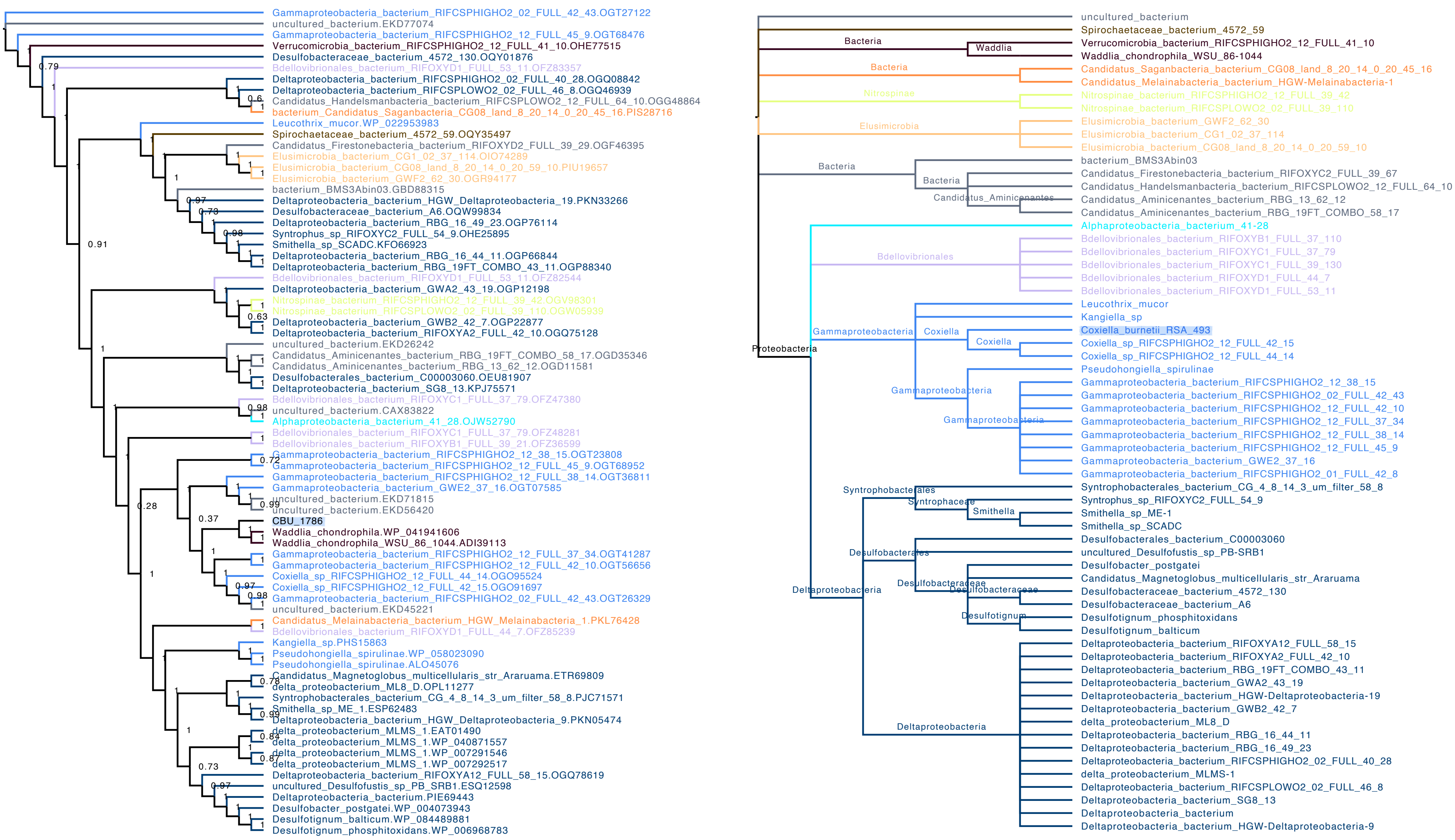

Gammaproteobacteria_bacterium_RIFCSPHIGHO2_02_FULL_42_43.OGT27122
uncultured_bacterium.EKD77074
Gammaproteobacteria_bacterium_RIFCSPHIGHO2_12_FULL_45_9.OGT68476
Verrucomicrobia_bacterium_RIFCSPHIGHO2_12_FULL_41_10.OHE77515
Desulfobacteraceae_bacterium_4572_130.OQY01876
Bdellovibrionales_bacterium_RIFOXYD1_FULL_53_11.OFZ83357
Deltaproteobacteria_bacterium_RIFCSPHIGHO2_02_FULL_40_28.OGQ08842
Deltaproteobacteria_bacterium_RIFCSPLOWO2_02_FULL_46_8.OGQ46939
Candidatus_Handelsmanbacteria_bacterium_RIFCSPLOWO2_12_FULL_64_10.OGG48864
bacterium_Candidatus_Saganbacteria_CG08_land_8_20_14_0_20_45_16.PIS28716
Leucothrix_mucor.WP_022953983
Spirochaetaceae_bacterium_4572_59.OQY35497
Candidatus_Firestonebacteria_bacterium_RIFOXYD2_FULL_39_29.OGF46395
Elusimicrobia_bacterium_CG1_02_37_114.OIO74289
Elusimicrobia_bacterium_CG08_land_8_20_14_0_20_59_10.PIU19657
Elusimicrobia_bacterium_GWF2_62_30.OGR94177
bacterium_BMS3Abin03.GBD88315
Deltaproteobacteria_bacterium_HGW_Deltaproteobacteria_19.PKN33266
Desulfobacteraceae_bacterium_A6.OQW99834
Deltaproteobacteria_bacterium_RBG_16_49_23.OGP76114
Syntrophus_sp_RIFOXYC2_FULL_54_9.OHE25895
Smithella_sp_SCADC.KFO66923
Deltaproteobacteria_bacterium_RBG_16_44_11.OGP66844
Deltaproteobacteria_bacterium_RBG_19FT_COMBO_43_11.OGP88340
Bdellovibrionales_bacterium_RIFOXYD1_FULL_53_11.OFZ82544
Deltaproteobacteria_bacterium_GWA2_43_19.OGP12198
Nitrospinae_bacterium_RIFCSPHIGHO2_12_FULL_39_42.OGV98301
Nitrospinae_bacterium_RIFCSPLOWO2_02_FULL_39_110.OGW05939
Deltaproteobacteria_bacterium_GWB2_42_7.OGP22877
Deltaproteobacteria_bacterium_RIFOXYA2_FULL_42_10.OGQ75128
uncultured_bacterium.EKD26242
Candidatus_Aminicenantes_bacterium_RBG_19FT_COMBO_58_17.OGD35346
Candidatus_Aminicenantes_bacterium_RBG_13_62_12.OGD11581
Desulfobacterales_bacterium_C00003060.OEU81907
Deltaproteobacteria_bacterium_SG8_13.KPJ75571
Bdellovibrionales_bacterium_RIFOXYC1_FULL_37_79.OFZ47380
uncultured_bacterium.CAX83822
Alphaproteobacteria_bacterium_41_28.OJW52790
Bdellovibrionales_bacterium_RIFOXYC1_FULL_37_79.OFZ48281
Bdellovibrionales_bacterium_RIFOXYB1_FULL_39_21.OFZ36599
Gammaproteobacteria_bacterium_RIFCSPHIGHO2_12_38_15.OGT23808
Gammaproteobacteria_bacterium_RIFCSPHIGHO2_12_FULL_45_9.OGT68952
Gammaproteobacteria_bacterium_RIFCSPHIGHO2_12_FULL_38_14.OGT36811
Gammaproteobacteria_bacterium_GWE2_37_16.OGT07585
uncultured_bacterium.EKD71815
uncultured_bacterium.EKD56420
CBU_1786
Waddlia_chondrophila.WP_041941606
Waddlia_chondrophila_WSU_86_1044.ADI39113
Gammaproteobacteria_bacterium_RIFCSPHIGHO2_12_FULL_37_34.OGT41287
Gammaproteobacteria_bacterium_RIFCSPHIGHO2_12_FULL_42_10.OGT56656
Coxiella_sp_RIFCSPHIGHO2_12_FULL_44_14.OGO95524
Coxiella_sp_RIFCSPHIGHO2_12_FULL_42_15.OGO91697
Gammaproteobacteria_bacterium_RIFCSPHIGHO2_02_FULL_42_43.OGT26329
uncultured_bacterium.EKD45221
Candidatus_Melainabacteria_bacterium_HGW_Melainabacteria_1.PKL76428
Bdellovibrionales_bacterium_RIFOXYD1_FULL_44_7.OFZ85239
Kangiella_sp.PHS15863
Pseudohongiella_spirulinae.WP_058023090
Pseudohongiella_spirulinae.ALO45076
Candidatus_Magnetoglobus_multicellularis_str_Araruama.ETR69809
delta_proteobacterium_ML8_D.OPL11277
Syntrophobacterales_bacterium_CG_4_8_14_3_um_filter_58_8.PJC71571
Smithella_sp_ME_1.ESP62483
Deltaproteobacteria_bacterium_HGW_Deltaproteobacteria_9.PKN05474
delta_proteobacterium_MLMS_1.EAT01490
delta_proteobacterium_MLMS_1.WP_040871557
delta_proteobacterium_MLMS_1.WP_007291546
delta_proteobacterium_MLMS_1.WP_007292517
Deltaproteobacteria_bacterium_RIFOXYA12_FULL_58_15.OGQ78619
uncultured_Desulfofustis_sp_PB_SRB1.ESQ12598
Deltaproteobacteria_bacterium.PIE69443
Desulfobacter_postgatei.WP_004073943
Desulfotignum_balticum.WP_084489881
Desulfotignum_phosphitoxidans.WP_006968783
uncultured_bacterium
Spirochaetaceae_bacterium_4572_59
Bacteria
Waddlia
Verrucomicrobia_bacterium_RIFCSPHIGHO2_12_FULL_41_10
Waddlia_chondrophila_WSU_86-1044
Bacteria
Candidatus_Saganbacteria_bacterium_CG08_land_8_20_14_0_20_45_16
Candidatus_Melainabacteria_bacterium_HGW-Melainabacteria-1
Nitrospinae
Nitrospinae_bacterium_RIFCSPHIGHO2_12_FULL_39_42
Nitrospinae_bacterium_RIFCSPLOWO2_02_FULL_39_110
Elusimicrobia
Elusimicrobia_bacterium_GWF2_62_30
Elusimicrobia_bacterium_CG1_02_37_114
Elusimicrobia_bacterium_CG08_land_8_20_14_0_20_59_10
Bacteria
bacterium_BMS3Abin03
Bacteria
Candidatus_Firestonebacteria_bacterium_RIFOXYC2_FULL_39_67
Candidatus_Handelsmanbacteria_bacterium_RIFCSPLOWO2_12_FULL_64_10
Candidatus_Aminicenantes
Candidatus_Aminicenantes_bacterium_RBG_13_62_12
Candidatus_Aminicenantes_bacterium_RBG_19FT_COMBO_58_17
Alphaproteobacteria_bacterium_41-28
Bdellovibrionales
Bdellovibrionales_bacterium_RIFOXYB1_FULL_37_110
Bdellovibrionales_bacterium_RIFOXYC1_FULL_37_79
Bdellovibrionales_bacterium_RIFOXYC1_FULL_39_130
Bdellovibrionales_bacterium_RIFOXYD1_FULL_44_7
Bdellovibrionales_bacterium_RIFOXYD1_FULL_53_11
Leucothrix_mucor
Kangiella_sp
Gammaproteobacteria
Coxiella
Coxiella_burnetii_RSA_493
Coxiella
Proteobacteria
Coxiella_sp_RIFCSPHIGHO2_12_FULL_42_15
Coxiella_sp_RIFCSPHIGHO2_12_FULL_44_14
Pseudohongiella_spirulinae
Gammaproteobacteria
Gammaproteobacteria_bacterium_RIFCSPHIGHO2_12_38_15
Gammaproteobacteria_bacterium_RIFCSPHIGHO2_02_FULL_42_43
Gammaproteobacteria_bacterium_RIFCSPHIGHO2_12_FULL_42_10
Gammaproteobacteria
Gammaproteobacteria_bacterium_RIFCSPHIGHO2_12_FULL_37_34
Gammaproteobacteria_bacterium_RIFCSPHIGHO2_12_FULL_38_14
Gammaproteobacteria_bacterium_RIFCSPHIGHO2_12_FULL_45_9
Gammaproteobacteria_bacterium_GWE2_37_16
Gammaproteobacteria_bacterium_RIFCSPHIGHO2_01_FULL_42_8
Syntrophobacterales
Syntrophobacterales_bacterium_CG_4_8_14_3_um_filter_58_8
Syntrophaceae
Syntrophus_sp_RIFOXYC2_FULL_54_9
Smithella
Smithella_sp_ME-1
Smithella_sp_SCADC
Desulfobacterales
Desulfobacterales_bacterium_C00003060
uncultured_Desulfofustis_sp_PB-SRB1
Desulfobacter_postgatei
Desulfobacteraceae
Desulfobacteraceae
Candidatus_Magnetoglobus_multicellularis_str_Araruama
Deltaproteobacteria
Desulfobacteraceae_bacterium_4572_130
Desulfobacteraceae_bacterium_A6
Desulfotignum
Desulfotignum_phosphitoxidans
Desulfotignum_balticum
Deltaproteobacteria_bacterium_RIFOXYA12_FULL_58_15
Deltaproteobacteria_bacterium_RIFOXYA2_FULL_42_10
Deltaproteobacteria_bacterium_RBG_19FT_COMBO_43_11
Deltaproteobacteria_bacterium_GWA2_43_19
Deltaproteobacteria_bacterium_HGW-Deltaproteobacteria-19
Deltaproteobacteria_bacterium_GWB2_42_7
delta_proteobacterium_ML8_D
Deltaproteobacteria
Deltaproteobacteria_bacterium_RBG_16_44_11
Deltaproteobacteria_bacterium_RBG_16_49_23
Deltaproteobacteria_bacterium_RIFCSPHIGHO2_02_FULL_40_28
delta_proteobacterium_MLMS-1
Deltaproteobacteria_bacterium_RIFCSPLOWO2_02_FULL_46_8
Deltaproteobacteria_bacterium_SG8_13
Deltaproteobacteria_bacterium
Deltaproteobacteria_bacterium_HGW-Deltaproteobacteria-9

CBU_1638

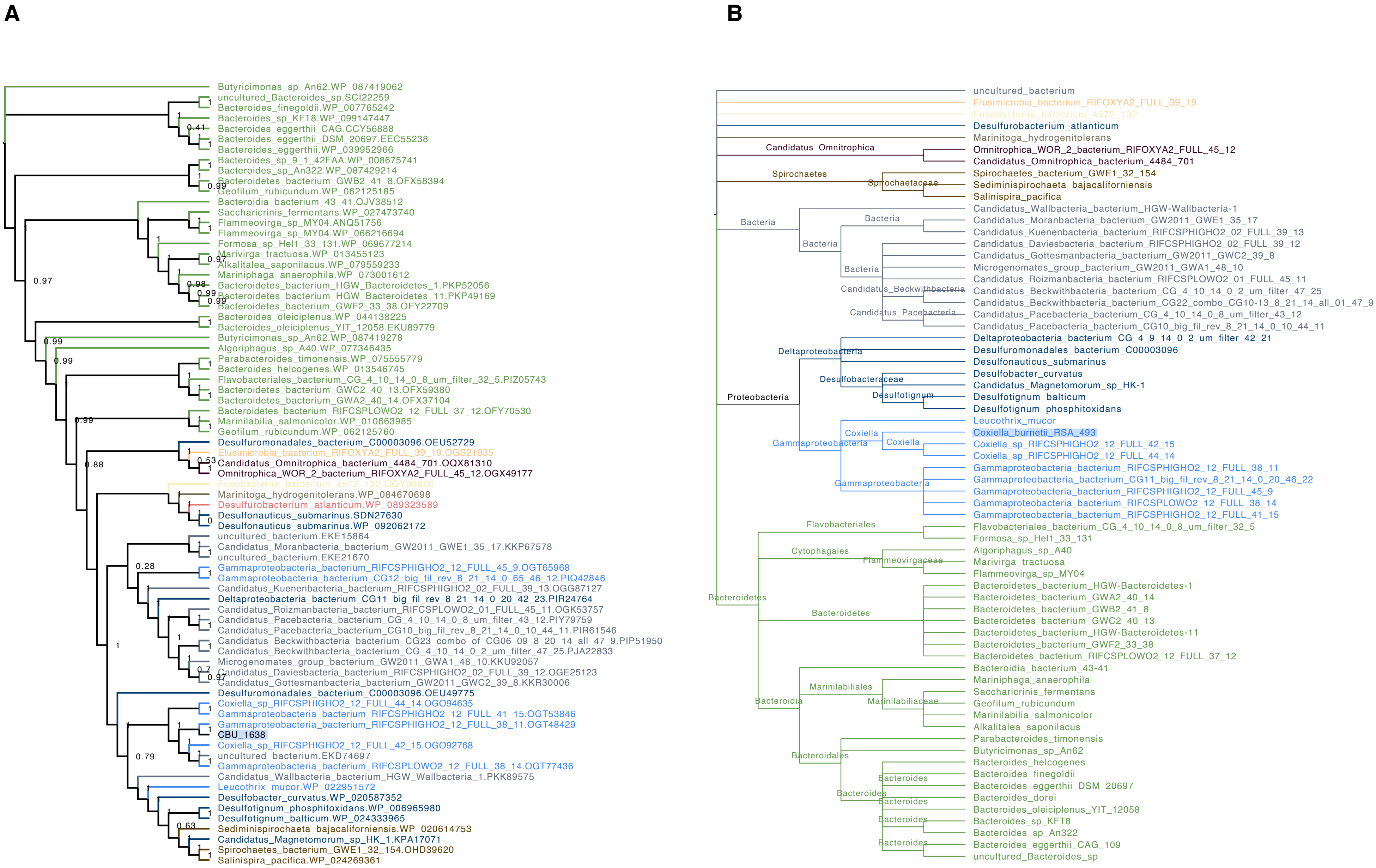
A
Butyricimonas_sp_An62.WP_087419062
uncultured_Bacteroides_sp.SCI22259
Bacteroides_finegoldii.WP_007765242
Bacteroides_sp_KFT8.WP_099147447
Bacteroides_eggerthii_CAG.CCY56888
Bacteroides_eggerthii_DSM_20697.EEC55238
Bacteroides_eggerthii.WP_039952966
Bacteroides_sp_9_1_42FAA.WP_008675741
Bacteroides_sp_An322.WP_087429214
Bacteroidetes_bacterium_GWB2_41_8.OFX58394
Geofilum_rubicundum.WP_062125185
Bacteroidia_bacterium_43_41.OJV38512
Saccharicrinis_fermentans.WP_027473740
Flammeovirga_sp_MY04.ANQ51756
Flammeovirga_sp_MY04.WP_066216694
Formosa_sp_Hel1_33_131.WP_069677214
Marivirga_tractuosa.WP_013455123
Alkalitalea_saponilacus.WP_079559233
Mariniphaga_anaerophila.WP_073001612
Bacteroidetes_bacterium_HGW_Bacteroidetes_1.PKP52056
Bacteroidetes_bacterium_HGW_Bacteroidetes_11.PKP49169
Bacteroidetes_bacterium_GWF2_33_38.OFY22709
Bacteroides_oleiciplenus.WP_044138225
Bacteroides_oleiciplenus_YIT_12058.EKU89779
Butyricimonas_sp_An62.WP_087419278
Algoriphagus_sp_A40.WP_077346435
Parabacteroides_timonensis.WP_075555779
Bacteroides_helcogenes.WP_013546745
Flavobacteriales_bacterium_CG_4_10_14_0_8_um_filter_32_5.PIZ05743
Bacteroidetes_bacterium_GWC2_40_13.OFX59380
Bacteroidetes_bacterium_GWA2_40_14.OFX37104
Bacteroidetes_bacterium_RIFCSPLOWO2_12_FULL_37_12.OFY70530
Marinilabilia_salmonicolor.WP_010663985
Geofilum_rubicundum.WP_062125760
Desulfuromonadales_bacterium_C00003096.OEU52729
Elusimicrobia_bacterium_RIFOXYA2_FULL_39_19.OGS21935
Candidatus_Omnitrophica_bacterium_4484_701.OQX81310
Omnitrophica_WOR_2_bacterium_RIFOXYA2_FULL_45_12.OGX49177
Fusobacteriia_bacterium_4572_132.OQY08680
Marinitoga_hydrogenitolerans.WP_084670698
Desulfurobacterium_atlanticum.WP_089323589
Desulfonauticus_submarinus.SDN27630
Desulfonauticus_submarinus.WP_092062172
uncultured_bacterium.EKE15864
Candidatus_Moranbacteria_bacterium_GW2011_GWE1_35_17.KKP67578
uncultured_bacterium.EKE21670
Gammaproteobacteria_bacterium_RIFCSPHIGHO2_12_FULL_45_9.OGT65968
Gammaproteobacteria_bacterium_CG12_big_fil_rev_8_21_14_0_65_46_12.PIQ42846
Candidatus_Kuenenbacteria_bacterium_RIFCSPHIGHO2_02_FULL_39_13.OGG87127
Deltaproteobacteria_bacterium_CG11_big_fil_rev_8_21_14_0_20_42_23.PIR24764
Candidatus_Roizmanbacteria_bacterium_RIFCSPLOWO2_01_FULL_45_11.OGK53757
Candidatus_Pacebacteria_bacterium_CG_4_10_14_0_8_um_filter_43_12.PIY79759
Candidatus_Pacebacteria_bacterium_CG10_big_fil_rev_8_21_14_0_10_44_11.PIR61546
Candidatus_Beckwithbacteria_bacterium_CG23_combo_of_CG06_09_8_20_14_all_47_9.PIP51950
Candidatus_Beckwithbacteria_bacterium_CG_4_10_14_0_2_um_filter_47_25.PJA22833
Microgenomates_group_bacterium_GW2011_GWA1_48_10.KKU92057
Candidatus_Daviesbacteria_bacterium_RIFCSPHIGHO2_02_FULL_39_12.OGE25123
Candidatus_Gottesmanbacteria_bacterium_GW2011_GWC2_39_8.KKR30006
Desulfuromonadales_bacterium_C00003096.OEU49775
Coxiella_sp_RIFCSPHIGHO2_12_FULL_44_14.OGO94635
Gammaproteobacteria_bacterium_RIFCSPHIGHO2_12_FULL_41_15.OGT53846
Gammaproteobacteria_bacterium_RIFCSPHIGHO2_12_FULL_38_11.OGT48429
CBU_1638
Coxiella_sp_RIFCSPHIGHO2_12_FULL_42_15.OGO92768
uncultured_bacterium.EKD74697
Gammaproteobacteria_bacterium_RIFCSPLOWO2_12_FULL_38_14.OGT77436
Candidatus_Wallbacteria_bacterium_HGW_Wallbacteria_1.PKK89575
Leucothrix_mucor.WP_022951572
Desulfobacter_curvatus.WP_020587352
Desulfotignum_phosphitoxidans.WP_006965980
Desulfotignum_balticum.WP_024333965
Sediminispirochaeta_bajacaliforniensis.WP_020614753
Candidatus_Magnetomorum_sp_HK_1.KPA17071
Spirochaetes_bacterium_GWE1_32_154.OHD39620
Salinispira_pacifica.WP_024269361
B
uncultured_bacterium
Elusimicrobia_bacterium_RIFOXYA2_FULL_39_19
Fusobacteriia_bacterium_4572_132
Desulfurobacterium_atlanticum
Marinitoga_hydrogenitolerans
Candidatus_Omnitrophica
Omnitrophica_WOR_2_bacterium_RIFOXYA2_FULL_45_12
Candidatus_Omnitrophica_bacterium_4484_701
Spirochaetes
Spirochaetaceae
Spirochaetes_bacterium_GWE1_32_154
Sediminispirochaeta_bajacaliforniensis
Salinispira_pacifica
Bacteria
Candidatus_Wallbacteria_bacterium_HGW-Wallbacteria-1
Candidatus_Moranbacteria_bacterium_GW2011_GWE1_35_17
Candidatus_Kuenenbacteria_bacterium_RIFCSPHIGHO2_02_FULL_39_13
Candidatus_Daviesbacteria_bacterium_RIFCSPHIGHO2_02_FULL_39_12
Candidatus_Gottesmanbacteria_bacterium_GW2011_GWC2_39_8
Microgenomates_group_bacterium_GW2011_GWA1_48_10
Candidatus_Roizmanbacteria_bacterium_RIFCSPLOWO2_01_FULL_45_11
Candidatus_Beckwithbacteria
Candidatus_Beckwithbacteria_bacterium_CG_4_10_14_0_2_um_filter_47_25
Candidatus_Beckwithbacteria_bacterium_CG22_combo_CG10-13_8_21_14_all_01_47_9
Candidatus_Pacebacteria
Candidatus_Pacebacteria_bacterium_CG_4_10_14_0_8_um_filter_43_12
Candidatus_Pacebacteria_bacterium_CG10_big_fil_rev_8_21_14_0_10_44_11
Proteobacteria
Deltaproteobacteria
Deltaproteobacteria_bacterium_CG_4_9_14_0_2_um_filter_42_21
Desulfuromonadales_bacterium_C00003096
Desulfonauticus_submarinus
Desulfobacteraceae
Desulfobacter_curvatus
Candidatus_Magnetomorum_sp_HK-1
Desulfotignum
Desulfotignum_balticum
Desulfotignum_phosphitoxidans
Gammaproteobacteria
Leucothrix_mucor
Coxiella
Coxiella_burnetii_RSA_493
Coxiella_sp_RIFCSPHIGHO2_12_FULL_42_15
Coxiella_sp_RIFCSPHIGHO2_12_FULL_44_14
Gammaproteobacteria_bacterium_RIFCSPHIGHO2_12_FULL_38_11
Gammaproteobacteria_bacterium_CG11_big_fil_rev_8_21_14_0_20_46_22
Gammaproteobacteria_bacterium_RIFCSPHIGHO2_12_FULL_45_9
Gammaproteobacteria_bacterium_RIFCSPLOWO2_12_FULL_38_14
Gammaproteobacteria_bacterium_RIFCSPHIGHO2_12_FULL_41_15
Bacteroidetes
Flavobacteriales
Flavobacteriales_bacterium_CG_4_10_14_0_8_um_filter_32_5
Formosa_sp_Hel1_33_131
Cytophagales
Flammeovirgaceae
Algoriphagus_sp_A40
Marivirga_tractuosa
Flammeovirga_sp_MY04
Bacteroidetes_bacterium_HGW-Bacteroidetes-1
Bacteroidetes_bacterium_GWA2_40_14
Bacteroidetes_bacterium_GWB2_41_8
Bacteroidetes_bacterium_GWC2_40_13
Bacteroidetes_bacterium_HGW-Bacteroidetes-11
Bacteroidetes_bacterium_GWF2_33_38
Bacteroidetes_bacterium_RIFCSPLOWO2_12_FULL_37_12
Bacteroidia
Marinilabiliales
Marinilabiliaceae
Bacteroidia_bacterium_43-41
Mariniphaga_anaerophila
Saccharicrinis_fermentans
Geofilum_rubicundum
Marinilabilia_salmonicolor
Alkalitalea_saponilacus
Bacteroidales
Bacteroides
Parabacteroides_timonensis
Butyricimonas_sp_An62
Bacteroides_helcogenes
Bacteroides_finegoldii
Bacteroides_eggerthii_DSM_20697
Bacteroides_dorei
Bacteroides_oleiciplenus_YIT_12058
Bacteroides_sp_KFT8
Bacteroides_sp_An322
Bacteroides_eggerthii_CAG_109
uncultured_Bacteroides_sp

CBU_1515

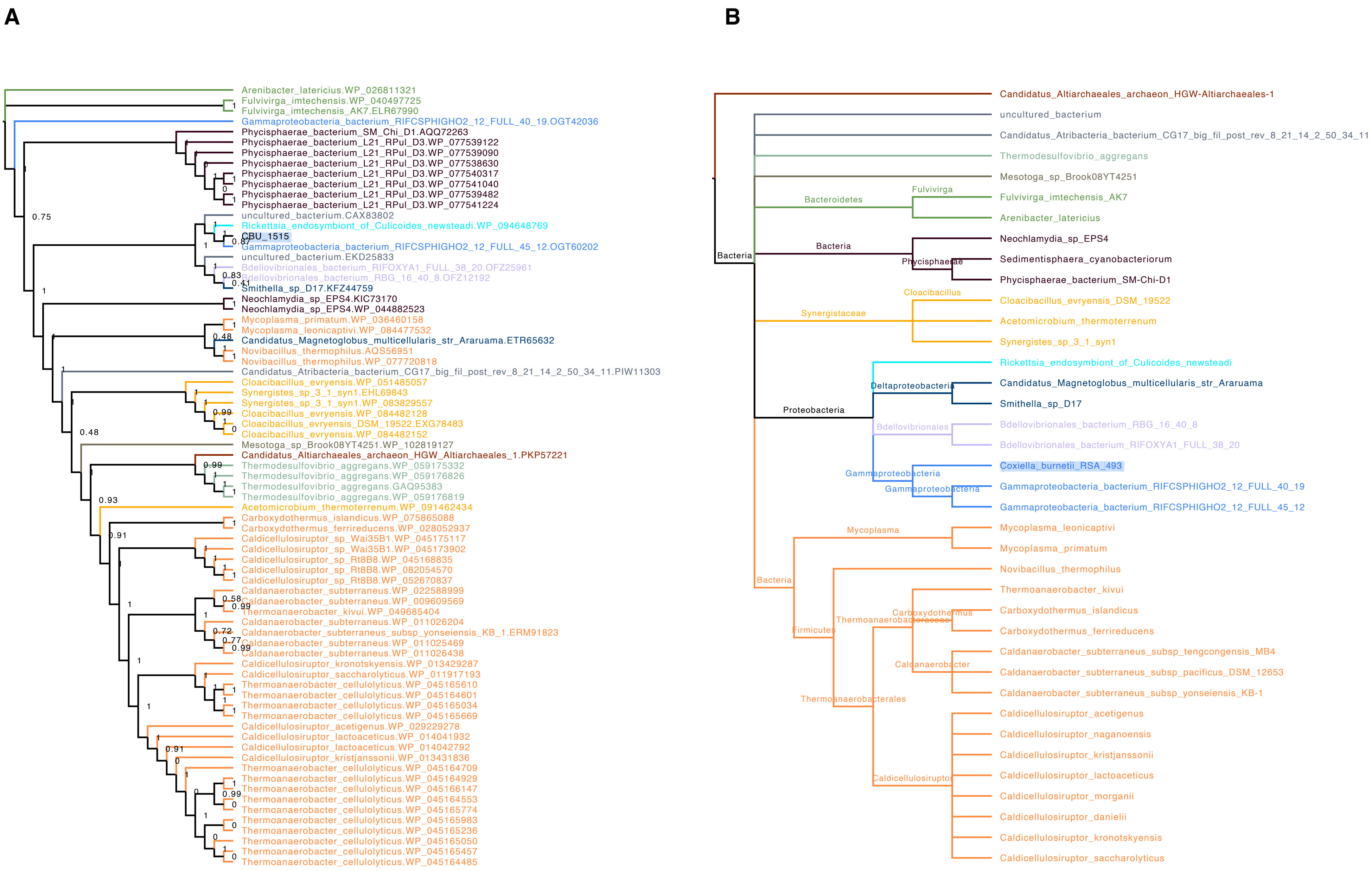
A
B
Arenibacter_latericius.WP_026811321
Fulvivirga_imtechensis.WP_040497725
Fulvivirga_imtechensis_AK7.ELR67990
Gammaproteobacteria_bacterium_RIFCSPHIGHO2_12_FULL_40_19.OGT42036
Phycisphaerae_bacterium_SM_Chi_D1.AQQ72263
Phycisphaerae_bacterium_L21_RPul_D3.WP_077539122
Phycisphaerae_bacterium_L21_RPul_D3.WP_077539090
Phycisphaerae_bacterium_L21_RPul_D3.WP_077538630
Phycisphaerae_bacterium_L21_RPul_D3.WP_077540317
Phycisphaerae_bacterium_L21_RPul_D3.WP_077541040
Phycisphaerae_bacterium_L21_RPul_D3.WP_077539482
Phycisphaerae_bacterium_L21_RPul_D3.WP_077541224
uncultured_bacterium.CAX83802
Rickettsia_endosymbiont_of_Culicoides_newsteadi.WP_094648769
CBU_1515
Gammaproteobacteria_bacterium_RIFCSPHIGHO2_12_FULL_45_12.OGT60202
uncultured_bacterium.EKD25833
Bdellovibrionales_bacterium_RIFOXYA1_FULL_38_20.OFZ25961
Bdellovibrionales_bacterium_RBG_16_40_8.OFZ12192
Smithella_sp_D17.KFZ44759
Neochlamydia_sp_EPS4.KIC73170
Neochlamydia_sp_EPS4.WP_044882523
Mycoplasma_primatum.WP_036460158
Mycoplasma_leonicaptivi.WP_084477532
Candidatus_Magnetoglobus_multicellularis_str_Araruama.ETR65632
Novibacillus_thermophilus.AQS56951
Novibacillus_thermophilus.WP_077720818
Candidatus_Atribacteria_bacterium_CG17_big_fil_post_rev_8_21_14_2_50_34_11.PIW11303
Cloacibacillus_evryensis.WP_051485057
Synergistes_sp_3_1_syn1.EHL69843
Synergistes_sp_3_1_syn1.WP_083829557
Cloacibacillus_evryensis.WP_084482128
Cloacibacillus_evryensis_DSM_19522.EXG78483
Cloacibacillus_evryensis.WP_084482152
Mesotoga_sp_Brook08YT4251.WP_102819127
Candidatus_Altiarchaeales_archaeon_HGW_Altiarchaeales_1.PKP57221
Thermodesulfovibrio_aggregans.WP_059175332
Thermodesulfovibrio_aggregans.WP_059176826
Thermodesulfovibrio_aggregans.GAQ95383
Thermodesulfovibrio_aggregans.WP_059176819
Acetomicrobium_thermoterrenum.WP_091462434
Carboxydothermus_islandicus.WP_075865088
Carboxydothermus_ferrireducens.WP_028052937
Caldicellulosiruptor_sp_Wai35B1.WP_045175117
Caldicellulosiruptor_sp_Wai35B1.WP_045173902
Caldicellulosiruptor_sp_Rt8B8.WP_045168835
Caldicellulosiruptor_sp_Rt8B8.WP_082054570
Caldicellulosiruptor_sp_Rt8B8.WP_052670837
Caldanaerobacter_subterraneus.WP_022588999
Caldanaerobacter_subterraneus.WP_009609569
Thermoanaerobacter_kivui.WP_049685404
Caldanaerobacter_subterraneus.WP_011026204
Caldanaerobacter_subterraneus_subsp_yonseiensis_KB_1.ERM91823
Caldanaerobacter_subterraneus.WP_011025469
Caldanaerobacter_subterraneus.WP_011026438
Caldicellulosiruptor_kronotskyensis.WP_013429287
Caldicellulosiruptor_saccharolyticus.WP_011917193
Thermoanaerobacter_cellulolyticus.WP_045165610
Thermoanaerobacter_cellulolyticus.WP_045164601
Thermoanaerobacter_cellulolyticus.WP_045165034
Thermoanaerobacter_cellulolyticus.WP_045165669
Caldicellulosiruptor_acetigenus.WP_029229278
Caldicellulosiruptor_lactoaceticus.WP_014041932
Caldicellulosiruptor_lactoaceticus.WP_014042792
Caldicellulosiruptor_kristjanssonii.WP_013431836
Thermoanaerobacter_cellulolyticus.WP_045164709
Thermoanaerobacter_cellulolyticus.WP_045164929
Thermoanaerobacter_cellulolyticus.WP_045166147
Thermoanaerobacter_cellulolyticus.WP_045164553
Thermoanaerobacter_cellulolyticus.WP_045165774
Thermoanaerobacter_cellulolyticus.WP_045165983
Thermoanaerobacter_cellulolyticus.WP_045165236
Thermoanaerobacter_cellulolyticus.WP_045165050
Thermoanaerobacter_cellulolyticus.WP_045165457
Thermoanaerobacter_cellulolyticus.WP_045164485
Candidatus_Altiarchaeales_archaeon_HGW-Altiarchaeales-1
uncultured_bacterium
Candidatus_Atribacteria_bacterium_CG17_big_fil_post_rev_8_21_14_2_50_34_11
Thermodesulfovibrio_aggregans
Mesotoga_sp_Brook08YT4251
Bacteroidetes
Fulvivirga
Fulvivirga_imtechensis_AK7
Arenibacter_latericius
Bacteria
Neochlamydia_sp_EPS4
Phycisphaerae
Sedimentisphaera_cyanobacteriorum
Phycisphaerae_bacterium_SM-Chi-D1
Cloacibacillus
Synergistaceae
Cloacibacillus_evryensis_DSM_19522
Acetomicrobium_thermoterrenum
Synergistes_sp_3_1_syn1
Rickettsia_endosymbiont_of_Culicoides_newsteadi
Deltaproteobacteria
Candidatus_Magnetoglobus_multicellularis_str_Araruama
Proteobacteria
Smithella_sp_D17
Bdellovibrionales
Bdellovibrionales_bacterium_RBG_16_40_8
Bdellovibrionales_bacterium_RIFOXYA1_FULL_38_20
Gammaproteobacteria
Coxiella_burnetii_RSA_493
Gammaproteobacteria_bacterium_RIFCSPHIGHO2_12_FULL_40_19
Gammaproteobacteria_bacterium_RIFCSPHIGHO2_12_FULL_45_12
Mycoplasma
Mycoplasma_leonicaptivi
Mycoplasma_primatum
Novibacillus_thermophilus
Thermoanaerobacter_kivui
Carboxydothermus
Carboxydothermus_islandicus
Thermoanaerobacteraceae
Firmicutes
Carboxydothermus_ferrireducens
Caldanaerobacter_subterraneus_subsp_tengcongensis_MB4
Caldanaerobacter
Caldanaerobacter_subterraneus_subsp_pacificus_DSM_12653
Caldanaerobacter_subterraneus_subsp_yonseiensis_KB-1
Thermoanaerobacterales
Caldicellulosiruptor_acetigenus
Caldicellulosiruptor_naganoensis
Caldicellulosiruptor_kristjanssonii
Caldicellulosiruptor
Caldicellulosiruptor_lactoaceticus
Caldicellulosiruptor_morganii
Caldicellulosiruptor_danielii
Caldicellulosiruptor_kronotskyensis
Caldicellulosiruptor_saccharolyticus

CBU_1508

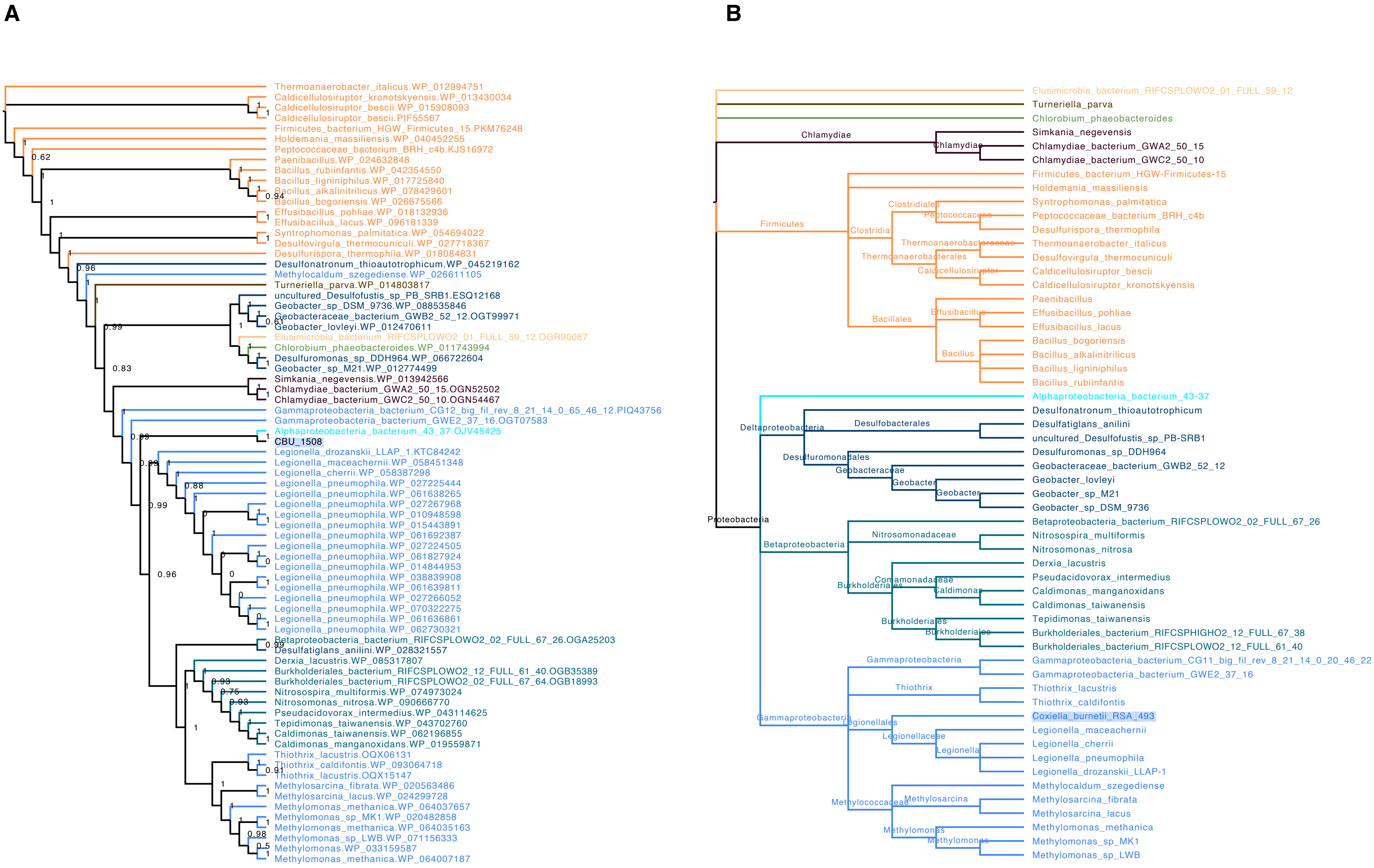

A
Thermoanaerobacter_italicus.WP_012994751
Caldicellulosiruptor_kronotskyensis.WP_013430034
Caldicellulosiruptor_bescii.WP_015908093
Caldicellulosiruptor_bescii.PIF55567
Firmicutes_bacterium_HGW_Firmicutes_15.PKM76248
Holdemania_massiliensis.WP_040452255
Peptococcaceae_bacterium_BRH_c4b.KJS16972
Paenibacillus.WP_024632848
Bacillus_rubiinfantis.WP_042354550
Bacillus_ligniniphilus.WP_017725840
Bacillus_alkalinitrilicus.WP_078429601
Bacillus_bogoriensis.WP_026675566
Effusibacillus_pohliae.WP_018132936
Effusibacillus_lacus.WP_096181339
Syntrophomonas_palmitatica.WP_054694022
Desulfovirgula_thermocuniculi.WP_027718367
Desulfurispora_thermophila.WP_018084831
Desulfonatronum_thioautotrophicum.WP_045219162
Methylocaldum_szegediense.WP_026611105
Turneriella_parva.WP_014803817
uncultured_Desulfofustis_sp_PB_SRB1.ESQ12168
Geobacter_sp_DSM_9736.WP_088535846
Geobacteraceae_bacterium_GWB2_52_12.OGT99971
Geobacter_lovleyi.WP_012470611
Elusimicrobia_bacterium_RIFCSPLOWO2_01_FULL_59_12.OGR90087
Chlorobium_phaeobacteroides.WP_011743994
Desulfuromonas_sp_DDH964.WP_066722604
Geobacter_sp_M21.WP_012774499
Simkania_negevensis.WP_013942566
Chlamydiae_bacterium_GWA2_50_15.OGN52502
Chlamydiae_bacterium_GWC2_50_10.OGN54467
Gammaproteobacteria_bacterium_CG12_big_fil_rev_8_21_14_0_65_46_12.PIQ43756
Gammaproteobacteria_bacterium_GWE2_37_16.OGT07583
Alphaproteobacteria_bacterium_43_37.OJV45425
CBU_1508
Legionella_drozanskii_LLAP_1.KTC84242
Legionella_maceachernii.WP_058451348
Legionella_cherrii.WP_058387298
Legionella_pneumophila.WP_027225444
Legionella_pneumophila.WP_061638265
Legionella_pneumophila.WP_027267968
Legionella_pneumophila.WP_010948598
Legionella_pneumophila.WP_015443891
Legionella_pneumophila.WP_061692387
Legionella_pneumophila.WP_027224505
Legionella_pneumophila.WP_061827924
Legionella_pneumophila.WP_014844953
Legionella_pneumophila.WP_038839908
Legionella_pneumophila.WP_061639811
Legionella_pneumophila.WP_027266052
Legionella_pneumophila.WP_070322275
Legionella_pneumophila.WP_061636861
Legionella_pneumophila.WP_062730321
Betaproteobacteria_bacterium_RIFCSPLOWO2_02_FULL_67_26.OGA25203
Desulfatiglans_anilini.WP_028321557
Derxia_lacustris.WP_085317807
Burkholderiales_bacterium_RIFCSPLOWO2_12_FULL_61_40.OGB35389
Burkholderiales_bacterium_RIFCSPLOWO2_02_FULL_67_64.OGB18993
Nitrosospira_multiformis.WP_074973024
Nitrosomonas_nitrosa.WP_090666770
Pseudacidovorax_intermedius.WP_043114625
Tepidimonas_taiwanensis.WP_043702760
Caldimonas_taiwanensis.WP_062196855
Caldimonas_manganoxidans.WP_019559871
Thiothrix_lacustris.OQX06131
Thiothrix_caldifontis.WP_093064718
Thiothrix_lacustris.OQX15147
Methylosarcina_fibrata.WP_020563486
Methylosarcina_lacus.WP_024299728
Methylomonas_methanica.WP_064037657
Methylomonas_sp_MK1.WP_020482858
Methylomonas_methanica.WP_064035163
Methylomonas_sp_LWB.WP_071156333
Methylomonas.WP_033159587
Methylomonas_methanica.WP_064007187
B
Elusimicrobia_bacterium_RIFCSPLOWO2_01_FULL_59_12
Turneriella_parva
Chlorobium_phaeobacteroides
Chlamydiae
Simkania_negevensis
Chlamydiae_bacterium_GWA2_50_15
Chlamydiae_bacterium_GWC2_50_10
Firmicutes
Clostridia
Clostridiales
Peptococcaceae
Thermoanaerobacterales
Thermoanaerobacteraceae
Caldicellulosiruptor
Bacillales
Effusibacillus
Bacillus
Firmicutes_bacterium_HGW-Firmicutes-15
Holdemania_massiliensis
Syntrophomonas_palmitatica
Peptococcaceae_bacterium_BRH_c4b
Desulfurispora_thermophila
Thermoanaerobacter_italicus
Desulfovirgula_thermocuniculi
Caldicellulosiruptor_bescii
Caldicellulosiruptor_kronotskyensis
Paenibacillus
Effusibacillus_pohliae
Effusibacillus_lacus
Bacillus_bogoriensis
Bacillus_alkalinitrilicus
Bacillus_ligniniphilus
Bacillus_rubiinfantis
Proteobacteria
Deltaproteobacteria
Desulfobacterales
Desulfuromonadales
Geobacteraceae
Geobacter
Alphaproteobacteria_bacterium_43-37
Desulfonatronum_thioautotrophicum
Desulfatiglans_anilini
uncultured_Desulfofustis_sp_PB-SRB1
Desulfuromonas_sp_DDH964
Geobacteraceae_bacterium_GWB2_52_12
Geobacter_lovleyi
Geobacter_sp_M21
Geobacter_sp_DSM_9736
Betaproteobacteria
Nitrosomonadaceae
Burkholderiales
Comamonadaceae
Caldimonas
Betaproteobacteria_bacterium_RIFCSPLOWO2_02_FULL_67_26
Nitrosospira_multiformis
Nitrosomonas_nitrosa
Derxia_lacustris
Pseudacidovorax_intermedius
Caldimonas_manganoxidans
Caldimonas_taiwanensis
Tepidimonas_taiwanensis
Burkholderiales_bacterium_RIFCSPHIGHO2_12_FULL_67_38
Burkholderiales_bacterium_RIFCSPLOWO2_12_FULL_61_40
Gammaproteobacteria
Thiothrix
Legionellales
Legionellaceae
Legionella
Methylococcaceae
Methylosarcina
Methylomonas
Gammaproteobacteria_bacterium_CG11_big_fil_rev_8_21_14_0_20_46_22
Gammaproteobacteria_bacterium_GWE2_37_16
Thiothrix_lacustris
Thiothrix_caldifontis
Coxiella_burnetii_RSA_493
Legionella_maceachernii
Legionella_cherrii
Legionella_pneumophila
Legionella_drozanskii_LLAP-1
Methylocaldum_szegediense
Methylosarcina_fibrata
Methylosarcina_lacus
Methylomonas_methanica
Methylomonas_sp_MK1
Methylomonas_sp_LWB

CBU_1491

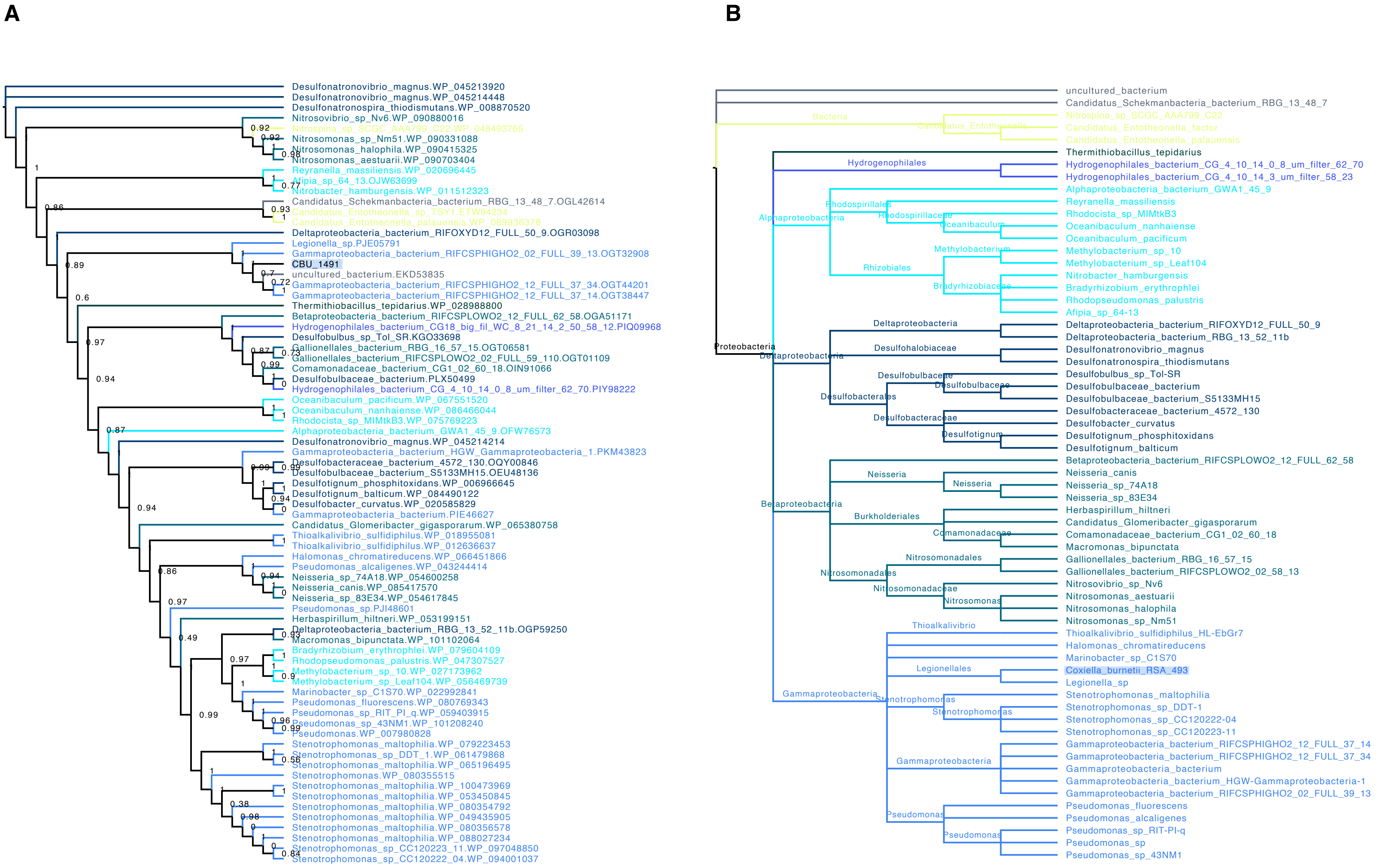

A
Desulfonatronovibrio_magnus.WP_045213920
Desulfonatronovibrio_magnus.WP_045214448
Desulfonatronospira_thiodismutans.WP_008870520
Nitrosovibrio_sp_Nv6.WP_090880016
Nitrospina_sp_SCGC_AAA799_C22.WP_048493765
Nitrosomonas_sp_Nm51.WP_090331088
Nitrosomonas_halophila.WP_090415325
Nitrosomonas_aestuarii.WP_090703404
Reyranella_massiliensis.WP_020696445
Afipia_sp_64_13.OJW63699
Nitrobacter_hamburgensis.WP_011512323
Candidatus_Schekmanbacteria_bacterium_RBG_13_48_7.OGL42614
Candidatus_Entotheonella_sp_TSY1.ETW94234
Candidatus_Entotheonella_palauensis.WP_089936378
Deltaproteobacteria_bacterium_RIFOXYD12_FULL_50_9.OGR03098
Legionella_sp.PJE05791
Gammaproteobacteria_bacterium_RIFCSPHIGHO2_02_FULL_39_13.OGT32908
CBU_1491
uncultured_bacterium.EKD53835
Gammaproteobacteria_bacterium_RIFCSPHIGHO2_12_FULL_37_34.OGT44201
Gammaproteobacteria_bacterium_RIFCSPHIGHO2_12_FULL_37_14.OGT38447
Thermithiobacillus_tepidarius.WP_028988800
Betaproteobacteria_bacterium_RIFCSPLOWO2_12_FULL_62_58.OGA51171
Hydrogenophilales_bacterium_CG18_big_fil_WC_8_21_14_2_50_58_12.PIQ09968
Desulfobulbus_sp_Tol_SR.KGO33698
Gallionellales_bacterium_RBG_16_57_15.OGT06581
Gallionellales_bacterium_RIFCSPLOWO2_02_FULL_59_110.OGT01109
Comamonadaceae_bacterium_CG1_02_60_18.OIN91066
Desulfobulbaceae_bacterium.PLX50499
Hydrogenophilales_bacterium_CG_4_10_14_0_8_um_filter_62_70.PIY98222
Oceanibaculum_pacificum.WP_067551520
Oceanibaculum_nanhaiense.WP_086466044
Rhodocista_sp_MIMtkB3.WP_075769223
Alphaproteobacteria_bacterium_GWA1_45_9.OFW76573
Desulfonatronovibrio_magnus.WP_045214214
Gammaproteobacteria_bacterium_HGW_Gammaproteobacteria_1.PKM43823
Desulfobacteraceae_bacterium_4572_130.OQY00846
Desulfobulbaceae_bacterium_S5133MH15.OEU48136
Desulfotignum_phosphitoxidans.WP_006966645
Desulfotignum_balticum.WP_084490122
Desulfobacter_curvatus.WP_020585829
Gammaproteobacteria_bacterium.PIE46627
Candidatus_Glomeribacter_gigasporarum.WP_065380758
Thioalkalivibrio_sulfidiphilus.WP_018955081
Thioalkalivibrio_sulfidiphilus.WP_012636637
Halomonas_chromatireducens.WP_066451866
Pseudomonas_alcaligenes.WP_043244414
Neisseria_sp_74A18.WP_054600258
Neisseria_canis.WP_085417570
Neisseria_sp_83E34.WP_054617845
Pseudomonas_sp.PJI48601
Herbaspirillum_hiltneri.WP_053199151
Deltaproteobacteria_bacterium_RBG_13_52_11b.OGP59250
Macromonas_bipunctata.WP_101102064
Bradyrhizobium_erythrophlei.WP_079604109
Rhodopseudomonas_palustris.WP_047307527
Methylobacterium_sp_10.WP_027173962
Methylobacterium_sp_Leaf104.WP_056469739
Marinobacter_sp_C1S70.WP_022992841
Pseudomonas_fluorescens.WP_080769343
Pseudomonas_sp_RIT_PI_q.WP_059403915
Pseudomonas_sp_43NM1.WP_101208240
Pseudomonas.WP_007980828
Stenotrophomonas_maltophilia.WP_079223453
Stenotrophomonas_sp_DDT_1.WP_061479868
Stenotrophomonas_maltophilia.WP_065196495
Stenotrophomonas.WP_080355515
Stenotrophomonas_maltophilia.WP_100473969
Stenotrophomonas_maltophilia.WP_053450845
Stenotrophomonas_maltophilia.WP_080354792
Stenotrophomonas_maltophilia.WP_049435905
Stenotrophomonas_maltophilia.WP_080356578
Stenotrophomonas_maltophilia.WP_088027234
Stenotrophomonas_sp_CC120223_11.WP_097048850
Stenotrophomonas_sp_CC120222_04.WP_094001037
B
uncultured_bacterium
Candidatus_Schekmanbacteria_bacterium_RBG_13_48_7
Bacteria
Candidatus_Entotheonella
Nitrospina_sp_SCGC_AAA799_C22
Candidatus_Entotheonella_factor
Candidatus_Entotheonella_palauensis
Thermithiobacillus_tepidarius
Hydrogenophilales
Hydrogenophilales_bacterium_CG_4_10_14_0_8_um_filter_62_70
Hydrogenophilales_bacterium_CG_4_10_14_3_um_filter_58_23
Alphaproteobacteria_bacterium_GWA1_45_9
Rhodospirillales
Reyranella_massiliensis
Alphaproteobacteria
Rhodospirillaceae
Rhodocista_sp_MIMtkB3
Oceanibaculum
Oceanibaculum_nanhaiense
Oceanibaculum_pacificum
Methylobacterium
Methylobacterium_sp_10
Rhizobiales
Methylobacterium_sp_Leaf104
Nitrobacter_hamburgensis
Bradyrhizobiaceae
Bradyrhizobium_erythrophlei
Rhodopseudomonas_palustris
Afipia_sp_64-13
Deltaproteobacteria
Deltaproteobacteria_bacterium_RIFOXYD12_FULL_50_9
Deltaproteobacteria_bacterium_RBG_13_52_11b
Proteobacteria
Desulfohalobiaceae
Desulfonatronovibrio_magnus
Deltaproteobacteria
Desulfonatronospira_thiodismutans
Desulfobulbaceae
Desulfobulbus_sp_Tol-SR
Desulfobulbaceae
Desulfobulbaceae_bacterium
Desulfobacterales
Desulfobulbaceae_bacterium_S5133MH15
Desulfobacteraceae_bacterium_4572_130
Desulfobacteraceae
Desulfobacter_curvatus
Desulfotignum
Desulfotignum_phosphitoxidans
Desulfotignum_balticum
Betaproteobacteria_bacterium_RIFCSPLOWO2_12_FULL_62_58
Neisseria
Neisseria_canis
Neisseria
Neisseria_sp_74A18
Betaproteobacteria
Neisseria_sp_83E34
Herbaspirillum_hiltneri
Burkholderiales
Candidatus_Glomeribacter_gigasporarum
Comamonadaceae
Comamonadaceae_bacterium_CG1_02_60_18
Macromonas_bipunctata
Nitrosomonadales
Gallionellales_bacterium_RBG_16_57_15
Nitrosomonadales
Gallionellales_bacterium_RIFCSPLOWO2_02_58_13
Nitrosovibrio_sp_Nv6
Nitrosomonadaceae
Nitrosomonas_aestuarii
Nitrosomonas
Nitrosomonas_halophila
Nitrosomonas_sp_Nm51
Thioalkalivibrio
Thioalkalivibrio_sulfidiphilus_HL-EbGr7
Halomonas_chromatireducens
Marinobacter_sp_C1S70
Legionellales
Coxiella_burnetii_RSA_493
Legionella_sp
Gammaproteobacteria
Stenotrophomonas
Stenotrophomonas_maltophilia
Stenotrophomonas
Stenotrophomonas_sp_DDT-1
Stenotrophomonas_sp_CC120222-04
Stenotrophomonas_sp_CC120223-11
Gammaproteobacteria_bacterium_RIFCSPHIGHO2_12_FULL_37_14
Gammaproteobacteria
Gammaproteobacteria_bacterium_RIFCSPHIGHO2_12_FULL_37_34
Gammaproteobacteria_bacterium
Gammaproteobacteria_bacterium_HGW-Gammaproteobacteria-1
Gammaproteobacteria_bacterium_RIFCSPHIGHO2_02_FULL_39_13
Pseudomonas_fluorescens
Pseudomonas
Pseudomonas_alcaligenes
Pseudomonas
Pseudomonas_sp_RIT-PI-q
Pseudomonas_sp
Pseudomonas_sp_43NM1

CBU_1485

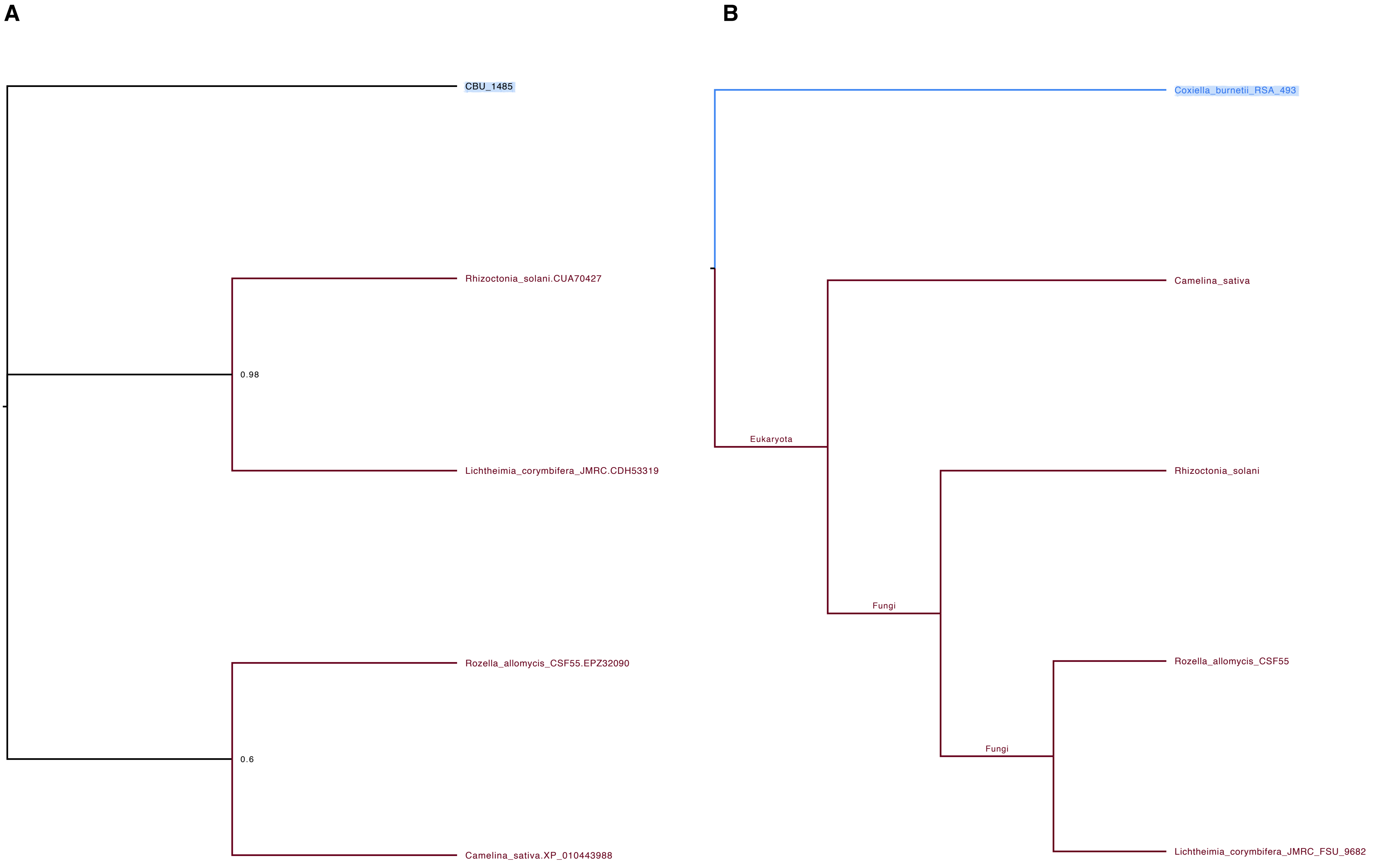

A
CBU_1485
Rhizoctonia_solani.CUA70427
0.98
Lichtheimia_corymbifera_JMRC.CDH53319
Rozella_allomycis_CSF55.EPZ32090
0.6
Camelina_sativa.XP_010443988
B
Coxiella_burnetii_RSA_493
Camelina_sativa
Eukaryota
Rhizoctonia_solani
Fungi
Rozella_allomycis_CSF55
Fungi
Lichtheimia_corymbifera_JMRC_FSU_9682

CBU_1458

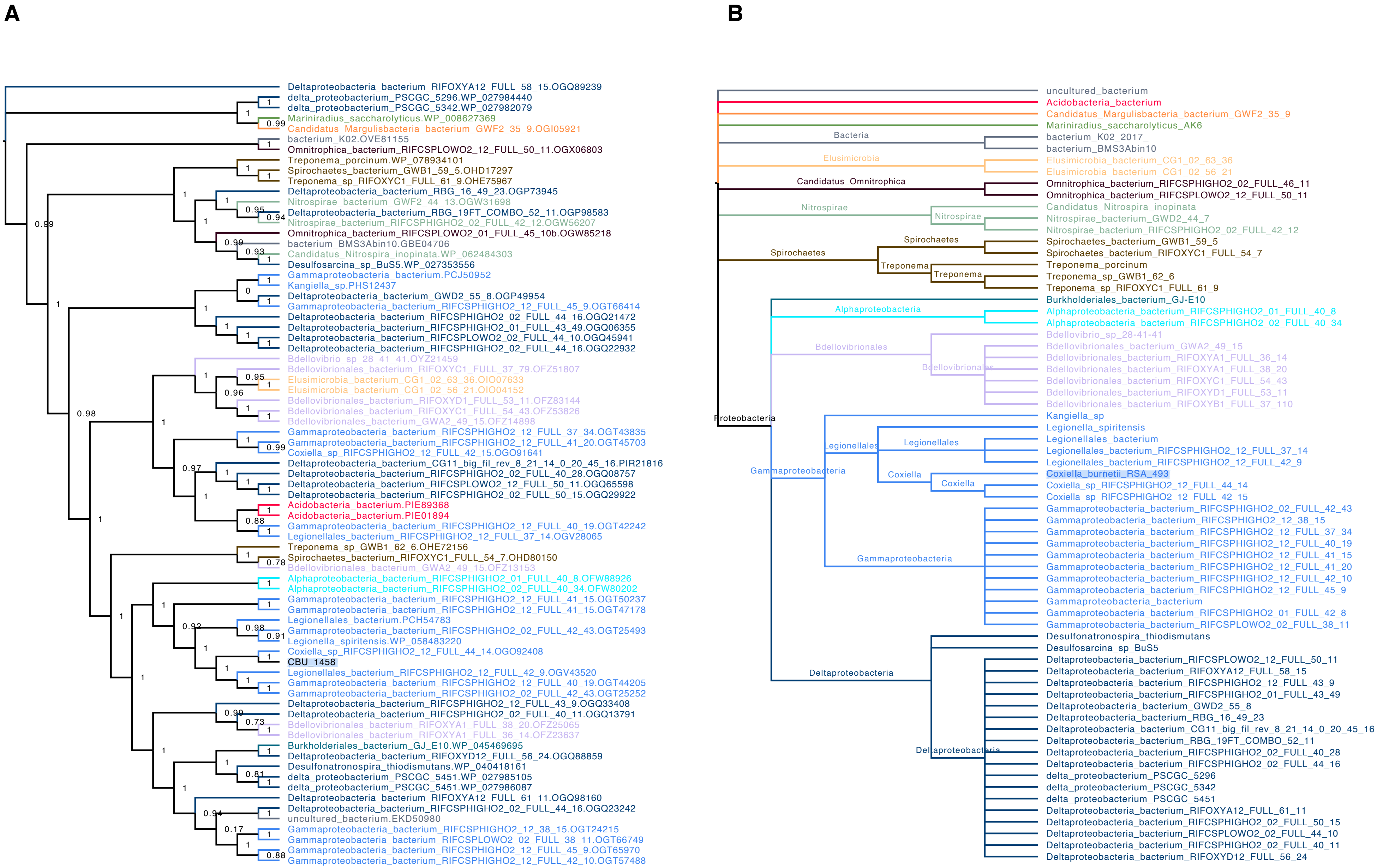

A
Deltaproteobacteria_bacterium_RIFOXYA12_FULL_58_15.OGQ89239
delta_proteobacterium_PSCGC_5296.WP_027984440
delta_proteobacterium_PSCGC_5342.WP_027982079
Mariniradius_saccharolyticus.WP_008627369
Candidatus_Margulisbacteria_bacterium_GWF2_35_9.OGI05921
bacterium_K02.OVE81155
Omnitrophica_bacterium_RIFCSPLOWO2_12_FULL_50_11.OGX06803
Treponema_porcinum.WP_078934101
Spirochaetes_bacterium_GWB1_59_5.OHD17297
Treponema_sp_RIFOXYC1_FULL_61_9.OHE75967
Deltaproteobacteria_bacterium_RBG_16_49_23.OGP73945
Nitrospirae_bacterium_GWF2_44_13.OGW31698
Deltaproteobacteria_bacterium_RBG_19FT_COMBO_52_11.OGP98583
Nitrospirae_bacterium_RIFCSPHIGHO2_02_FULL_42_12.OGW56207
Omnitrophica_bacterium_RIFCSPLOWO2_01_FULL_45_10b.OGW85218
bacterium_BMS3Abin10.GBE04706
Candidatus_Nitrospira_inopinata.WP_062484303
Desulfosarcina_sp_BuS5.WP_027353556
Gammaproteobacteria_bacterium.PCJ50952
Kangiella_sp.PHS12437
Deltaproteobacteria_bacterium_GWD2_55_8.OGP49954
Gammaproteobacteria_bacterium_RIFCSPHIGHO2_12_FULL_45_9.OGT66414
Deltaproteobacteria_bacterium_RIFCSPHIGHO2_02_FULL_44_16.OGQ21472
Deltaproteobacteria_bacterium_RIFCSPHIGHO2_01_FULL_43_49.OGQ06355
Deltaproteobacteria_bacterium_RIFCSPLOWO2_02_FULL_44_10.OGQ45941
Deltaproteobacteria_bacterium_RIFCSPHIGHO2_02_FULL_44_16.OGQ22932
Bdellovibrio_sp_28_41_41.OYZ21459
Bdellovibrionales_bacterium_RIFOXYC1_FULL_37_79.OFZ51807
Elusimicrobia_bacterium_CG1_02_63_36.OIO07633
Elusimicrobia_bacterium_CG1_02_56_21.OIO04152
Bdellovibrionales_bacterium_RIFOXYD1_FULL_53_11.OFZ83144
Bdellovibrionales_bacterium_RIFOXYC1_FULL_54_43.OFZ53826
Bdellovibrionales_bacterium_GWA2_49_15.OFZ14898
Gammaproteobacteria_bacterium_RIFCSPHIGHO2_12_FULL_37_34.OGT43835
Gammaproteobacteria_bacterium_RIFCSPHIGHO2_12_FULL_41_20.OGT45703
Coxiella_sp_RIFCSPHIGHO2_12_FULL_42_15.OGO91641
Deltaproteobacteria_bacterium_CG11_big_fil_rev_8_21_14_0_20_45_16.PIR21816
Deltaproteobacteria_bacterium_RIFCSPHIGHO2_02_FULL_40_28.OGQ08757
Deltaproteobacteria_bacterium_RIFCSPLOWO2_12_FULL_50_11.OGQ65598
Deltaproteobacteria_bacterium_RIFCSPHIGHO2_02_FULL_50_15.OGQ29922
Acidobacteria_bacterium.PIE89368
Acidobacteria_bacterium.PIE01894
Gammaproteobacteria_bacterium_RIFCSPHIGHO2_12_FULL_40_19.OGT42242
Legionellales_bacterium_RIFCSPHIGHO2_12_FULL_37_14.OGV28065
Treponema_sp_GWB1_62_6.OHE72156
Spirochaetes_bacterium_RIFOXYC1_FULL_54_7.OHD80150
Bdellovibrionales_bacterium_GWA2_49_15.OFZ13153
Alphaproteobacteria_bacterium_RIFCSPHIGHO2_01_FULL_40_8.OFW88926
Alphaproteobacteria_bacterium_RIFCSPHIGHO2_02_FULL_40_34.OFW80202
Gammaproteobacteria_bacterium_RIFCSPHIGHO2_12_FULL_41_15.OGT50237
Gammaproteobacteria_bacterium_RIFCSPHIGHO2_12_FULL_41_15.OGT47178
Legionellales_bacterium.PCH54783
Gammaproteobacteria_bacterium_RIFCSPHIGHO2_02_FULL_42_43.OGT25493
Legionella_spiritensis.WP_058483220
Coxiella_sp_RIFCSPHIGHO2_12_FULL_44_14.OGO92408
CBU_1458
Legionellales_bacterium_RIFCSPHIGHO2_12_FULL_42_9.OGV43520
Gammaproteobacteria_bacterium_RIFCSPHIGHO2_12_FULL_40_19.OGT44205
Gammaproteobacteria_bacterium_RIFCSPHIGHO2_02_FULL_42_43.OGT25252
Deltaproteobacteria_bacterium_RIFCSPHIGHO2_12_FULL_43_9.OGQ33408
Deltaproteobacteria_bacterium_RIFCSPHIGHO2_02_FULL_40_11.OGQ13791
Bdellovibrionales_bacterium_RIFOXYA1_FULL_38_20.OFZ25065
Bdellovibrionales_bacterium_RIFOXYA1_FULL_36_14.OFZ23637
Burkholderiales_bacterium_GJ_E10.WP_045469695
Deltaproteobacteria_bacterium_RIFOXYD12_FULL_56_24.OGQ88859
Desulfonatronospira_thiodismutans.WP_040418161
delta_proteobacterium_PSCGC_5451.WP_027985105
delta_proteobacterium_PSCGC_5451.WP_027986087
Deltaproteobacteria_bacterium_RIFOXYA12_FULL_61_11.OGQ98160
Deltaproteobacteria_bacterium_RIFCSPHIGHO2_02_FULL_44_16.OGQ23242
uncultured_bacterium.EKD50980
Gammaproteobacteria_bacterium_RIFCSPHIGHO2_12_38_15.OGT24215
Gammaproteobacteria_bacterium_RIFCSPLOWO2_02_FULL_38_11.OGT66749
Gammaproteobacteria_bacterium_RIFCSPHIGHO2_12_FULL_45_9.OGT65970
Gammaproteobacteria_bacterium_RIFCSPHIGHO2_12_FULL_42_10.OGT57488
B
uncultured_bacterium
Acidobacteria_bacterium
Candidatus_Margulisbacteria_bacterium_GWF2_35_9
Mariniradius_saccharolyticus_AK6
Bacteria
bacterium_K02_2017_
bacterium_BMS3Abin10
Elusimicrobia
Elusimicrobia_bacterium_CG1_02_63_36
Elusimicrobia_bacterium_CG1_02_56_21
Candidatus_Omnitrophica
Omnitrophica_bacterium_RIFCSPHIGHO2_02_FULL_46_11
Omnitrophica_bacterium_RIFCSPLOWO2_12_FULL_50_11
Nitrospirae
Candidatus_Nitrospira_inopinata
Nitrospirae_bacterium_GWD2_44_7
Nitrospirae_bacterium_RIFCSPHIGHO2_02_FULL_42_12
Spirochaetes
Spirochaetes_bacterium_GWB1_59_5
Spirochaetes_bacterium_RIFOXYC1_FULL_54_7
Treponema
Treponema_porcinum
Treponema_sp_GWB1_62_6
Treponema_sp_RIFOXYC1_FULL_61_9
Burkholderiales_bacterium_GJ-E10
Alphaproteobacteria
Alphaproteobacteria_bacterium_RIFCSPHIGHO2_01_FULL_40_8
Alphaproteobacteria_bacterium_RIFCSPHIGHO2_02_FULL_40_34
Bdellovibrio_sp_28-41-41
Bdellovibrionales
Bdellovibrionales_bacterium_GWA2_49_15
Bdellovibrionales_bacterium_RIFOXYA1_FULL_36_14
Bdellovibrionales_bacterium_RIFOXYA1_FULL_38_20
Bdellovibrionales_bacterium_RIFOXYC1_FULL_54_43
Bdellovibrionales_bacterium_RIFOXYD1_FULL_53_11
Bdellovibrionales_bacterium_RIFOXYB1_FULL_37_110
Proteobacteria
Kangiella_sp
Legionella_spiritensis
Legionellales
Legionellales_bacterium
Legionellales_bacterium_RIFCSPHIGHO2_12_FULL_37_14
Legionellales_bacterium_RIFCSPHIGHO2_12_FULL_42_9
Gammaproteobacteria
Coxiella
Coxiella_burnetii_RSA_493
Coxiella_sp_RIFCSPHIGHO2_12_FULL_44_14
Coxiella_sp_RIFCSPHIGHO2_12_FULL_42_15
Gammaproteobacteria_bacterium_RIFCSPHIGHO2_02_FULL_42_43
Gammaproteobacteria_bacterium_RIFCSPHIGHO2_12_38_15
Gammaproteobacteria_bacterium_RIFCSPHIGHO2_12_FULL_37_34
Gammaproteobacteria_bacterium_RIFCSPHIGHO2_12_FULL_40_19
Gammaproteobacteria_bacterium_RIFCSPHIGHO2_12_FULL_41_15
Gammaproteobacteria_bacterium_RIFCSPHIGHO2_12_FULL_41_20
Gammaproteobacteria_bacterium_RIFCSPHIGHO2_12_FULL_42_10
Gammaproteobacteria_bacterium_RIFCSPHIGHO2_12_FULL_45_9
Gammaproteobacteria_bacterium
Gammaproteobacteria_bacterium_RIFCSPHIGHO2_01_FULL_42_8
Gammaproteobacteria_bacterium_RIFCSPLOWO2_02_FULL_38_11
Desulfonatronospira_thiodismutans
Desulfosarcina_sp_BuS5
Deltaproteobacteria
Deltaproteobacteria_bacterium_RIFCSPLOWO2_12_FULL_50_11
Deltaproteobacteria_bacterium_RIFOXYA12_FULL_58_15
Deltaproteobacteria_bacterium_RIFCSPHIGHO2_12_FULL_43_9
Deltaproteobacteria_bacterium_RIFCSPHIGHO2_01_FULL_43_49
Deltaproteobacteria_bacterium_GWD2_55_8
Deltaproteobacteria_bacterium_RBG_16_49_23
Deltaproteobacteria_bacterium_CG11_big_fil_rev_8_21_14_0_20_45_16
Deltaproteobacteria_bacterium_RBG_19FT_COMBO_52_11
Deltaproteobacteria_bacterium_RIFCSPHIGHO2_02_FULL_40_28
Deltaproteobacteria_bacterium_RIFCSPHIGHO2_02_FULL_44_16
delta_proteobacterium_PSCGC_5296
delta_proteobacterium_PSCGC_5342
delta_proteobacterium_PSCGC_5451
Deltaproteobacteria_bacterium_RIFOXYA12_FULL_61_11
Deltaproteobacteria_bacterium_RIFCSPHIGHO2_02_FULL_50_15
Deltaproteobacteria_bacterium_RIFCSPLOWO2_02_FULL_44_10
Deltaproteobacteria_bacterium_RIFCSPHIGHO2_02_FULL_40_11
Deltaproteobacteria_bacterium_RIFOXYD12_FULL_56_24

CBU_1333

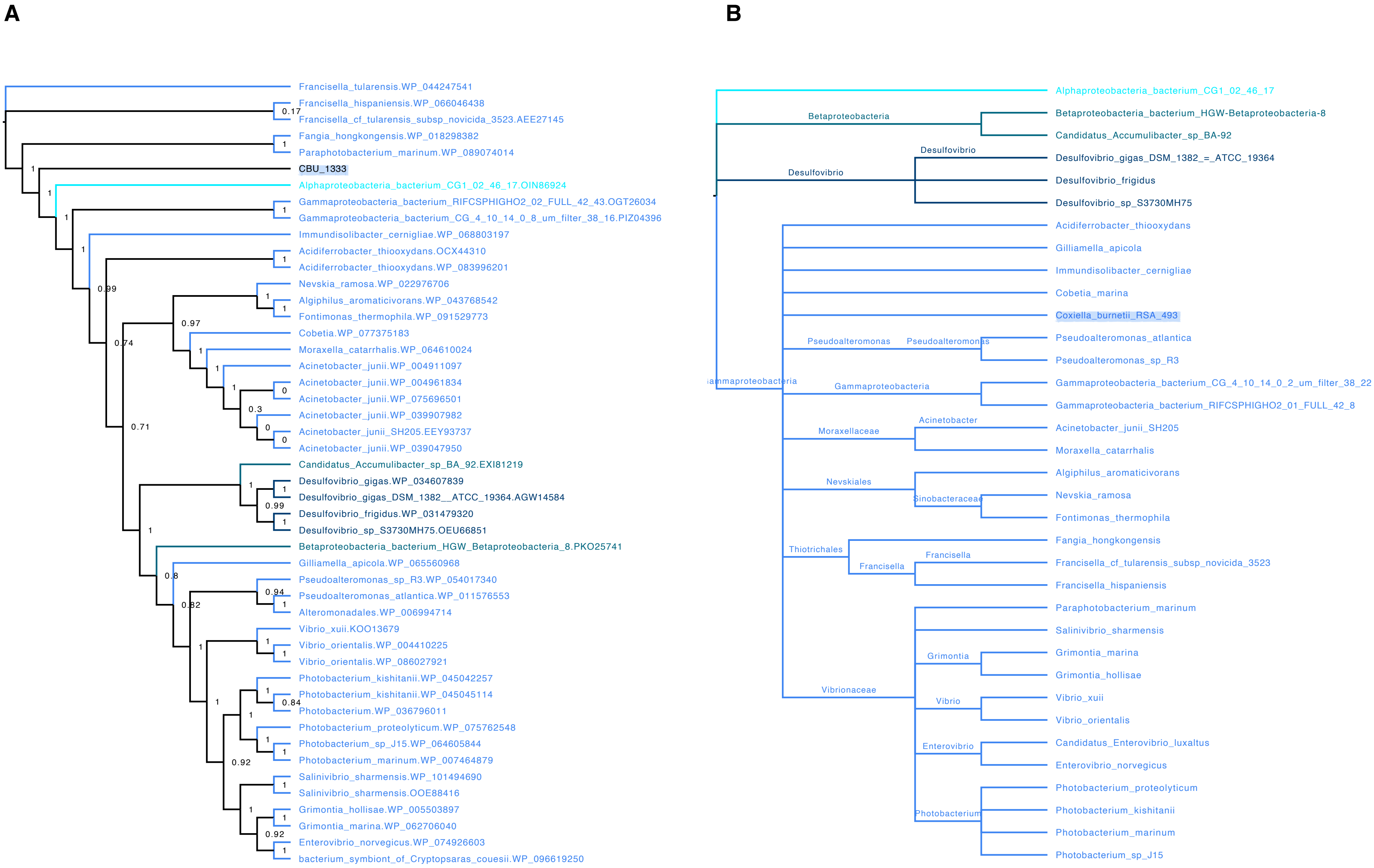

A
Francisella_tularensis.WP_044247541
Francisella_hispaniensis.WP_066046438
Francisella_cf_tularensis_subsp_novicida_3523.AEE27145
Fangia_hongkongensis.WP_018298382
Paraphotobacterium_marinum.WP_089074014
CBU_1333
Alphaproteobacteria_bacterium_CG1_02_46_17.OIN86924
Gammaproteobacteria_bacterium_RIFCSPHIGHO2_02_FULL_42_43.OGT26034
Gammaproteobacteria_bacterium_CG_4_10_14_0_8_um_filter_38_16.PIZ04396
Immundisolibacter_cernigliae.WP_068803197
Acidiferrobacter_thiooxydans.OCX44310
Acidiferrobacter_thiooxydans.WP_083996201
Nevskia_ramosa.WP_022976706
Algiphilus_aromaticivorans.WP_043768542
Fontimonas_thermophila.WP_091529773
Cobetia.WP_077375183
Moraxella_catarrhalis.WP_064610024
Acinetobacter_junii.WP_004911097
Acinetobacter_junii.WP_004961834
Acinetobacter_junii.WP_075696501
Acinetobacter_junii.WP_039907982
Acinetobacter_junii_SH205.EEY93737
Acinetobacter_junii.WP_039047950
Candidatus_Accumulibacter_sp_BA_92.EXI81219
Desulfovibrio_gigas.WP_034607839
Desulfovibrio_gigas_DSM_1382__ATCC_19364.AGW14584
Desulfovibrio_frigidus.WP_031479320
Desulfovibrio_sp_S3730MH75.OEU66851
Betaproteobacteria_bacterium_HGW_Betaproteobacteria_8.PKO25741
Gilliamella_apicola.WP_065560968
Pseudoalteromonas_sp_R3.WP_054017340
Pseudoalteromonas_atlantica.WP_011576553
Alteromonadales.WP_006994714
Vibrio_xuii.KOO13679
Vibrio_orientalis.WP_004410225
Vibrio_orientalis.WP_086027921
Photobacterium_kishitanii.WP_045042257
Photobacterium_kishitanii.WP_045045114
Photobacterium.WP_036796011
Photobacterium_proteolyticum.WP_075762548
Photobacterium_sp_J15.WP_064605844
Photobacterium_marinum.WP_007464879
Salinivibrio_sharmensis.WP_101494690
Salinivibrio_sharmensis.OOE88416
Grimontia_hollisae.WP_005503897
Grimontia_marina.WP_062706040
Enterovibrio_norvegicus.WP_074926603
bacterium_symbiont_of_Cryptopsaras_couesii.WP_096619250
B
Alphaproteobacteria_bacterium_CG1_02_46_17
Betaproteobacteria
Betaproteobacteria_bacterium_HGW-Betaproteobacteria-8
Candidatus_Accumulibacter_sp_BA-92
Desulfovibrio
Desulfovibrio_gigas_DSM_1382_=_ATCC_19364
Desulfovibrio_frigidus
Desulfovibrio_sp_S3730MH75
Acidiferrobacter_thiooxydans
Gilliamella_apicola
Immundisolibacter_cernigliae
Cobetia_marina
Coxiella_burnetii_RSA_493
Pseudoalteromonas
Pseudoalteromonas_atlantica
Pseudoalteromonas_sp_R3
Gammaproteobacteria
Gammaproteobacteria_bacterium_CG_4_10_14_0_2_um_filter_38_22
Gammaproteobacteria_bacterium_RIFCSPHIGHO2_01_FULL_42_8
Moraxellaceae
Acinetobacter
Acinetobacter_junii_SH205
Moraxella_catarrhalis
Nevskiales
Sinobacteraceae
Algiphilus_aromaticivorans
Nevskia_ramosa
Fontimonas_thermophila
Thiotrichales
Francisella
Fangia_hongkongensis
Francisella_cf_tularensis_subsp_novicida_3523
Francisella_hispaniensis
Paraphotobacterium_marinum
Salinivibrio_sharmensis
Grimontia
Grimontia_marina
Grimontia_hollisae
Vibrionaceae
Vibrio
Vibrio_xuii
Vibrio_orientalis
Enterovibrio
Candidatus_Enterovibrio_luxaltus
Enterovibrio_norvegicus
Photobacterium
Photobacterium_proteolyticum
Photobacterium_kishitanii
Photobacterium_marinum
Photobacterium_sp_J15

CBU_1308

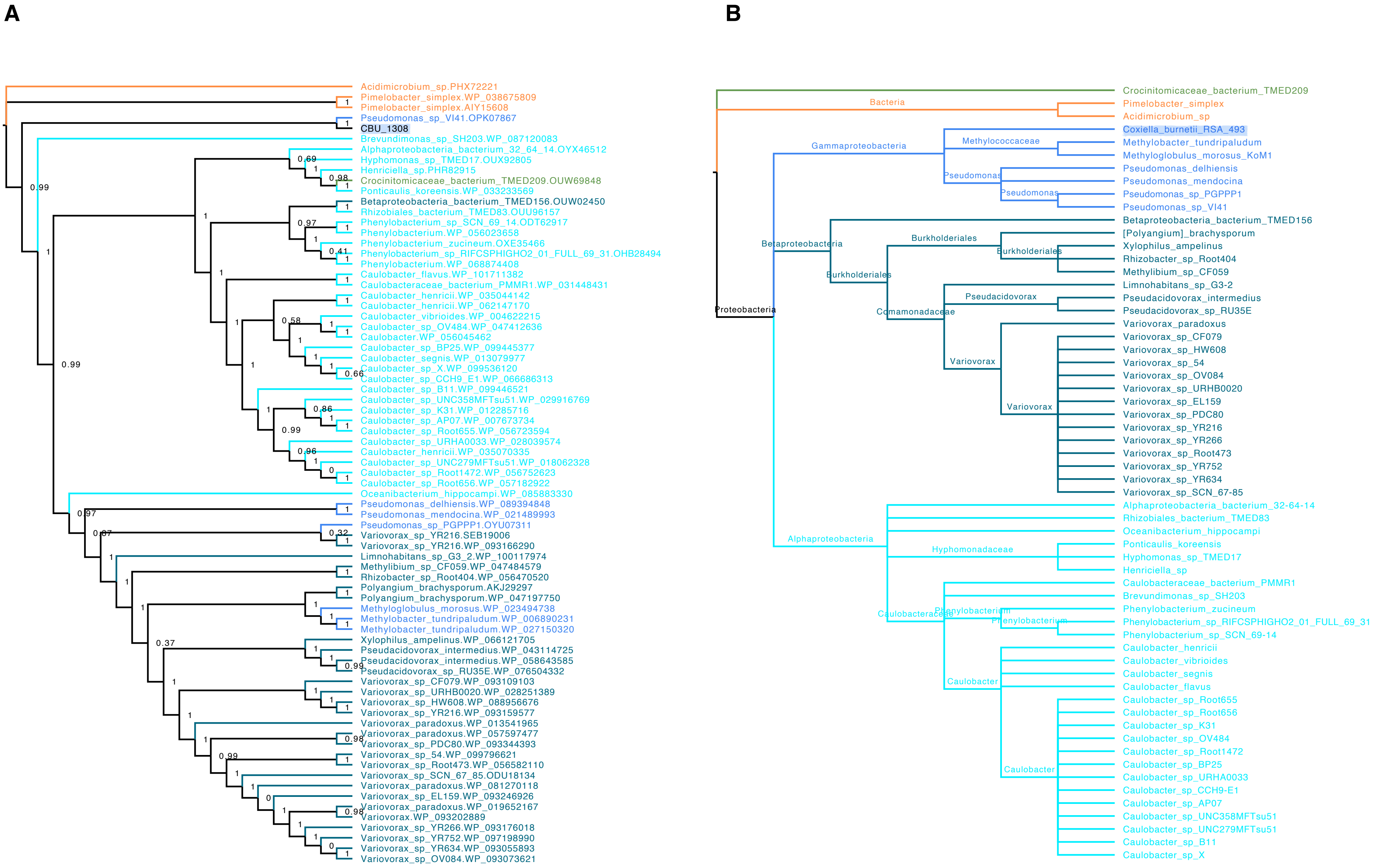

A
Acidimicrobium_sp.PHX72221
Pimelobacter_simplex.WP_038675809
Pimelobacter_simplex.AIY15608
Pseudomonas_sp_VI41.OPK07867
CBU_1308
Brevundimonas_sp_SH203.WP_087120083
Alphaproteobacteria_bacterium_32_64_14.OYX46512
Hyphomonas_sp_TMED17.OUX92805
Henriciella_sp.PHR82915
Crocinitomicaceae_bacterium_TMED209.OUW69848
Ponticaulis_koreensis.WP_033233569
Betaproteobacteria_bacterium_TMED156.OUW02450
Rhizobiales_bacterium_TMED83.OUU96157
Phenylobacterium_sp_SCN_69_14.ODT62917
Phenylobacterium.WP_056023658
Phenylobacterium_zucineum.OXE35466
Phenylobacterium_sp_RIFCSPHIGHO2_01_FULL_69_31.OHB28494
Phenylobacterium.WP_068874408
Caulobacter_flavus.WP_101711382
Caulobacteraceae_bacterium_PMMR1.WP_031448431
Caulobacter_henricii.WP_035044142
Caulobacter_henricii.WP_062147170
Caulobacter_vibrioides.WP_004622215
Caulobacter_sp_OV484.WP_047412636
Caulobacter.WP_056045462
Caulobacter_sp_BP25.WP_099445377
Caulobacter_segnis.WP_013079977
Caulobacter_sp_X.WP_099536120
Caulobacter_sp_CCH9_E1.WP_066686313
Caulobacter_sp_B11.WP_099446521
Caulobacter_sp_UNC358MFTsu51.WP_029916769
Caulobacter_sp_K31.WP_012285716
Caulobacter_sp_AP07.WP_007673734
Caulobacter_sp_Root655.WP_056723594
Caulobacter_sp_URHA0033.WP_028039574
Caulobacter_henricii.WP_035070335
Caulobacter_sp_UNC279MFTsu51.WP_018062328
Caulobacter_sp_Root1472.WP_056752623
Caulobacter_sp_Root656.WP_057182922
Oceanibacterium_hippocampi.WP_085883330
Pseudomonas_delhiensis.WP_089394848
Pseudomonas_mendocina.WP_021489993
Pseudomonas_sp_PGPPP1.OYU07311
Variovorax_sp_YR216.SEB19006
Variovorax_sp_YR216.WP_093166290
Limnohabitans_sp_G3_2.WP_100117974
Methylibium_sp_CF059.WP_047484579
Rhizobacter_sp_Root404.WP_056470520
Polyangium_brachysporum.AKJ29297
Polyangium_brachysporum.WP_047197750
Methyloglobulus_morosus.WP_023494738
Methylobacter_tundripaludum.WP_006890231
Methylobacter_tundripaludum.WP_027150320
Xylophilus_ampelinus.WP_066121705
Pseudacidovorax_intermedius.WP_043114725
Pseudacidovorax_intermedius.WP_058643585
Pseudacidovorax_sp_RU35E.WP_076504332
Variovorax_sp_CF079.WP_093109103
Variovorax_sp_URHB0020.WP_028251389
Variovorax_sp_HW608.WP_088956676
Variovorax_sp_YR216.WP_093159577
Variovorax_paradoxus.WP_013541965
Variovorax_paradoxus.WP_057597477
Variovorax_sp_PDC80.WP_093344393
Variovorax_sp_54.WP_099796621
Variovorax_sp_Root473.WP_056582110
Variovorax_sp_SCN_67_85.ODU18134
Variovorax_paradoxus.WP_081270118
Variovorax_sp_EL159.WP_093246926
Variovorax_paradoxus.WP_019652167
Variovorax.WP_093202889
Variovorax_sp_YR266.WP_093176018
Variovorax_sp_YR752.WP_097198990
Variovorax_sp_YR634.WP_093055893
Variovorax_sp_OV084.WP_093073621
B
Bacteria
Proteobacteria
Gammaproteobacteria
Methylococcaceae
Pseudomonas
Betaproteobacteria
Burkholderiales
Comamonadaceae
Pseudacidovorax
Variovorax
Alphaproteobacteria
Hyphomonadaceae
Caulobacteraceae
Phenylobacterium
Caulobacter
Crocinitomicaceae_bacterium_TMED209
Pimelobacter_simplex
Acidimicrobium_sp
Coxiella_burnetii_RSA_493
Methylobacter_tundripaludum
Methyloglobulus_morosus_KoM1
Pseudomonas_delhiensis
Pseudomonas_mendocina
Pseudomonas_sp_PGPPP1
Pseudomonas_sp_VI41
Betaproteobacteria_bacterium_TMED156
[Polyangium]_brachysporum
Xylophilus_ampelinus
Rhizobacter_sp_Root404
Methylibium_sp_CF059
Limnohabitans_sp_G3-2
Pseudacidovorax_intermedius
Pseudacidovorax_sp_RU35E
Variovorax_paradoxus
Variovorax_sp_CF079
Variovorax_sp_HW608
Variovorax_sp_54
Variovorax_sp_OV084
Variovorax_sp_URHB0020
Variovorax_sp_EL159
Variovorax_sp_PDC80
Variovorax_sp_YR216
Variovorax_sp_YR266
Variovorax_sp_Root473
Variovorax_sp_YR752
Variovorax_sp_YR634
Variovorax_sp_SCN_67-85
Alphaproteobacteria_bacterium_32-64-14
Rhizobiales_bacterium_TMED83
Oceanibacterium_hippocampi
Ponticaulis_koreensis
Hyphomonas_sp_TMED17
Henriciella_sp
Caulobacteraceae_bacterium_PMMR1
Brevundimonas_sp_SH203
Phenylobacterium_zucineum
Phenylobacterium_sp_RIFCSPHIGHO2_01_FULL_69_31
Phenylobacterium_sp_SCN_69-14
Caulobacter_henricii
Caulobacter_vibrioides
Caulobacter_segnis
Caulobacter_flavus
Caulobacter_sp_Root655
Caulobacter_sp_Root656
Caulobacter_sp_K31
Caulobacter_sp_OV484
Caulobacter_sp_Root1472
Caulobacter_sp_BP25
Caulobacter_sp_URHA0033
Caulobacter_sp_CCH9-E1
Caulobacter_sp_AP07
Caulobacter_sp_UNC358MFTsu51
Caulobacter_sp_UNC279MFTsu51
Caulobacter_sp_B11
Caulobacter_sp_X

CBU_1217

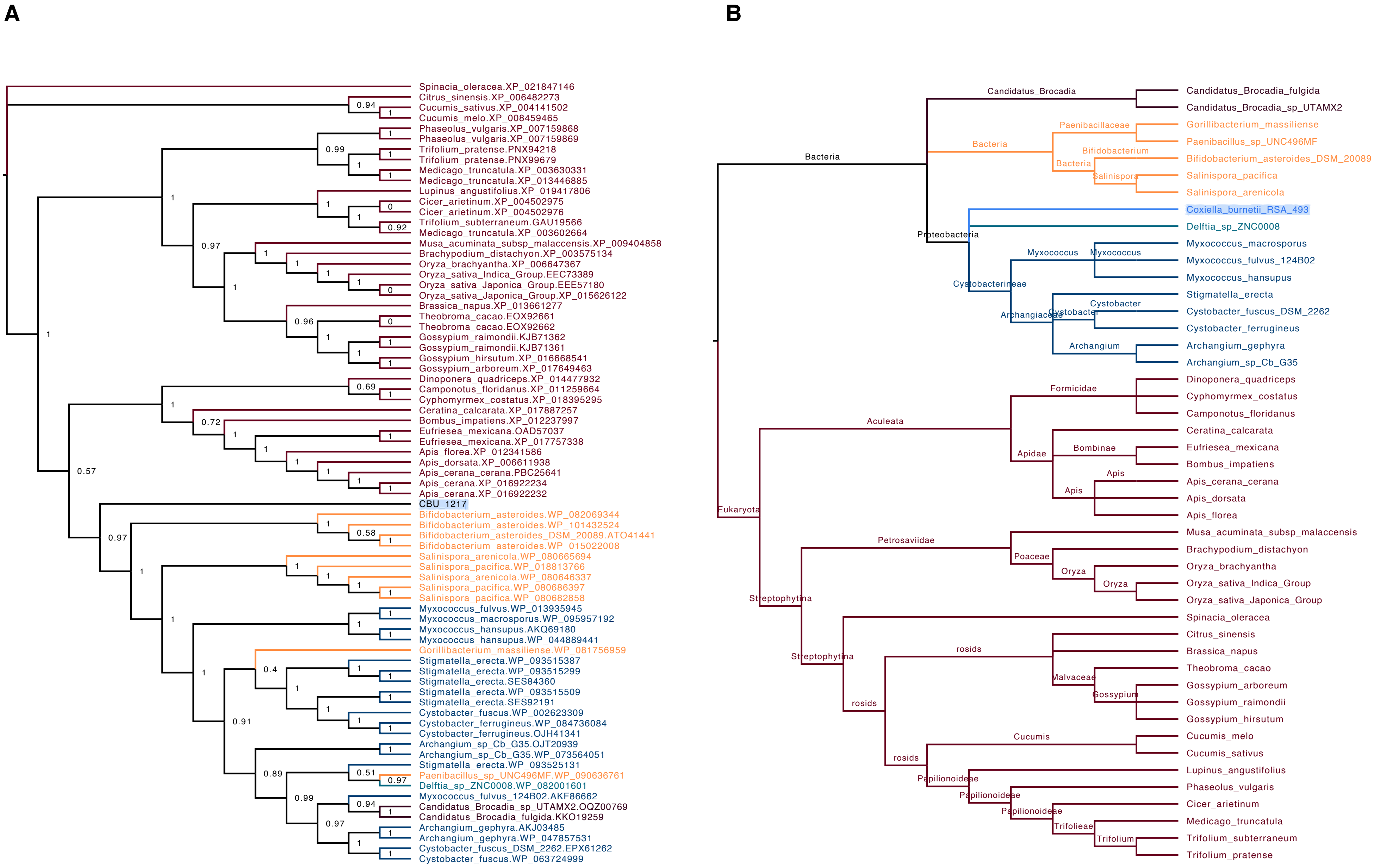
A
Spinacia_oleracea.XP_021847146
Citrus_sinensis.XP_006482273
Cucumis_sativus.XP_004141502
Cucumis_melo.XP_008459465
Phaseolus_vulgaris.XP_007159868
Phaseolus_vulgaris.XP_007159869
Trifolium_pratense.PNX94218
Trifolium_pratense.PNX99679
Medicago_truncatula.XP_003630331
Medicago_truncatula.XP_013446885
Lupinus_angustifolius.XP_019417806
Cicer_arietinum.XP_004502975
Cicer_arietinum.XP_004502976
Trifolium_subterraneum.GAU19566
Medicago_truncatula.XP_003602664
Musa_acuminata_subsp_malaccensis.XP_009404858
Brachypodium_distachyon.XP_003575134
Oryza_brachyantha.XP_006647367
Oryza_sativa_Indica_Group.EEC73389
Oryza_sativa_Japonica_Group.EEE57180
Oryza_sativa_Japonica_Group.XP_015626122
Brassica_napus.XP_013661277
Theobroma_cacao.EOX92661
Theobroma_cacao.EOX92662
Gossypium_raimondii.KJB71362
Gossypium_raimondii.KJB71361
Gossypium_hirsutum.XP_016668541
Gossypium_arboreum.XP_017649463
Dinoponera_quadriceps.XP_014477932
Camponotus_floridanus.XP_011259664
Cyphomyrmex_costatus.XP_018395295
Ceratina_calcarata.XP_017887257
Bombus_impatiens.XP_012237997
Eufriesea_mexicana.OAD57037
Eufriesea_mexicana.XP_017757338
Apis_florea.XP_012341586
Apis_dorsata.XP_006611938
Apis_cerana_cerana.PBC25641
Apis_cerana.XP_016922234
Apis_cerana.XP_016922232
CBU_1217
Bifidobacterium_asteroides.WP_082069344
Bifidobacterium_asteroides.WP_101432524
Bifidobacterium_asteroides_DSM_20089.ATO41441
Bifidobacterium_asteroides.WP_015022008
Salinispora_arenicola.WP_080665694
Salinispora_pacifica.WP_018813766
Salinispora_arenicola.WP_080646337
Salinispora_pacifica.WP_080686397
Salinispora_pacifica.WP_080682858
Myxococcus_fulvus.WP_013935945
Myxococcus_macrosporus.WP_095957192
Myxococcus_hansupus.AKQ69180
Myxococcus_hansupus.WP_044889441
Gorillibacterium_massiliense.WP_081756959
Stigmatella_erecta.WP_093515387
Stigmatella_erecta.WP_093515299
Stigmatella_erecta.SES84360
Stigmatella_erecta.WP_093515509
Stigmatella_erecta.SES92191
Cystobacter_fuscus.WP_002623309
Cystobacter_ferrugineus.WP_084736084
Cystobacter_ferrugineus.OJH41341
Archangium_sp_Cb_G35.OJT20939
Archangium_sp_Cb_G35.WP_073564051
Stigmatella_erecta.WP_093525131
Paenibacillus_sp_UNC496MF.WP_090636761
Delftia_sp_ZNC0008.WP_082001601
Myxococcus_fulvus_124B02.AKF86662
Candidatus_Brocadia_sp_UTAMX2.OQZ00769
Candidatus_Brocadia_fulgida.KKO19259
Archangium_gephyra.AKJ03485
Archangium_gephyra.WP_047857531
Cystobacter_fuscus_DSM_2262.EPX61262
Cystobacter_fuscus.WP_063724999
B
Candidatus_Brocadia_fulgida
Candidatus_Brocadia_sp_UTAMX2
Gorillibacterium_massiliense
Paenibacillus_sp_UNC496MF
Bifidobacterium_asteroides_DSM_20089
Salinispora_pacifica
Salinispora_arenicola
Coxiella_burnetii_RSA_493
Delftia_sp_ZNC0008
Myxococcus_macrosporus
Myxococcus_fulvus_124B02
Myxococcus_hansupus
Stigmatella_erecta
Cystobacter_fuscus_DSM_2262
Cystobacter_ferrugineus
Archangium_gephyra
Archangium_sp_Cb_G35
Dinoponera_quadriceps
Cyphomyrmex_costatus
Camponotus_floridanus
Ceratina_calcarata
Eufriesea_mexicana
Bombus_impatiens
Apis_cerana_cerana
Apis_dorsata
Apis_florea
Musa_acuminata_subsp_malaccensis
Brachypodium_distachyon
Oryza_brachyantha
Oryza_sativa_Indica_Group
Oryza_sativa_Japonica_Group
Spinacia_oleracea
Citrus_sinensis
Brassica_napus
Theobroma_cacao
Gossypium_arboreum
Gossypium_raimondii
Gossypium_hirsutum
Cucumis_melo
Cucumis_sativus
Lupinus_angustifolius
Phaseolus_vulgaris
Cicer_arietinum
Medicago_truncatula
Trifolium_subterraneum
Trifolium_pratense

CBU_1206

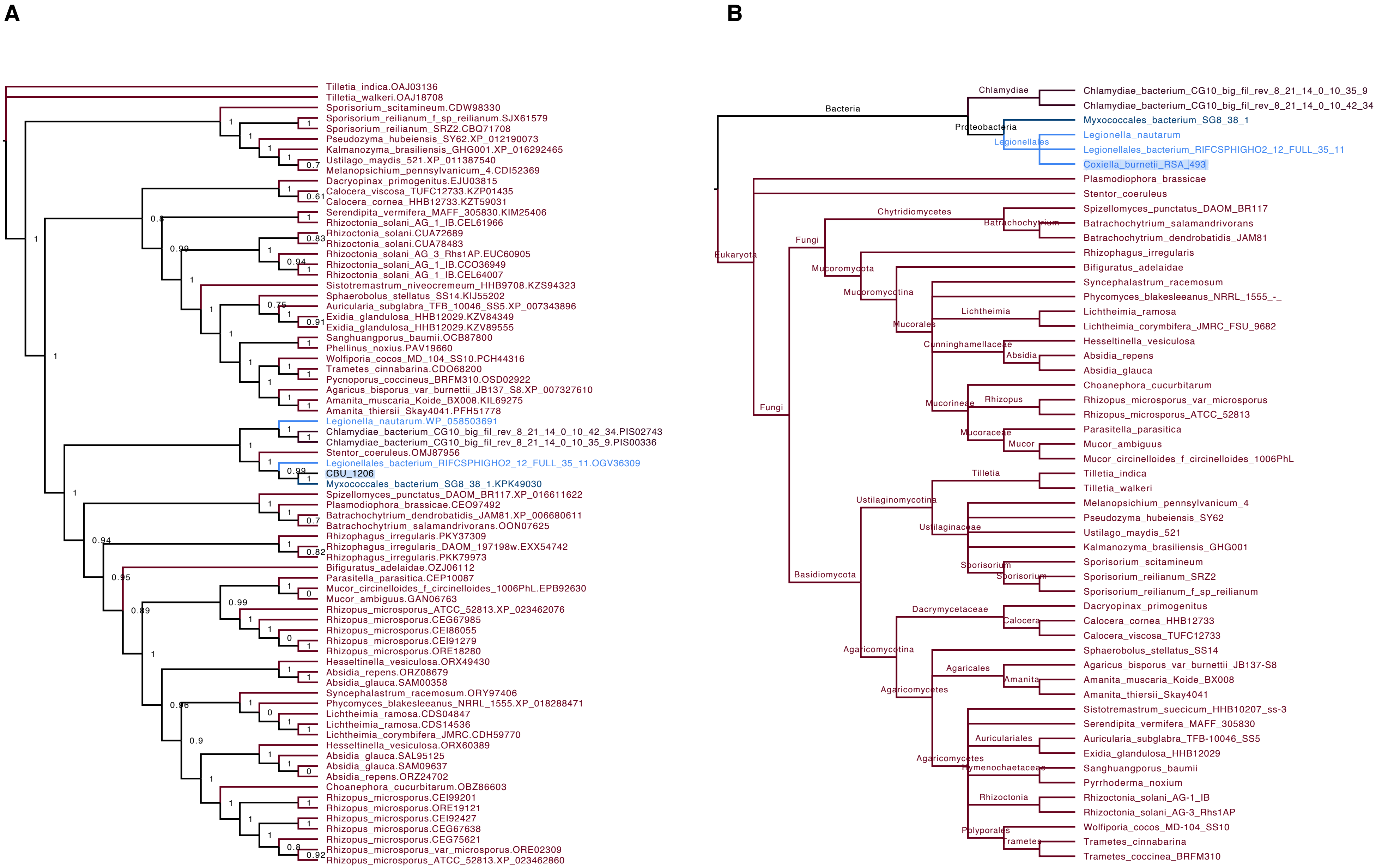

A
Tilletia_indica.OAJ03136
Tilletia_walkeri.OAJ18708
Sporisorium_scitamineum.CDW98330
Sporisorium_reilianum_f_sp_reilianum.SJX61579
Sporisorium_reilianum_SRZ2.CBQ71708
Pseudozyma_hubeiensis_SY62.XP_012190073
Kalmanozyma_brasiliensis_GHG001.XP_016292465
Ustilago_maydis_521.XP_011387540
Melanopsichium_pennsylvanicum_4.CDI52369
Dacryopinax_primogenitus.EJU03815
Calocera_viscosa_TUFC12733.KZP01435
Calocera_cornea_HHB12733.KZT59031
Serendipita_vermifera_MAFF_305830.KIM25406
Rhizoctonia_solani_AG_1_IB.CEL61966
Rhizoctonia_solani.CUA72689
Rhizoctonia_solani.CUA78483
Rhizoctonia_solani_AG_3_Rhs1AP.EUC60905
Rhizoctonia_solani_AG_1_IB.CCO36949
Rhizoctonia_solani_AG_1_IB.CEL64007
Sistotremastrum_niveocremeum_HHB9708.KZS94323
Sphaerobolus_stellatus_SS14.KIJ55202
Auricularia_subglabra_TFB_10046_SS5.XP_007343896
Exidia_glandulosa_HHB12029.KZV84349
Exidia_glandulosa_HHB12029.KZV89555
Sanghuangporus_baumii.OCB87800
Phellinus_noxius.PAV19660
Wolfiporia_cocos_MD_104_SS10.PCH44316
Trametes_cinnabarina.CDO68200
Pycnoporus_coccineus_BRFM310.OSD02922
Agaricus_bisporus_var_burnettii_JB137_S8.XP_007327610
Amanita_muscaria_Koide_BX008.KIL69275
Amanita_thiersii_Skay4041.PFH51778
Legionella_nautarum.WP_058503691
Chlamydiae_bacterium_CG10_big_fil_rev_8_21_14_0_10_42_34.PIS02743
Chlamydiae_bacterium_CG10_big_fil_rev_8_21_14_0_10_35_9.PIS00336
Stentor_coeruleus.OMJ87956
Legionellales_bacterium_RIFCSPHIGHO2_12_FULL_35_11.OGV36309
CBU_1206
Myxococcales_bacterium_SG8_38_1.KPK49030
Spizellomyces_punctatus_DAOM_BR117.XP_016611622
Plasmodiophora_brassicae.CEO97492
Batrachochytrium_dendrobatidis_JAM81.XP_006680611
Batrachochytrium_salamandrivorans.OON07625
Rhizophagus_irregularis.PKY37309
Rhizophagus_irregularis_DAOM_197198w.EXX54742
Rhizophagus_irregularis.PKK79973
Bifiguratus_adelaidae.OZJ06112
Parasitella_parasitica.CEP10087
Mucor_circinelloides_f_circinelloides_1006PhL.EPB92630
Mucor_ambiguus.GAN06763
Rhizopus_microsporus_ATCC_52813.XP_023462076
Rhizopus_microsporus.CEG67985
Rhizopus_microsporus.CEI86055
Rhizopus_microsporus.CEI91279
Rhizopus_microsporus.ORE18280
Hesseltinella_vesiculosa.ORX49430
Absidia_repens.ORZ08679
Absidia_glauca.SAM00358
Syncephalastrum_racemosum.ORY97406
Phycomyces_blakesleeanus_NRRL_1555.XP_018288471
Lichtheimia_ramosa.CDS04847
Lichtheimia_ramosa.CDS14536
Lichtheimia_corymbifera_JMRC.CDH59770
Hesseltinella_vesiculosa.ORX60389
Absidia_glauca.SAL95125
Absidia_glauca.SAM09637
Absidia_repens.ORZ24702
Choanephora_cucurbitarum.OBZ86603
Rhizopus_microsporus.CEI99201
Rhizopus_microsporus.ORE19121
Rhizopus_microsporus.CEI92427
Rhizopus_microsporus.CEG67638
Rhizopus_microsporus.CEG75621
Rhizopus_microsporus_var_microsporus.ORE02309
Rhizopus_microsporus_ATCC_52813.XP_023462860
B
Bacteria
Chlamydiae
Proteobacteria
Legionellales
Eukaryota
Fungi
Chytridiomycetes
Batrachochytrium
Mucoromycota
Mucoromycotina
Mucorales
Lichtheimia
Cunninghamellaceae
Absidia
Mucorineae
Rhizopus
Mucoraceae
Mucor
Ustilaginomycotina
Tilletia
Ustilaginaceae
Sporisorium
Basidiomycota
Dacrymycetaceae
Calocera
Agaricomycotina
Agaricales
Amanita
Agaricomycetes
Auriculariales
Hymenochaetaceae
Rhizoctonia
Polyporales
Trametes
Chlamydiae_bacterium_CG10_big_fil_rev_8_21_14_0_10_35_9
Chlamydiae_bacterium_CG10_big_fil_rev_8_21_14_0_10_42_34
Myxococcales_bacterium_SG8_38_1
Legionella_nautarum
Legionellales_bacterium_RIFCSPHIGHO2_12_FULL_35_11
Coxiella_burnetii_RSA_493
Plasmodiophora_brassicae
Stentor_coeruleus
Spizellomyces_punctatus_DAOM_BR117
Batrachochytrium_salamandrivorans
Batrachochytrium_dendrobatidis_JAM81
Rhizophagus_irregularis
Bifiguratus_adelaidae
Syncephalastrum_racemosum
Phycomyces_blakesleeanus_NRRL_1555_-_
Lichtheimia_ramosa
Lichtheimia_corymbifera_JMRC_FSU_9682
Hesseltinella_vesiculosa
Absidia_repens
Absidia_glauca
Choanephora_cucurbitarum
Rhizopus_microsporus_var_microsporus
Rhizopus_microsporus_ATCC_52813
Parasitella_parasitica
Mucor_ambiguus
Mucor_circinelloides_f_circinelloides_1006PhL
Tilletia_indica
Tilletia_walkeri
Melanopsichium_pennsylvanicum_4
Pseudozyma_hubeiensis_SY62
Ustilago_maydis_521
Kalmanozyma_brasiliensis_GHG001
Sporisorium_scitamineum
Sporisorium_reilianum_SRZ2
Sporisorium_reilianum_f_sp_reilianum
Dacryopinax_primogenitus
Calocera_cornea_HHB12733
Calocera_viscosa_TUFC12733
Sphaerobolus_stellatus_SS14
Agaricus_bisporus_var_burnettii_JB137-S8
Amanita_muscaria_Koide_BX008
Amanita_thiersii_Skay4041
Sistotremastrum_suecicum_HHB10207_ss-3
Serendipita_vermifera_MAFF_305830
Auricularia_subglabra_TFB-10046_SS5
Exidia_glandulosa_HHB12029
Sanghuangporus_baumii
Pyrrhoderma_noxium
Rhizoctonia_solani_AG-1_IB
Rhizoctonia_solani_AG-3_Rhs1AP
Wolfiporia_cocos_MD-104_SS10
Trametes_cinnabarina
Trametes_coccinea_BRFM310

CBU_1151

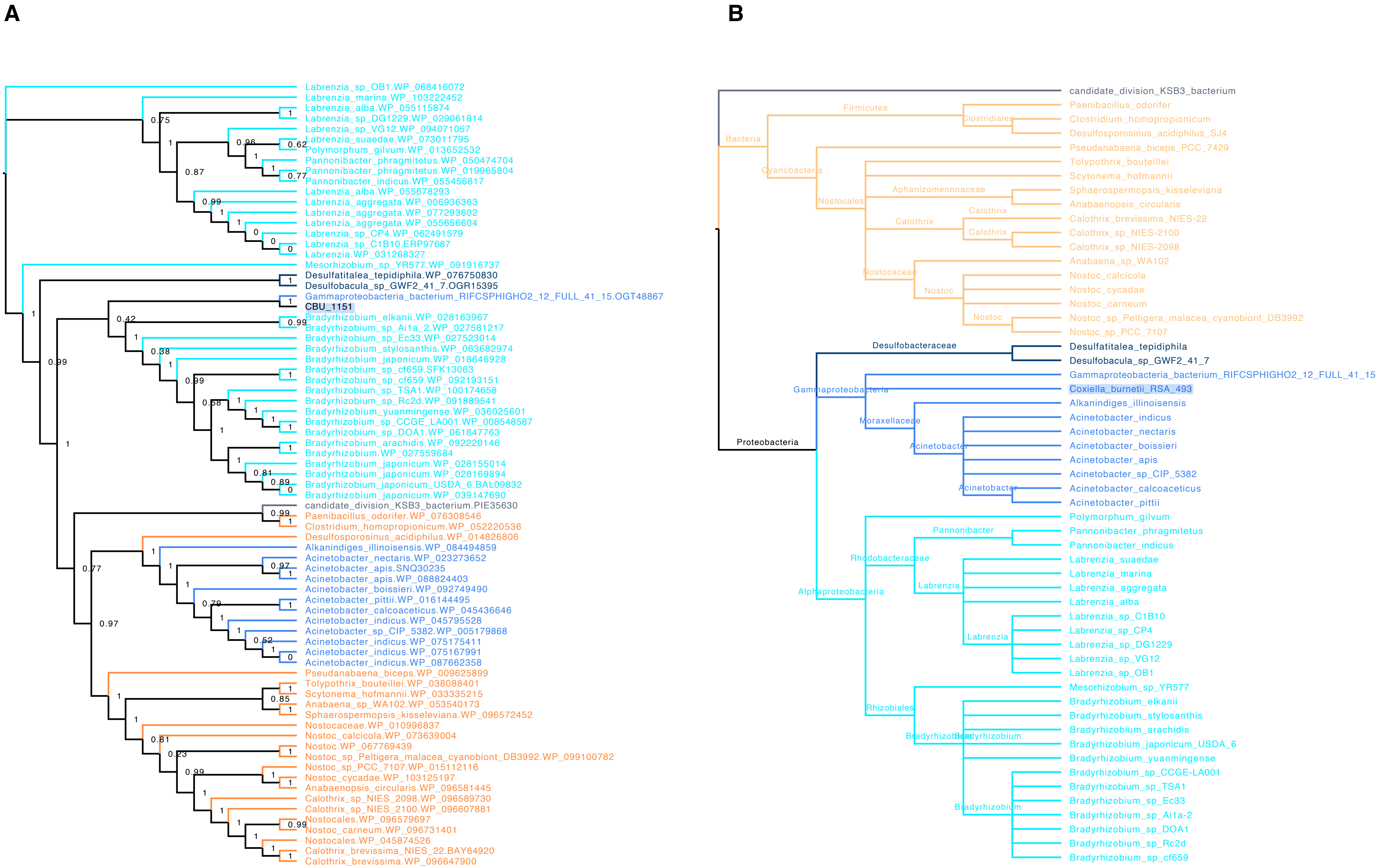

A
Labrenzia_sp_OB1.WP_068416072
Labrenzia_marina.WP_103222452
Labrenzia_alba.WP_055115874
Labrenzia_sp_DG1229.WP_029061814
Labrenzia_sp_VG12.WP_094071057
Labrenzia_suaedae.WP_073011795
Polymorphum_gilvum.WP_013652532
Pannonibacter_phragmitetus.WP_050474704
Pannonibacter_phragmitetus.WP_019965804
Pannonibacter_indicus.WP_055456817
Labrenzia_alba.WP_055678293
Labrenzia_aggregata.WP_008936363
Labrenzia_aggregata.WP_077293602
Labrenzia_aggregata.WP_055656604
Labrenzia_sp_CP4.WP_062491579
Labrenzia_sp_C1B10.ERP97687
Labrenzia.WP_031268327
Mesorhizobium_sp_YR577.WP_091916737
Desulfatitalea_tepidiphila.WP_076750830
Desulfobacula_sp_GWF2_41_7.OGR15395
Gammaproteobacteria_bacterium_RIFCSPHIGHO2_12_FULL_41_15.OGT48867
CBU_1151
Bradyrhizobium_elkanii.WP_028163967
Bradyrhizobium_sp_Ai1a_2.WP_027581217
Bradyrhizobium_sp_Ec33.WP_027523014
Bradyrhizobium_stylosanthis.WP_063682974
Bradyrhizobium_japonicum.WP_018646928
Bradyrhizobium_sp_cf659.SFK13063
Bradyrhizobium_sp_cf659.WP_092193151
Bradyrhizobium_sp_TSA1.WP_100174658
Bradyrhizobium_sp_Rc2d.WP_091889541
Bradyrhizobium_yuanmingense.WP_036025601
Bradyrhizobium_sp_CCGE_LA001.WP_008548587
Bradyrhizobium_sp_DOA1.WP_061847763
Bradyrhizobium_arachidis.WP_092220146
Bradyrhizobium.WP_027559684
Bradyrhizobium_japonicum.WP_028155014
Bradyrhizobium_japonicum.WP_028169894
Bradyrhizobium_japonicum_USDA_6.BAL09832
Bradyrhizobium_japonicum.WP_039147690
candidate_division_KSB3_bacterium.PIE35630
Paenibacillus_odorifer.WP_076308546
Clostridium_homopropionicum.WP_052220536
Desulfosporosinus_acidiphilus.WP_014826806
Alkanindiges_illinoisensis.WP_084494859
Acinetobacter_nectaris.WP_023273652
Acinetobacter_apis.SNQ30235
Acinetobacter_apis.WP_088824403
Acinetobacter_boissieri.WP_092749490
Acinetobacter_pittii.WP_016144495
Acinetobacter_calcoaceticus.WP_045436646
Acinetobacter_indicus.WP_045795528
Acinetobacter_sp_CIP_5382.WP_005179868
Acinetobacter_indicus.WP_075175411
Acinetobacter_indicus.WP_075167991
Acinetobacter_indicus.WP_087662358
Pseudanabaena_biceps.WP_009625899
Tolypothrix_bouteillei.WP_038088401
Scytonema_hofmannii.WP_033335215
Anabaena_sp_WA102.WP_053540173
Sphaerospermopsis_kisseleviana.WP_096572452
Nostocaceae.WP_010996837
Nostoc_calcicola.WP_073639004
Nostoc.WP_067769439
Nostoc_sp_Peltigera_malacea_cyanobiont_DB3992.WP_099100782
Nostoc_sp_PCC_7107.WP_015112116
Nostoc_cycadae.WP_103125197
Anabaenopsis_circularis.WP_096581445
Calothrix_sp_NIES_2098.WP_096589730
Calothrix_sp_NIES_2100.WP_096607881
Nostocales.WP_096579697
Nostoc_carneum.WP_096731401
Nostocales.WP_045874526
Calothrix_brevissima_NIES_22.BAY64920
Calothrix_brevissima.WP_096647900
B
candidate_division_KSB3_bacterium
Bacteria
Firmicutes
Clostridiales
Paenibacillus_odorifer
Clostridium_homopropionicum
Desulfosporosinus_acidiphilus_SJ4
Cyanobacteria
Pseudanabaena_biceps_PCC_7429
Nostocales
Tolypothrix_bouteillei
Scytonema_hofmannii
Aphanizomenonaceae
Sphaerospermopsis_kisseleviana
Anabaenopsis_circularis
Calothrix
Calothrix_brevissima_NIES-22
Calothrix_sp_NIES-2100
Calothrix_sp_NIES-2098
Nostocaceae
Anabaena_sp_WA102
Nostoc
Nostoc_calcicola
Nostoc_cycadae
Nostoc_carneum
Nostoc_sp_Peltigera_malacea_cyanobiont_DB3992
Nostoc_sp_PCC_7107
Desulfobacteraceae
Desulfatitalea_tepidiphila
Desulfobacula_sp_GWF2_41_7
Gammaproteobacteria
Gammaproteobacteria_bacterium_RIFCSPHIGHO2_12_FULL_41_15
Coxiella_burnetii_RSA_493
Moraxellaceae
Alkanindiges_illinoisensis
Acinetobacter
Acinetobacter_indicus
Acinetobacter_nectaris
Acinetobacter_boissieri
Acinetobacter_apis
Acinetobacter_sp_CIP_5382
Acinetobacter_calcoaceticus
Acinetobacter_pittii
Proteobacteria
Polymorphum_gilvum
Pannonibacter
Pannonibacter_phragmitetus
Pannonibacter_indicus
Rhodobacteraceae
Labrenzia
Labrenzia_suaedae
Labrenzia_marina
Alphaproteobacteria
Labrenzia_aggregata
Labrenzia_alba
Labrenzia_sp_C1B10
Labrenzia_sp_CP4
Labrenzia_sp_DG1229
Labrenzia_sp_VG12
Labrenzia_sp_OB1
Rhizobiales
Mesorhizobium_sp_YR577
Bradyrhizobium
Bradyrhizobium_elkanii
Bradyrhizobium_stylosanthis
Bradyrhizobium_arachidis
Bradyrhizobium_japonicum_USDA_6
Bradyrhizobium_yuanmingense
Bradyrhizobium_sp_CCGE-LA001
Bradyrhizobium_sp_TSA1
Bradyrhizobium_sp_Ec33
Bradyrhizobium_sp_Ai1a-2
Bradyrhizobium_sp_DOA1
Bradyrhizobium_sp_Rc2d
Bradyrhizobium_sp_cf659

CBU_1127

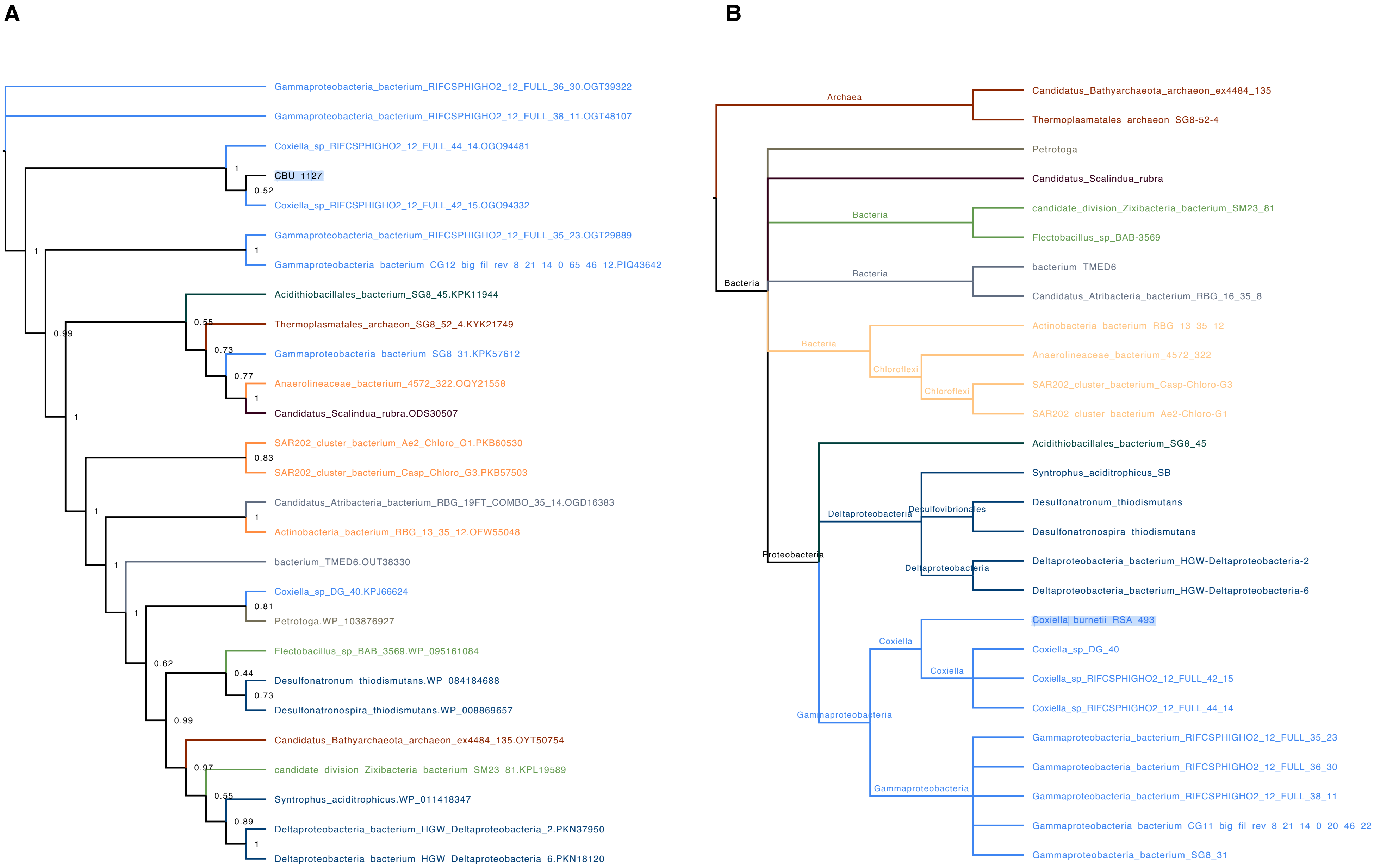
A
Gammaproteobacteria_bacterium_RIFCSPHIGHO2_12_FULL_36_30.OGT39322
Gammaproteobacteria_bacterium_RIFCSPHIGHO2_12_FULL_38_11.OGT48107
Coxiella_sp_RIFCSPHIGHO2_12_FULL_44_14.OGO94481
CBU_1127
Coxiella_sp_RIFCSPHIGHO2_12_FULL_42_15.OGO94332
Gammaproteobacteria_bacterium_RIFCSPHIGHO2_12_FULL_35_23.OGT29889
Gammaproteobacteria_bacterium_CG12_big_fil_rev_8_21_14_0_65_46_12.PIQ43642
Acidithiobacillales_bacterium_SG8_45.KPK11944
Thermoplasmatales_archaeon_SG8_52_4.KYK21749
Gammaproteobacteria_bacterium_SG8_31.KPK57612
Anaerolineaceae_bacterium_4572_322.OQY21558
Candidatus_Scalindua_rubra.ODS30507
SAR202_cluster_bacterium_Ae2_Chloro_G1.PKB60530
SAR202_cluster_bacterium_Casp_Chloro_G3.PKB57503
Candidatus_Atribacteria_bacterium_RBG_19FT_COMBO_35_14.OGD16383
Actinobacteria_bacterium_RBG_13_35_12.OFW55048
bacterium_TMED6.OUT38330
Coxiella_sp_DG_40.KPJ66624
Petrotoga.WP_103876927
Flectobacillus_sp_BAB_3569.WP_095161084
Desulfonatronum_thiodismutans.WP_084184688
Desulfonatronospira_thiodismutans.WP_008869657
Candidatus_Bathyarchaeota_archaeon_ex4484_135.OYT50754
candidate_division_Zixibacteria_bacterium_SM23_81.KPL19589
Syntrophus_aciditrophicus.WP_011418347
Deltaproteobacteria_bacterium_HGW_Deltaproteobacteria_2.PKN37950
Deltaproteobacteria_bacterium_HGW_Deltaproteobacteria_6.PKN18120
1
0.52
1
1
0.99
0.55
0.73
0.77
1
1
0.83
1
1
1
1
0.81
0.62
0.44
0.73
0.99
0.97
0.55
0.89
1
B
Archaea
Candidatus_Bathyarchaeota_archaeon_ex4484_135
Thermoplasmatales_archaeon_SG8-52-4
Petrotoga
Candidatus_Scalindua_rubra
Bacteria
candidate_division_Zixibacteria_bacterium_SM23_81
Flectobacillus_sp_BAB-3569
Bacteria
bacterium_TMED6
Candidatus_Atribacteria_bacterium_RBG_16_35_8
Bacteria
Bacteria
Actinobacteria_bacterium_RBG_13_35_12
Chloroflexi
Anaerolineaceae_bacterium_4572_322
Chloroflexi
SAR202_cluster_bacterium_Casp-Chloro-G3
SAR202_cluster_bacterium_Ae2-Chloro-G1
Acidithiobacillales_bacterium_SG8_45
Syntrophus_aciditrophicus_SB
Deltaproteobacteria
Desulfovibrionales
Desulfonatronum_thiodismutans
Desulfonatronospira_thiodismutans
Proteobacteria
Deltaproteobacteria
Deltaproteobacteria_bacterium_HGW-Deltaproteobacteria-2
Deltaproteobacteria_bacterium_HGW-Deltaproteobacteria-6
Coxiella_burnetii_RSA_493
Coxiella
Coxiella_sp_DG_40
Coxiella
Coxiella_sp_RIFCSPHIGHO2_12_FULL_42_15
Coxiella_sp_RIFCSPHIGHO2_12_FULL_44_14
Gammaproteobacteria
Gammaproteobacteria_bacterium_RIFCSPHIGHO2_12_FULL_35_23
Gammaproteobacteria_bacterium_RIFCSPHIGHO2_12_FULL_36_30
Gammaproteobacteria
Gammaproteobacteria_bacterium_RIFCSPHIGHO2_12_FULL_38_11
Gammaproteobacteria_bacterium_CG11_big_fil_rev_8_21_14_0_20_46_22
Gammaproteobacteria_bacterium_SG8_31

CBU_1112

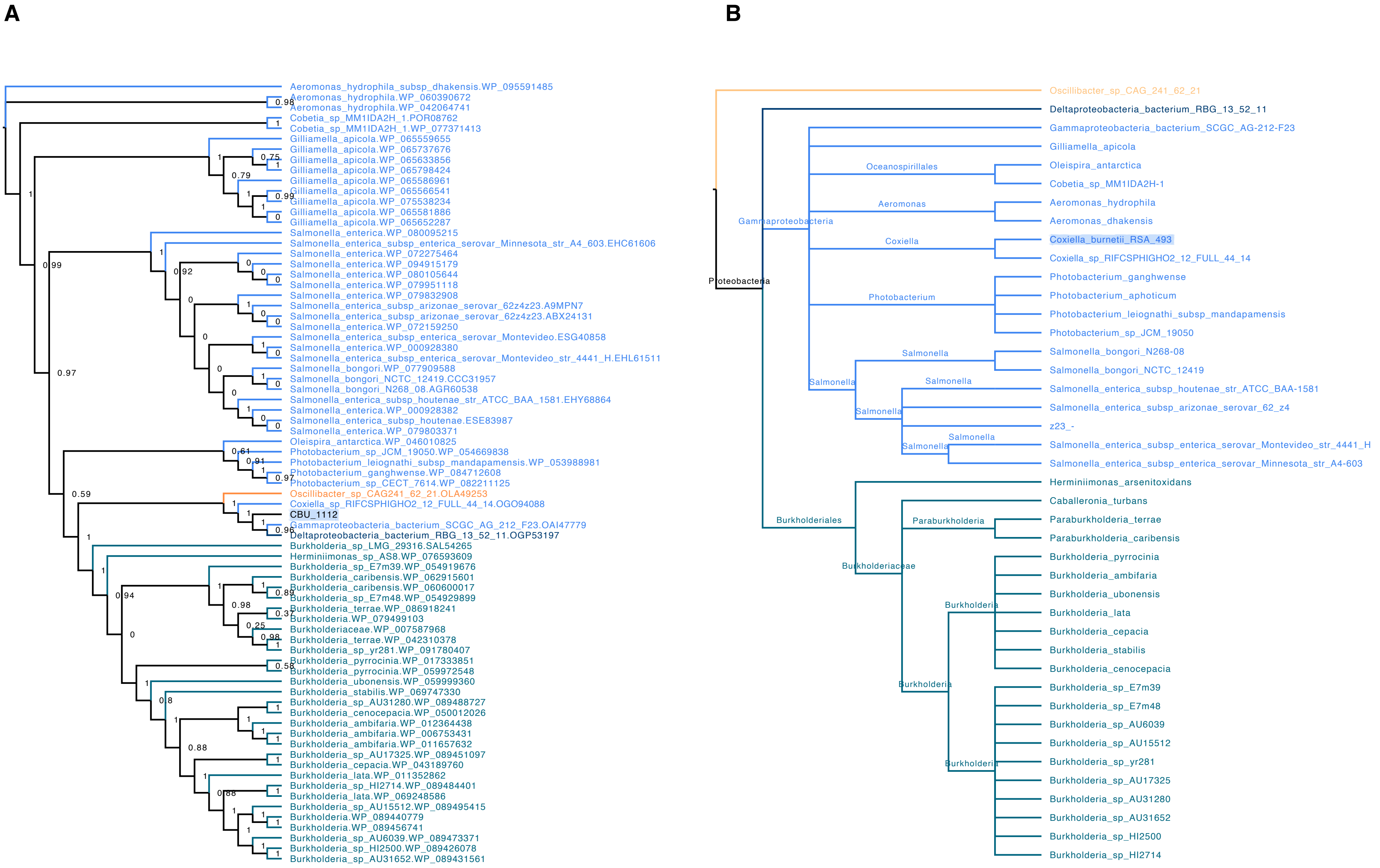
A
Aeromonas_hydrophila_subsp_dhakensis.WP_095591485
Aeromonas_hydrophila.WP_060390672
Aeromonas_hydrophila.WP_042064741
Cobetia_sp_MM1IDA2H_1.POR08762
Cobetia_sp_MM1IDA2H_1.WP_077371413
Gilliamella_apicola.WP_065559655
Gilliamella_apicola.WP_065737676
Gilliamella_apicola.WP_065633856
Gilliamella_apicola.WP_065798424
Gilliamella_apicola.WP_065586961
Gilliamella_apicola.WP_065566541
Gilliamella_apicola.WP_075538234
Gilliamella_apicola.WP_065581886
Gilliamella_apicola.WP_065652287
Salmonella_enterica.WP_080095215
Salmonella_enterica_subsp_enterica_serovar_Minnesota_str_A4_603.EHC61606
Salmonella_enterica.WP_072275464
Salmonella_enterica.WP_094915179
Salmonella_enterica.WP_080105644
Salmonella_enterica.WP_079951118
Salmonella_enterica.WP_079832908
Salmonella_enterica_subsp_arizonae_serovar_62z4z23.A9MPN7
Salmonella_enterica_subsp_arizonae_serovar_62z4z23.ABX24131
Salmonella_enterica.WP_072159250
Salmonella_enterica_subsp_enterica_serovar_Montevideo.ESG40858
Salmonella_enterica.WP_000928380
Salmonella_enterica_subsp_enterica_serovar_Montevideo_str_4441_H.EHL61511
Salmonella_bongori.WP_077909588
Salmonella_bongori_NCTC_12419.CCC31957
Salmonella_bongori_N268_08.AGR60538
Salmonella_enterica_subsp_houtenae_str_ATCC_BAA_1581.EHY68864
Salmonella_enterica.WP_000928382
Salmonella_enterica_subsp_houtenae.ESE83987
Salmonella_enterica.WP_079803371
Oleispira_antarctica.WP_046010825
Photobacterium_sp_JCM_19050.WP_054669838
Photobacterium_leiognathi_subsp_mandapamensis.WP_053988981
Photobacterium_ganghwense.WP_084712608
Photobacterium_sp_CECT_7614.WP_082211125
Oscillibacter_sp_CAG241_62_21.OLA49253
Coxiella_sp_RIFCSPHIGHO2_12_FULL_44_14.OGO94088
CBU_1112
Gammaproteobacteria_bacterium_SCGC_AG_212_F23.OAI47779
Deltaproteobacteria_bacterium_RBG_13_52_11.OGP53197
Burkholderia_sp_LMG_29316.SAL54265
Herminiimonas_sp_AS8.WP_076593609
Burkholderia_sp_E7m39.WP_054919676
Burkholderia_caribensis.WP_062915601
Burkholderia_caribensis.WP_060600017
Burkholderia_sp_E7m48.WP_054929899
Burkholderia_terrae.WP_086918241
Burkholderia.WP_079499103
Burkholderiaceae.WP_007587968
Burkholderia_terrae.WP_042310378
Burkholderia_sp_yr281.WP_091780407
Burkholderia_pyrrocinia.WP_017333851
Burkholderia_pyrrocinia.WP_059972548
Burkholderia_ubonensis.WP_059999360
Burkholderia_stabilis.WP_069747330
Burkholderia_sp_AU31280.WP_089488727
Burkholderia_cenocepacia.WP_050012026
Burkholderia_ambifaria.WP_012364438
Burkholderia_ambifaria.WP_006753431
Burkholderia_ambifaria.WP_011657632
Burkholderia_sp_AU17325.WP_089451097
Burkholderia_cepacia.WP_043189760
Burkholderia_lata.WP_011352862
Burkholderia_sp_HI2714.WP_089484401
Burkholderia_lata.WP_069248586
Burkholderia_sp_AU15512.WP_089495415
Burkholderia.WP_089440779
Burkholderia.WP_089456741
Burkholderia_sp_AU6039.WP_089473371
Burkholderia_sp_HI2500.WP_089426078
Burkholderia_sp_AU31652.WP_089431561
B
Oscillibacter_sp_CAG_241_62_21
Deltaproteobacteria_bacterium_RBG_13_52_11
Gammaproteobacteria_bacterium_SCGC_AG-212-F23
Gilliamella_apicola
Oceanospirillales
Oleispira_antarctica
Cobetia_sp_MM1IDA2H-1
Aeromonas
Aeromonas_hydrophila
Aeromonas_dhakensis
Gammaproteobacteria
Coxiella
Coxiella_burnetii_RSA_493
Coxiella_sp_RIFCSPHIGHO2_12_FULL_44_14
Proteobacteria
Photobacterium
Photobacterium_ganghwense
Photobacterium_aphoticum
Photobacterium_leiognathi_subsp_mandapamensis
Photobacterium_sp_JCM_19050
Salmonella
Salmonella_bongori_N268-08
Salmonella_bongori_NCTC_12419
Salmonella_enterica_subsp_houtenae_str_ATCC_BAA-1581
Salmonella_enterica_subsp_arizonae_serovar_62_z4
z23_-
Salmonella_enterica_subsp_enterica_serovar_Montevideo_str_4441_H
Salmonella_enterica_subsp_enterica_serovar_Minnesota_str_A4-603
Herminiimonas_arsenitoxidans
Caballeronia_turbans
Burkholderiales
Paraburkholderia
Paraburkholderia_terrae
Paraburkholderia_caribensis
Burkholderiaceae
Burkholderia_pyrrocinia
Burkholderia_ambifaria
Burkholderia_ubonensis
Burkholderia
Burkholderia_lata
Burkholderia_cepacia
Burkholderia_stabilis
Burkholderia_cenocepacia
Burkholderia_sp_E7m39
Burkholderia_sp_E7m48
Burkholderia_sp_AU6039
Burkholderia_sp_AU15512
Burkholderia_sp_yr281
Burkholderia_sp_AU17325
Burkholderia_sp_AU31280
Burkholderia_sp_AU31652
Burkholderia_sp_HI2500
Burkholderia_sp_HI2714

CBU_1097

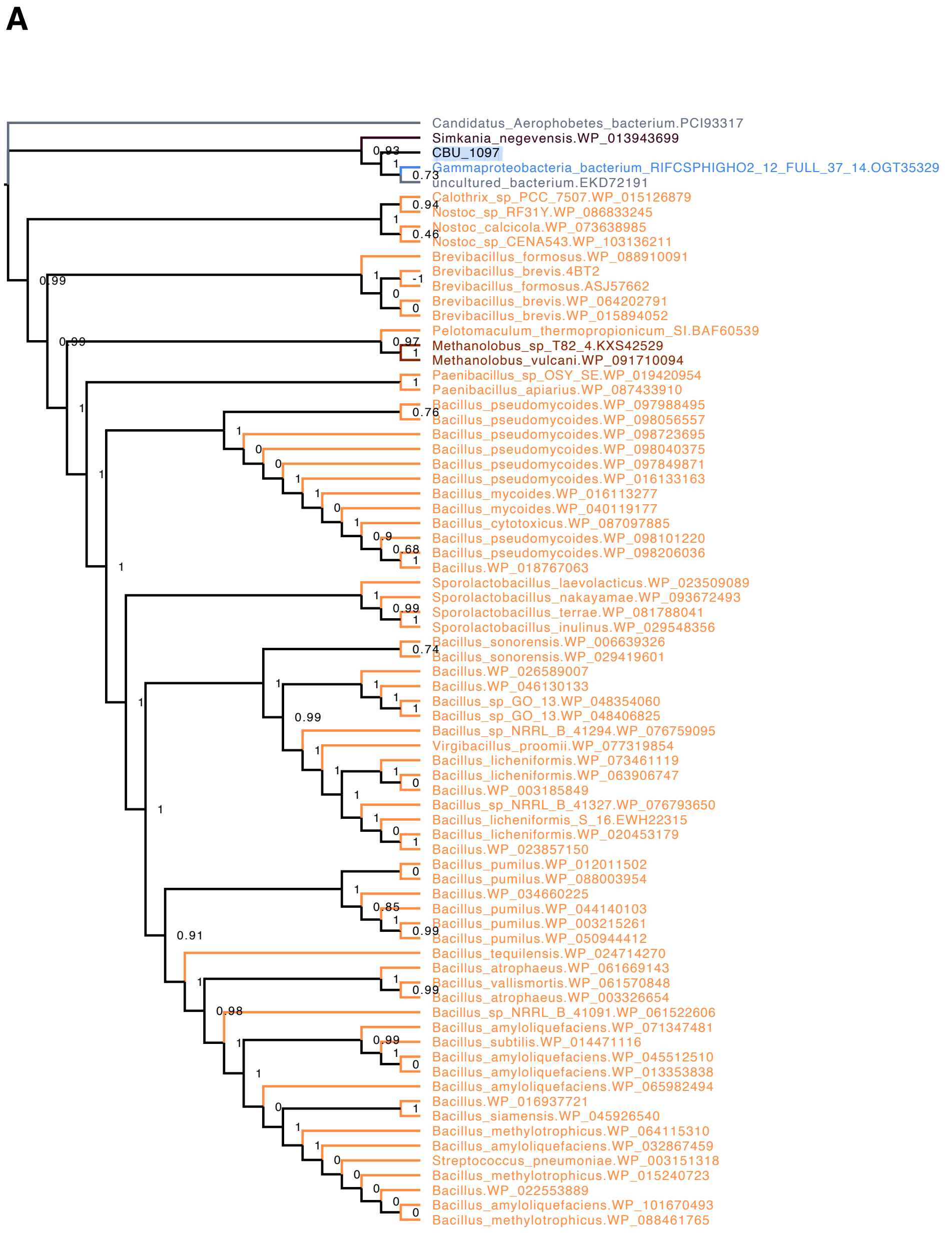
A
Candidatus_Aerophobetes_bacterium.PCI93317
Simkania_negevensis.WP_013943699
CBU_1097
Gammaproteobacteria_bacterium_RIFCSPHIGHO2_12_FULL_37_14.OGT35329
uncultured_bacterium.EKD72191
Calothrix_sp_PCC_7507.WP_015126879
Nostoc_sp_RF31Y.WP_086833245
Nostoc_calcicola.WP_073638985
Nostoc_sp_CENA543.WP_103136211
Brevibacillus_formosus.WP_088910091
Brevibacillus_brevis.4BT2
Brevibacillus_formosus.ASJ57662
Brevibacillus_brevis.WP_064202791
Brevibacillus_brevis.WP_015894052
Pelotomaculum_thermopropionicum_SI.BAF60539
Methanolobus_sp_T82_4.KXS42529
Methanolobus_vulcani.WP_091710094
Paenibacillus_sp_OSY_SE.WP_019420954
Paenibacillus_apiarius.WP_087433910
Bacillus_pseudomycoides.WP_097988495
Bacillus_pseudomycoides.WP_098056557
Bacillus_pseudomycoides.WP_098723695
Bacillus_pseudomycoides.WP_098040375
Bacillus_pseudomycoides.WP_097849871
Bacillus_pseudomycoides.WP_016133163
Bacillus_mycoides.WP_016113277
Bacillus_mycoides.WP_040119177
Bacillus_cytotoxicus.WP_087097885
Bacillus_pseudomycoides.WP_098101220
Bacillus_pseudomycoides.WP_098206036
Bacillus.WP_018767063
Sporolactobacillus_laevolacticus.WP_023509089
Sporolactobacillus_nakayamae.WP_093672493
Sporolactobacillus_terrae.WP_081788041
Sporolactobacillus_inulinus.WP_029548356
Bacillus_sonorensis.WP_006639326
Bacillus_sonorensis.WP_029419601
Bacillus.WP_026589007
Bacillus.WP_046130133
Bacillus_sp_GO_13.WP_048354060
Bacillus_sp_GO_13.WP_048406825
Bacillus_sp_NRRL_B_41294.WP_076759095
Virgibacillus_proomii.WP_077319854
Bacillus_licheniformis.WP_073461119
Bacillus_licheniformis.WP_063906747
Bacillus.WP_003185849
Bacillus_sp_NRRL_B_41327.WP_076793650
Bacillus_licheniformis_S_16.EWH22315
Bacillus_licheniformis.WP_020453179
Bacillus.WP_023857150
Bacillus_pumilus.WP_012011502
Bacillus_pumilus.WP_088003954
Bacillus.WP_034660225
Bacillus_pumilus.WP_044140103
Bacillus_pumilus.WP_003215261
Bacillus_pumilus.WP_050944412
Bacillus_tequilensis.WP_024714270
Bacillus_atrophaeus.WP_061669143
Bacillus_vallismortis.WP_061570848
Bacillus_atrophaeus.WP_003326654
Bacillus_sp_NRRL_B_41091.WP_061522606
Bacillus_amyloliquefaciens.WP_071347481
Bacillus_subtilis.WP_014471116
Bacillus_amyloliquefaciens.WP_045512510
Bacillus_amyloliquefaciens.WP_013353838
Bacillus_amyloliquefaciens.WP_065982494
Bacillus.WP_016937721
Bacillus_siamensis.WP_045926540
Bacillus_methylotrophicus.WP_064115310
Bacillus_amyloliquefaciens.WP_032867459
Streptococcus_pneumoniae.WP_003151318
Bacillus_methylotrophicus.WP_015240723
Bacillus.WP_022553889
Bacillus_amyloliquefaciens.WP_101670493
Bacillus_methylotrophicus.WP_088461765

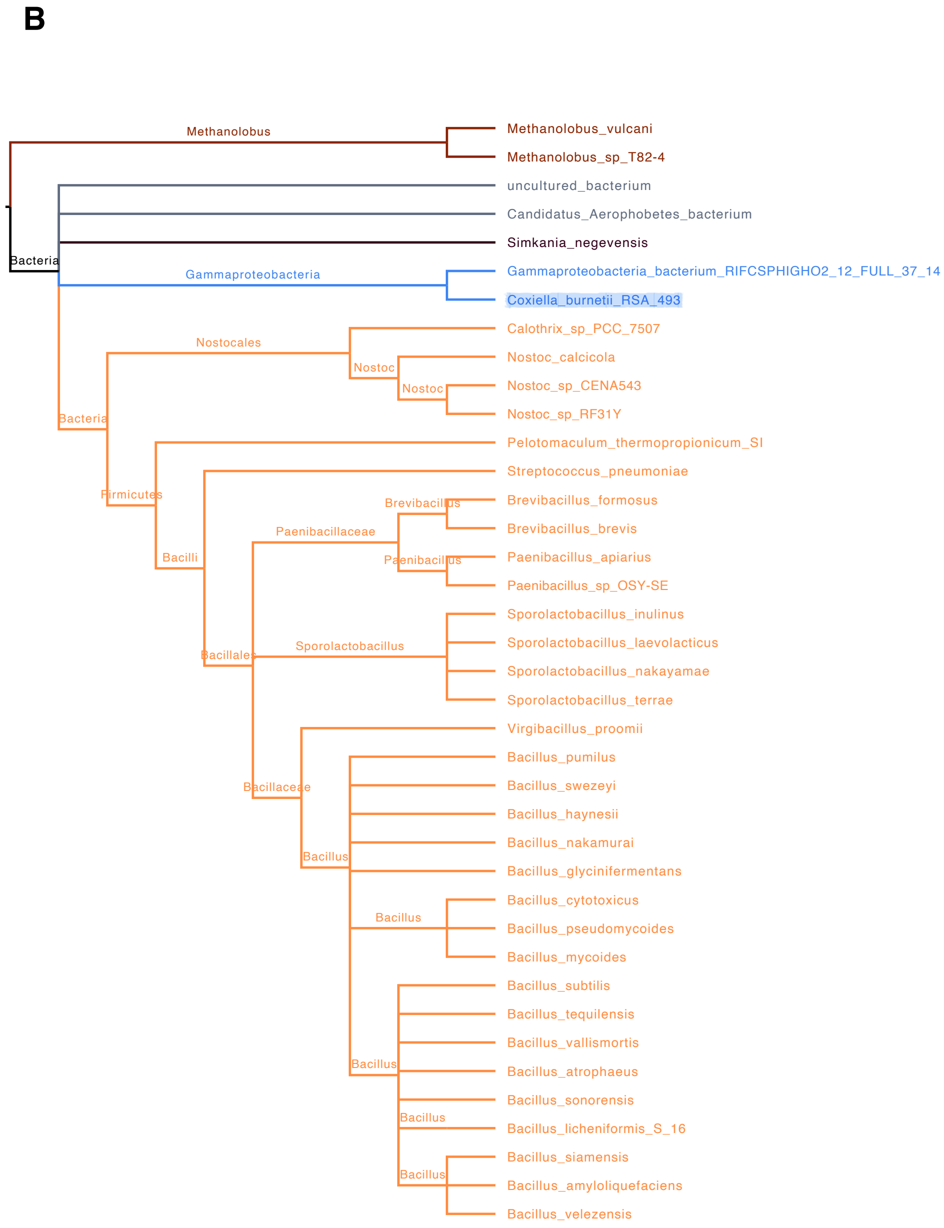
B
Methanolobus
Methanolobus_vulcani
Methanolobus_sp_T82-4
uncultured_bacterium
Candidatus_Aerophobetes_bacterium
Simkania_negevensis
Bacteria
Gammaproteobacteria
Gammaproteobacteria_bacterium_RIFCSPHIGHO2_12_FULL_37_14
Coxiella_burnetii_RSA_493
Nostocales
Calothrix_sp_PCC_7507
Nostoc
Nostoc_calcicola
Nostoc_sp_CENA543
Nostoc_sp_RF31Y
Pelotomaculum_thermopropionicum_SI
Firmicutes
Streptococcus_pneumoniae
Brevibacillus
Brevibacillus_formosus
Brevibacillus_brevis
Paenibacillaceae
Paenibacillus
Paenibacillus_apiarius
Paenibacillus_sp_OSY-SE
Bacilli
Sporolactobacillus
Sporolactobacillus_inulinus
Sporolactobacillus_laevolacticus
Sporolactobacillus_nakayamae
Sporolactobacillus_terrae
Bacillales
Virgibacillus_proomii
Bacillaceae
Bacillus_pumilus
Bacillus_swezeyi
Bacillus_haynesii
Bacillus_nakamurai
Bacillus_glycinifermentans
Bacillus
Bacillus_cytotoxicus
Bacillus_pseudomycoides
Bacillus_mycoides
Bacillus_subtilis
Bacillus_tequilensis
Bacillus_vallismortis
Bacillus_atrophaeus
Bacillus_sonorensis
Bacillus_licheniformis_S_16
Bacillus_siamensis
Bacillus_amyloliquefaciens
Bacillus_velezensis

CBU_1071

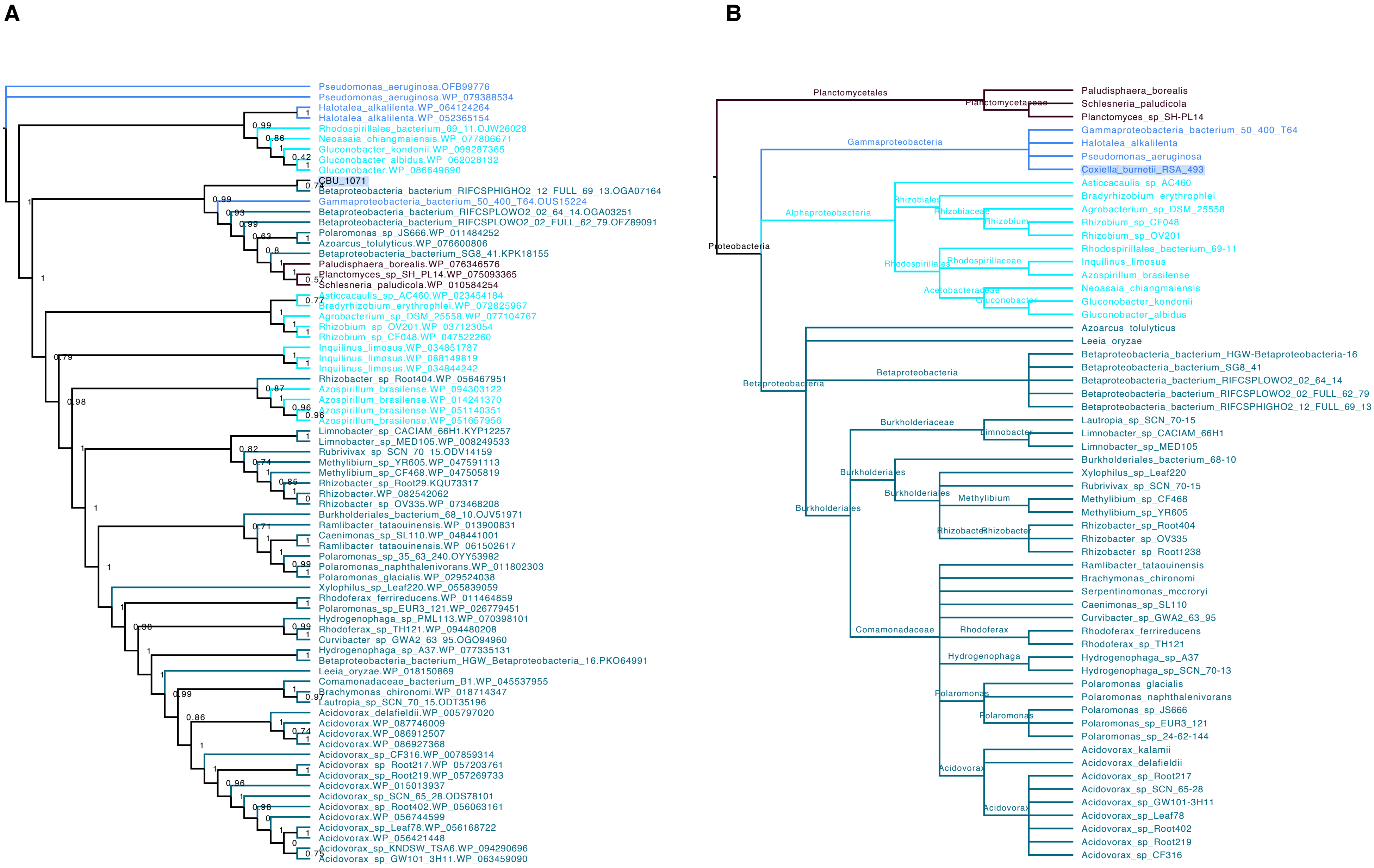
A
Pseudomonas_aeruginosa.OFB99776
Pseudomonas_aeruginosa.WP_079388534
Halotalea_alkalilenta.WP_064124264
Halotalea_alkalilenta.WP_052365154
Rhodospirillales_bacterium_69_11.OJW26028
Neoasaia_chiangmaiensis.WP_077806671
Gluconobacter_kondonii.WP_099287365
Gluconobacter_albidus.WP_062028132
Gluconobacter.WP_086649690
CBU_1071
Betaproteobacteria_bacterium_RIFCSPHIGHO2_12_FULL_69_13.OGA07164
Gammaproteobacteria_bacterium_50_400_T64.OUS15224
Betaproteobacteria_bacterium_RIFCSPLOWO2_02_64_14.OGA03251
Betaproteobacteria_bacterium_RIFCSPLOWO2_02_FULL_62_79.OFZ89091
Polaromonas_sp_JS666.WP_011484252
Azoarcus_tolulyticus.WP_076600806
Betaproteobacteria_bacterium_SG8_41.KPK18155
Paludisphaera_borealis.WP_076346576
Planctomyces_sp_SH_PL14.WP_075093365
Schlesneria_paludicola.WP_010584254
Asticcacaulis_sp_AC460.WP_023454184
Bradyrhizobium_erythrophlei.WP_072825967
Agrobacterium_sp_DSM_25558.WP_077104767
Rhizobium_sp_OV201.WP_037123054
Rhizobium_sp_CF048.WP_047522260
Inquilinus_limosus.WP_034851787
Inquilinus_limosus.WP_088149819
Inquilinus_limosus.WP_034844242
Rhizobacter_sp_Root404.WP_056467951
Azospirillum_brasilense.WP_094303122
Azospirillum_brasilense.WP_014241370
Azospirillum_brasilense.WP_051140351
Azospirillum_brasilense.WP_051657956
Limnobacter_sp_CACIAM_66H1.KYP12257
Limnobacter_sp_MED105.WP_008249533
Rubrivivax_sp_SCN_70_15.ODV14159
Methylibium_sp_YR605.WP_047591113
Methylibium_sp_CF468.WP_047505819
Rhizobacter_sp_Root29.KQU73317
Rhizobacter.WP_082542062
Rhizobacter_sp_OV335.WP_073468208
Burkholderiales_bacterium_68_10.OJV51971
Ramlibacter_tataouinensis.WP_013900831
Caenimonas_sp_SL110.WP_048441001
Ramlibacter_tataouinensis.WP_061502617
Polaromonas_sp_35_63_240.OYY53982
Polaromonas_naphthalenivorans.WP_011802303
Polaromonas_glacialis.WP_029524038
Xylophilus_sp_Leaf220.WP_055839059
Rhodoferax_ferrireducens.WP_011464859
Polaromonas_sp_EUR3_121.WP_026779451
Hydrogenophaga_sp_PML113.WP_070398101
Rhodoferax_sp_TH121.WP_094480208
Curvibacter_sp_GWA2_63_95.OGO94960
Hydrogenophaga_sp_A37.WP_077335131
Betaproteobacteria_bacterium_HGW_Betaproteobacteria_16.PKO64991
Leeia_oryzae.WP_018150869
Comamonadaceae_bacterium_B1.WP_045537955
Brachymonas_chironomi.WP_018714347
Lautropia_sp_SCN_70_15.ODT35196
Acidovorax_delafieldii.WP_005797020
Acidovorax.WP_087746009
Acidovorax.WP_086912507
Acidovorax.WP_086927368
Acidovorax_sp_CF316.WP_007859314
Acidovorax_sp_Root217.WP_057203761
Acidovorax_sp_Root219.WP_057269733
Acidovorax.WP_015013937
Acidovorax_sp_SCN_65_28.ODS78101
Acidovorax_sp_Root402.WP_056063161
Acidovorax.WP_056744599
Acidovorax_sp_Leaf78.WP_056168722
Acidovorax.WP_056421448
Acidovorax_sp_KNDSW_TSA6.WP_094290696
Acidovorax_sp_GW101_3H11.WP_063459090
B
Planctomycetales
Planctomycetaceae
Paludisphaera_borealis
Schlesneria_paludicola
Planctomyces_sp_SH-PL14
Gammaproteobacteria
Gammaproteobacteria_bacterium_50_400_T64
Halotalea_alkalilenta
Pseudomonas_aeruginosa
Coxiella_burnetii_RSA_493
Proteobacteria
Alphaproteobacteria
Rhizobiales
Rhizobiaceae
Rhizobium
Rhodospirillales
Rhodospirillaceae
Acetobacteraceae
Gluconobacter
Asticcacaulis_sp_AC460
Bradyrhizobium_erythrophlei
Agrobacterium_sp_DSM_25558
Rhizobium_sp_CF048
Rhizobium_sp_OV201
Rhodospirillales_bacterium_69-11
Inquilinus_limosus
Azospirillum_brasilense
Neoasaia_chiangmaiensis
Gluconobacter_kondonii
Gluconobacter_albidus
Betaproteobacteria
Azoarcus_tolulyticus
Leeia_oryzae
Betaproteobacteria_bacterium_HGW-Betaproteobacteria-16
Betaproteobacteria_bacterium_SG8_41
Betaproteobacteria_bacterium_RIFCSPLOWO2_02_64_14
Betaproteobacteria_bacterium_RIFCSPLOWO2_02_FULL_62_79
Betaproteobacteria_bacterium_RIFCSPHIGHO2_12_FULL_69_13
Burkholderiales
Burkholderiaceae
Limnobacter
Methylibium
Rhizobacter
Comamonadaceae
Rhodoferax
Hydrogenophaga
Polaromonas
Acidovorax
Lautropia_sp_SCN_70-15
Limnobacter_sp_CACIAM_66H1
Limnobacter_sp_MED105
Burkholderiales_bacterium_68-10
Xylophilus_sp_Leaf220
Rubrivivax_sp_SCN_70-15
Methylibium_sp_CF468
Methylibium_sp_YR605
Rhizobacter_sp_Root404
Rhizobacter_sp_OV335
Rhizobacter_sp_Root1238
Ramlibacter_tataouinensis
Brachymonas_chironomi
Serpentinomonas_mccroryi
Caenimonas_sp_SL110
Curvibacter_sp_GWA2_63_95
Rhodoferax_ferrireducens
Rhodoferax_sp_TH121
Hydrogenophaga_sp_A37
Hydrogenophaga_sp_SCN_70-13
Polaromonas_glacialis
Polaromonas_naphthalenivorans
Polaromonas_sp_JS666
Polaromonas_sp_EUR3_121
Polaromonas_sp_24-62-144
Acidovorax_kalamii
Acidovorax_delafieldii
Acidovorax_sp_Root217
Acidovorax_sp_SCN_65-28
Acidovorax_sp_GW101-3H11
Acidovorax_sp_Leaf78
Acidovorax_sp_Root402
Acidovorax_sp_Root219
Acidovorax_sp_CF316

CBU_1065

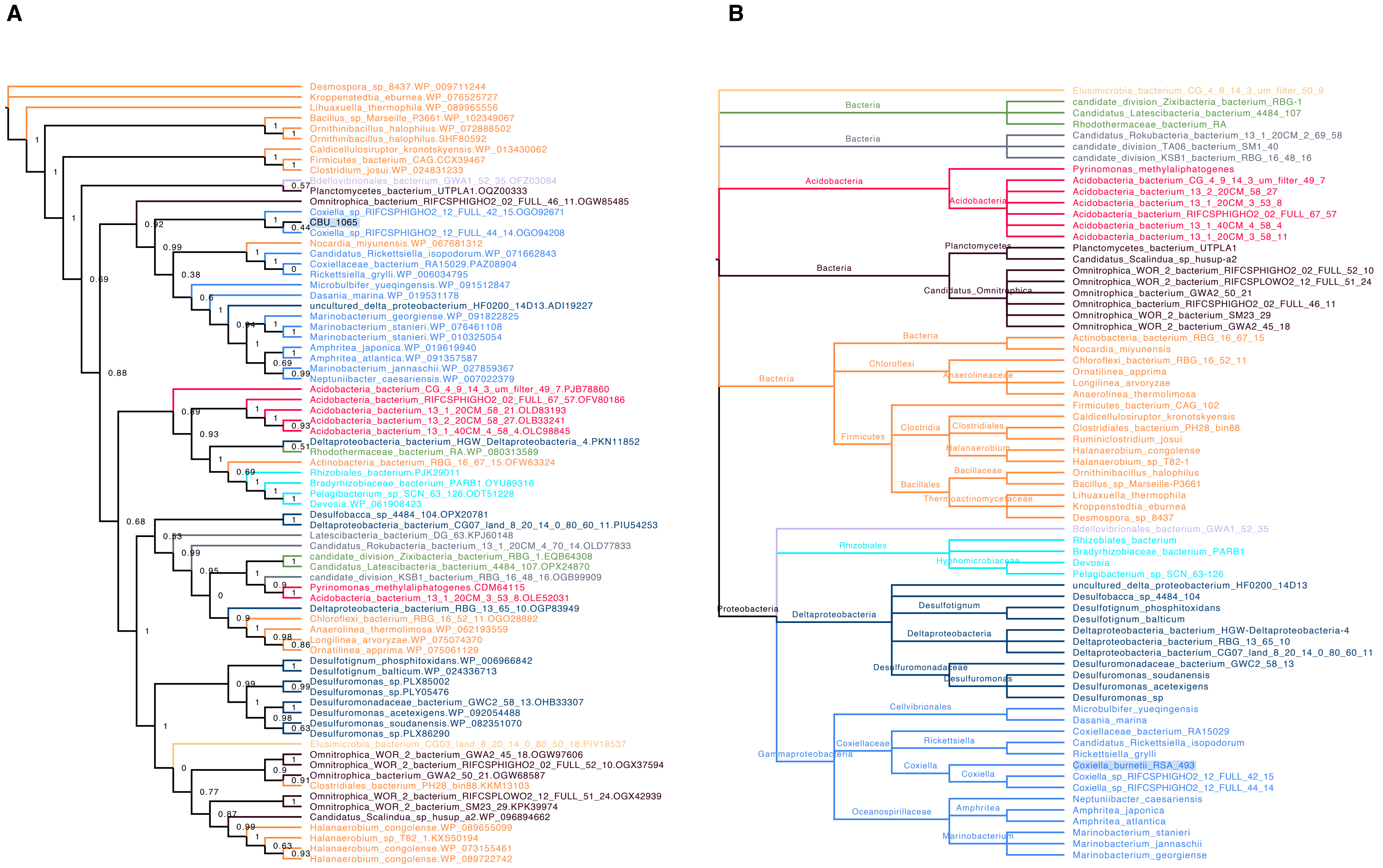
A
Desmospora_sp_8437.WP_009711244
Kroppenstedtia_eburnea.WP_076525727
Lihuaxuella_thermophila.WP_089965556
Bacillus_sp_Marseille_P3661.WP_102349067
Ornithinibacillus_halophilus.WP_072888502
Ornithinibacillus_halophilus.SHF80592
Caldicellulosiruptor_kronotskyensis.WP_013430062
Firmicutes_bacterium_CAG.CCX39467
Clostridium_josui.WP_024831233
Bdellovibrionales_bacterium_GWA1_52_35.OFZ03084
Planctomycetes_bacterium_UTPLA1.OQZ00333
Omnitrophica_bacterium_RIFCSPHIGHO2_02_FULL_46_11.OGW85485
Coxiella_sp_RIFCSPHIGHO2_12_FULL_42_15.OGO92671
CBU_1065
Coxiella_sp_RIFCSPHIGHO2_12_FULL_44_14.OGO94208
Nocardia_miyunensis.WP_067681312
Candidatus_Rickettsiella_isopodorum.WP_071662843
Coxiellaceae_bacterium_RA15029.PAZ08904
Rickettsiella_grylli.WP_006034795
Microbulbifer_yueqingensis.WP_091512847
Dasania_marina.WP_019531178
uncultured_delta_proteobacterium_HF0200_14D13.ADI19227
Marinobacterium_georgiense.WP_091822825
Marinobacterium_stanieri.WP_076461108
Marinobacterium_stanieri.WP_010325054
Amphritea_japonica.WP_019619940
Amphritea_atlantica.WP_091357587
Marinobacterium_jannaschii.WP_027859367
Neptuniibacter_caesariensis.WP_007022379
Acidobacteria_bacterium_CG_4_9_14_3_um_filter_49_7.PJB78860
Acidobacteria_bacterium_RIFCSPHIGHO2_02_FULL_67_57.OFV80186
Acidobacteria_bacterium_13_1_20CM_58_21.OLD83193
Acidobacteria_bacterium_13_2_20CM_58_27.OLB33241
Acidobacteria_bacterium_13_1_40CM_4_58_4.OLC98845
Deltaproteobacteria_bacterium_HGW_Deltaproteobacteria_4.PKN11852
Rhodothermaceae_bacterium_RA.WP_080313589
Actinobacteria_bacterium_RBG_16_67_15.OFW63324
Rhizobiales_bacterium.PJK29011
Bradyrhizobiaceae_bacterium_PARB1.OYU89316
Pelagibacterium_sp_SCN_63_126.ODT51228
Devosia.WP_061908423
Desulfobacca_sp_4484_104.OPX20781
Deltaproteobacteria_bacterium_CG07_land_8_20_14_0_80_60_11.PIU54253
Latescibacteria_bacterium_DG_63.KPJ60148
Candidatus_Rokubacteria_bacterium_13_1_20CM_4_70_14.OLD77833
candidate_division_Zixibacteria_bacterium_RBG_1.EQB64308
Candidatus_Latescibacteria_bacterium_4484_107.OPX24870
candidate_division_KSB1_bacterium_RBG_16_48_16.OGB99909
Pyrinomonas_methylaliphatogenes.CDM64115
Acidobacteria_bacterium_13_1_20CM_3_53_8.OLE52031
Deltaproteobacteria_bacterium_RBG_13_65_10.OGP83949
Chloroflexi_bacterium_RBG_16_52_11.OGO28882
Anaerolinea_thermolimosa.WP_062193559
Longilinea_arvoryzae.WP_075074370
Ornatilinea_apprima.WP_075061129
Desulfotignum_phosphitoxidans.WP_006966842
Desulfotignum_balticum.WP_024336713
Desulfuromonas_sp.PLX85002
Desulfuromonas_sp.PLY05476
Desulfuromonadaceae_bacterium_GWC2_58_13.OHB33307
Desulfuromonas_acetexigens.WP_092054488
Desulfuromonas_soudanensis.WP_082351070
Desulfuromonas_sp.PLX86290
Elusimicrobia_bacterium_CG03_land_8_20_14_0_80_50_18.PIV18537
Omnitrophica_WOR_2_bacterium_GWA2_45_18.OGW97606
Omnitrophica_WOR_2_bacterium_RIFCSPHIGHO2_02_FULL_52_10.OGX37594
Omnitrophica_bacterium_GWA2_50_21.OGW68587
Clostridiales_bacterium_PH28_bin88.KKM13103
Omnitrophica_WOR_2_bacterium_RIFCSPLOWO2_12_FULL_51_24.OGX42939
Omnitrophica_WOR_2_bacterium_SM23_29.KPK39974
Candidatus_Scalindua_sp_husup_a2.WP_096894662
Halanaerobium_congolense.WP_089655099
Halanaerobium_sp_T82_1.KXS50194
Halanaerobium_congolense.WP_073155461
Halanaerobium_congolense.WP_089722742
B
Elusimicrobia_bacterium_CG_4_8_14_3_um_filter_50_9
Bacteria
candidate_division_Zixibacteria_bacterium_RBG-1
Candidatus_Latescibacteria_bacterium_4484_107
Rhodothermaceae_bacterium_RA
Bacteria
Candidatus_Rokubacteria_bacterium_13_1_20CM_2_69_58
candidate_division_TA06_bacterium_SM1_40
candidate_division_KSB1_bacterium_RBG_16_48_16
Acidobacteria
Pyrinomonas_methylaliphatogenes
Acidobacteria
Acidobacteria_bacterium_CG_4_9_14_3_um_filter_49_7
Acidobacteria_bacterium_13_2_20CM_58_27
Acidobacteria_bacterium_13_1_20CM_3_53_8
Acidobacteria_bacterium_RIFCSPHIGHO2_02_FULL_67_57
Acidobacteria_bacterium_13_1_40CM_4_58_4
Acidobacteria_bacterium_13_1_20CM_3_58_11
Planctomycetes
Planctomycetes_bacterium_UTPLA1
Candidatus_Scalindua_sp_husup-a2
Bacteria
Candidatus_Omnitrophica
Omnitrophica_WOR_2_bacterium_RIFCSPHIGHO2_02_FULL_52_10
Omnitrophica_WOR_2_bacterium_RIFCSPLOWO2_12_FULL_51_24
Omnitrophica_bacterium_GWA2_50_21
Omnitrophica_bacterium_RIFCSPHIGHO2_02_FULL_46_11
Omnitrophica_WOR_2_bacterium_SM23_29
Omnitrophica_WOR_2_bacterium_GWA2_45_18
Bacteria
Actinobacteria_bacterium_RBG_16_67_15
Nocardia_miyunensis
Bacteria
Chloroflexi
Anaerolineaceae
Chloroflexi_bacterium_RBG_16_52_11
Ornatilinea_apprima
Longilinea_arvoryzae
Anaerolinea_thermolimosa
Firmicutes_bacterium_CAG_102
Firmicutes
Clostridia
Clostridiales
Caldicellulosiruptor_kronotskyensis
Clostridiales_bacterium_PH28_bin88
Ruminiclostridium_josui
Halanaerobium
Halanaerobium_congolense
Halanaerobium_sp_T82-1
Bacillales
Bacillaceae
Ornithinibacillus_halophilus
Bacillus_sp_Marseille-P3661
Thermoactinomycetaceae
Lihuaxuella_thermophila
Kroppenstedtia_eburnea
Desmospora_sp_8437
Bdellovibrionales_bacterium_GWA1_52_35
Rhizobiales
Rhizobiales_bacterium
Hyphomicrobiaceae
Bradyrhizobiaceae_bacterium_PARB1
Devosia
Pelagibacterium_sp_SCN_63-126
uncultured_delta_proteobacterium_HF0200_14D13
Proteobacteria
Deltaproteobacteria
Desulfotignum
Desulfobacca_sp_4484_104
Desulfotignum_phosphitoxidans
Desulfotignum_balticum
Deltaproteobacteria
Deltaproteobacteria_bacterium_HGW-Deltaproteobacteria-4
Deltaproteobacteria_bacterium_RBG_13_65_10
Deltaproteobacteria_bacterium_CG07_land_8_20_14_0_80_60_11
Desulfuromonadaceae
Desulfuromonas
Desulfuromonadaceae_bacterium_GWC2_58_13
Desulfuromonas_soudanensis
Desulfuromonas_acetexigens
Desulfuromonas_sp
Cellvibrionales
Microbulbifer_yueqingensis
Dasania_marina
Coxiellaceae
Rickettsiella
Coxiellaceae_bacterium_RA15029
Candidatus_Rickettsiella_isopodorum
Rickettsiella_grylli
Gammaproteobacteria
Coxiella
Coxiella
Coxiella_burnetii_RSA_493
Coxiella_sp_RIFCSPHIGHO2_12_FULL_42_15
Coxiella_sp_RIFCSPHIGHO2_12_FULL_44_14
Oceanospirillaceae
Amphritea
Neptuniibacter_caesariensis
Amphritea_japonica
Amphritea_atlantica
Marinobacterium
Marinobacterium_stanieri
Marinobacterium_jannaschii
Marinobacterium_georgiense

CBU_1031

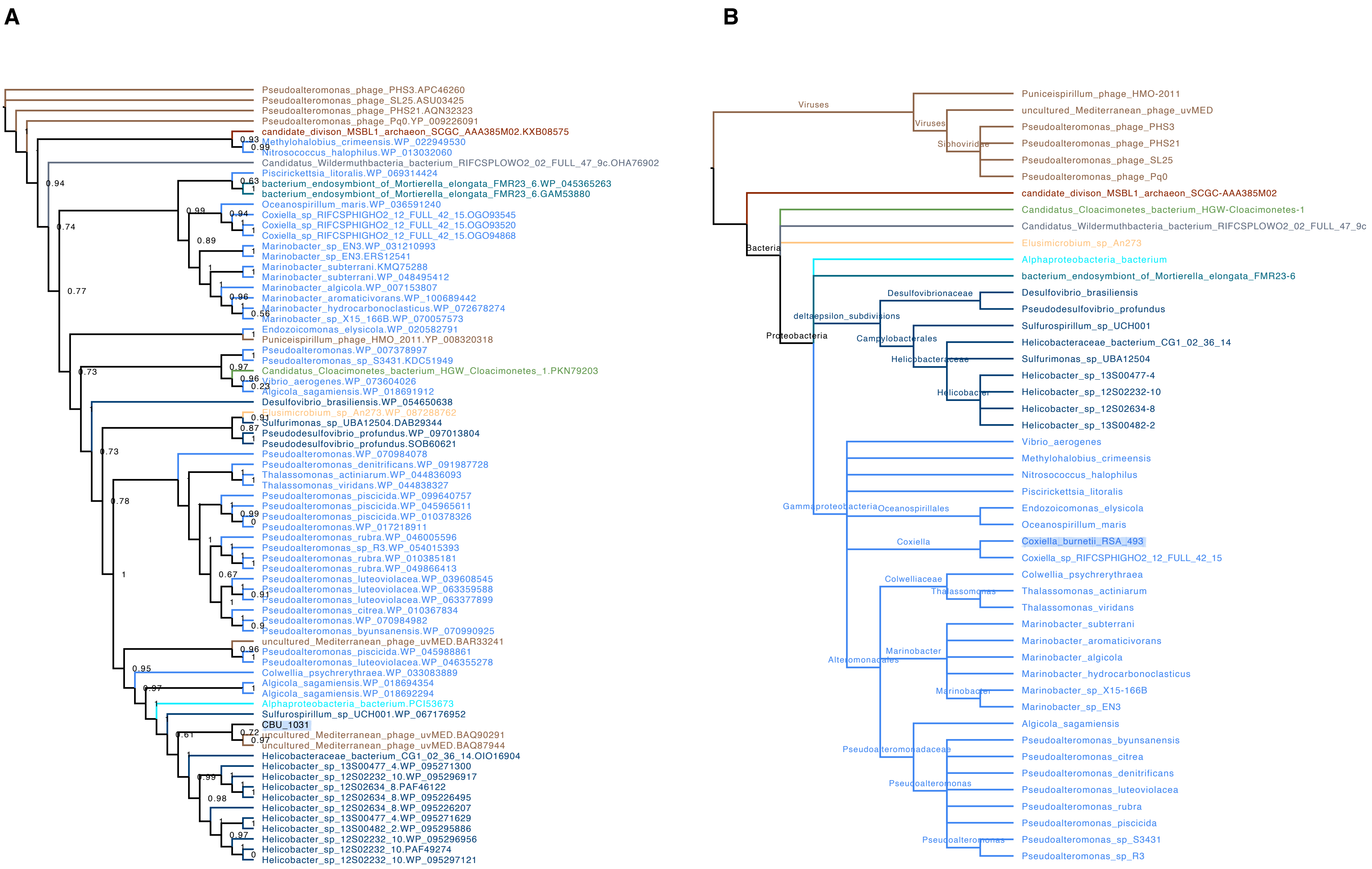
A
Pseudoalteromonas_phage_PHS3.APC46260
Pseudoalteromonas_phage_SL25.ASU03425
Pseudoalteromonas_phage_PHS21.AQN32323
Pseudoalteromonas_phage_Pq0.YP_009226091
candidate_divison_MSBL1_archaeon_SCGC_AAA385M02.KXB08575
Methylohalobius_crimeensis.WP_022949530
Nitrosococcus_halophilus.WP_013032060
Candidatus_Wildermuthbacteria_bacterium_RIFCSPLOWO2_02_FULL_47_9c.OHA76902
Piscirickettsia_litoralis.WP_069314424
bacterium_endosymbiont_of_Mortierella_elongata_FMR23_6.WP_045365263
bacterium_endosymbiont_of_Mortierella_elongata_FMR23_6.GAM53880
Oceanospirillum_maris.WP_036591240
Coxiella_sp_RIFCSPHIGHO2_12_FULL_42_15.OGO93545
Coxiella_sp_RIFCSPHIGHO2_12_FULL_42_15.OGO93520
Coxiella_sp_RIFCSPHIGHO2_12_FULL_42_15.OGO94868
Marinobacter_sp_EN3.WP_031210993
Marinobacter_sp_EN3.ERS12541
Marinobacter_subterrani.KMQ75288
Marinobacter_subterrani.WP_048495412
Marinobacter_algicola.WP_007153807
Marinobacter_aromaticivorans.WP_100689442
Marinobacter_hydrocarbonoclasticus.WP_072678274
Marinobacter_sp_X15_166B.WP_070057573
Endozoicomonas_elysicola.WP_020582791
Puniceispirillum_phage_HMO_2011.YP_008320318
Pseudoalteromonas.WP_007378997
Pseudoalteromonas_sp_S3431.KDC51949
Candidatus_Cloacimonetes_bacterium_HGW_Cloacimonetes_1.PKN79203
Vibrio_aerogenes.WP_073604026
Algicola_sagamiensis.WP_018691912
Desulfovibrio_brasiliensis.WP_054650638
Elusimicrobium_sp_An273.WP_087288762
Sulfurimonas_sp_UBA12504.DAB29344
Pseudodesulfovibrio_profundus.WP_097013804
Pseudodesulfovibrio_profundus.SOB60621
Pseudoalteromonas.WP_070984078
Pseudoalteromonas_denitrificans.WP_091987728
Thalassomonas_actiniarum.WP_044836093
Thalassomonas_viridans.WP_044838327
Pseudoalteromonas_piscicida.WP_099640757
Pseudoalteromonas_piscicida.WP_045965611
Pseudoalteromonas_piscicida.WP_010378326
Pseudoalteromonas.WP_017218911
Pseudoalteromonas_rubra.WP_046005596
Pseudoalteromonas_sp_R3.WP_054015393
Pseudoalteromonas_rubra.WP_010385181
Pseudoalteromonas_rubra.WP_049866413
Pseudoalteromonas_luteoviolacea.WP_039608545
Pseudoalteromonas_luteoviolacea.WP_063359588
Pseudoalteromonas_luteoviolacea.WP_063377899
Pseudoalteromonas_citrea.WP_010367834
Pseudoalteromonas.WP_070984982
Pseudoalteromonas_byunsanensis.WP_070990925
uncultured_Mediterranean_phage_uvMED.BAR33241
Pseudoalteromonas_piscicida.WP_045988861
Pseudoalteromonas_luteoviolacea.WP_046355278
Colwellia_psychrerythraea.WP_033083889
Algicola_sagamiensis.WP_018694354
Algicola_sagamiensis.WP_018692294
Alphaproteobacteria_bacterium.PCI53673
Sulfurospirillum_sp_UCH001.WP_067176952
CBU_1031
uncultured_Mediterranean_phage_uvMED.BAQ90291
uncultured_Mediterranean_phage_uvMED.BAQ87944
Helicobacteraceae_bacterium_CG1_02_36_14.OIO16904
Helicobacter_sp_13S00477_4.WP_095271300
Helicobacter_sp_12S02232_10.WP_095296917
Helicobacter_sp_12S02634_8.PAF46122
Helicobacter_sp_12S02634_8.WP_095226495
Helicobacter_sp_12S02634_8.WP_095226207
Helicobacter_sp_13S00477_4.WP_095271629
Helicobacter_sp_13S00482_2.WP_095295886
Helicobacter_sp_12S02232_10.WP_095296956
Helicobacter_sp_12S02232_10.PAF49274
Helicobacter_sp_12S02232_10.WP_095297121
B
Viruses
Siphoviridae
Bacteria
Proteobacteria
deltaepsilon_subdivisions
Desulfovibrionaceae
Campylobacterales
Helicobacteraceae
Helicobacter
Gammaproteobacteria
Oceanospirillales
Coxiella
Colwelliaceae
Thalassomonas
Alteromonadales
Marinobacter
Pseudoalteromonadaceae
Pseudoalteromonas
Puniceispirillum_phage_HMO-2011
uncultured_Mediterranean_phage_uvMED
Pseudoalteromonas_phage_PHS3
Pseudoalteromonas_phage_PHS21
Pseudoalteromonas_phage_SL25
Pseudoalteromonas_phage_Pq0
candidate_divison_MSBL1_archaeon_SCGC-AAA385M02
Candidatus_Cloacimonetes_bacterium_HGW-Cloacimonetes-1
Candidatus_Wildermuthbacteria_bacterium_RIFCSPLOWO2_02_FULL_47_9c
Elusimicrobium_sp_An273
Alphaproteobacteria_bacterium
bacterium_endosymbiont_of_Mortierella_elongata_FMR23-6
Desulfovibrio_brasiliensis
Pseudodesulfovibrio_profundus
Sulfurospirillum_sp_UCH001
Helicobacteraceae_bacterium_CG1_02_36_14
Sulfurimonas_sp_UBA12504
Helicobacter_sp_13S00477-4
Helicobacter_sp_12S02232-10
Helicobacter_sp_12S02634-8
Helicobacter_sp_13S00482-2
Vibrio_aerogenes
Methylohalobius_crimeensis
Nitrosococcus_halophilus
Piscirickettsia_litoralis
Endozoicomonas_elysicola
Oceanospirillum_maris
Coxiella_burnetii_RSA_493
Coxiella_sp_RIFCSPHIGHO2_12_FULL_42_15
Colwellia_psychrerythraea
Thalassomonas_actiniarum
Thalassomonas_viridans
Marinobacter_subterrani
Marinobacter_aromaticivorans
Marinobacter_algicola
Marinobacter_hydrocarbonoclasticus
Marinobacter_sp_X15-166B
Marinobacter_sp_EN3
Algicola_sagamiensis
Pseudoalteromonas_byunsanensis
Pseudoalteromonas_citrea
Pseudoalteromonas_denitrificans
Pseudoalteromonas_luteoviolacea
Pseudoalteromonas_rubra
Pseudoalteromonas_piscicida
Pseudoalteromonas_sp_S3431
Pseudoalteromonas_sp_R3

CBU_1028

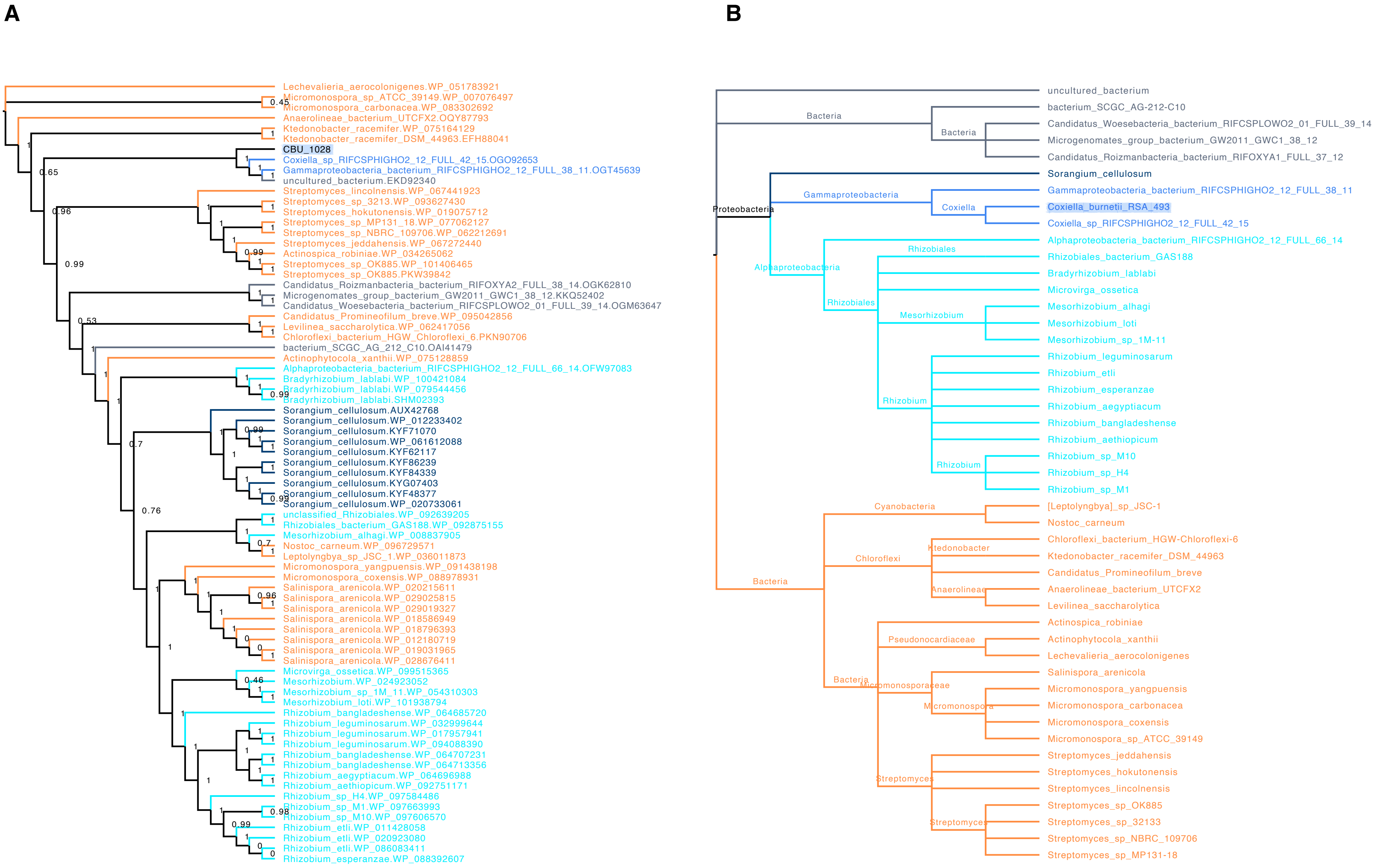
A
Lechevalieria_aerocolonigenes.WP_051783921
Micromonospora_sp_ATCC_39149.WP_007076497
Micromonospora_carbonacea.WP_083302692
Anaerolineae_bacterium_UTCFX2.OQY87793
Ktedonobacter_racemifer.WP_075164129
Ktedonobacter_racemifer_DSM_44963.EFH88041
CBU_1028
Coxiella_sp_RIFCSPHIGHO2_12_FULL_42_15.OGO92653
Gammaproteobacteria_bacterium_RIFCSPHIGHO2_12_FULL_38_11.OGT45639
uncultured_bacterium.EKD92340
Streptomyces_lincolnensis.WP_067441923
Streptomyces_sp_3213.WP_093627430
Streptomyces_hokutonensis.WP_019075712
Streptomyces_sp_MP131_18.WP_077062127
Streptomyces_sp_NBRC_109706.WP_062212691
Streptomyces_jeddahensis.WP_067272440
Actinospica_robiniae.WP_034265062
Streptomyces_sp_OK885.WP_101406465
Streptomyces_sp_OK885.PKW39842
Candidatus_Roizmanbacteria_bacterium_RIFOXYA2_FULL_38_14.OGK62810
Microgenomates_group_bacterium_GW2011_GWC1_38_12.KKQ52402
Candidatus_Woesebacteria_bacterium_RIFCSPLOWO2_01_FULL_39_14.OGM63647
Candidatus_Promineofilum_breve.WP_095042856
Levilinea_saccharolytica.WP_062417056
Chloroflexi_bacterium_HGW_Chloroflexi_6.PKN90706
bacterium_SCGC_AG_212_C10.OAI41479
Actinophytocola_xanthii.WP_075128859
Alphaproteobacteria_bacterium_RIFCSPHIGHO2_12_FULL_66_14.OFW97083
Bradyrhizobium_lablabi.WP_100421084
Bradyrhizobium_lablabi.WP_079544456
Bradyrhizobium_lablabi.SHM02393
Sorangium_cellulosum.AUX42768
Sorangium_cellulosum.WP_012233402
Sorangium_cellulosum.KYF71070
Sorangium_cellulosum.WP_061612088
Sorangium_cellulosum.KYF62117
Sorangium_cellulosum.KYF86239
Sorangium_cellulosum.KYF84339
Sorangium_cellulosum.KYG07403
Sorangium_cellulosum.KYF48377
Sorangium_cellulosum.WP_020733061
unclassified_Rhizobiales.WP_092639205
Rhizobiales_bacterium_GAS188.WP_092875155
Mesorhizobium_alhagi.WP_008837905
Nostoc_carneum.WP_096729519
Leptolyngbya_sp_JSC_1.WP_036011873
Micromonospora_yangpuensis.WP_091438198
Micromonospora_coxensis.WP_088978931
Salinispora_arenicola.WP_020215611
Salinispora_arenicola.WP_029025815
Salinispora_arenicola.WP_029019327
Salinispora_arenicola.WP_018586949
Salinispora_arenicola.WP_018796393
Salinispora_arenicola.WP_012180719
Salinispora_arenicola.WP_019031965
Salinispora_arenicola.WP_028676411
Microvirga_ossetica.WP_099515365
Mesorhizobium.WP_024923052
Mesorhizobium_sp_1M_11.WP_054310303
Mesorhizobium_loti.WP_101938794
Rhizobium_bangladeshense.WP_064685720
Rhizobium_leguminosarum.WP_032999644
Rhizobium_leguminosarum.WP_017957941
Rhizobium_leguminosarum.WP_094088390
Rhizobium_bangladeshense.WP_064707231
Rhizobium_bangladeshense.WP_064713356
Rhizobium_aegyptiacum.WP_064696988
Rhizobium_aethiopicum.WP_092751171
Rhizobium_sp_H4.WP_097584486
Rhizobium_sp_M1.WP_097663993
Rhizobium_sp_M10.WP_097606570
Rhizobium_etli.WP_011428058
Rhizobium_etli.WP_020923080
Rhizobium_etli.WP_086083411
Rhizobium_esperanzae.WP_088392607
B
uncultured_bacterium
Bacteria
bacterium_SCGC_AG-212-C10
Bacteria
Candidatus_Woesebacteria_bacterium_RIFCSPLOWO2_01_FULL_39_14
Microgenomates_group_bacterium_GW2011_GWC1_38_12
Candidatus_Roizmanbacteria_bacterium_RIFOXYA1_FULL_37_12
Sorangium_cellulosum
Gammaproteobacteria
Gammaproteobacteria_bacterium_RIFCSPHIGHO2_12_FULL_38_11
Proteobacteria
Coxiella
Coxiella_burnetii_RSA_493
Coxiella_sp_RIFCSPHIGHO2_12_FULL_42_15
Alphaproteobacteria_bacterium_RIFCSPHIGHO2_12_FULL_66_14
Rhizobiales
Rhizobiales_bacterium_GAS188
Alphaproteobacteria
Bradyrhizobium_lablabi
Microvirga_ossetica
Rhizobiales
Mesorhizobium_alhagi
Mesorhizobium
Mesorhizobium_loti
Mesorhizobium_sp_1M-11
Rhizobium_leguminosarum
Rhizobium_etli
Rhizobium_esperanzae
Rhizobium
Rhizobium_aegyptiacum
Rhizobium_bangladeshense
Rhizobium_aethiopicum
Rhizobium_sp_M10
Rhizobium
Rhizobium_sp_H4
Rhizobium_sp_M1
Cyanobacteria
[Leptolyngbya]_sp_JSC-1
Nostoc_carneum
Chloroflexi_bacterium_HGW-Chloroflexi-6
Ktedonobacter
Chloroflexi
Ktedonobacter_racemifer_DSM_44963
Bacteria
Candidatus_Promineofilum_breve
Anaerolineae
Anaerolineae_bacterium_UTCFX2
Levilinea_saccharolytica
Actinospica_robiniae
Pseudonocardiaceae
Actinophytocola_xanthii
Lechevalieria_aerocolonigenes
Salinispora_arenicola
Bacteria
Micromonosporaceae
Micromonospora_yangpuensis
Micromonospora
Micromonospora_carbonacea
Micromonospora_coxensis
Micromonospora_sp_ATCC_39149
Streptomyces_jeddahensis
Streptomyces_hokutonensis
Streptomyces
Streptomyces_lincolnensis
Streptomyces_sp_OK885
Streptomyces
Streptomyces_sp_32133
Streptomyces_sp_NBRC_109706
Streptomyces_sp_MP131-18

CBU_1027

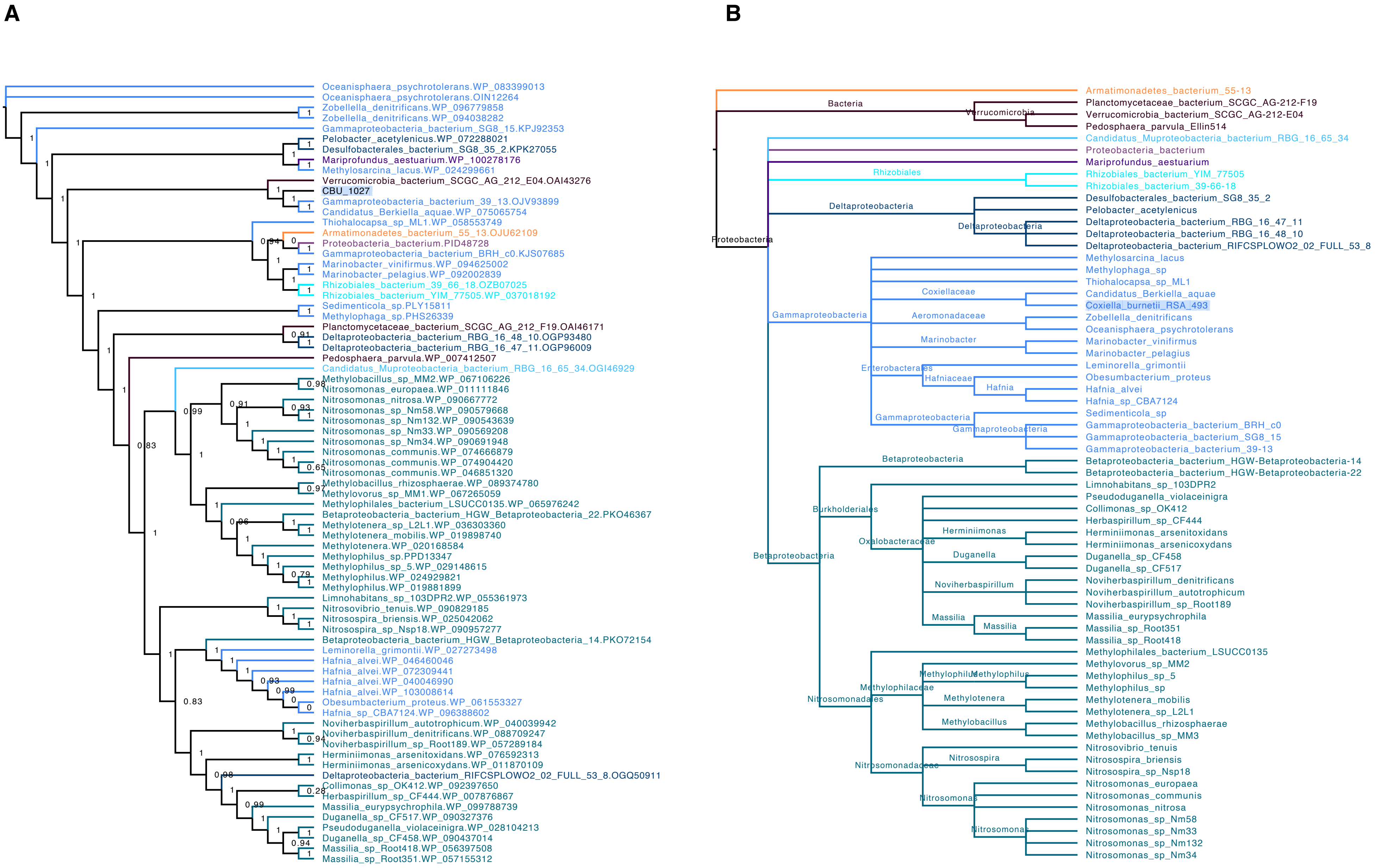

A
Oceanisphaera_psychrotolerans.WP_083399013
Oceanisphaera_psychrotolerans.OIN12264
Zobellella_denitrificans.WP_096779858
Zobellella_denitrificans.WP_094038282
Gammaproteobacteria_bacterium_SG8_15.KPJ92353
Pelobacter_acetylenicus.WP_072288021
Desulfobacterales_bacterium_SG8_35_2.KPK27055
Mariprofundus_aestuarium.WP_100278176
Methylosarcina_lacus.WP_024299661
Verrucomicrobia_bacterium_SCGC_AG_212_E04.OAI43276
CBU_1027
Gammaproteobacteria_bacterium_39_13.OJV93899
Candidatus_Berkiella_aquae.WP_075065754
Thiohalocapsa_sp_ML1.WP_058553749
Armatimonadetes_bacterium_55_13.OJU62109
Proteobacteria_bacterium.PID48728
Gammaproteobacteria_bacterium_BRH_c0.KJS07685
Marinobacter_vinifirmus.WP_094625002
Marinobacter_pelagius.WP_092002839
Rhizobiales_bacterium_39_66_18.OZB07025
Rhizobiales_bacterium_YIM_77505.WP_037018192
Sedimenticola_sp.PLY15811
Methylophaga_sp.PHS26339
Planctomycetaceae_bacterium_SCGC_AG_212_F19.OAI46171
Deltaproteobacteria_bacterium_RBG_16_48_10.OGP93480
Deltaproteobacteria_bacterium_RBG_16_47_11.OGP96009
Pedosphaera_parvula.WP_007412507
Candidatus_Muproteobacteria_bacterium_RBG_16_65_34.OGI46929
Methylobacillus_sp_MM2.WP_067106226
Nitrosomonas_europaea.WP_011111846
Nitrosomonas_nitrosa.WP_090667772
Nitrosomonas_sp_Nm58.WP_090579668
Nitrosomonas_sp_Nm132.WP_090543639
Nitrosomonas_sp_Nm33.WP_090569208
Nitrosomonas_sp_Nm34.WP_090691948
Nitrosomonas_communis.WP_074666879
Nitrosomonas_communis.WP_074904420
Nitrosomonas_communis.WP_046851320
Methylobacillus_rhizosphaerae.WP_089374780
Methylovorus_sp_MM1.WP_067265059
Methylophilales_bacterium_LSUCC0135.WP_065976242
Betaproteobacteria_bacterium_HGW_Betaproteobacteria_22.PKO46367
Methylotenera_sp_L2L1.WP_036303360
Methylotenera_mobilis.WP_019898740
Methylotenera.WP_020168584
Methylophilus_sp.PPD13347
Methylophilus_sp_5.WP_029148615
Methylophilus.WP_024929821
Methylophilus.WP_019881899
Limnohabitans_sp_103DPR2.WP_055361973
Nitrosovibrio_tenuis.WP_090829185
Nitrosospira_briensis.WP_025042062
Nitrosospira_sp_Nsp18.WP_090957277
Betaproteobacteria_bacterium_HGW_Betaproteobacteria_14.PKO72154
Leminorella_grimontii.WP_027273498
Hafnia_alvei.WP_046460046
Hafnia_alvei.WP_072309441
Hafnia_alvei.WP_040046990
Hafnia_alvei.WP_103008614
Obesumbacterium_proteus.WP_061553327
Hafnia_sp_CBA7124.WP_096388602
Noviherbaspirillum_autotrophicum.WP_040039942
Noviherbaspirillum_denitrificans.WP_088709247
Noviherbaspirillum_sp_Root189.WP_057289184
Herminiimonas_arsenitoxidans.WP_076592313
Herminiimonas_arsenicoxydans.WP_011870109
Deltaproteobacteria_bacterium_RIFCSPLOWO2_02_FULL_53_8.OGQ50911
Collimonas_sp_OK412.WP_092397650
Herbaspirillum_sp_CF444.WP_007876867
Massilia_eurypsychrophila.WP_099788739
Duganella_sp_CF517.WP_090327376
Pseudoduganella_violaceinigra.WP_028104213
Duganella_sp_CF458.WP_090437014
Massilia_sp_Root418.WP_056397508
Massilia_sp_Root351.WP_057155312
B
Armatimonadetes_bacterium_55-13
Bacteria
Verrucomicrobia
Planctomycetaceae_bacterium_SCGC_AG-212-F19
Verrucomicrobia_bacterium_SCGC_AG-212-E04
Pedosphaera_parvula_Ellin514
Candidatus_Muproteobacteria_bacterium_RBG_16_65_34
Proteobacteria_bacterium
Mariprofundus_aestuarium
Rhizobiales
Rhizobiales_bacterium_YIM_77505
Rhizobiales_bacterium_39-66-18
Deltaproteobacteria
Desulfobacterales_bacterium_SG8_35_2
Pelobacter_acetylenicus
Deltaproteobacteria
Deltaproteobacteria_bacterium_RBG_16_47_11
Proteobacteria
Deltaproteobacteria_bacterium_RBG_16_48_10
Deltaproteobacteria_bacterium_RIFCSPLOWO2_02_FULL_53_8
Methylosarcina_lacus
Methylophaga_sp
Thiohalocapsa_sp_ML1
Coxiellaceae
Candidatus_Berkiella_aquae
Coxiella_burnetii_RSA_493
Gammaproteobacteria
Aeromonadaceae
Zobellella_denitrificans
Oceanisphaera_psychrotolerans
Marinobacter
Marinobacter_vinifirmus
Marinobacter_pelagius
Enterobacterales
Leminorella_grimontii
Hafniaceae
Obesumbacterium_proteus
Hafnia
Hafnia_alvei
Hafnia_sp_CBA7124
Gammaproteobacteria
Sedimenticola_sp
Gammaproteobacteria_bacterium_BRH_c0
Gammaproteobacteria
Gammaproteobacteria_bacterium_SG8_15
Gammaproteobacteria_bacterium_39-13
Betaproteobacteria
Betaproteobacteria_bacterium_HGW-Betaproteobacteria-14
Betaproteobacteria_bacterium_HGW-Betaproteobacteria-22
Limnohabitans_sp_103DPR2
Pseudoduganella_violaceinigra
Burkholderiales
Collimonas_sp_OK412
Herbaspirillum_sp_CF444
Herminiimonas
Herminiimonas_arsenitoxidans
Oxalobacteraceae
Herminiimonas_arsenicoxydans
Betaproteobacteria
Duganella
Duganella_sp_CF458
Duganella_sp_CF517
Noviherbaspirillum
Noviherbaspirillum_denitrificans
Noviherbaspirillum_autotrophicum
Noviherbaspirillum_sp_Root189
Massilia
Massilia_eurypsychrophila
Massilia
Massilia_sp_Root351
Massilia_sp_Root418
Methylophilales_bacterium_LSUCC0135
Methylovorus_sp_MM2
Methylophilus
Methylophilus
Methylophilus_sp_5
Methylophilaceae
Methylophilus_sp
Nitrosomonadales
Methylotenera
Methylotenera_mobilis
Methylotenera_sp_L2L1
Methylobacillus
Methylobacillus_rhizosphaerae
Methylobacillus_sp_MM3
Nitrosovibrio_tenuis
Nitrosospira
Nitrosospira_briensis
Nitrosomonadaceae
Nitrosospira_sp_Nsp18
Nitrosomonas_europaea
Nitrosomonas
Nitrosomonas_communis
Nitrosomonas_nitrosa
Nitrosomonas_sp_Nm58
Nitrosomonas
Nitrosomonas_sp_Nm33
Nitrosomonas_sp_Nm132
Nitrosomonas_sp_Nm34

CBU_1023

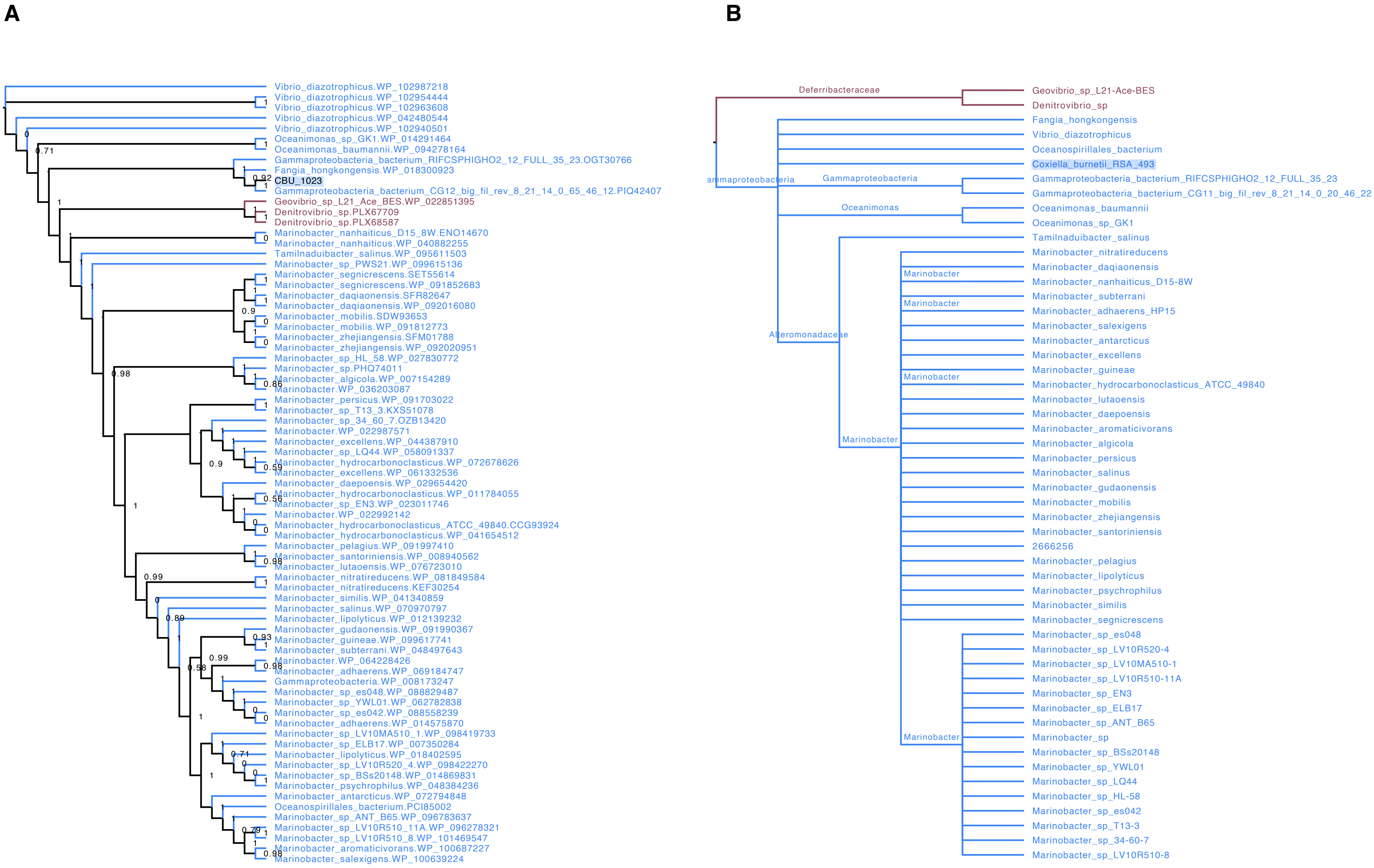

A
Vibrio_diazotrophicus.WP_102987218
Vibrio_diazotrophicus.WP_102954444
Vibrio_diazotrophicus.WP_102963608
Vibrio_diazotrophicus.WP_042480544
Vibrio_diazotrophicus.WP_102940501
Oceanimonas_sp_GK1.WP_014291464
Oceanimonas_baumannii.WP_094278164
Gammaproteobacteria_bacterium_RIFCSPHIGHO2_12_FULL_35_23.OGT30766
Fangia_hongkongensis.WP_018300923
CBU_1023
Gammaproteobacteria_bacterium_CG12_big_fil_rev_8_21_14_0_65_46_12.PIQ42407
Geovibrio_sp_L21_Ace_BES.WP_022851395
Denitrovibrio_sp.PLX67709
Denitrovibrio_sp.PLX68587
Marinobacter_nanhaiticus_D15_8W.ENO14670
Marinobacter_nanhaiticus.WP_040882255
Tamilnaduibacter_salinus.WP_095611503
Marinobacter_sp_PWS21.WP_099615136
Marinobacter_segnicrescens.SET55614
Marinobacter_segnicrescens.WP_091852683
Marinobacter_daqiaonensis.SFR82647
Marinobacter_daqiaonensis.WP_092016080
Marinobacter_mobilis.SDW93653
Marinobacter_mobilis.WP_091812773
Marinobacter_zhejiangensis.SFM01788
Marinobacter_zhejiangensis.WP_092020951
Marinobacter_sp_HL_58.WP_027830772
Marinobacter_sp.PHQ74011
Marinobacter_algicola.WP_007154289
Marinobacter.WP_036203087
Marinobacter_persicus.WP_091703022
Marinobacter_sp_T13_3.KXS51078
Marinobacter_sp_34_60_7.OZB13420
Marinobacter.WP_022987571
Marinobacter_excellens.WP_044387910
Marinobacter_sp_LQ44.WP_058091337
Marinobacter_hydrocarbonoclasticus.WP_072678626
Marinobacter_excellens.WP_061332536
Marinobacter_daepoensis.WP_029654420
Marinobacter_hydrocarbonoclasticus.WP_011784055
Marinobacter_sp_EN3.WP_023011746
Marinobacter.WP_022992142
Marinobacter_hydrocarbonoclasticus_ATCC_49840.CCG93924
Marinobacter_hydrocarbonoclasticus.WP_041654512
Marinobacter_pelagius.WP_091997410
Marinobacter_santoriniensis.WP_008940562
Marinobacter_lutaoensis.WP_076723010
Marinobacter_nitratireducens.WP_081849584
Marinobacter_nitratireducens.KEF30254
Marinobacter_similis.WP_041340859
Marinobacter_salinus.WP_070970797
Marinobacter_lipolyticus.WP_012139232
Marinobacter_gudaonensis.WP_091990367
Marinobacter_guineae.WP_099617741
Marinobacter_subterrani.WP_048497643
Marinobacter.WP_064228426
Marinobacter_adhaerens.WP_069184747
Gammaproteobacteria.WP_008173247
Marinobacter_sp_es048.WP_088829487
Marinobacter_sp_YWL01.WP_062782838
Marinobacter_sp_es042.WP_088558239
Marinobacter_adhaerens.WP_014575870
Marinobacter_sp_LV10MA510_1.WP_098419733
Marinobacter_sp_ELB17.WP_007350284
Marinobacter_lipolyticus.WP_018402595
Marinobacter_sp_LV10R520_4.WP_098422270
Marinobacter_sp_BSs20148.WP_014869831
Marinobacter_psychrophilus.WP_048384236
Marinobacter_antarcticus.WP_072794848
Oceanospirillales_bacterium.PCI85002
Marinobacter_sp_ANT_B65.WP_096783637
Marinobacter_sp_LV10R510_11A.WP_096278321
Marinobacter_sp_LV10R510_8.WP_101469547
Marinobacter_aromaticivorans.WP_100687227
Marinobacter_salexigens.WP_100639224
B
Deferribacteraceae
Geovibrio_sp_L21-Ace-BES
Denitrovibrio_sp
ammaproteobacteria
Fangia_hongkongensis
Vibrio_diazotrophicus
Oceanospirillales_bacterium
Coxiella_burnetii_RSA_493
Gammaproteobacteria
Gammaproteobacteria_bacterium_RIFCSPHIGHO2_12_FULL_35_23
Gammaproteobacteria_bacterium_CG11_big_fil_rev_8_21_14_0_20_46_22
Oceanimonas
Oceanimonas_baumannii
Oceanimonas_sp_GK1
Tamilnaduibacter_salinus
Alteromonadaceae
Marinobacter
Marinobacter_nitratireducens
Marinobacter_daqiaonensis
Marinobacter_nanhaiticus_D15-8W
Marinobacter_subterrani
Marinobacter_adhaerens_HP15
Marinobacter_salexigens
Marinobacter_antarcticus
Marinobacter_excellens
Marinobacter_guineae
Marinobacter_hydrocarbonoclasticus_ATCC_49840
Marinobacter_lutaoensis
Marinobacter_daepoensis
Marinobacter_aromaticivorans
Marinobacter_algicola
Marinobacter_persicus
Marinobacter_salinus
Marinobacter_gudaonensis
Marinobacter_mobilis
Marinobacter_zhejiangensis
Marinobacter_santoriniensis
2666256
Marinobacter_pelagius
Marinobacter_lipolyticus
Marinobacter_psychrophilus
Marinobacter_similis
Marinobacter_segnicrescens
Marinobacter_sp_es048
Marinobacter_sp_LV10R520-4
Marinobacter_sp_LV10MA510-1
Marinobacter_sp_LV10R510-11A
Marinobacter_sp_EN3
Marinobacter_sp_ELB17
Marinobacter_sp_ANT_B65
Marinobacter_sp
Marinobacter_sp_BSs20148
Marinobacter_sp_YWL01
Marinobacter_sp_LQ44
Marinobacter_sp_HL-58
Marinobacter_sp_es042
Marinobacter_sp_T13-3
Marinobacter_sp_34-60-7
Marinobacter_sp_LV10R510-8

CBU_1022

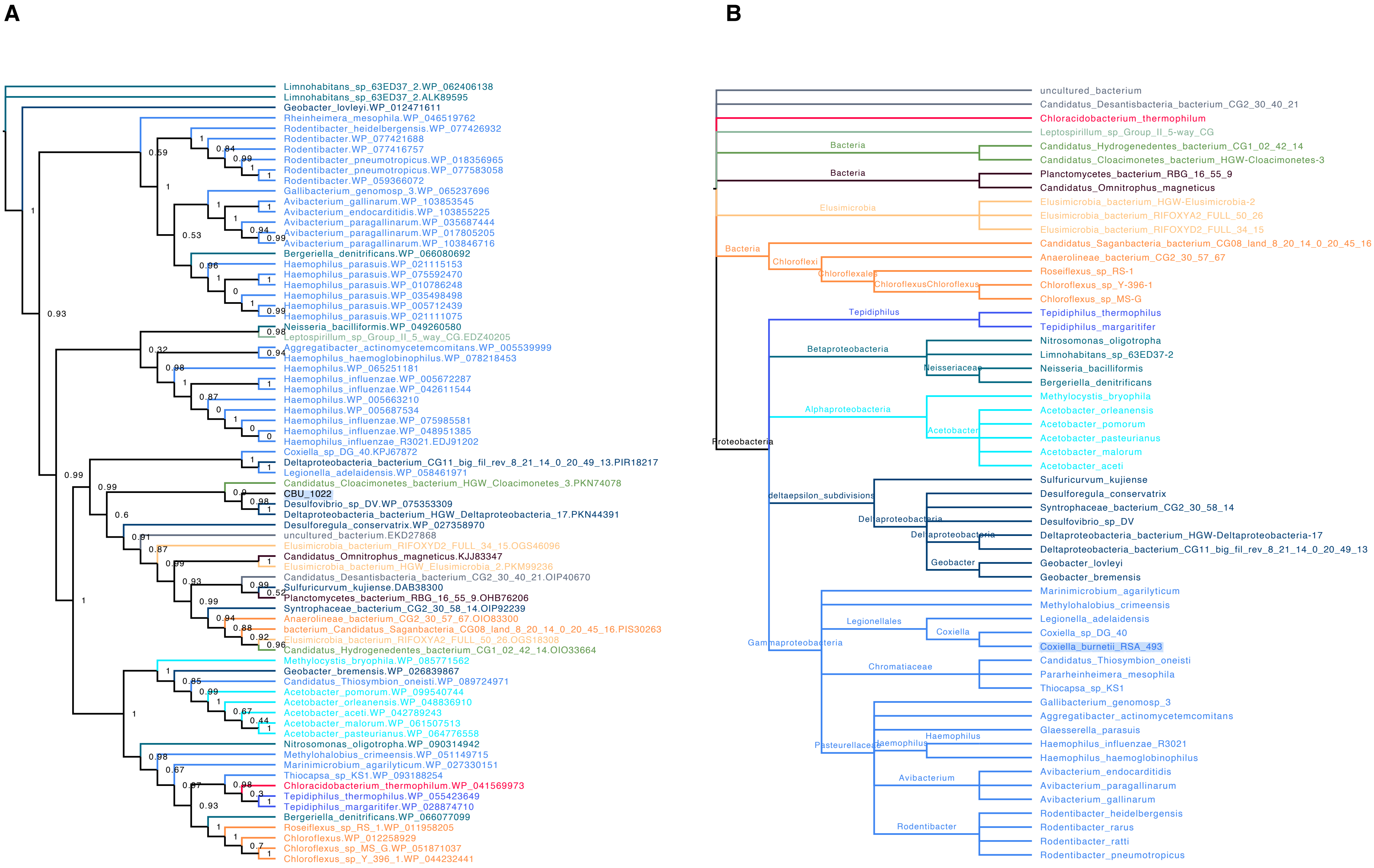
A
Limnohabitans_sp_63ED37_2.WP_062406138
Limnohabitans_sp_63ED37_2.ALK89595
Geobacter_lovleyi.WP_012471611
Rheinheimera_mesophila.WP_046519762
Rodentibacter_heidelbergensis.WP_077426932
Rodentibacter.WP_077421688
Rodentibacter.WP_077416757
Rodentibacter_pneumotropicus.WP_018356965
Rodentibacter_pneumotropicus.WP_077583058
Rodentibacter.WP_059366072
Gallibacterium_genomosp_3.WP_065237696
Avibacterium_gallinarum.WP_103853545
Avibacterium_endocarditidis.WP_103855225
Avibacterium_paragallinarum.WP_035687444
Avibacterium_paragallinarum.WP_017805205
Avibacterium_paragallinarum.WP_103846716
Bergeriella_denitrificans.WP_066080692
Haemophilus_parasuis.WP_021115153
Haemophilus_parasuis.WP_075592470
Haemophilus_parasuis.WP_010786248
Haemophilus_parasuis.WP_035498498
Haemophilus_parasuis.WP_005712439
Haemophilus_parasuis.WP_021111075
Neisseria_bacilliformis.WP_049260580
Leptospirillum_sp_Group_II_5_way_CG.EDZ40205
Aggregatibacter_actinomycetemcomitans.WP_005539999
Haemophilus_haemoglobinophilus.WP_078218453
Haemophilus.WP_065251181
Haemophilus_influenzae.WP_005672287
Haemophilus_influenzae.WP_042611544
Haemophilus.WP_005663210
Haemophilus.WP_005687534
Haemophilus_influenzae.WP_075985581
Haemophilus_influenzae.WP_048951385
Haemophilus_influenzae_R3021.EDJ91202
Coxiella_sp_DG_40.KPJ67872
Deltaproteobacteria_bacterium_CG11_big_fil_rev_8_21_14_0_20_49_13.PIR18217
Legionella_adelaidensis.WP_058461971
Candidatus_Cloacimonetes_bacterium_HGW_Cloacimonetes_3.PKN74078
CBU_1022
Desulfovibrio_sp_DV.WP_075353309
Deltaproteobacteria_bacterium_HGW_Deltaproteobacteria_17.PKN44391
Desulforegula_conservatrix.WP_027358970
uncultured_bacterium.EKD27868
Elusimicrobia_bacterium_RIFOXYD2_FULL_34_15.OGS46096
Candidatus_Omnitrophus_magneticus.KJJ83347
Elusimicrobia_bacterium_HGW_Elusimicrobia_2.PKM99236
Candidatus_Desantisbacteria_bacterium_CG2_30_40_21.OIP40670
Sulfuricurvum_kujiense.DAB38300
Planctomycetes_bacterium_RBG_16_55_9.OHB76206
Syntrophaceae_bacterium_CG2_30_58_14.OIP92239
Anaerolineae_bacterium_CG2_30_57_67.OIO83300
bacterium_Candidatus_Saganbacteria_CG08_land_8_20_14_0_20_45_16.PIS30263
Elusimicrobia_bacterium_RIFOXYA2_FULL_50_26.OGS18308
Candidatus_Hydrogenedentes_bacterium_CG1_02_42_14.OIO33664
Methylocystis_bryophila.WP_085771562
Geobacter_bremensis.WP_026839867
Candidatus_Thiosymbion_oneisti.WP_089724971
Acetobacter_pomorum.WP_099540744
Acetobacter_orleanensis.WP_048836910
Acetobacter_aceti.WP_042789243
Acetobacter_malorum.WP_061507513
Acetobacter_pasteurianus.WP_064776558
Nitrosomonas_oligotropha.WP_090314942
Methylohalobius_crimeensis.WP_051149715
Marinimicrobium_agarilyticum.WP_027330151
Thiocapsa_sp_KS1.WP_093188254
Chloracidobacterium_thermophilum.WP_041569973
Tepidiphilus_thermophilus.WP_055423649
Tepidiphilus_margaritifer.WP_028874710
Bergeriella_denitrificans.WP_066077099
Roseiflexus_sp_RS_1.WP_011958205
Chloroflexus.WP_012258929
Chloroflexus_sp_MS_G.WP_051871037
Chloroflexus_sp_Y_396_1.WP_044232441
B
uncultured_bacterium
Candidatus_Desantisbacteria_bacterium_CG2_30_40_21
Chloracidobacterium_thermophilum
Leptospirillum_sp_Group_II_5-way_CG
Bacteria
Candidatus_Hydrogenedentes_bacterium_CG1_02_42_14
Candidatus_Cloacimonetes_bacterium_HGW-Cloacimonetes-3
Bacteria
Planctomycetes_bacterium_RBG_16_55_9
Candidatus_Omnitrophus_magneticus
Elusimicrobia
Elusimicrobia_bacterium_HGW-Elusimicrobia-2
Elusimicrobia_bacterium_RIFOXYA2_FULL_50_26
Elusimicrobia_bacterium_RIFOXYD2_FULL_34_15
Bacteria
Candidatus_Saganbacteria_bacterium_CG08_land_8_20_14_0_20_45_16
Chloroflexi
Anaerolineae_bacterium_CG2_30_57_67
Chloroflexales
Roseiflexus_sp_RS-1
ChloroflexusChloroflexus
Chloroflexus_sp_Y-396-1
Chloroflexus_sp_MS-G
Tepidiphilus
Tepidiphilus_thermophilus
Tepidiphilus_margaritifer
Betaproteobacteria
Nitrosomonas_oligotropha
Limnohabitans_sp_63ED37-2
Neisseriaceae
Neisseria_bacilliformis
Bergeriella_denitrificans
Alphaproteobacteria
Methylocystis_bryophila
Acetobacter
Acetobacter_orleanensis
Acetobacter_pomorum
Acetobacter_pasteurianus
Acetobacter_malorum
Acetobacter_aceti
Proteobacteria
deltaepsilon_subdivisions
Sulfuricurvum_kujiense
Deltaproteobacteria
Desulforegula_conservatrix
Syntrophaceae_bacterium_CG2_30_58_14
Desulfovibrio_sp_DV
Deltaproteobacteria
Deltaproteobacteria_bacterium_HGW-Deltaproteobacteria-17
Deltaproteobacteria_bacterium_CG11_big_fil_rev_8_21_14_0_20_49_13
Geobacter
Geobacter_lovleyi
Geobacter_bremensis
Marinimicrobium_agarilyticum
Methylohalobius_crimeensis
Legionellales
Legionella_adelaidensis
Coxiella
Coxiella_sp_DG_40
Coxiella_burnetii_RSA_493
Gammaproteobacteria
Chromatiaceae
Candidatus_Thiosymbion_oneisti
Pararheinheimera_mesophila
Thiocapsa_sp_KS1
Gallibacterium_genomosp_3
Aggregatibacter_actinomycetemcomitans
Glaesserella_parasuis
Haemophilus
Pasteurellaceae
Haemophilus
Haemophilus_influenzae_R3021
Haemophilus_haemoglobinophilus
Avibacterium
Avibacterium_endocarditidis
Avibacterium_paragallinarum
Avibacterium_gallinarum
Rodentibacter
Rodentibacter_heidelbergensis
Rodentibacter_rarus
Rodentibacter_ratti
Rodentibacter_pneumotropicus

CBU_1017

A B

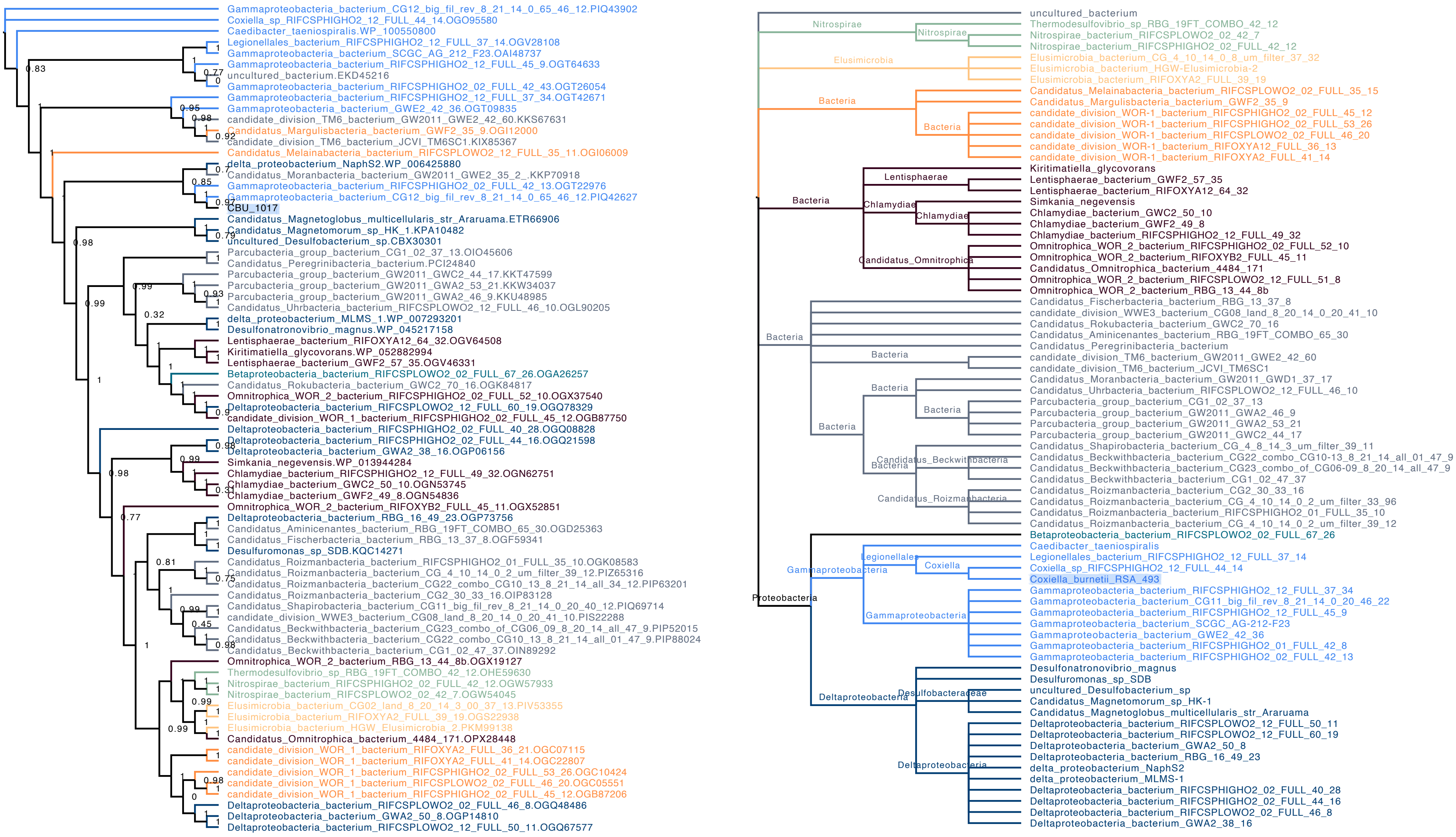

Gammaproteobacteria_bacterium_CG12_big_fil_rev_8_21_14_0_65_46_12.PIQ43902
Coxiella_sp_RIFCSPHIGHO2_12_FULL_44_14.OGO95580
Caedibacter_taeniospiralis.WP_100550800
Legionellales_bacterium_RIFCSPHIGHO2_12_FULL_37_14.OGV28108
Gammaproteobacteria_bacterium_SCGC_AG_212_F23.OAI48737
Gammaproteobacteria_bacterium_RIFCSPHIGHO2_12_FULL_45_9.OGT64633
uncultured_bacterium.EKD45216
Gammaproteobacteria_bacterium_RIFCSPHIGHO2_02_FULL_42_43.OGT26054
Gammaproteobacteria_bacterium_RIFCSPHIGHO2_12_FULL_37_34.OGT42671
Gammaproteobacteria_bacterium_GWE2_42_36.OGT09835
candidate_division_TM6_bacterium_GW2011_GWE2_42_60.KKS67631
Candidatus_Margulisbacteria_bacterium_GWF2_35_9.OGI12000
candidate_division_TM6_bacterium_JCVI_TM6SC1.KIX85367
Candidatus_Melainabacteria_bacterium_RIFCSPLOWO2_12_FULL_35_11.OGI06009
delta_proteobacterium_NaphS2.WP_006425880
Candidatus_Moranbacteria_bacterium_GW2011_GWE2_35_2_.KKP70918
Gammaproteobacteria_bacterium_RIFCSPHIGHO2_02_FULL_42_13.OGT22976
Gammaproteobacteria_bacterium_CG12_big_fil_rev_8_21_14_0_65_46_12.PIQ42627
CBU_1017
Candidatus_Magnetoglobus_multicellularis_str_Araruama.ETR66906
Candidatus_Magnetomorum_sp_HK_1.KPA10482
uncultured_Desulfobacterium_sp.CBX30301
Parcubacteria_group_bacterium_CG1_02_37_13.OIO45606
Candidatus_Peregrinibacteria_bacterium.PCI24840
Parcubacteria_group_bacterium_GW2011_GWC2_44_17.KKT47599
Parcubacteria_group_bacterium_GW2011_GWA2_53_21.KKW34037
Parcubacteria_group_bacterium_GW2011_GWA2_46_9.KKU48985
Candidatus_Uhrbacteria_bacterium_RIFCSPLOWO2_12_FULL_46_10.OGL90205
delta_proteobacterium_MLMS_1.WP_007293201
Desulfonatronovibrio_magnus.WP_045217158
Lentisphaerae_bacterium_RIFOXYA12_64_32.OGV64508
Kiritimatiella_glycovorans.WP_052882994
Lentisphaerae_bacterium_GWF2_57_35.OGV46331
Betaproteobacteria_bacterium_RIFCSPLOWO2_02_FULL_67_26.OGA26257
Candidatus_Rokubacteria_bacterium_GWC2_70_16.OGK84817
Omnitrophica_WOR_2_bacterium_RIFCSPHIGHO2_02_FULL_52_10.OGX37540
Deltaproteobacteria_bacterium_RIFCSPLOWO2_12_FULL_60_19.OGQ78329
candidate_division_WOR_1_bacterium_RIFCSPHIGHO2_02_FULL_45_12.OGB87750
Deltaproteobacteria_bacterium_RIFCSPHIGHO2_02_FULL_40_28.OGQ08828
Deltaproteobacteria_bacterium_RIFCSPHIGHO2_02_FULL_44_16.OGQ21598
Deltaproteobacteria_bacterium_GWA2_38_16.OGP06156
Simkania_negevensis.WP_013944284
Chlamydiae_bacterium_RIFCSPHIGHO2_12_FULL_49_32.OGN62751
Chlamydiae_bacterium_GWC2_50_10.OGN53745
Chlamydiae_bacterium_GWF2_49_8.OGN54836
Omnitrophica_WOR_2_bacterium_RIFOXYB2_FULL_45_11.OGX52851
Deltaproteobacteria_bacterium_RBG_16_49_23.OGP73756
Candidatus_Aminicenantes_bacterium_RBG_19FT_COMBO_65_30.OGD25363
Candidatus_Fischerbacteria_bacterium_RBG_13_37_8.OGF59341
Desulfuromonas_sp_SDB.KQC14271
Candidatus_Roizmanbacteria_bacterium_RIFCSPHIGHO2_01_FULL_35_10.OGK08583
Candidatus_Roizmanbacteria_bacterium_CG_4_10_14_0_2_um_filter_39_12.PIZ65316
Candidatus_Roizmanbacteria_bacterium_CG22_combo_CG10_13_8_21_14_all_34_12.PIP63201
Candidatus_Roizmanbacteria_bacterium_CG2_30_33_16.OIP83128
Candidatus_Shapirobacteria_bacterium_CG11_big_fil_rev_8_21_14_0_20_40_12.PIQ69714
candidate_division_WWE3_bacterium_CG08_land_8_20_14_0_20_41_10.PIS22288
Candidatus_Beckwithbacteria_bacterium_CG23_combo_of_CG06_09_8_20_14_all_47_9.PIP52015
Candidatus_Beckwithbacteria_bacterium_CG22_combo_CG10_13_8_21_14_all_01_47_9.PIP88024
Candidatus_Beckwithbacteria_bacterium_CG1_02_47_37.OIN89292
Omnitrophica_WOR_2_bacterium_RBG_13_44_8b.OGX19127
Thermodesulfovibrio_sp_RBG_19FT_COMBO_42_12.OHE59630
Nitrospirae_bacterium_RIFCSPHIGHO2_02_FULL_42_12.OGW57933
Nitrospirae_bacterium_RIFCSPLOWO2_02_42_7.OGW54045
Elusimicrobia_bacterium_CG02_land_8_20_14_3_00_37_13.PIV53355
Elusimicrobia_bacterium_RIFOXYA2_FULL_39_19.OGS22938
Elusimicrobia_bacterium_HGW_Elusimicrobia_2.PKM99138
Candidatus_Omnitrophica_bacterium_4484_171.OPX28448
candidate_division_WOR_1_bacterium_RIFOXYA2_FULL_36_21.OGC07115
candidate_division_WOR_1_bacterium_RIFOXYA2_FULL_41_14.OGC22807
candidate_division_WOR_1_bacterium_RIFCSPHIGHO2_02_FULL_53_26.OGC10424
candidate_division_WOR_1_bacterium_RIFCSPLOWO2_02_FULL_46_20.OGC05551
candidate_division_WOR_1_bacterium_RIFCSPHIGHO2_02_FULL_45_12.OGB87206
Deltaproteobacteria_bacterium_RIFCSPLOWO2_02_FULL_46_8.OGQ48486
Deltaproteobacteria_bacterium_GWA2_50_8.OGP14810
Deltaproteobacteria_bacterium_RIFCSPLOWO2_12_FULL_50_11.OGQ67577
uncultured_bacterium
Nitrospirae
Thermodesulfovibrio_sp_RBG_19FT_COMBO_42_12
Nitrospirae_bacterium_RIFCSPLOWO2_02_42_7
Nitrospirae_bacterium_RIFCSPHIGHO2_02_FULL_42_12
Elusimicrobia
Elusimicrobia_bacterium_CG_4_10_14_0_8_um_filter_37_32
Elusimicrobia_bacterium_HGW-Elusimicrobia-2
Elusimicrobia_bacterium_RIFOXYA2_FULL_39_19
Bacteria
Candidatus_Melainabacteria_bacterium_RIFCSPLOWO2_02_FULL_35_15
Candidatus_Margulisbacteria_bacterium_GWF2_35_9
candidate_division_WOR-1_bacterium_RIFCSPHIGHO2_02_FULL_45_12
candidate_division_WOR-1_bacterium_RIFCSPHIGHO2_02_FULL_53_26
candidate_division_WOR-1_bacterium_RIFCSPLOWO2_02_FULL_46_20
candidate_division_WOR-1_bacterium_RIFOXYA12_FULL_36_13
candidate_division_WOR-1_bacterium_RIFOXYA2_FULL_41_14
Lentisphaerae
Chlamydiae
Candidatus_Omnitrophica
Kiritimatiella_glycovorans
Lentisphaerae_bacterium_GWF2_57_35
Lentisphaerae_bacterium_RIFOXYA12_64_32
Simkania_negevensis
Chlamydiae_bacterium_GWC2_50_10
Chlamydiae_bacterium_GWF2_49_8
Chlamydiae_bacterium_RIFCSPHIGHO2_12_FULL_49_32
Omnitrophica_WOR_2_bacterium_RIFCSPHIGHO2_02_FULL_52_10
Omnitrophica_WOR_2_bacterium_RIFOXYB2_FULL_45_11
Candidatus_Omnitrophica_bacterium_4484_171
Omnitrophica_WOR_2_bacterium_RIFCSPLOWO2_12_FULL_51_8
Omnitrophica_WOR_2_bacterium_RBG_13_44_8b
Candidatus_Fischerbacteria_bacterium_RBG_13_37_8
candidate_division_WWE3_bacterium_CG08_land_8_20_14_0_20_41_10
Candidatus_Rokubacteria_bacterium_GWC2_70_16
Candidatus_Aminicenantes_bacterium_RBG_19FT_COMBO_65_30
Candidatus_Peregrinibacteria_bacterium
candidate_division_TM6_bacterium_GW2011_GWE2_42_60
candidate_division_TM6_bacterium_JCVI_TM6SC1
Candidatus_Moranbacteria_bacterium_GW2011_GWD1_37_17
Candidatus_Uhrbacteria_bacterium_RIFCSPLOWO2_12_FULL_46_10
Parcubacteria_group_bacterium_CG1_02_37_13
Parcubacteria_group_bacterium_GW2011_GWA2_46_9
Parcubacteria_group_bacterium_GW2011_GWA2_53_21
Parcubacteria_group_bacterium_GW2011_GWC2_44_17
Candidatus_Beckwithbacteria
Candidatus_Roizmanbacteria
Candidatus_Shapirobacteria_bacterium_CG_4_8_14_3_um_filter_39_11
Candidatus_Beckwithbacteria_bacterium_CG22_combo_CG10-13_8_21_14_all_01_47_9
Candidatus_Beckwithbacteria_bacterium_CG23_combo_of_CG06-09_8_20_14_all_47_9
Candidatus_Beckwithbacteria_bacterium_CG1_02_47_37
Candidatus_Roizmanbacteria_bacterium_CG2_30_33_16
Candidatus_Roizmanbacteria_bacterium_CG_4_10_14_0_2_um_filter_33_96
Candidatus_Roizmanbacteria_bacterium_RIFCSPHIGHO2_01_FULL_35_10
Candidatus_Roizmanbacteria_bacterium_CG_4_10_14_0_2_um_filter_39_12
Proteobacteria
Gammaproteobacteria
Legionellales
Coxiella
Deltaproteobacteria
Desulfobacteraceae
Betaproteobacteria_bacterium_RIFCSPLOWO2_02_FULL_67_26
Caedibacter_taeniospiralis
Legionellales_bacterium_RIFCSPHIGHO2_12_FULL_37_14
Coxiella_sp_RIFCSPHIGHO2_12_FULL_44_14
Coxiella_burnetii_RSA_493
Gammaproteobacteria_bacterium_RIFCSPHIGHO2_12_FULL_37_34
Gammaproteobacteria_bacterium_CG11_big_fil_rev_8_21_14_0_20_46_22
Gammaproteobacteria_bacterium_RIFCSPHIGHO2_12_FULL_45_9
Gammaproteobacteria_bacterium_SCGC_AG-212-F23
Gammaproteobacteria_bacterium_GWE2_42_36
Gammaproteobacteria_bacterium_RIFCSPHIGHO2_01_FULL_42_8
Gammaproteobacteria_bacterium_RIFCSPHIGHO2_02_FULL_42_13
Desulfonatronovibrio_magnus
Desulfuromonas_sp_SDB
uncultured_Desulfobacterium_sp
Candidatus_Magnetomorum_sp_HK-1
Candidatus_Magnetoglobus_multicellularis_str_Araruama
Deltaproteobacteria_bacterium_RIFCSPLOWO2_12_FULL_50_11
Deltaproteobacteria_bacterium_RIFCSPLOWO2_12_FULL_60_19
Deltaproteobacteria_bacterium_GWA2_50_8
Deltaproteobacteria_bacterium_RBG_16_49_23
delta_proteobacterium_NaphS2
delta_proteobacterium_MLMS-1
Deltaproteobacteria_bacterium_RIFCSPHIGHO2_02_FULL_40_28
Deltaproteobacteria_bacterium_RIFCSPHIGHO2_02_FULL_44_16
Deltaproteobacteria_bacterium_RIFCSPLOWO2_02_FULL_46_8
Deltaproteobacteria_bacterium_GWA2_38_16

CBU_1015

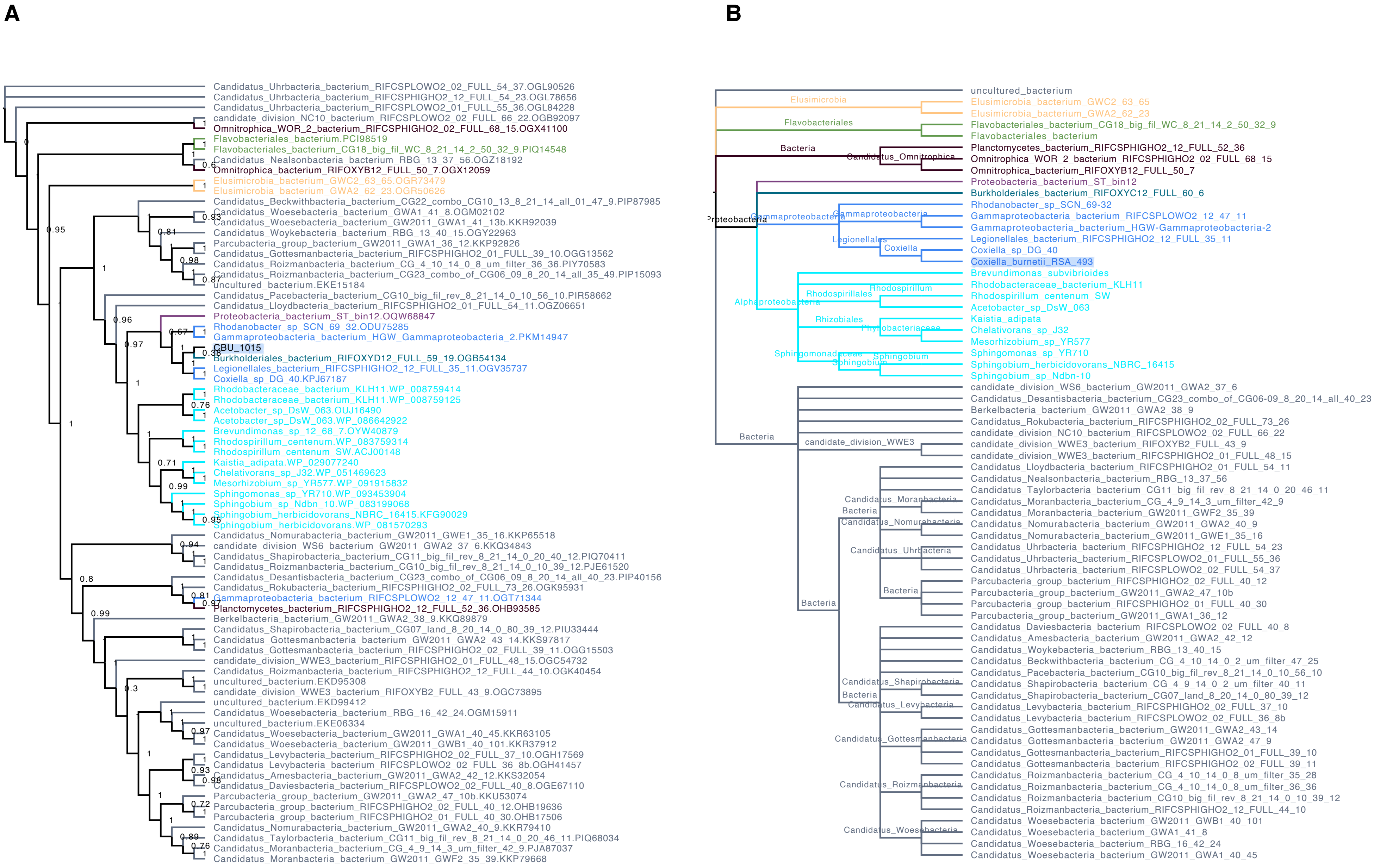

CBU_0961

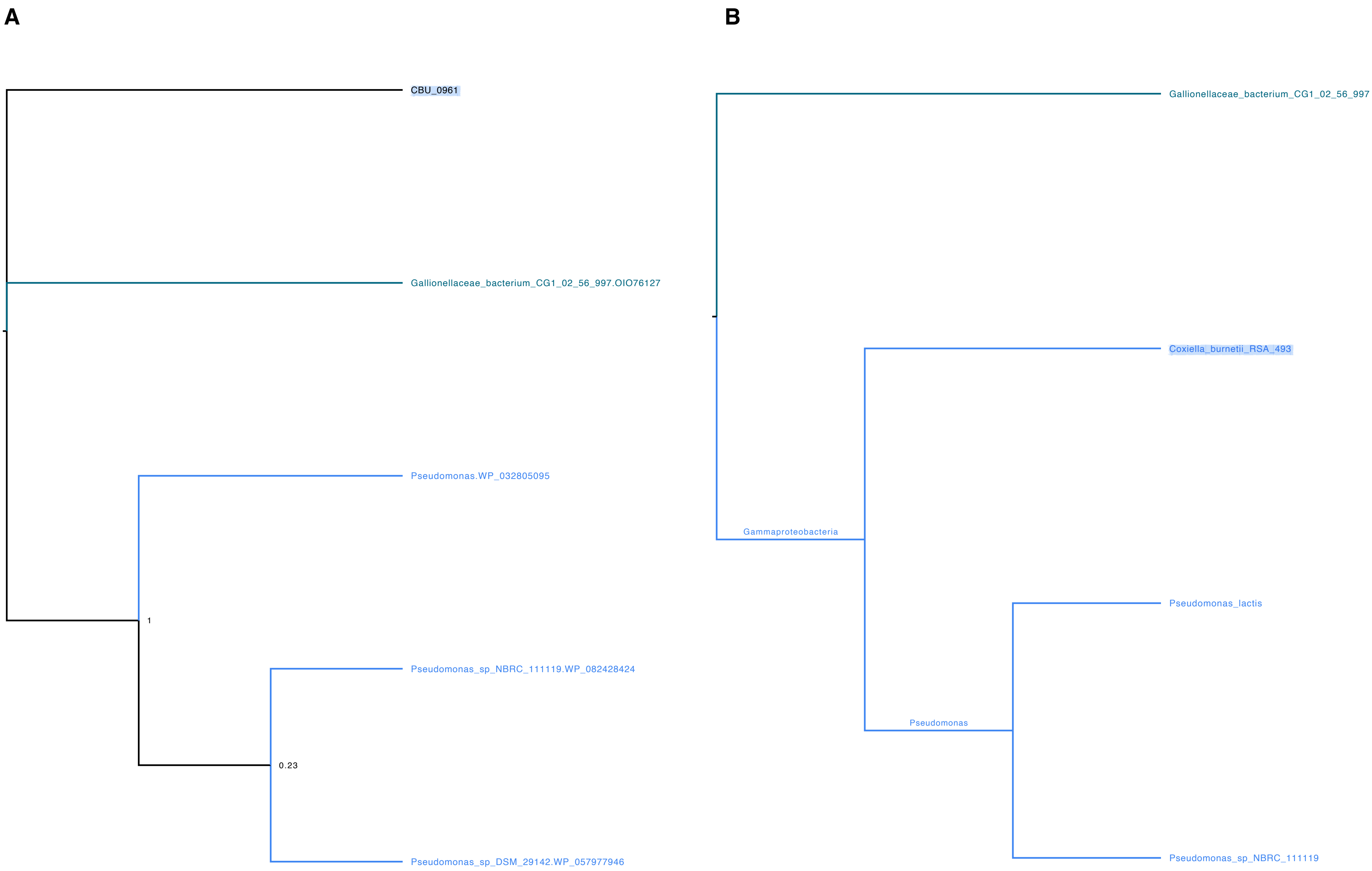
A
CBU_0961
Gallionellaceae_bacterium_CG1_02_56_997.OIO76127
Pseudomonas.WP_032805095
1
Pseudomonas_sp_NBRC_111119.WP_082428424
0.23
Pseudomonas_sp_DSM_29142.WP_057977946
B
Gallionellaceae_bacterium_CG1_02_56_997
Coxiella_burnetii_RSA_493
Gammaproteobacteria
Pseudomonas_lactis
Pseudomonas
Pseudomonas_sp_NBRC_111119

CBU_0960

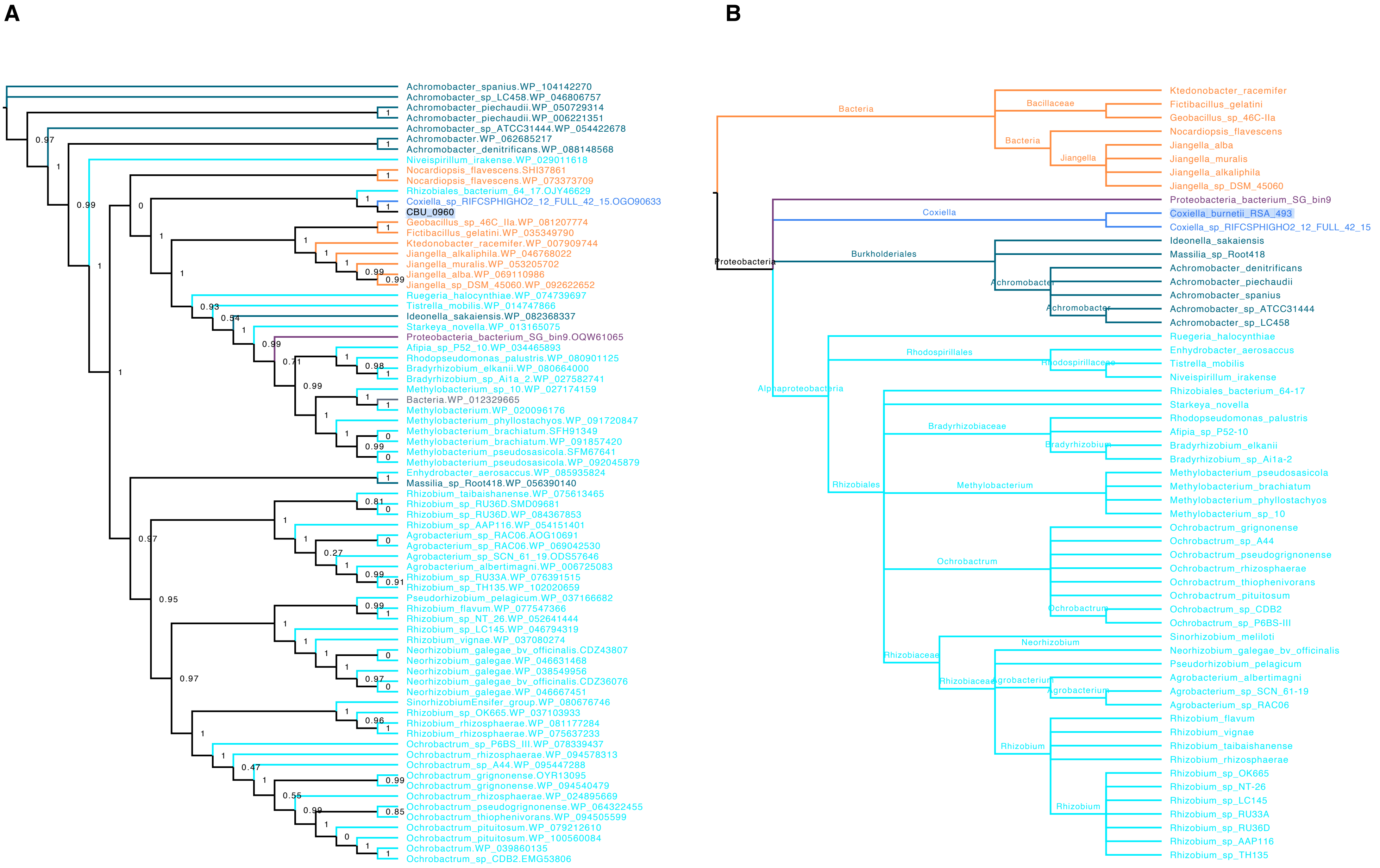
A
Achromobacter_spanius.WP_104142270
Achromobacter_sp_LC458.WP_046806757
Achromobacter_piechaudii.WP_050729314
Achromobacter_piechaudii.WP_006221351
Achromobacter_sp_ATCC31444.WP_054422678
Achromobacter.WP_062685217
Achromobacter_denitrificans.WP_088148568
Niveispirillum_irakense.WP_029011618
Nocardiopsis_flavescens.SHI37861
Nocardiopsis_flavescens.WP_073373709
Rhizobiales_bacterium_64_17.OJY46629
Coxiella_sp_RIFCSPHIGHO2_12_FULL_42_15.OGO90633
CBU_0960
Geobacillus_sp_46C_IIa.WP_081207774
Fictibacillus_gelatini.WP_035349790
Ktedonobacter_racemifer.WP_007909744
Jiangella_alkaliphila.WP_046768022
Jiangella_muralis.WP_053205702
Jiangella_alba.WP_069110986
Jiangella_sp_DSM_45060.WP_092622652
Ruegeria_halocynthiae.WP_074739697
Tistrella_mobilis.WP_014747866
Ideonella_sakaiensis.WP_082368337
Starkeya_novella.WP_013165075
Proteobacteria_bacterium_SG_bin9.OQW61065
Afipia_sp_P52_10.WP_034465893
Rhodopseudomonas_palustris.WP_080901125
Bradyrhizobium_elkanii.WP_080664000
Bradyrhizobium_sp_Ai1a_2.WP_027582741
Methylobacterium_sp_10.WP_027174159
Bacteria.WP_012329665
Methylobacterium.WP_020096176
Methylobacterium_phyllostachyos.WP_091720847
Methylobacterium_brachiatum.SFH91349
Methylobacterium_brachiatum.WP_091857420
Methylobacterium_pseudosasicola.SFM67641
Methylobacterium_pseudosasicola.WP_092045879
Enhydrobacter_aerosaccus.WP_085935824
Massilia_sp_Root418.WP_056390140
Rhizobium_taibaishanense.WP_075613465
Rhizobium_sp_RU36D.SMD09681
Rhizobium_sp_RU36D.WP_084367853
Rhizobium_sp_AAP116.WP_054151401
Agrobacterium_sp_RAC06.AOG10691
Agrobacterium_sp_RAC06.WP_069042530
Agrobacterium_sp_SCN_61_19.ODS57646
Agrobacterium_albertimagni.WP_006725083
Rhizobium_sp_RU33A.WP_076391515
Rhizobium_sp_TH135.WP_102020659
Pseudorhizobium_pelagicum.WP_037166682
Rhizobium_flavum.WP_077547366
Rhizobium_sp_NT_26.WP_052641444
Rhizobium_sp_LC145.WP_046794319
Rhizobium_vignae.WP_037080274
Neorhizobium_galegae_bv_officinalis.CDZ43807
Neorhizobium_galegae.WP_046631468
Neorhizobium_galegae.WP_038549956
Neorhizobium_galegae_bv_officinalis.CDZ36076
Neorhizobium_galegae.WP_046667451
SinorhizobiumEnsifer_group.WP_080676746
Rhizobium_sp_OK665.WP_037103933
Rhizobium_rhizosphaerae.WP_081177284
Rhizobium_rhizosphaerae.WP_075637233
Ochrobactrum_sp_P6BS_III.WP_078339437
Ochrobactrum_rhizosphaerae.WP_094578313
Ochrobactrum_sp_A44.WP_095447288
Ochrobactrum_grignonense.OYR13095
Ochrobactrum_grignonense.WP_094540479
Ochrobactrum_rhizosphaerae.WP_024895669
Ochrobactrum_pseudogrignonense.WP_064322455
Ochrobactrum_thiophenivorans.WP_094505599
Ochrobactrum_pituitosum.WP_079212610
Ochrobactrum_pituitosum.WP_100560084
Ochrobactrum.WP_039860135
Ochrobactrum_sp_CDB2.EMG53806
B
Bacteria
Bacillaceae
Bacteria
Jiangella
Ktedonobacter_racemifer
Fictibacillus_gelatini
Geobacillus_sp_46C-IIa
Nocardiopsis_flavescens
Jiangella_alba
Jiangella_muralis
Jiangella_alkaliphila
Jiangella_sp_DSM_45060
Proteobacteria
Proteobacteria_bacterium_SG_bin9
Coxiella
Coxiella_burnetii_RSA_493
Coxiella_sp_RIFCSPHIGHO2_12_FULL_42_15
Burkholderiales
Achromobacter
Achromobacter
Ideonella_sakaiensis
Massilia_sp_Root418
Achromobacter_denitrificans
Achromobacter_piechaudii
Achromobacter_spanius
Achromobacter_sp_ATCC31444
Achromobacter_sp_LC458
Alphaproteobacteria
Rhodospirillales
Rhodospirillaceae
Ruegeria_halocynthiae
Enhydrobacter_aerosaccus
Tistrella_mobilis
Niveispirillum_irakense
Rhizobiales_bacterium_64-17
Starkeya_novella
Bradyrhizobiaceae
Bradyrhizobium
Rhodopseudomonas_palustris
Afipia_sp_P52-10
Bradyrhizobium_elkanii
Bradyrhizobium_sp_Ai1a-2
Rhizobiales
Methylobacterium
Methylobacterium_pseudosasicola
Methylobacterium_brachiatum
Methylobacterium_phyllostachyos
Methylobacterium_sp_10
Ochrobactrum
Ochrobactrum
Ochrobactrum_grignonense
Ochrobactrum_sp_A44
Ochrobactrum_pseudogrignonense
Ochrobactrum_rhizosphaerae
Ochrobactrum_thiophenivorans
Ochrobactrum_pituitosum
Ochrobactrum_sp_CDB2
Ochrobactrum_sp_P6BS-III
Rhizobiaceae
Rhizobiaceae
Neorhizobium
Agrobacterium
Agrobacterium
Sinorhizobium_meliloti
Neorhizobium_galegae_bv_officinalis
Pseudorhizobium_pelagicum
Agrobacterium_albertimagni
Agrobacterium_sp_SCN_61-19
Agrobacterium_sp_RAC06
Rhizobium
Rhizobium
Rhizobium_flavum
Rhizobium_vignae
Rhizobium_taibaishanense
Rhizobium_rhizosphaerae
Rhizobium_sp_OK665
Rhizobium_sp_NT-26
Rhizobium_sp_LC145
Rhizobium_sp_RU33A
Rhizobium_sp_RU36D
Rhizobium_sp_AAP116
Rhizobium_sp_TH135

CBU_0949

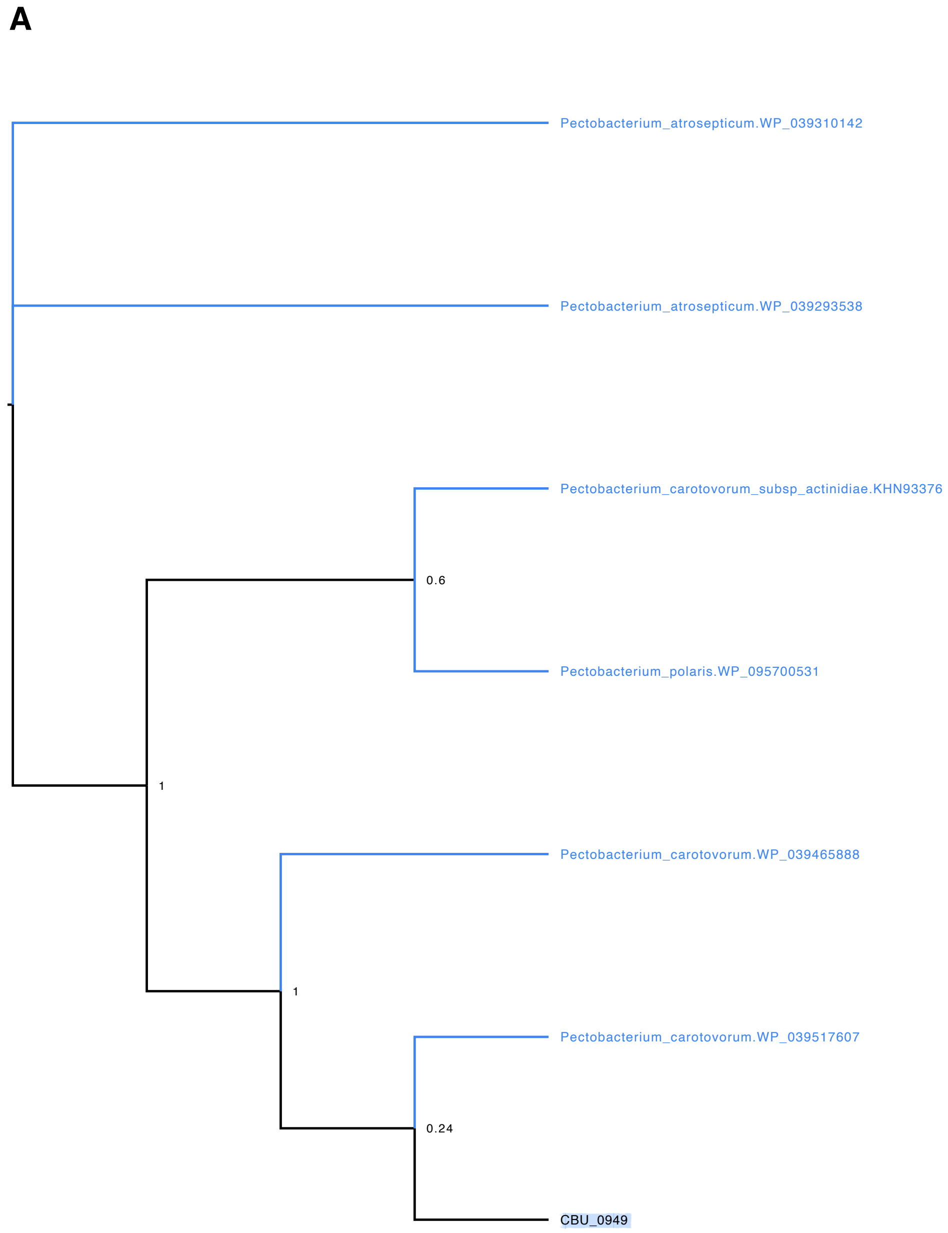
A
Pectobacterium_atrosepticum.WP_039310142
Pectobacterium_atrosepticum.WP_039293538
Pectobacterium_carotovorum_subsp_actinidiae.KHN93376
0.6
Pectobacterium_polaris.WP_095700531
1
Pectobacterium_carotovorum.WP_039465888
1
Pectobacterium_carotovorum.WP_039517607
0.24
CBU_0949

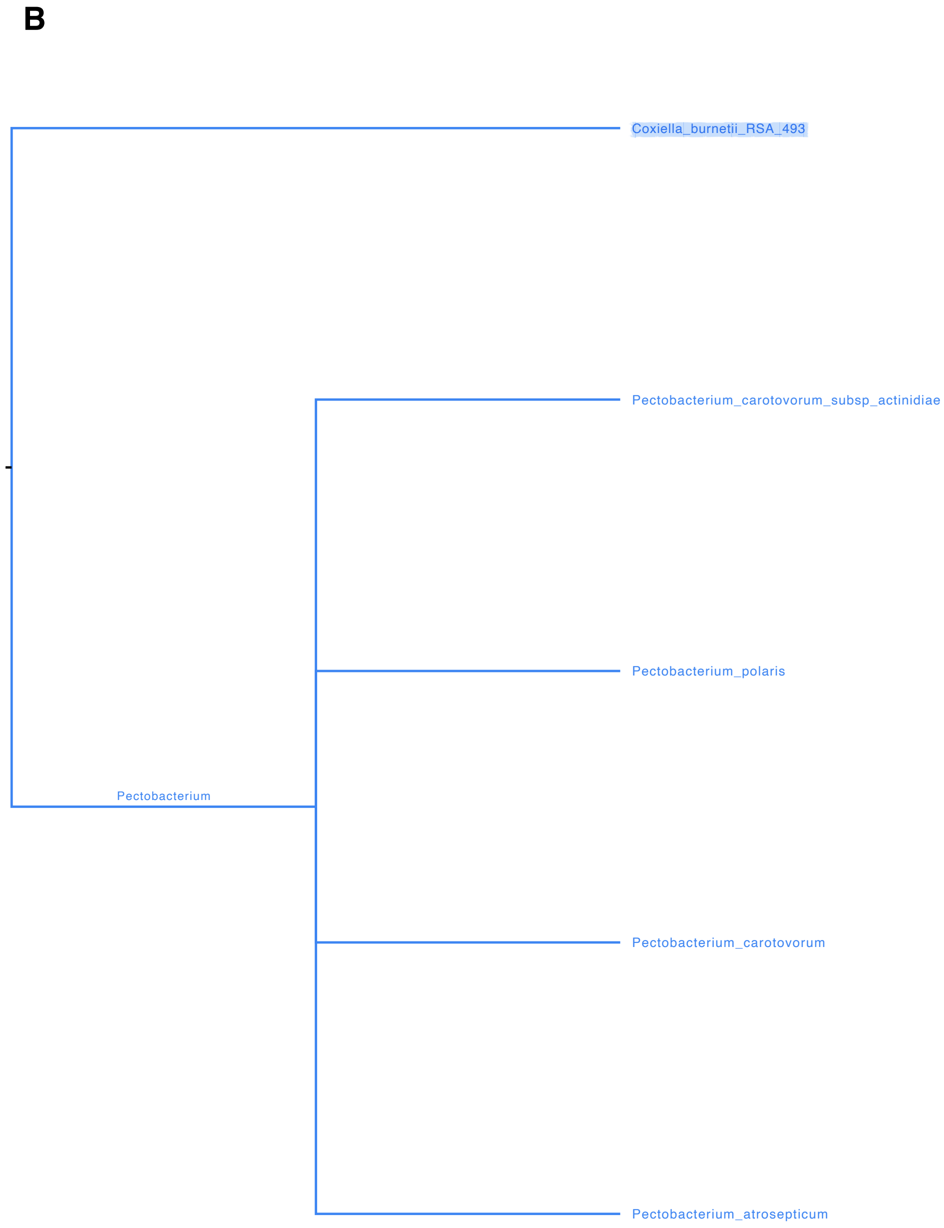
B
Coxiella_burnetii_RSA_493
Pectobacterium_carotovorum_subsp_actinidiae
Pectobacterium_polaris
Pectobacterium
Pectobacterium_carotovorum
Pectobacterium_atrosepticum

CBU_0946

A **B**

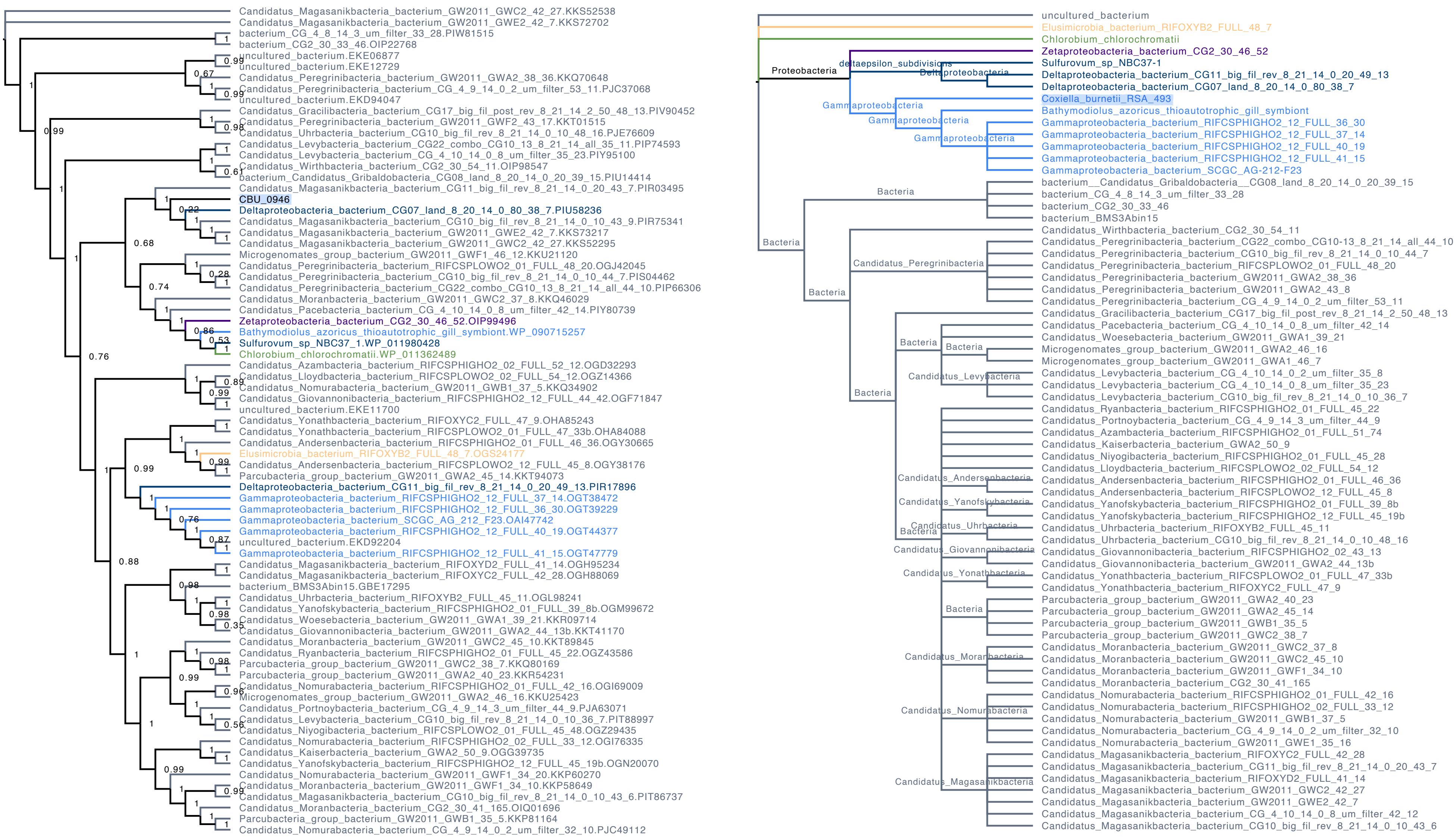

Candidatus_Magasanikbacteria_bacterium_GW2011_GWC2_42_27.KKS52538
Candidatus_Magasanikbacteria_bacterium_GW2011_GWE2_42_7.KKS72702
bacterium_CG_4_8_14_3_um_filter_33_28.PIW81515
bacterium_CG2_30_33_46.OIP22768
uncultured_bacterium.EKE06877
uncultured_bacterium.EKE12729
Candidatus_Peregrinibacteria_bacterium_GW2011_GWA2_38_36.KKQ70648
Candidatus_Peregrinibacteria_bacterium_CG_4_9_14_0_2_um_filter_53_11.PJC37068
uncultured_bacterium.EKD94047
Candidatus_Gracilibacteria_bacterium_CG17_big_fil_post_rev_8_21_14_2_50_48_13.PIV90452
Candidatus_Peregrinibacteria_bacterium_GW2011_GWF2_43_17.KKT01515
Candidatus_Uhrbacteria_bacterium_CG10_big_fil_rev_8_21_14_0_10_48_16.PJE76609
Candidatus_Levybacteria_bacterium_CG22_combo_CG10_13_8_21_14_all_35_11.PIP74593
Candidatus_Levybacteria_bacterium_CG_4_10_14_0_8_um_filter_35_23.PIY95100
Candidatus_Wirthbacteria_bacterium_CG2_30_54_11.OIP98547
bacterium_Candidatus_Gribaldobacteria_CG08_land_8_20_14_0_20_39_15.PIU14414
Candidatus_Magasanikbacteria_bacterium_CG11_big_fil_rev_8_21_14_0_20_43_7.PIR03495
CBU_0946
Deltaproteobacteria_bacterium_CG07_land_8_20_14_0_80_38_7.PIU58236
Candidatus_Magasanikbacteria_bacterium_CG10_big_fil_rev_8_21_14_0_10_43_9.PIR75341
Candidatus_Magasanikbacteria_bacterium_GW2011_GWE2_42_7.KKS73217
Candidatus_Magasanikbacteria_bacterium_GW2011_GWC2_42_27.KKS52295
Microgenomates_group_bacterium_GW2011_GWF1_46_12.KKU21120
Candidatus_Peregrinibacteria_bacterium_RIFCSPLOWO2_01_FULL_48_20.OGJ42045
Candidatus_Peregrinibacteria_bacterium_CG10_big_fil_rev_8_21_14_0_10_44_7.PIS04462
Candidatus_Peregrinibacteria_bacterium_CG22_combo_CG10_13_8_21_14_all_44_10.PIP66306
Candidatus_Moranbacteria_bacterium_GW2011_GWC2_37_8.KKQ46029
Candidatus_Pacebacteria_bacterium_CG_4_10_14_0_8_um_filter_42_14.PIY80739
Zetaproteobacteria_bacterium_CG2_30_46_52.OIP99496
Bathymodiolus_azoricus_thioautotrophic_gill_symbiont.WP_090715257
Sulfurovum_sp_NBC37_1.WP_011980428
Chlorobium_chlorochromatii.WP_011362489
Candidatus_Azambacteria_bacterium_RIFCSPHIGHO2_02_FULL_52_12.OGD32293
Candidatus_Lloydbacteria_bacterium_RIFCSPLOWO2_02_FULL_54_12.OGZ14366
Candidatus_Nomurabacteria_bacterium_GW2011_GWB1_37_5.KKQ34902
Candidatus_Giovannonibacteria_bacterium_RIFCSPHIGHO2_01_FULL_44_42.OGF71847
uncultured_bacterium.EKE11700
Candidatus_Yonathbacteria_bacterium_RIFOXYC2_FULL_47_9.OHA85243
Candidatus_Yonathbacteria_bacterium_RIFCSPLOWO2_01_FULL_47_33b.OHA84088
Candidatus_Andersenbacteria_bacterium_RIFCSPLOWO2_01_FULL_46_36.OGY30665
Elusimicrobia_bacterium_RIFOXYB2_FULL_48_7.OGS24177
Candidatus_Andersenbacteria_bacterium_RIFCSPLOWO2_12_FULL_45_8.OGY38176
Parcubacteria_group_bacterium_GW2011_GWA2_45_14.KKT94073
Deltaproteobacteria_bacterium_CG11_big_fil_rev_8_21_14_0_20_49_13.PIR17896
Gammaproteobacteria_bacterium_RIFCSPHIGHO2_12_FULL_37_14.OGT38472
Gammaproteobacteria_bacterium_RIFCSPHIGHO2_12_FULL_36_30.OGT39229
Gammaproteobacteria_bacterium_SCGC_AG_212_F23.OAI47742
Gammaproteobacteria_bacterium_RIFCSPHIGHO2_12_FULL_40_19.OGT44377
uncultured_bacterium.EKD92204
Gammaproteobacteria_bacterium_RIFCSPHIGHO2_12_FULL_41_15.OGT47779
Candidatus_Magasanikbacteria_bacterium_RIFOXYD2_FULL_41_14.OGH95234
Candidatus_Magasanikbacteria_bacterium_RIFOXYC2_FULL_42_28.OGH88069
bacterium_BMS3Abin15.GBE17295
Candidatus_Uhrbacteria_bacterium_RIFOXYB2_FULL_45_11.OGL98241
Candidatus_Yanofskybacteria_bacterium_RIFCSPHIGHO2_01_FULL_39_8b.OGM99672
Candidatus_Woesebacteria_bacterium_GW2011_GWA1_39_21.KKR09714
Candidatus_Giovannonibacteria_bacterium_GW2011_GWA2_44_13b.KKT41170
Candidatus_Moranbacteria_bacterium_GW2011_GWC2_45_10.KKT89845
Candidatus_Ryanbacteria_bacterium_RIFCSPHIGHO2_01_FULL_45_22.OGZ43586
Parcubacteria_group_bacterium_GW2011_GWC2_38_7.KKQ80169
Parcubacteria_group_bacterium_GW2011_GWA2_40_23.KKR54231
Candidatus_Nomurabacteria_bacterium_RIFCSPHIGHO2_01_FULL_42_16.OGI69009
Microgenomates_group_bacterium_GW2011_GWA2_46_16.KKU25423
Candidatus_Portnoybacteria_bacterium_CG_4_9_14_3_um_filter_44_9.PJA63071
Candidatus_Levybacteria_bacterium_CG10_big_fil_rev_8_21_14_0_10_36_7.PIT88997
Candidatus_Niyogibacteria_bacterium_RIFCSPLOWO2_01_FULL_45_48.OGZ29435
Candidatus_Nomurabacteria_bacterium_RIFCSPHIGHO2_02_FULL_33_12.OGI76335
Candidatus_Kaiserbacteria_bacterium_GWA2_50_9.OGG39735
Candidatus_Yanofskybacteria_bacterium_RIFCSPHIGHO2_12_FULL_45_19b.OGN20070
Candidatus_Nomurabacteria_bacterium_GW2011_GWF1_34_20.KKP60270
Candidatus_Moranbacteria_bacterium_GW2011_GWF1_34_10.KKP58649
Candidatus_Magasanikbacteria_bacterium_CG10_big_fil_rev_8_21_14_0_10_43_6.PIT86737
Candidatus_Moranbacteria_bacterium_CG2_30_41_165.OIQ01696
Parcubacteria_group_bacterium_GW2011_GWB1_35_5.KKP81164
Candidatus_Nomurabacteria_bacterium_CG_4_9_14_0_2_um_filter_32_10.PJC49112
uncultured_bacterium
Elusimicrobia_bacterium_RIFOXYB2_FULL_48_7
Chlorobium_chlorochromatii
Proteobacteria
deltaepsilon_subdivisions
Deltaproteobacteria
Zetaproteobacteria_bacterium_CG2_30_46_52
Sulfurovum_sp_NBC37-1
Deltaproteobacteria_bacterium_CG11_big_fil_rev_8_21_14_0_20_49_13
Deltaproteobacteria_bacterium_CG07_land_8_20_14_0_80_38_7
Gammaproteobacteria
Coxiella_burnetii_RSA_493
Bathymodiolus_azoricus_thioautotrophic_gill_symbiont
Gammaproteobacteria_bacterium_RIFCSPHIGHO2_12_FULL_36_30
Gammaproteobacteria_bacterium_RIFCSPHIGHO2_12_FULL_37_14
Gammaproteobacteria_bacterium_RIFCSPHIGHO2_12_FULL_40_19
Gammaproteobacteria_bacterium_RIFCSPHIGHO2_12_FULL_41_15
Gammaproteobacteria_bacterium_SCGC_AG-212-F23
Bacteria
bacterium__Candidatus_Gribaldobacteria__CG08_land_8_20_14_0_20_39_15
bacterium_CG_4_8_14_3_um_filter_33_28
bacterium_CG2_30_33_46
bacterium_BMS3Abin15
Candidatus_Wirthbacteria_bacterium_CG2_30_54_11
Candidatus_Peregrinibacteria
Candidatus_Peregrinibacteria_bacterium_CG22_combo_CG10-13_8_21_14_all_44_10
Candidatus_Peregrinibacteria_bacterium_CG10_big_fil_rev_8_21_14_0_10_44_7
Candidatus_Peregrinibacteria_bacterium_RIFCSPLOWO2_01_FULL_48_20
Candidatus_Peregrinibacteria_bacterium_GW2011_GWA2_38_36
Candidatus_Peregrinibacteria_bacterium_GW2011_GWA2_43_8
Candidatus_Peregrinibacteria_bacterium_CG_4_9_14_0_2_um_filter_53_11
Candidatus_Gracilibacteria_bacterium_CG17_big_fil_post_rev_8_21_14_2_50_48_13
Candidatus_Pacebacteria_bacterium_CG_4_10_14_0_8_um_filter_42_14
Candidatus_Woesebacteria_bacterium_GW2011_GWA1_39_21
Microgenomates_group_bacterium_GW2011_GWA2_46_16
Microgenomates_group_bacterium_GW2011_GWA1_46_7
Candidatus_Levybacteria
Candidatus_Levybacteria_bacterium_CG_4_10_14_0_2_um_filter_35_8
Candidatus_Levybacteria_bacterium_CG_4_10_14_0_8_um_filter_35_23
Candidatus_Levybacteria_bacterium_CG10_big_fil_rev_8_21_14_0_10_36_7
Candidatus_Ryanbacteria_bacterium_RIFCSPHIGHO2_01_FULL_45_22
Candidatus_Portnoybacteria_bacterium_CG_4_9_14_3_um_filter_44_9
Candidatus_Azambacteria_bacterium_RIFCSPHIGHO2_01_FULL_51_74
Candidatus_Kaiserbacteria_bacterium_GWA2_50_9
Candidatus_Niyogibacteria_bacterium_RIFCSPHIGHO2_01_FULL_45_28
Candidatus_Lloydbacteria_bacterium_RIFCSPLOWO2_02_FULL_54_12
Candidatus_Andersenbacteria
Candidatus_Andersenbacteria_bacterium_RIFCSPHIGHO2_01_FULL_46_36
Candidatus_Andersenbacteria_bacterium_RIFCSPLOWO2_12_FULL_45_8
Candidatus_Yanofskybacteria
Candidatus_Yanofskybacteria_bacterium_RIFCSPHIGHO2_01_FULL_39_8b
Candidatus_Yanofskybacteria_bacterium_RIFCSPHIGHO2_12_FULL_45_19b
Candidatus_Uhrbacteria
Candidatus_Uhrbacteria_bacterium_RIFOXYB2_FULL_45_11
Candidatus_Uhrbacteria_bacterium_CG10_big_fil_rev_8_21_14_0_10_48_16
Candidatus_Giovannonibacteria
Candidatus_Giovannonibacteria_bacterium_RIFCSPHIGHO2_02_43_13
Candidatus_Giovannonibacteria_bacterium_GW2011_GWA2_44_13b
Candidatus_Yonathbacteria
Candidatus_Yonathbacteria_bacterium_RIFCSPLOWO2_01_FULL_47_33b
Candidatus_Yonathbacteria_bacterium_RIFOXYC2_FULL_47_9
Parcubacteria_group_bacterium_GW2011_GWA2_40_23
Parcubacteria_group_bacterium_GW2011_GWA2_45_14
Parcubacteria_group_bacterium_GW2011_GWB1_35_5
Parcubacteria_group_bacterium_GW2011_GWC2_38_7
Candidatus_Moranbacteria
Candidatus_Moranbacteria_bacterium_GW2011_GWC2_37_8
Candidatus_Moranbacteria_bacterium_GW2011_GWC2_45_10
Candidatus_Moranbacteria_bacterium_GW2011_GWF1_34_10
Candidatus_Moranbacteria_bacterium_CG2_30_41_165
Candidatus_Nomurabacteria
Candidatus_Nomurabacteria_bacterium_RIFCSPHIGHO2_01_FULL_42_16
Candidatus_Nomurabacteria_bacterium_RIFCSPHIGHO2_02_FULL_33_12
Candidatus_Nomurabacteria_bacterium_GW2011_GWB1_37_5
Candidatus_Nomurabacteria_bacterium_CG_4_9_14_0_2_um_filter_32_10
Candidatus_Nomurabacteria_bacterium_GW2011_GWE1_35_16
Candidatus_Magasanikbacteria
Candidatus_Magasanikbacteria_bacterium_RIFOXYC2_FULL_42_28
Candidatus_Magasanikbacteria_bacterium_CG11_big_fil_rev_8_21_14_0_20_43_7
Candidatus_Magasanikbacteria_bacterium_RIFOXYD2_FULL_41_14
Candidatus_Magasanikbacteria_bacterium_GW2011_GWC2_42_27
Candidatus_Magasanikbacteria_bacterium_GW2011_GWE2_42_7
Candidatus_Magasanikbacteria_bacterium_CG_4_10_14_0_8_um_filter_42_12
Candidatus_Magasanikbacteria_bacterium_CG10_big_fil_rev_8_21_14_0_10_43_6

CBU_0945

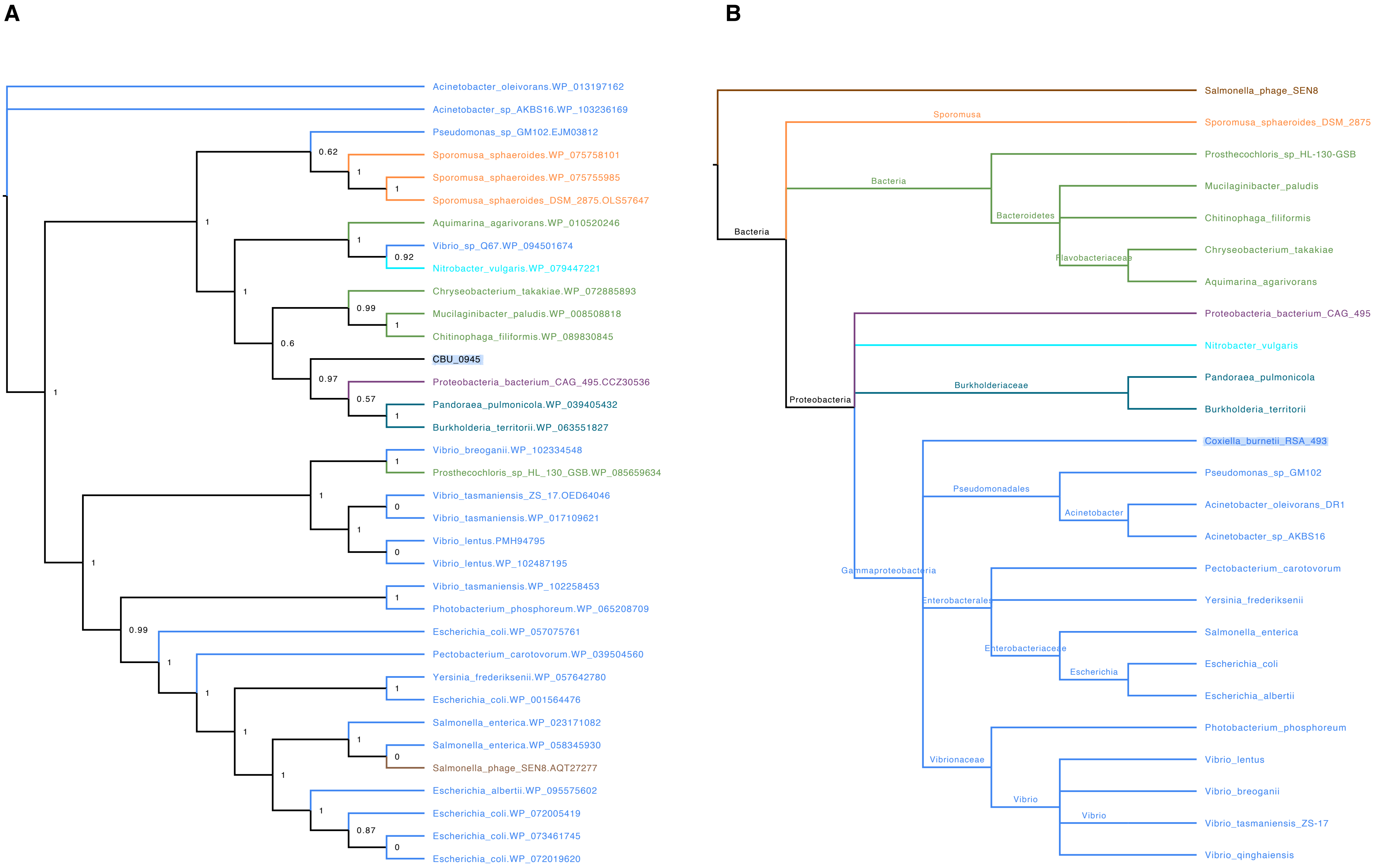
A
Acinetobacter_oleivorans.WP_013197162
Acinetobacter_sp_AKBS16.WP_103236169
Pseudomonas_sp_GM102.EJM03812
Sporomusa_sphaeroides.WP_075758101
Sporomusa_sphaeroides.WP_075755985
Sporomusa_sphaeroides_DSM_2875.OLS57647
Aquimarina_agarivorans.WP_010520246
Vibrio_sp_Q67.WP_094501674
Nitrobacter_vulgaris.WP_079447221
Chryseobacterium_takakiae.WP_072885893
Mucilaginibacter_paludis.WP_008508818
Chitinophaga_filiformis.WP_089830845
CBU_0945
Proteobacteria_bacterium_CAG_495.CCZ30536
Pandoraea_pulmonicola.WP_039405432
Burkholderia_territorii.WP_063551827
Vibrio_breoganii.WP_102334548
Prosthecochloris_sp_HL_130_GSB.WP_085659634
Vibrio_tasmaniensis_ZS_17.OED64046
Vibrio_tasmaniensis.WP_017109621
Vibrio_lentus.PMH94795
Vibrio_lentus.WP_102487195
Vibrio_tasmaniensis.WP_102258453
Photobacterium_phosphoreum.WP_065208709
Escherichia_coli.WP_057075761
Pectobacterium_carotovorum.WP_039504560
Yersinia_frederiksenii.WP_057642780
Escherichia_coli.WP_001564476
Salmonella_enterica.WP_023171082
Salmonella_enterica.WP_058345930
Salmonella_phage_SEN8.AQT27277
Escherichia_albertii.WP_095575602
Escherichia_coli.WP_072005419
Escherichia_coli.WP_073461745
Escherichia_coli.WP_072019620
0.62
0.92
0.99
0.6
0.97
0.57
0.99
0.87
B
Salmonella_phage_SEN8
Sporomusa
Sporomusa_sphaeroides_DSM_2875
Bacteria
Prosthecochloris_sp_HL-130-GSB
Mucilaginibacter_paludis
Bacteroidetes
Chitinophaga_filiformis
Chryseobacterium_takakiae
Flavobacteriaceae
Aquimarina_agarivorans
Proteobacteria_bacterium_CAG_495
Nitrobacter_vulgaris
Burkholderiaceae
Pandoraea_pulmonicola
Proteobacteria
Burkholderia_territorii
Coxiella_burnetii_RSA_493
Pseudomonas_sp_GM102
Pseudomonadales
Acinetobacter_oleivorans_DR1
Acinetobacter
Acinetobacter_sp_AKBS16
Gammaproteobacteria
Pectobacterium_carotovorum
Enterobacterales
Yersinia_frederiksenii
Salmonella_enterica
Enterobacteriaceae
Escherichia_coli
Escherichia
Escherichia_albertii
Photobacterium_phosphoreum
Vibrionaceae
Vibrio_lentus
Vibrio_breoganii
Vibrio
Vibrio_tasmaniensis_ZS-17
Vibrio_qinghaiensis

CBU_0932

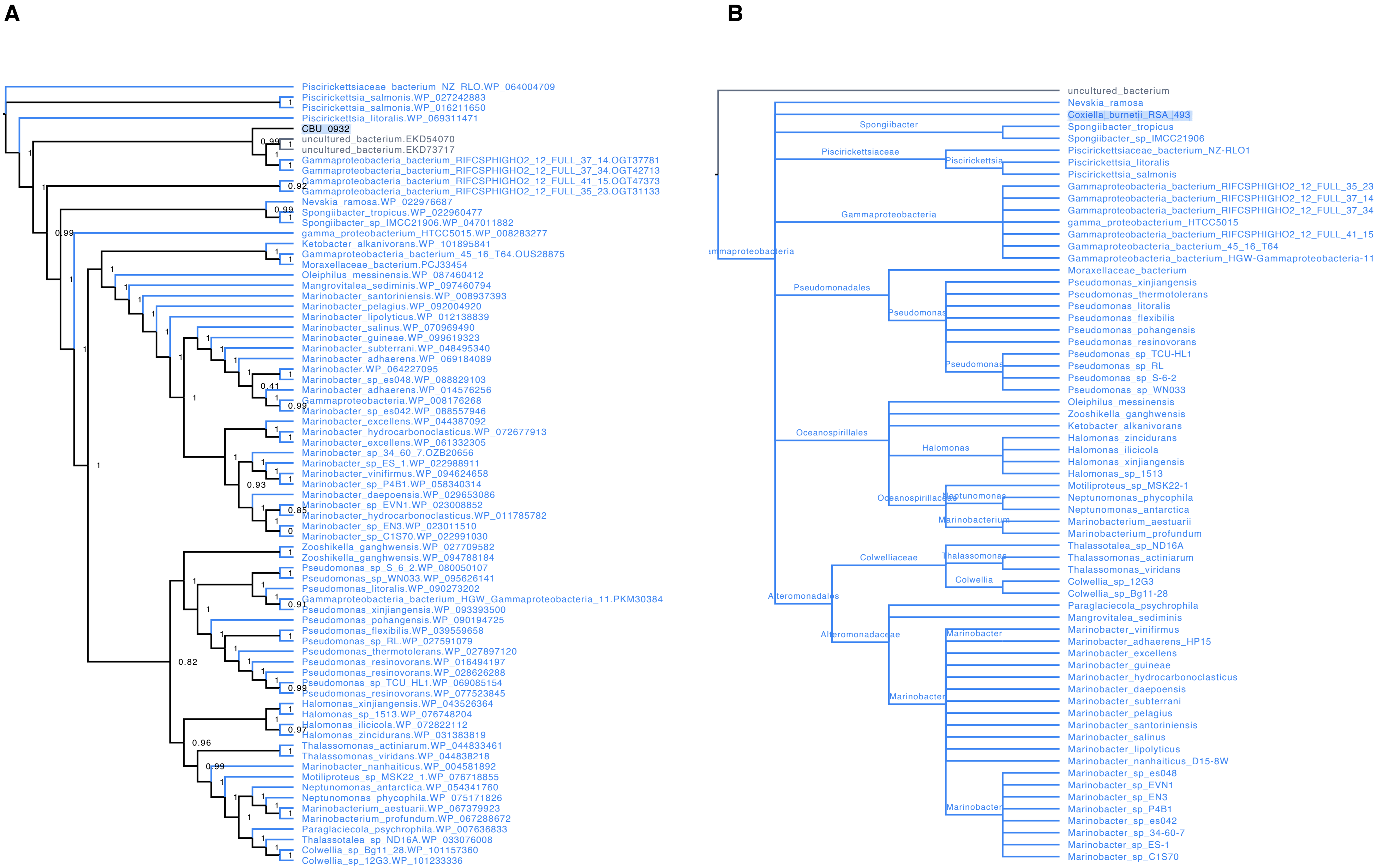

A
Piscirickettsiaceae_bacterium_NZ_RLO.WP_064004709
Piscirickettsia_salmonis.WP_027242883
Piscirickettsia_salmonis.WP_016211650
Piscirickettsia_litoralis.WP_069311471
CBU_0932
uncultured_bacterium.EKD54070
uncultured_bacterium.EKD73717
Gammaproteobacteria_bacterium_RIFCSPHIGHO2_12_FULL_37_14.OGT37781
Gammaproteobacteria_bacterium_RIFCSPHIGHO2_12_FULL_37_34.OGT42713
Gammaproteobacteria_bacterium_RIFCSPHIGHO2_12_FULL_41_15.OGT47373
Gammaproteobacteria_bacterium_RIFCSPHIGHO2_12_FULL_35_23.OGT31133
Nevskia_ramosa.WP_022976687
Spongiibacter_tropicus.WP_022960477
Spongiibacter_sp_IMCC21906.WP_047011882
gamma_proteobacterium_HTCC5015.WP_008283277
Ketobacter_alkanivorans.WP_101895841
Gammaproteobacteria_bacterium_45_16_T64.OUS28875
Moraxellaceae_bacterium.PCJ33454
Oleiphilus_messinensis.WP_087460412
Mangrovitalea_sediminis.WP_097460794
Marinobacter_santoriniensis.WP_008937393
Marinobacter_pelagius.WP_092004920
Marinobacter_lipolyticus.WP_012138839
Marinobacter_salinus.WP_070969490
Marinobacter_guineae.WP_099619323
Marinobacter_subterrani.WP_048495340
Marinobacter_adhaerens.WP_069184089
Marinobacter.WP_064227095
Marinobacter_sp_es048.WP_088829103
Marinobacter_adhaerens.WP_014576256
Gammaproteobacteria.WP_008176268
Marinobacter_sp_es042.WP_088557946
Marinobacter_excellens.WP_044387092
Marinobacter_hydrocarbonoclasticus.WP_072677913
Marinobacter_excellens.WP_061332305
Marinobacter_sp_34_60_7.OZB20656
Marinobacter_sp_ES_1.WP_022988911
Marinobacter_vinifirmus.WP_094624658
Marinobacter_sp_P4B1.WP_058340314
Marinobacter_daepoensis.WP_029653086
Marinobacter_sp_EVN1.WP_023008852
Marinobacter_hydrocarbonoclasticus.WP_011785782
Marinobacter_sp_EN3.WP_023011510
Marinobacter_sp_C1S70.WP_022991030
Zooshikella_ganghwensis.WP_027709582
Zooshikella_ganghwensis.WP_094788184
Pseudomonas_sp_S_6_2.WP_080050107
Pseudomonas_sp_WN033.WP_095626141
Pseudomonas_litoralis.WP_090273202
Gammaproteobacteria_bacterium_HGW_Gammaproteobacteria_11.PKM30384
Pseudomonas_xinjiangensis.WP_093393500
Pseudomonas_pohangensis.WP_090194725
Pseudomonas_flexibilis.WP_039559658
Pseudomonas_sp_RL.WP_027591079
Pseudomonas_thermotolerans.WP_027897120
Pseudomonas_resinovorans.WP_016494197
Pseudomonas_resinovorans.WP_028626288
Pseudomonas_sp_TCU_HL1.WP_069085154
Pseudomonas_resinovorans.WP_077523845
Halomonas_xinjiangensis.WP_043526364
Halomonas_sp_1513.WP_076748204
Halomonas_ilicicola.WP_072822112
Halomonas_zincidurans.WP_031383819
Thalassomonas_actiniarum.WP_044833461
Thalassomonas_viridans.WP_044838218
Marinobacter_nanhaiticus.WP_004581892
Motiliproteus_sp_MSK22_1.WP_076718855
Neptunomonas_antarctica.WP_054341760
Neptunomonas_phycophila.WP_075171826
Marinobacterium_aestuarii.WP_067379923
Marinobacterium_profundum.WP_067288672
Paraglaciecola_psychrophila.WP_007636833
Thalassotalea_sp_ND16A.WP_033076008
Colwellia_sp_Bg11_28.WP_101157360
Colwellia_sp_12G3.WP_101233336
B
uncultured_bacterium
Nevskia_ramosa
Coxiella_burnetii_RSA_493
Spongiibacter
Spongiibacter_tropicus
Spongiibacter_sp_IMCC21906
Piscirickettsiaceae
Piscirickettsia
Piscirickettsiaceae_bacterium_NZ-RLO1
Piscirickettsia_litoralis
Piscirickettsia_salmonis
Gammaproteobacteria
Gammaproteobacteria_bacterium_RIFCSPHIGHO2_12_FULL_35_23
Gammaproteobacteria_bacterium_RIFCSPHIGHO2_12_FULL_37_14
Gammaproteobacteria_bacterium_RIFCSPHIGHO2_12_FULL_37_34
gamma_proteobacterium_HTCC5015
Gammaproteobacteria_bacterium_RIFCSPHIGHO2_12_FULL_41_15
Gammaproteobacteria_bacterium_45_16_T64
Gammaproteobacteria_bacterium_HGW-Gammaproteobacteria-11
Pseudomonadales
Moraxellaceae_bacterium
Pseudomonas
Pseudomonas_xinjiangensis
Pseudomonas_thermotolerans
Pseudomonas_litoralis
Pseudomonas_flexibilis
Pseudomonas_pohangensis
Pseudomonas_resinovorans
Pseudomonas_sp_TCU-HL1
Pseudomonas_sp_RL
Pseudomonas_sp_S-6-2
Pseudomonas_sp_WN033
Oceanospirillales
Oleiphilus_messinensis
Zooshikella_ganghwensis
Ketobacter_alkanivorans
Halomonas
Halomonas_zincidurans
Halomonas_ilicicola
Halomonas_xinjiangensis
Halomonas_sp_1513
Oceanospirillaceae
Neptunomonas
Marinobacterium
Motiliproteus_sp_MSK22-1
Neptunomonas_phycophila
Neptunomonas_antarctica
Marinobacterium_aestuarii
Marinobacterium_profundum
Alteromonadales
Colwelliaceae
Thalassomonas
Colwellia
Thalassotalea_sp_ND16A
Thalassomonas_actiniarum
Thalassomonas_viridans
Colwellia_sp_12G3
Colwellia_sp_Bg11-28
Alteromonadaceae
Paraglaciecola_psychrophila
Mangrovitalea_sediminis
Marinobacter
Marinobacter_vinifirmus
Marinobacter_adhaerens_HP15
Marinobacter_excellens
Marinobacter_guineae
Marinobacter_hydrocarbonoclasticus
Marinobacter_daepoensis
Marinobacter_subterrani
Marinobacter_pelagius
Marinobacter_santoriniensis
Marinobacter_salinus
Marinobacter_lipolyticus
Marinobacter_nanhaiticus_D15-8W
Marinobacter_sp_es048
Marinobacter_sp_EVN1
Marinobacter_sp_EN3
Marinobacter_sp_P4B1
Marinobacter_sp_es042
Marinobacter_sp_34-60-7
Marinobacter_sp_ES-1
Marinobacter_sp_C1S70

CBU_0931

A

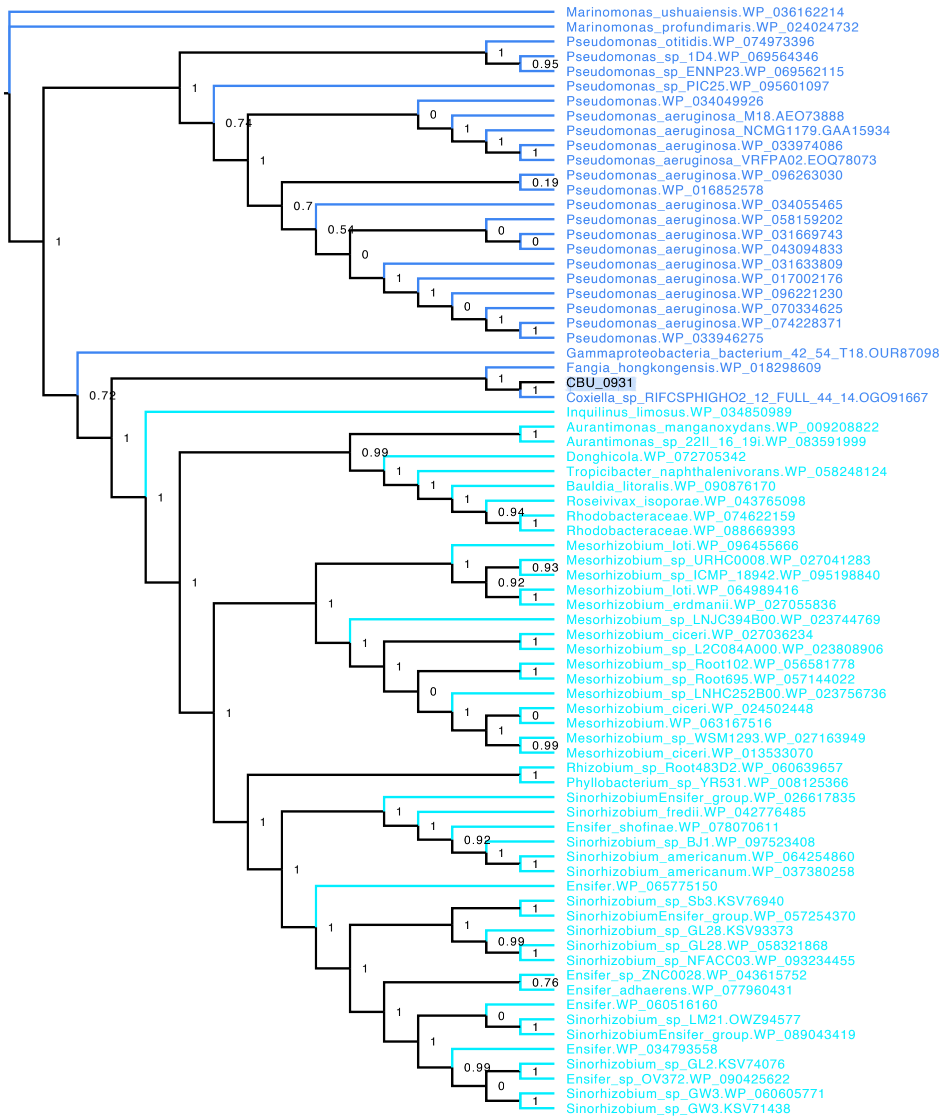

Marinomonas_ushuaiensis.WP_036162214
Marinomonas_profundimaris.WP_024024732
Pseudomonas_otitidis.WP_074973396
Pseudomonas_sp_1D4.WP_069564346
Pseudomonas_sp_ENNP23.WP_069562115
Pseudomonas_sp_PIC25.WP_095601097
Pseudomonas.WP_034049926
Pseudomonas_aeruginosa_M18.AEO73888
Pseudomonas_aeruginosa_NCMG1179.GAA15934
Pseudomonas_aeruginosa.WP_033974086
Pseudomonas_aeruginosa_VRFPA02.EOQ78073
Pseudomonas_aeruginosa.WP_096263030
Pseudomonas.WP_016852578
Pseudomonas_aeruginosa.WP_034055465
Pseudomonas_aeruginosa.WP_058159202
Pseudomonas_aeruginosa.WP_031669743
Pseudomonas_aeruginosa.WP_043094833
Pseudomonas_aeruginosa.WP_031633809
Pseudomonas_aeruginosa.WP_017002176
Pseudomonas_aeruginosa.WP_096221230
Pseudomonas_aeruginosa.WP_070334625
Pseudomonas_aeruginosa.WP_074228371
Pseudomonas.WP_033946275
Gammaproteobacteria_bacterium_42_54_T18.OUR87098
Fangia_hongkongensis.WP_018298609
CBU_0931
Coxiella_sp_RIFCSPHIGHO2_12_FULL_44_14.OGO91667
Inquilinus_limosus.WP_034850989
Aurantimonas_manganoxydans.WP_009208822
Aurantimonas_sp_22II_16_19i.WP_083591999
Donghicola.WP_072705342
Tropicibacter_naphthalenivorans.WP_058248124
Bauldia_litoralis.WP_090876170
Roseivivax_isoporae.WP_043765098
Rhodobacteraceae.WP_074622159
Rhodobacteraceae.WP_088669393
Mesorhizobium_loti.WP_096455666
Mesorhizobium_sp_URHC0008.WP_027041283
Mesorhizobium_sp_ICMP_18942.WP_095198840
Mesorhizobium_loti.WP_064989416
Mesorhizobium_erdmanii.WP_027055836
Mesorhizobium_sp_LNJC394B00.WP_023744769
Mesorhizobium_ciceri.WP_027036234
Mesorhizobium_sp_L2C084A000.WP_023808906
Mesorhizobium_sp_Root102.WP_056581778
Mesorhizobium_sp_Root695.WP_057144022
Mesorhizobium_sp_LNHC252B00.WP_023756736
Mesorhizobium_ciceri.WP_024502448
Mesorhizobium.WP_063167516
Mesorhizobium_sp_WSM1293.WP_027163949
Mesorhizobium_ciceri.WP_013533070
Rhizobium_sp_Root483D2.WP_060639657
Phyllobacterium_sp_YR531.WP_008125366
SinorhizobiumEnsifer_group.WP_026617835
Sinorhizobium_fredii.WP_042776485
Ensifer_shofinae.WP_078070611
Sinorhizobium_sp_BJ1.WP_097523408
Sinorhizobium_americanum.WP_064254860
Sinorhizobium_americanum.WP_037380258
Ensifer.WP_065775150
Sinorhizobium_sp_Sb3.KSV76940
SinorhizobiumEnsifer_group.WP_057254370
Sinorhizobium_sp_GL28.KSV93373
Sinorhizobium_sp_GL28.WP_058321868
Sinorhizobium_sp_NFACC03.WP_093234455
Ensifer_sp_ZNC0028.WP_043615752
Ensifer_adhaerens.WP_077960431
Ensifer.WP_060516160
Sinorhizobium_sp_LM21.OWZ94577
SinorhizobiumEnsifer_group.WP_089043419
Ensifer.WP_034793558
Sinorhizobium_sp_GL2.KSV74076
Ensifer_sp_OV372.WP_090425622
Sinorhizobium_sp_GW3.WP_060605771
Sinorhizobium_sp_GW3.KSV71438

B

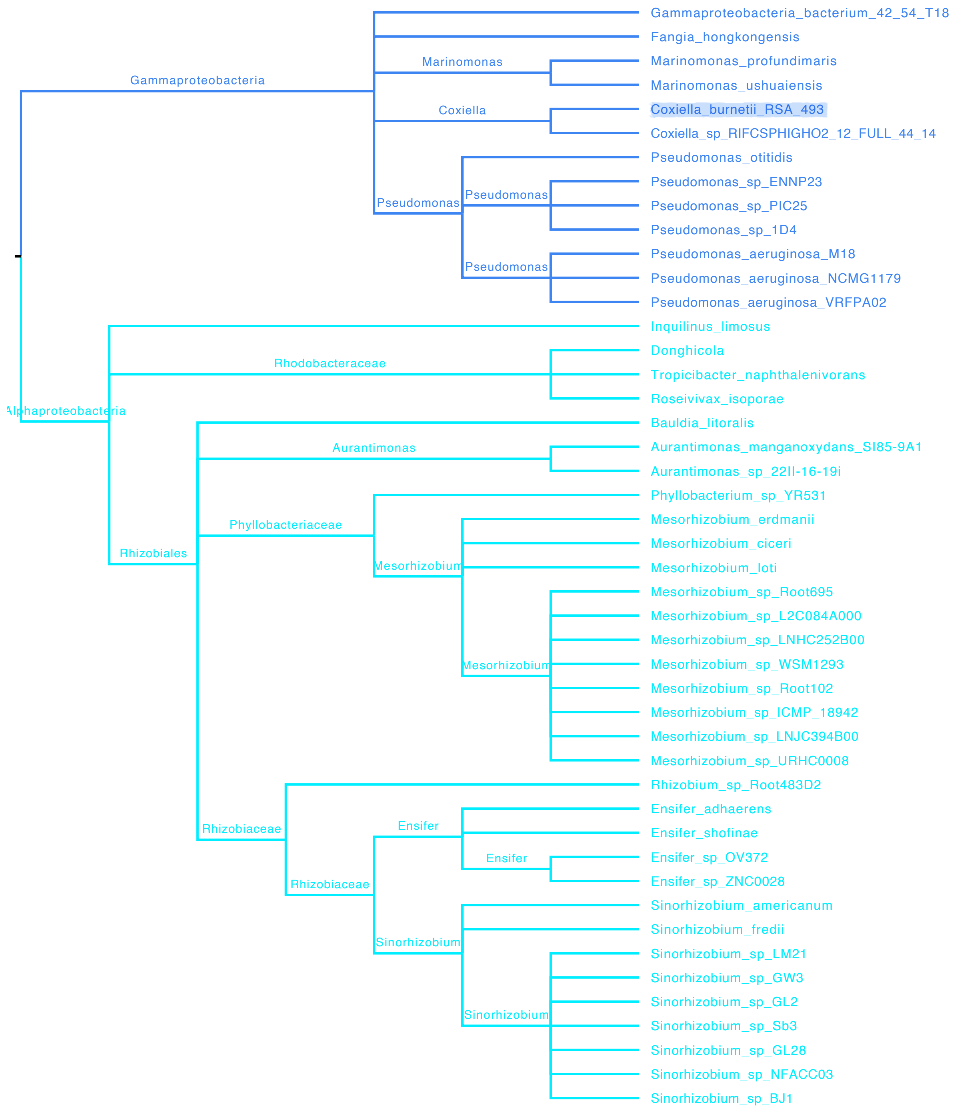

Gammaproteobacteria
Marinomonas
Coxiella
Pseudomonas
Pseudomonas
Pseudomonas
Alphaproteobacteria
Rhodobacteraceae
Aurantimonas
Phyllobacteriaceae
Rhizobiales
Mesorhizobium
Mesorhizobium
Rhizobiaceae
Ensifer
Ensifer
Rhizobiaceae
Sinorhizobium
Sinorhizobium
Gammaproteobacteria_bacterium_42_54_T18
Fangia_hongkongensis
Marinomonas_profundimaris
Marinomonas_ushuaiensis
Coxiella_burnetii_RSA_493
Coxiella_sp_RIFCSPHIGHO2_12_FULL_44_14
Pseudomonas_otitidis
Pseudomonas_sp_ENNP23
Pseudomonas_sp_PIC25
Pseudomonas_sp_1D4
Pseudomonas_aeruginosa_M18
Pseudomonas_aeruginosa_NCMG1179
Pseudomonas_aeruginosa_VRFPA02
Inquilinus_limosus
Donghicola
Tropicibacter_naphthalenivorans
Roseivivax_isoporae
Bauldia_litoralis
Aurantimonas_manganoxydans_SI85-9A1
Aurantimonas_sp_22II-16-19i
Phyllobacterium_sp_YR531
Mesorhizobium_erdmanii
Mesorhizobium_ciceri
Mesorhizobium_loti
Mesorhizobium_sp_Root695
Mesorhizobium_sp_L2C084A000
Mesorhizobium_sp_LNHC252B00
Mesorhizobium_sp_WSM1293
Mesorhizobium_sp_Root102
Mesorhizobium_sp_ICMP_18942
Mesorhizobium_sp_LNJC394B00
Mesorhizobium_sp_URHC0008
Rhizobium_sp_Root483D2
Ensifer_adhaerens
Ensifer_shofinae
Ensifer_sp_OV372
Ensifer_sp_ZNC0028
Sinorhizobium_americanum
Sinorhizobium_fredii
Sinorhizobium_sp_LM21
Sinorhizobium_sp_GW3
Sinorhizobium_sp_GL2
Sinorhizobium_sp_Sb3
Sinorhizobium_sp_GL28
Sinorhizobium_sp_NFACC03
Sinorhizobium_sp_BJ1

CBU_0927

A

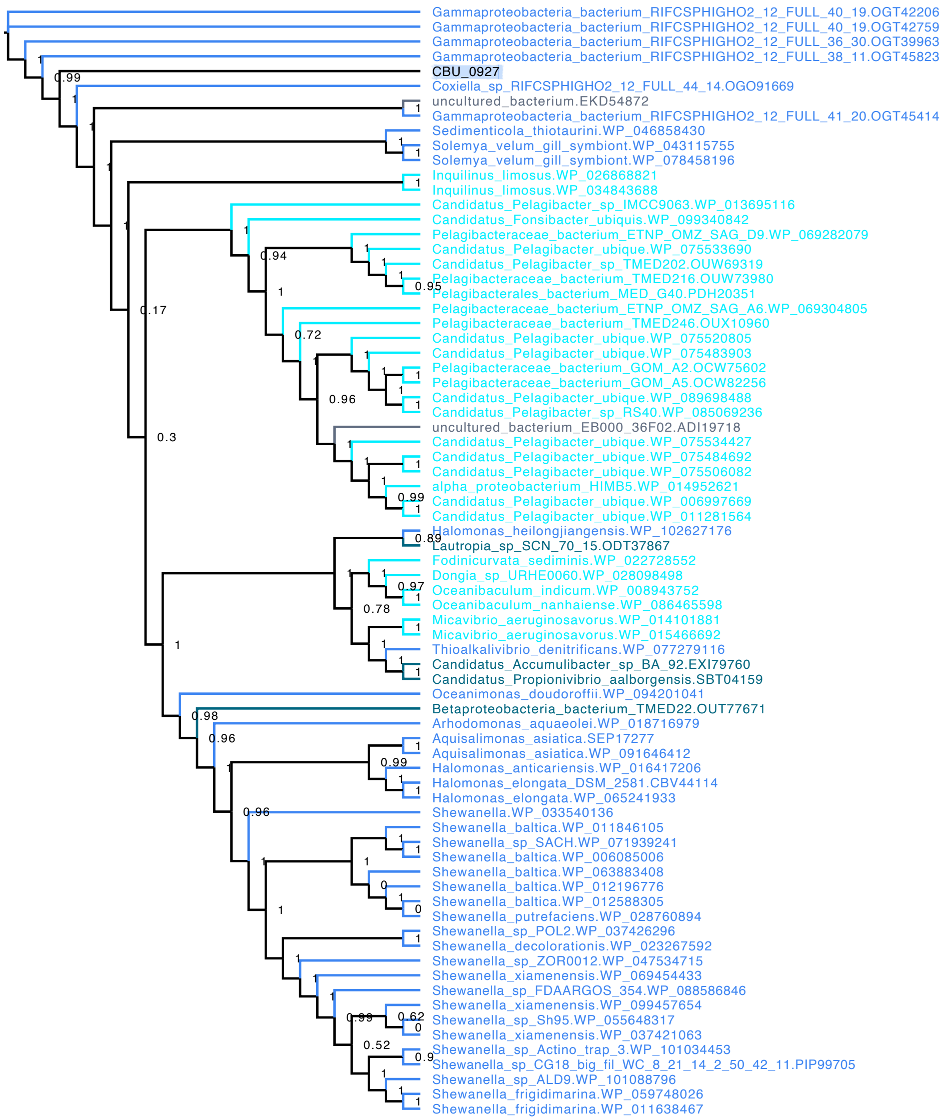

Gammaproteobacteria_bacterium_RIFCSPHIGHO2_12_FULL_40_19.OGT42206
Gammaproteobacteria_bacterium_RIFCSPHIGHO2_12_FULL_40_19.OGT42759
Gammaproteobacteria_bacterium_RIFCSPHIGHO2_12_FULL_36_30.OGT39963
Gammaproteobacteria_bacterium_RIFCSPHIGHO2_12_FULL_38_11.OGT45823
CBU_0927
Coxiella_sp_RIFCSPHIGHO2_12_FULL_44_14.OGO91669
uncultured_bacterium.EKD54872
Gammaproteobacteria_bacterium_RIFCSPHIGHO2_12_FULL_41_20.OGT45414
Sedimenticola_thiotaurini.WP_046858430
Solemya_velum_gill_symbiont.WP_043115755
Solemya_velum_gill_symbiont.WP_078458196
Inquilinus_limosus.WP_026868821
Inquilinus_limosus.WP_034843688
Candidatus_Pelagibacter_sp_IMCC9063.WP_013695116
Candidatus_Fonsibacter_ubiquis.WP_099340842
Pelagibacteraceae_bacterium_ETNP_OMZ_SAG_D9.WP_069282079
Candidatus_Pelagibacter_ubique.WP_075533690
Candidatus_Pelagibacter_sp_TMED202.OUW69319
Pelagibacteraceae_bacterium_TMED216.OUW73980
Pelagibacterales_bacterium_MED_G40.PDH20351
Pelagibacteraceae_bacterium_ETNP_OMZ_SAG_A6.WP_069304805
Pelagibacteraceae_bacterium_TMED246.OUX10960
Candidatus_Pelagibacter_ubique.WP_075520805
Candidatus_Pelagibacter_ubique.WP_075483903
Pelagibacteraceae_bacterium_GOM_A2.OCW75602
Pelagibacteraceae_bacterium_GOM_A5.OCW82256
Candidatus_Pelagibacter_ubique.WP_089698488
Candidatus_Pelagibacter_sp_RS40.WP_085069236
uncultured_bacterium_EB000_36F02.ADI19718
Candidatus_Pelagibacter_ubique.WP_075534427
Candidatus_Pelagibacter_ubique.WP_075484692
Candidatus_Pelagibacter_ubique.WP_075506082
alpha_proteobacterium_HIMB5.WP_014952621
Candidatus_Pelagibacter_ubique.WP_006997669
Candidatus_Pelagibacter_ubique.WP_011281564
Halomonas_heilongjiangensis.WP_102627176
Lautropia_sp_SCN_70_15.ODT37867
Fodinicurvata_sediminis.WP_022728552
Dongia_sp_URHE0060.WP_028098498
Oceanibaculum_indicum.WP_008943752
Oceanibaculum_nanhaiense.WP_086465598
Micavibrio_aeruginosavorus.WP_014101881
Micavibrio_aeruginosavorus.WP_015466692
Thioalkalivibrio_denitrificans.WP_077279116
Candidatus_Accumulibacter_sp_BA_92.EXI79760
Candidatus_Propionivibrio_aalborgensis.SBT04159
Oceanimonas_doudoroffii.WP_094201041
Betaproteobacteria_bacterium_TMED22.OUT77671
Arhodomonas_aquaeolei.WP_018716979
Aquisalimonas_asiatica.SEP17277
Aquisalimonas_asiatica.WP_091646412
Halomonas_anticariensis.WP_016417206
Halomonas_elongata_DSM_2581.CBV44114
Halomonas_elongata.WP_065241933
Shewanella.WP_033540136
Shewanella_baltica.WP_011846105
Shewanella_sp_SACH.WP_071939241
Shewanella_baltica.WP_006085006
Shewanella_baltica.WP_063883408
Shewanella_baltica.WP_012196776
Shewanella_baltica.WP_012588305
Shewanella_putrefaciens.WP_028760894
Shewanella_sp_POL2.WP_037426296
Shewanella_decolorationis.WP_023267592
Shewanella_sp_ZOR0012.WP_047534715
Shewanella_xiamenensis.WP_069454433
Shewanella_sp_FDAARGOS_354.WP_088586846
Shewanella_xiamenensis.WP_099457654
Shewanella_sp_Sh95.WP_055648317
Shewanella_xiamenensis.WP_037421063
Shewanella_sp_Actino_trap_3.WP_101034453
Shewanella_sp_CG18_big_fil_WC_8_21_14_2_50_42_11.PIP99705
Shewanella_sp_ALD9.WP_101088796
Shewanella_frigidimarina.WP_059748026
Shewanella_frigidimarina.WP_011638467

B

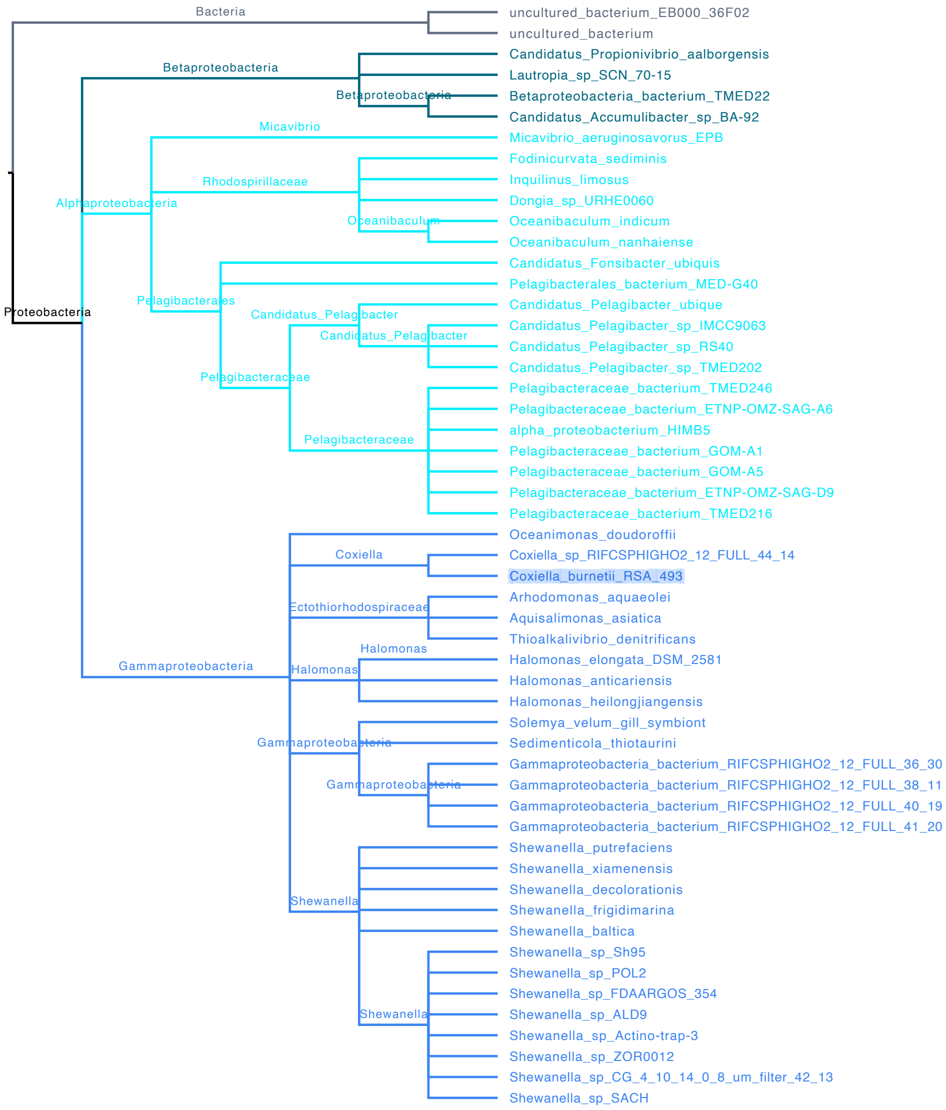

Bacteria
Betaproteobacteria
Micavibrio
Rhodospirillaceae
Alphaproteobacteria
Oceanibaculum
Pelagibacterales
Candidatus_Pelagibacter
Pelagibacteraceae
Proteobacteria
Coxiella
Ectothiorhodospiraceae
Halomonas
Gammaproteobacteria
Shewanella
uncultured_bacterium_EB000_36F02
uncultured_bacterium
Candidatus_Propionivibrio_aalborgensis
Lautropia_sp_SCN_70-15
Betaproteobacteria_bacterium_TMED22
Candidatus_Accumulibacter_sp_BA-92
Micavibrio_aeruginosavorus_EPB
Fodinicurvata_sediminis
Inquilinus_limosus
Dongia_sp_URHE0060
Oceanibaculum_indicum
Oceanibaculum_nanhaiense
Candidatus_Fonsibacter_ubiquis
Pelagibacterales_bacterium_MED-G40
Candidatus_Pelagibacter_ubique
Candidatus_Pelagibacter_sp_IMCC9063
Candidatus_Pelagibacter_sp_RS40
Candidatus_Pelagibacter_sp_TMED202
Pelagibacteraceae_bacterium_TMED246
Pelagibacteraceae_bacterium_ETNP-OMZ-SAG-A6
alpha_proteobacterium_HIMB5
Pelagibacteraceae_bacterium_GOM-A1
Pelagibacteraceae_bacterium_GOM-A5
Pelagibacteraceae_bacterium_ETNP-OMZ-SAG-D9
Pelagibacteraceae_bacterium_TMED216
Oceanimonas_doudoroffii
Coxiella_sp_RIFCSPHIGHO2_12_FULL_44_14
Coxiella_burnetii_RSA_493
Arhodomonas_aquaeolei
Aquisalimonas_asiatica
Thioalkalivibrio_denitrificans
Halomonas_elongata_DSM_2581
Halomonas_anticariensis
Halomonas_heilongjiangensis
Solemya_velum_gill_symbiont
Sedimenticola_thiotaurini
Gammaproteobacteria_bacterium_RIFCSPHIGHO2_12_FULL_36_30
Gammaproteobacteria_bacterium_RIFCSPHIGHO2_12_FULL_38_11
Gammaproteobacteria_bacterium_RIFCSPHIGHO2_12_FULL_40_19
Gammaproteobacteria_bacterium_RIFCSPHIGHO2_12_FULL_41_20
Shewanella_putrefaciens
Shewanella_xiamenensis
Shewanella_decolorationis
Shewanella_frigidimarina
Shewanella_baltica
Shewanella_sp_Sh95
Shewanella_sp_POL2
Shewanella_sp_FDAARGOS_354
Shewanella_sp_ALD9
Shewanella_sp_Actino-trap-3
Shewanella_sp_ZOR0012
Shewanella_sp_CG_4_10_14_0_8_um_filter_42_13
Shewanella_sp_SACH

CBU_0923

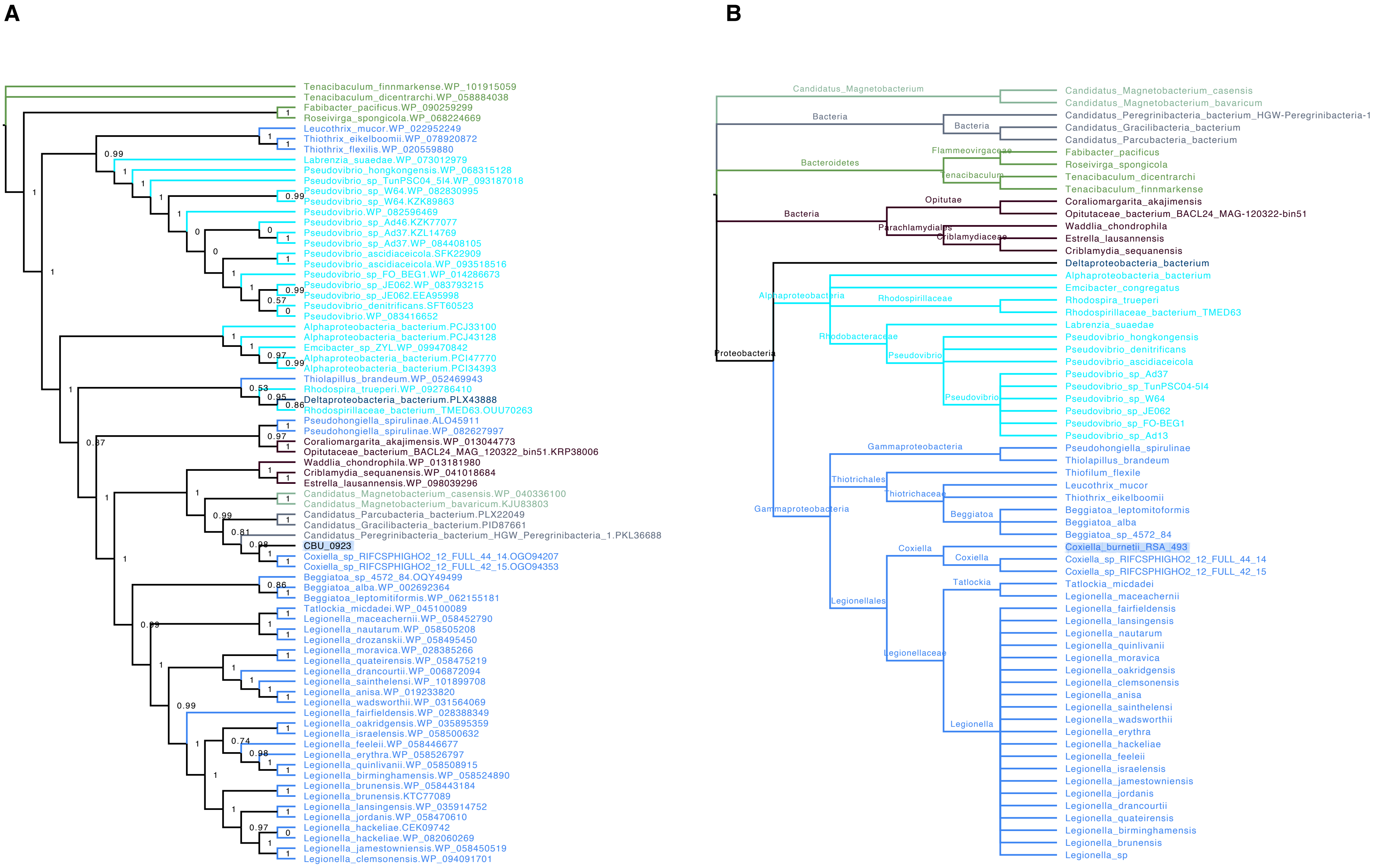
A
Tenacibaculum_finnmarkense.WP_101915059
Tenacibaculum_dicentrarchi.WP_058884038
Fabibacter_pacificus.WP_090259299
Roseivirga_spongicola.WP_068224669
Leucothrix_mucor.WP_022952249
Thiothrix_eikelboomii.WP_078920872
Thiothrix_flexilis.WP_020559880
Labrenzia_suaedae.WP_073012979
Pseudovibrio_hongkongensis.WP_068315128
Pseudovibrio_sp_TunPSC04_5I4.WP_093187018
Pseudovibrio_sp_W64.WP_082830995
Pseudovibrio_sp_W64.KZK89863
Pseudovibrio.WP_082596469
Pseudovibrio_sp_Ad46.KZK77077
Pseudovibrio_sp_Ad37.KZL14769
Pseudovibrio_sp_Ad37.WP_084408105
Pseudovibrio_ascidiaceicola.SFK22909
Pseudovibrio_ascidiaceicola.WP_093518516
Pseudovibrio_sp_FO_BEG1.WP_014286673
Pseudovibrio_sp_JE062.WP_083793215
Pseudovibrio_sp_JE062.EEA95998
Pseudovibrio_denitrificans.SFT60523
Pseudovibrio.WP_083416652
Alphaproteobacteria_bacterium.PCJ33100
Alphaproteobacteria_bacterium.PCJ43128
Emcibacter_sp_ZYL.WP_099470842
Alphaproteobacteria_bacterium.PCI47770
Alphaproteobacteria_bacterium.PCI34393
Thiolapillus_brandeum.WP_052469943
Rhodospira_trueperi.WP_092786410
Deltaproteobacteria_bacterium.PLX43888
Rhodospirillaceae_bacterium_TMED63.OUU70263
Pseudohongiella_spirulinae.ALO45911
Pseudohongiella_spirulinae.WP_082627997
Coraliomargarita_akajimensis.WP_013044773
Opitutaceae_bacterium_BACL24_MAG_120322_bin51.KRP38006
Waddlia_chondrophila.WP_013181980
Criblamydia_sequanensis.WP_041018684
Estrella_lausannensis.WP_098039296
Candidatus_Magnetobacterium_casensis.WP_040336100
Candidatus_Magnetobacterium_bavaricum.KJU83803
Candidatus_Parcubacteria_bacterium.PLX22049
Candidatus_Gracilibacteria_bacterium.PID87661
Candidatus_Peregrinibacteria_bacterium_HGW_Peregrinibacteria_1.PKL36688
CBU_0923
Coxiella_sp_RIFCSPHIGHO2_12_FULL_44_14.OGO94207
Coxiella_sp_RIFCSPHIGHO2_12_FULL_42_15.OGO94353
Beggiatoa_sp_4572_84.OQY49499
Beggiatoa_alba.WP_002692364
Beggiatoa_leptomitiformis.WP_062155181
Tatlockia_micdadei.WP_045100089
Legionella_maceachernii.WP_058452790
Legionella_nautarum.WP_058505208
Legionella_drozanskii.WP_058495450
Legionella_moravica.WP_028385266
Legionella_quateirensis.WP_058475219
Legionella_drancourtii.WP_006872094
Legionella_sainthelensi.WP_101899708
Legionella_anisa.WP_019233820
Legionella_wadsworthii.WP_031564069
Legionella_fairfieldensis.WP_028388349
Legionella_oakridgensis.WP_035895359
Legionella_israelensis.WP_058500632
Legionella_feeleii.WP_058446677
Legionella_erythra.WP_058526797
Legionella_quinlivanii.WP_058508915
Legionella_birminghamensis.WP_058524890
Legionella_brunensis.WP_058443184
Legionella_brunensis.KTC77089
Legionella_lansingensis.WP_035914752
Legionella_jordanis.WP_058470610
Legionella_hackeliae.CEK09742
Legionella_hackeliae.WP_082060269
Legionella_jamestowniensis.WP_058450519
Legionella_clemsonensis.WP_094091701
B
Candidatus_Magnetobacterium
Bacteria
Bacteroidetes
Flammeovirgaceae
Tenacibaculum
Opitutae
Parachlamydiales
Criblamydiaceae
Proteobacteria
Alphaproteobacteria
Rhodospirillaceae
Rhodobacteraceae
Pseudovibrio
Gammaproteobacteria
Thiotrichales
Thiotrichaceae
Beggiatoa
Coxiella
Tatlockia
Legionellales
Legionellaceae
Legionella
Candidatus_Magnetobacterium_casensis
Candidatus_Magnetobacterium_bavaricum
Candidatus_Peregrinibacteria_bacterium_HGW-Peregrinibacteria-1
Candidatus_Gracilibacteria_bacterium
Candidatus_Parcubacteria_bacterium
Fabibacter_pacificus
Roseivirga_spongicola
Tenacibaculum_dicentrarchi
Tenacibaculum_finnmarkense
Coraliomargarita_akajimensis
Opitutaceae_bacterium_BACL24_MAG-120322-bin51
Waddlia_chondrophila
Estrella_lausannensis
Criblamydia_sequanensis
Deltaproteobacteria_bacterium
Alphaproteobacteria_bacterium
Emcibacter_congregatus
Rhodospira_trueperi
Rhodospirillaceae_bacterium_TMED63
Labrenzia_suaedae
Pseudovibrio_hongkongensis
Pseudovibrio_denitrificans
Pseudovibrio_ascidiaceicola
Pseudovibrio_sp_Ad37
Pseudovibrio_sp_TunPSC04-5I4
Pseudovibrio_sp_W64
Pseudovibrio_sp_JE062
Pseudovibrio_sp_FO-BEG1
Pseudovibrio_sp_Ad13
Pseudohongiella_spirulinae
Thiolapillus_brandeum
Thiofilum_flexile
Leucothrix_mucor
Thiothrix_eikelboomii
Beggiatoa_leptomitoformis
Beggiatoa_alba
Beggiatoa_sp_4572_84
Coxiella_burnetii_RSA_493
Coxiella_sp_RIFCSPHIGHO2_12_FULL_44_14
Coxiella_sp_RIFCSPHIGHO2_12_FULL_42_15
Tatlockia_micdadei
Legionella_maceachernii
Legionella_fairfieldensis
Legionella_lansingensis
Legionella_nautarum
Legionella_quinlivanii
Legionella_moravica
Legionella_oakridgensis
Legionella_clemsonensis
Legionella_anisa
Legionella_sainthelensi
Legionella_wadsworthii
Legionella_erythra
Legionella_hackeliae
Legionella_feeleii
Legionella_israelensis
Legionella_jamestowniensis
Legionella_jordanis
Legionella_drancourtii
Legionella_quateirensis
Legionella_birminghamensis
Legionella_brunensis
Legionella_sp

CBU_0921

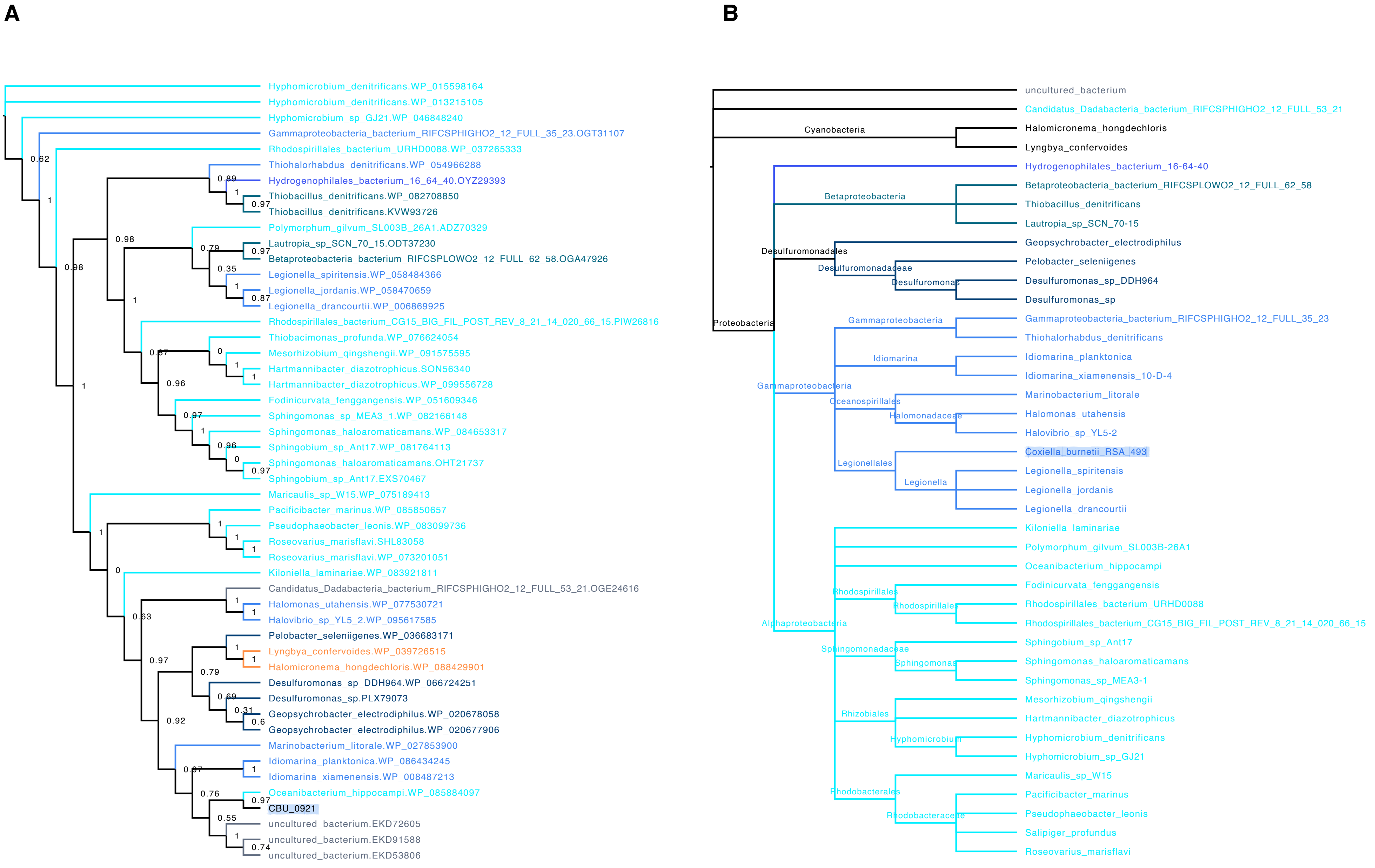
A
Hyphomicrobium_denitrificans.WP_015598164
Hyphomicrobium_denitrificans.WP_013215105
Hyphomicrobium_sp_GJ21.WP_046848240
Gammaproteobacteria_bacterium_RIFCSPHIGHO2_12_FULL_35_23.OGT31107
Rhodospirillales_bacterium_URHD0088.WP_037265333
Thiohalorhabdus_denitrificans.WP_054966288
Hydrogenophilales_bacterium_16_64_40.OYZ29393
Thiobacillus_denitrificans.WP_082708850
Thiobacillus_denitrificans.KVW93726
Polymorphum_gilvum_SL003B_26A1.ADZ70329
Lautropia_sp_SCN_70_15.ODT37230
Betaproteobacteria_bacterium_RIFCSPLOWO2_12_FULL_62_58.OGA47926
Legionella_spiritensis.WP_058484366
Legionella_jordanis.WP_058470659
Legionella_drancourtii.WP_006869925
Rhodospirillales_bacterium_CG15_BIG_FIL_POST_REV_8_21_14_020_66_15.PIW26816
Thiobacimonas_profunda.WP_076624054
Mesorhizobium_qingshengii.WP_091575595
Hartmannibacter_diazotrophicus.SON56340
Hartmannibacter_diazotrophicus.WP_099556728
Fodinicurvata_fenggangensis.WP_051609346
Sphingomonas_sp_MEA3_1.WP_082166148
Sphingomonas_haloaromaticamans.WP_084653317
Sphingobium_sp_Ant17.WP_081764113
Sphingomonas_haloaromaticamans.OHT21737
Sphingobium_sp_Ant17.EXS70467
Maricaulis_sp_W15.WP_075189413
Pacificibacter_marinus.WP_085850657
Pseudophaeobacter_leonis.WP_083099736
Roseovarius_marisflavi.SHL83058
Roseovarius_marisflavi.WP_073201051
Kiloniella_laminariae.WP_083921811
Candidatus_Dadabacteria_bacterium_RIFCSPHIGHO2_12_FULL_53_21.OGE24616
Halomonas_utahensis.WP_077530721
Halovibrio_sp_YL5_2.WP_095617585
Pelobacter_seleniigenes.WP_036683171
Lyngbya_confervoides.WP_039726515
Halomicronema_hongdechloris.WP_088429901
Desulfuromonas_sp_DDH964.WP_066724251
Desulfuromonas_sp.PLX79073
Geopsychrobacter_electrodiphilus.WP_020678058
Geopsychrobacter_electrodiphilus.WP_020677906
Marinobacterium_litorale.WP_027853900
Idiomarina_planktonica.WP_086434245
Idiomarina_xiamenensis.WP_008487213
Oceanibacterium_hippocampi.WP_085884097
CBU_0921
uncultured_bacterium.EKD72605
uncultured_bacterium.EKD91588
uncultured_bacterium.EKD53806
B
uncultured_bacterium
Candidatus_Dadabacteria_bacterium_RIFCSPHIGHO2_12_FULL_53_21
Cyanobacteria
Halomicronema_hongdechloris
Lyngbya_confervoides
Hydrogenophilales_bacterium_16-64-40
Betaproteobacteria
Betaproteobacteria_bacterium_RIFCSPLOWO2_12_FULL_62_58
Thiobacillus_denitrificans
Lautropia_sp_SCN_70-15
Desulfuromonadales
Geopsychrobacter_electrodiphilus
Desulfuromonadaceae
Pelobacter_seleniigenes
Desulfuromonas
Desulfuromonas_sp_DDH964
Desulfuromonas_sp
Proteobacteria
Gammaproteobacteria
Gammaproteobacteria_bacterium_RIFCSPHIGHO2_12_FULL_35_23
Thiohalorhabdus_denitrificans
Idiomarina
Idiomarina_planktonica
Idiomarina_xiamenensis_10-D-4
Gammaproteobacteria
Oceanospirillales
Marinobacterium_litorale
Halomonadaceae
Halomonas_utahensis
Halovibrio_sp_YL5-2
Coxiella_burnetii_RSA_493
Legionellales
Legionella
Legionella_spiritensis
Legionella_jordanis
Legionella_drancourtii
Kiloniella_laminariae
Polymorphum_gilvum_SL003B-26A1
Oceanibacterium_hippocampi
Rhodospirillales
Fodinicurvata_fenggangensis
Rhodospirillales
Rhodospirillales_bacterium_URHD0088
Alphaproteobacteria
Rhodospirillales_bacterium_CG15_BIG_FIL_POST_REV_8_21_14_020_66_15
Sphingomonadaceae
Sphingobium_sp_Ant17
Sphingomonas
Sphingomonas_haloaromaticamans
Sphingomonas_sp_MEA3-1
Rhizobiales
Mesorhizobium_qingshengii
Hartmannibacter_diazotrophicus
Hyphomicrobium
Hyphomicrobium_denitrificans
Hyphomicrobium_sp_GJ21
Rhodobacterales
Maricaulis_sp_W15
Pacificibacter_marinus
Rhodobacteraceae
Pseudophaeobacter_leonis
Salipiger_profundus
Roseovarius_marisflavi

CBU_0920

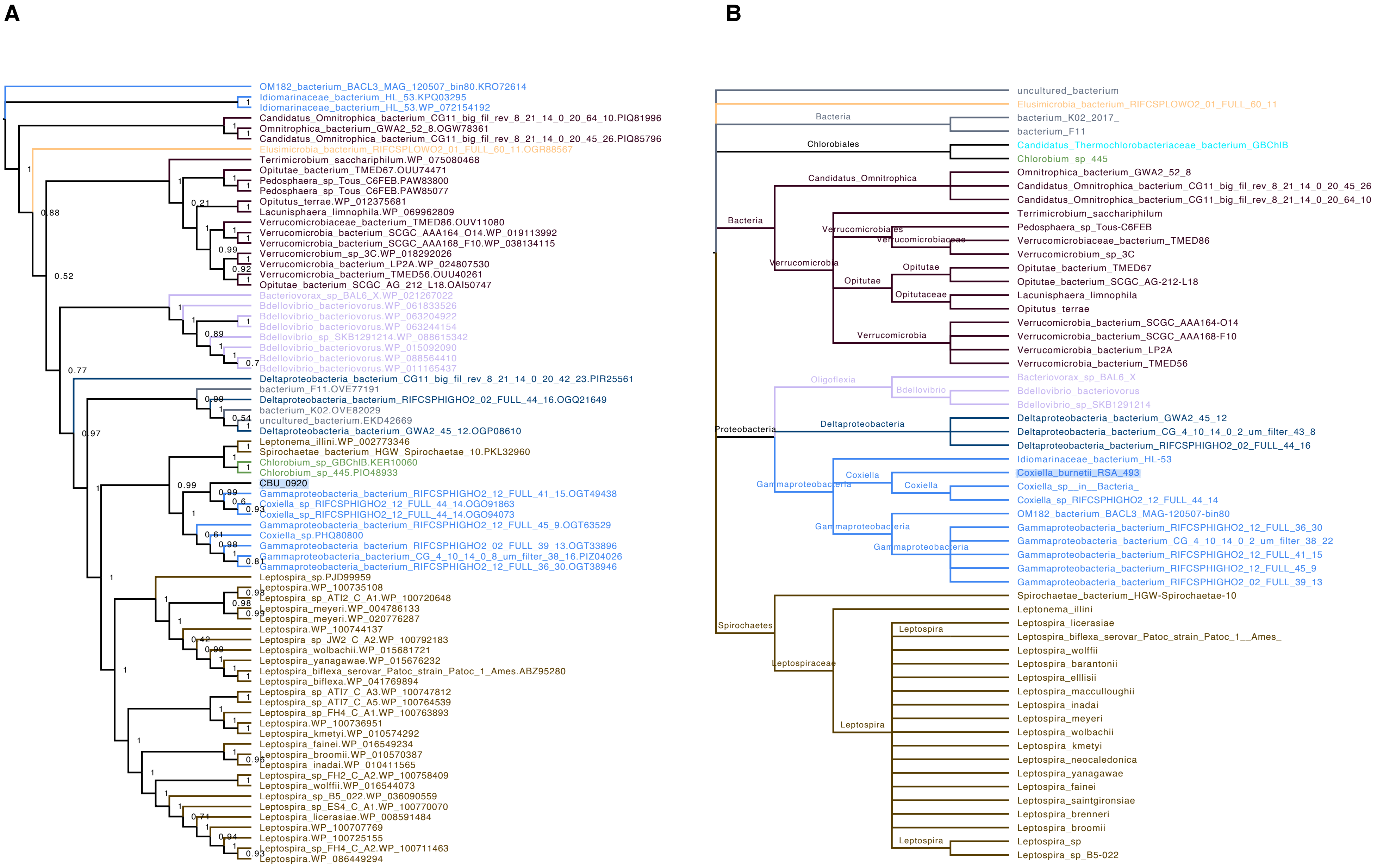

A
OM182_bacterium_BACL3_MAG_120507_bin80.KRO72614
Idiomarinaceae_bacterium_HL_53.KPQ03295
Idiomarinaceae_bacterium_HL_53.WP_072154192
Candidatus_Omnitrophica_bacterium_CG11_big_fil_rev_8_21_14_0_20_64_10.PIQ81996
Omnitrophica_bacterium_GWA2_52_8.OGW78361
Candidatus_Omnitrophica_bacterium_CG11_big_fil_rev_8_21_14_0_20_45_26.PIQ85796
Elusimicrobia_bacterium_RIFCSPLOWO2_01_FULL_60_11.OGR88567
Terrimicrobium_sacchariphilum.WP_075080468
Opitutae_bacterium_TMED67.OUU74471
Pedosphaera_sp_Tous_C6FEB.PAW83800
Pedosphaera_sp_Tous_C6FEB.PAW85077
Opitutus_terrae.WP_012375681
Lacunisphaera_limnophila.WP_069962809
Verrucomicrobiaceae_bacterium_TMED86.OUV11080
Verrucomicrobia_bacterium_SCGC_AAA164_O14.WP_019113992
Verrucomicrobia_bacterium_SCGC_AAA168_F10.WP_038134115
Verrucomicrobium_sp_3C.WP_018292026
Verrucomicrobia_bacterium_LP2A.WP_024807530
Verrucomicrobia_bacterium_TMED56.OUU40261
Opitutae_bacterium_SCGC_AG_212_L18.OAI50747
Bacteriovorax_sp_BAL6_X.WP_021267022
Bdellovibrio_bacteriovorus.WP_061833526
Bdellovibrio_bacteriovorus.WP_063204922
Bdellovibrio_bacteriovorus.WP_063244154
Bdellovibrio_sp_SKB1291214.WP_088615342
Bdellovibrio_bacteriovorus.WP_015092090
Bdellovibrio_bacteriovorus.WP_088564410
Bdellovibrio_bacteriovorus.WP_011165437
Deltaproteobacteria_bacterium_CG11_big_fil_rev_8_21_14_0_20_42_23.PIR25561
bacterium_F11.OVE77191
Deltaproteobacteria_bacterium_RIFCSPHIGHO2_02_FULL_44_16.OGQ21649
bacterium_K02.OVE82029
uncultured_bacterium.EKD42669
Deltaproteobacteria_bacterium_GWA2_45_12.OGP08610
Leptonema_illini.WP_002773346
Spirochaetae_bacterium_HGW_Spirochaetae_10.PKL32960
Chlorobium_sp_GBChlB.KER10060
Chlorobium_sp_445.PIO48933
CBU_0920
Gammaproteobacteria_bacterium_RIFCSPHIGHO2_12_FULL_41_15.OGT49438
Coxiella_sp_RIFCSPHIGHO2_12_FULL_44_14.OGO91863
Coxiella_sp_RIFCSPHIGHO2_12_FULL_44_14.OGO94073
Gammaproteobacteria_bacterium_RIFCSPHIGHO2_12_FULL_45_9.OGT63529
Coxiella_sp.PHQ80800
Gammaproteobacteria_bacterium_RIFCSPHIGHO2_02_FULL_39_13.OGT33896
Gammaproteobacteria_bacterium_CG_4_10_14_0_8_um_filter_38_16.PIZ04026
Gammaproteobacteria_bacterium_RIFCSPHIGHO2_12_FULL_36_30.OGT38946
Leptospira_sp.PJD99959
Leptospira.WP_100735108
Leptospira_sp_ATI2_C_A1.WP_100720648
Leptospira_meyeri.WP_004786133
Leptospira_meyeri.WP_020776287
Leptospira.WP_100744137
Leptospira_sp_JW2_C_A2.WP_100792183
Leptospira_wolbachii.WP_015681721
Leptospira_yanagawae.WP_015676232
Leptospira_biflexa_serovar_Patoc_strain_Patoc_1_Ames.ABZ95280
Leptospira_biflexa.WP_041769894
Leptospira_sp_ATI7_C_A3.WP_100747812
Leptospira_sp_ATI7_C_A5.WP_100764539
Leptospira_sp_FH4_C_A1.WP_100763893
Leptospira.WP_100736951
Leptospira_kmetyi.WP_010574292
Leptospira_fainei.WP_016549234
Leptospira_broomii.WP_010570387
Leptospira_inadai.WP_010411565
Leptospira_sp_FH2_C_A2.WP_100758409
Leptospira_wolffii.WP_016544073
Leptospira_sp_B5_022.WP_036090559
Leptospira_sp_ES4_C_A1.WP_100770070
Leptospira_licerasiae.WP_008591484
Leptospira.WP_100707769
Leptospira.WP_100725155
Leptospira_sp_FH4_C_A2.WP_100711463
Leptospira.WP_086449294
B
uncultured_bacterium
Elusimicrobia_bacterium_RIFCSPLOWO2_01_FULL_60_11
Bacteria
bacterium_K02_2017_
bacterium_F11
Chlorobiales
Candidatus_Thermochlorobacteriaceae_bacterium_GBChlB
Chlorobium_sp_445
Candidatus_Omnitrophica
Omnitrophica_bacterium_GWA2_52_8
Candidatus_Omnitrophica_bacterium_CG11_big_fil_rev_8_21_14_0_20_45_26
Candidatus_Omnitrophica_bacterium_CG11_big_fil_rev_8_21_14_0_20_64_10
Terrimicrobium_sacchariphilum
Verrucomicrobiales
Pedosphaera_sp_Tous-C6FEB
Verrucomicrobiaceae
Verrucomicrobiaceae_bacterium_TMED86
Verrucomicrobium_sp_3C
Verrucomicrobia
Opitutae
Opitutae_bacterium_TMED67
Opitutae_bacterium_SCGC_AG-212-L18
Opitutaceae
Lacunisphaera_limnophila
Opitutus_terrae
Verrucomicrobia_bacterium_SCGC_AAA164-O14
Verrucomicrobia_bacterium_SCGC_AAA168-F10
Verrucomicrobia_bacterium_LP2A
Verrucomicrobia_bacterium_TMED56
Oligoflexia
Bdellovibrio
Bacteriovorax_sp_BAL6_X
Bdellovibrio_bacteriovorus
Bdellovibrio_sp_SKB1291214
Proteobacteria
Deltaproteobacteria
Deltaproteobacteria_bacterium_GWA2_45_12
Deltaproteobacteria_bacterium_CG_4_10_14_0_2_um_filter_43_8
Deltaproteobacteria_bacterium_RIFCSPHIGHO2_02_FULL_44_16
Idiomarinaceae_bacterium_HL-53
Coxiella
Coxiella_burnetii_RSA_493
Gammaproteobacteria
Coxiella_sp__in__Bacteria_
Coxiella_sp_RIFCSPHIGHO2_12_FULL_44_14
OM182_bacterium_BACL3_MAG-120507-bin80
Gammaproteobacteria_bacterium_RIFCSPHIGHO2_12_FULL_36_30
Gammaproteobacteria_bacterium_CG_4_10_14_0_2_um_filter_38_22
Gammaproteobacteria_bacterium_RIFCSPHIGHO2_12_FULL_41_15
Gammaproteobacteria_bacterium_RIFCSPHIGHO2_12_FULL_45_9
Gammaproteobacteria_bacterium_RIFCSPHIGHO2_02_FULL_39_13
Spirochaetes
Spirochaetae_bacterium_HGW-Spirochaetae-10
Leptonema_illini
Leptospiraceae
Leptospira
Leptospira_licerasiae
Leptospira_biflexa_serovar_Patoc_strain_Patoc_1__Ames_
Leptospira_wolffii
Leptospira_barantonii
Leptospira_elllisii
Leptospira_macculloughii
Leptospira_inadai
Leptospira_meyeri
Leptospira_wolbachii
Leptospira_kmetyi
Leptospira_neocaledonica
Leptospira_yanagawae
Leptospira_fainei
Leptospira_saintgironsiae
Leptospira_brenneri
Leptospira_broomii
Leptospira_sp
Leptospira_sp_B5-022

CBU_0916

A

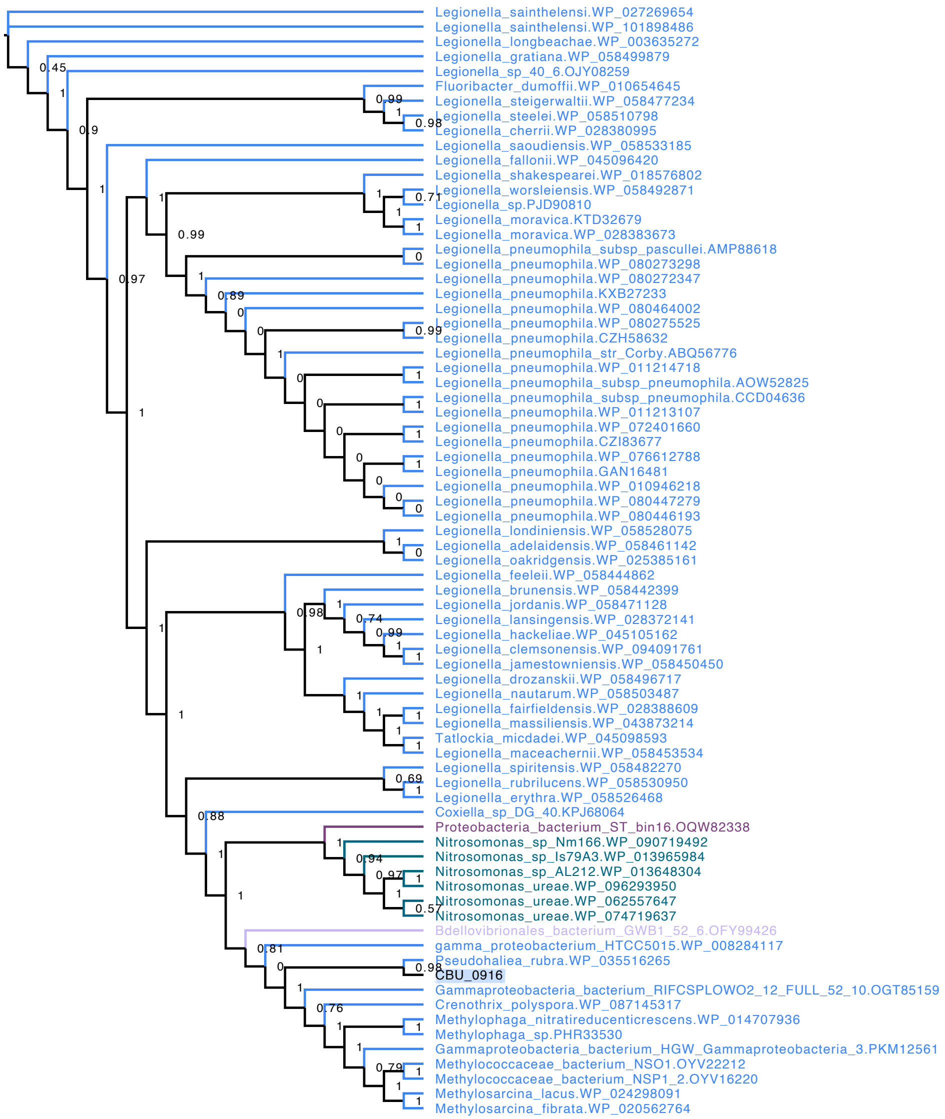
Legionella_sainthelensi.WP_027269654
Legionella_sainthelensi.WP_101898486
Legionella_longbeachae.WP_003635272
Legionella_gratiana.WP_058499879
Legionella_sp_40_6.OJY08259
Fluoribacter_dumoffii.WP_010654645
Legionella_steigerwaltii.WP_058477234
Legionella_steelei.WP_058510798
Legionella_cherrii.WP_028380995
Legionella_saoudiensis.WP_058533185
Legionella_fallonii.WP_045096420
Legionella_shakespearei.WP_018576802
Legionella_worsleiensis.WP_058492871
Legionella_sp.PJD90810
Legionella_moravica.KTD32679
Legionella_moravica.WP_028383673
Legionella_pneumophila_subsp_pascullei.AMP88618
Legionella_pneumophila.WP_080273298
Legionella_pneumophila.WP_080272347
Legionella_pneumophila.KXB27233
Legionella_pneumophila.WP_080464002
Legionella_pneumophila.WP_080275525
Legionella_pneumophila.CZH58632
Legionella_pneumophila_str_Corby.ABQ56776
Legionella_pneumophila.WP_011214718
Legionella_pneumophila_subsp_pneumophila.AOW52825
Legionella_pneumophila_subsp_pneumophila.CCD04636
Legionella_pneumophila.WP_011213107
Legionella_pneumophila.WP_072401660
Legionella_pneumophila.CZI83677
Legionella_pneumophila.WP_076612788
Legionella_pneumophila.GAN16481
Legionella_pneumophila.WP_010946218
Legionella_pneumophila.WP_080447279
Legionella_pneumophila.WP_080446193
Legionella_londiniensis.WP_058528075
Legionella_adelaidensis.WP_058461142
Legionella_oakridgensis.WP_025385161
Legionella_feeleii.WP_058444862
Legionella_brunensis.WP_058442399
Legionella_jordanis.WP_058471128
Legionella_lansingensis.WP_028372141
Legionella_hackeliae.WP_045105162
Legionella_clemsonensis.WP_094091761
Legionella_jamestowniensis.WP_058450450
Legionella_drozanskii.WP_058496717
Legionella_nautarum.WP_058503487
Legionella_fairfieldensis.WP_028388609
Legionella_massiliensis.WP_043873214
Tatlockia_micdadei.WP_045098593
Legionella_maceachernii.WP_058453534
Legionella_spiritensis.WP_058482270
Legionella_rubrilucens.WP_058530950
Legionella_erythra.WP_058526468
Coxiella_sp_DG_40.KPJ68064
Proteobacteria_bacterium_ST_bin16.OQW82338
Nitrosomonas_sp_Nm166.WP_090719492
Nitrosomonas_sp_Is79A3.WP_013965984
Nitrosomonas_sp_AL212.WP_013648304
Nitrosomonas_ureae.WP_096293950
Nitrosomonas_ureae.WP_062557647
Nitrosomonas_ureae.WP_074719637
Bdellovibrionales_bacterium_GWB1_52_6.OFY99426
gamma_proteobacterium_HTCC5015.WP_008284117
Pseudohaliea_rubra.WP_035516265
CBU_0916
Gammaproteobacteria_bacterium_RIFCSPLOWO2_12_FULL_52_10.OGT85159
Crenothrix_polyspora.WP_087145317
Methylophaga_nitratireducenticrescens.WP_014707936
Methylophaga_sp.PHR33530
Gammaproteobacteria_bacterium_HGW_Gammaproteobacteria_3.PKM12561
Methylococcaceae_bacterium_NSO1.OYV22212
Methylococcaceae_bacterium_NSP1_2.OYV16220
Methylosarcina_lacus.WP_024298091
Methylosarcina_fibrata.WP_020562764

B

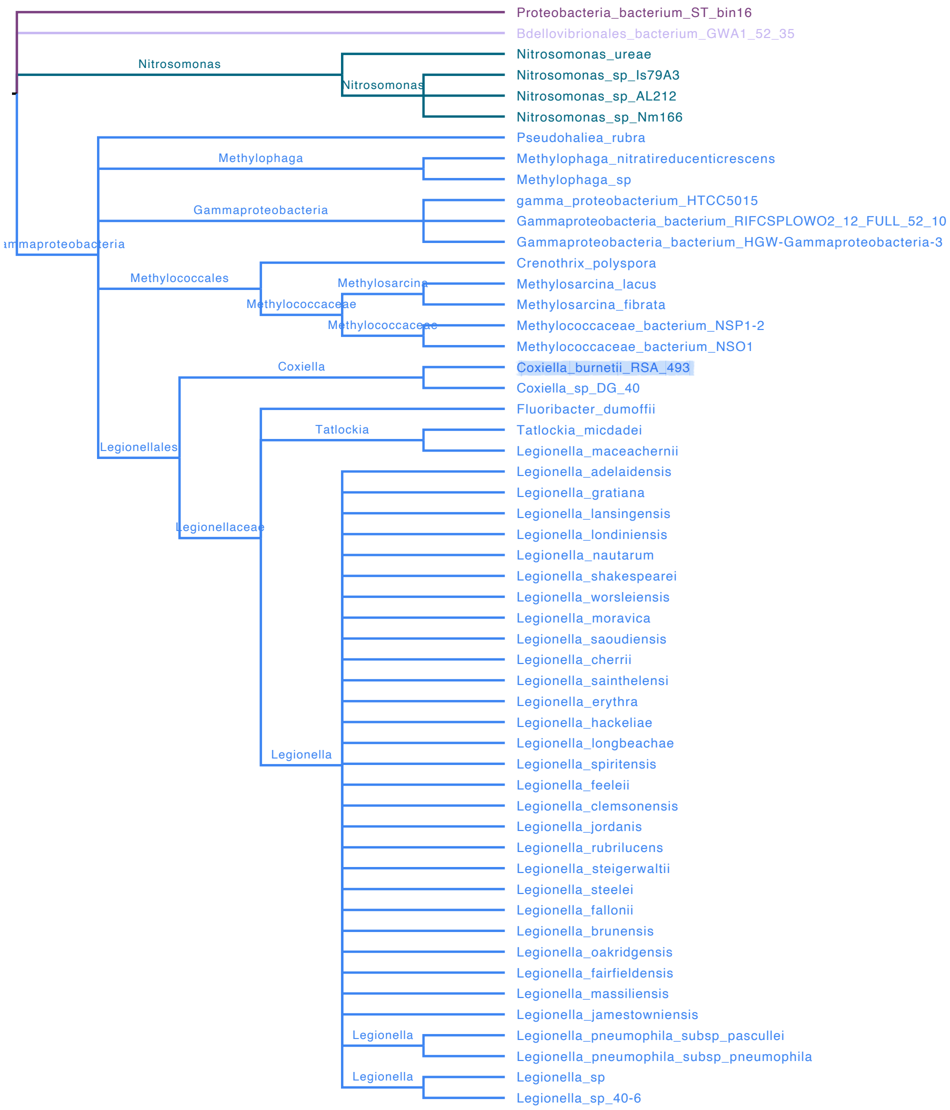
Proteobacteria_bacterium_ST_bin16
Bdellovibrionales_bacterium_GWA1_52_35
Nitrosomonas
Nitrosomonas
Nitrosomonas_ureae
Nitrosomonas_sp_Is79A3
Nitrosomonas_sp_AL212
Nitrosomonas_sp_Nm166
Pseudohaliea_rubra
Methylophaga
Methylophaga_nitratireducenticrescens
Methylophaga_sp
Gammaproteobacteria
gamma_proteobacterium_HTCC5015
Gammaproteobacteria_bacterium_RIFCSPLOWO2_12_FULL_52_10
Gammaproteobacteria_bacterium_HGW-Gammaproteobacteria-3
Crenothrix_polyspora
Methylococcales
Methylosarcina
Methylosarcina_lacus
Methylosarcina_fibrata
Methylococcaceae
Methylococcaceae
Methylococcaceae_bacterium_NSP1-2
Methylococcaceae_bacterium_NSO1
Coxiella
Coxiella_burnetii_RSA_493
Coxiella_sp_DG_40
Fluoribacter_dumoffii
Tatlockia
Tatlockia_micdadei
Legionella_maceachernii
Legionellales
Legionellaceae
Legionella_adelaidensis
Legionella_gratiana
Legionella_lansingensis
Legionella_londiniensis
Legionella_nautarum
Legionella_shakespearei
Legionella_worsleiensis
Legionella_moravica
Legionella_saoudiensis
Legionella_cherrii
Legionella_sainthelensi
Legionella_erythra
Legionella_hackeliae
Legionella_longbeachae
Legionella
Legionella_spiritensis
Legionella_feeleii
Legionella_clemsonensis
Legionella_jordanis
Legionella_rubrilucens
Legionella_steigerwaltii
Legionella_steelei
Legionella_fallonii
Legionella_brunensis
Legionella_oakridgensis
Legionella_fairfieldensis
Legionella_massiliensis
Legionella_jamestowniensis
Legionella
Legionella_pneumophila_subsp_pascullei
Legionella_pneumophila_subsp_pneumophila
Legionella
Legionella_sp
Legionella_sp_40-6

CBU_0912

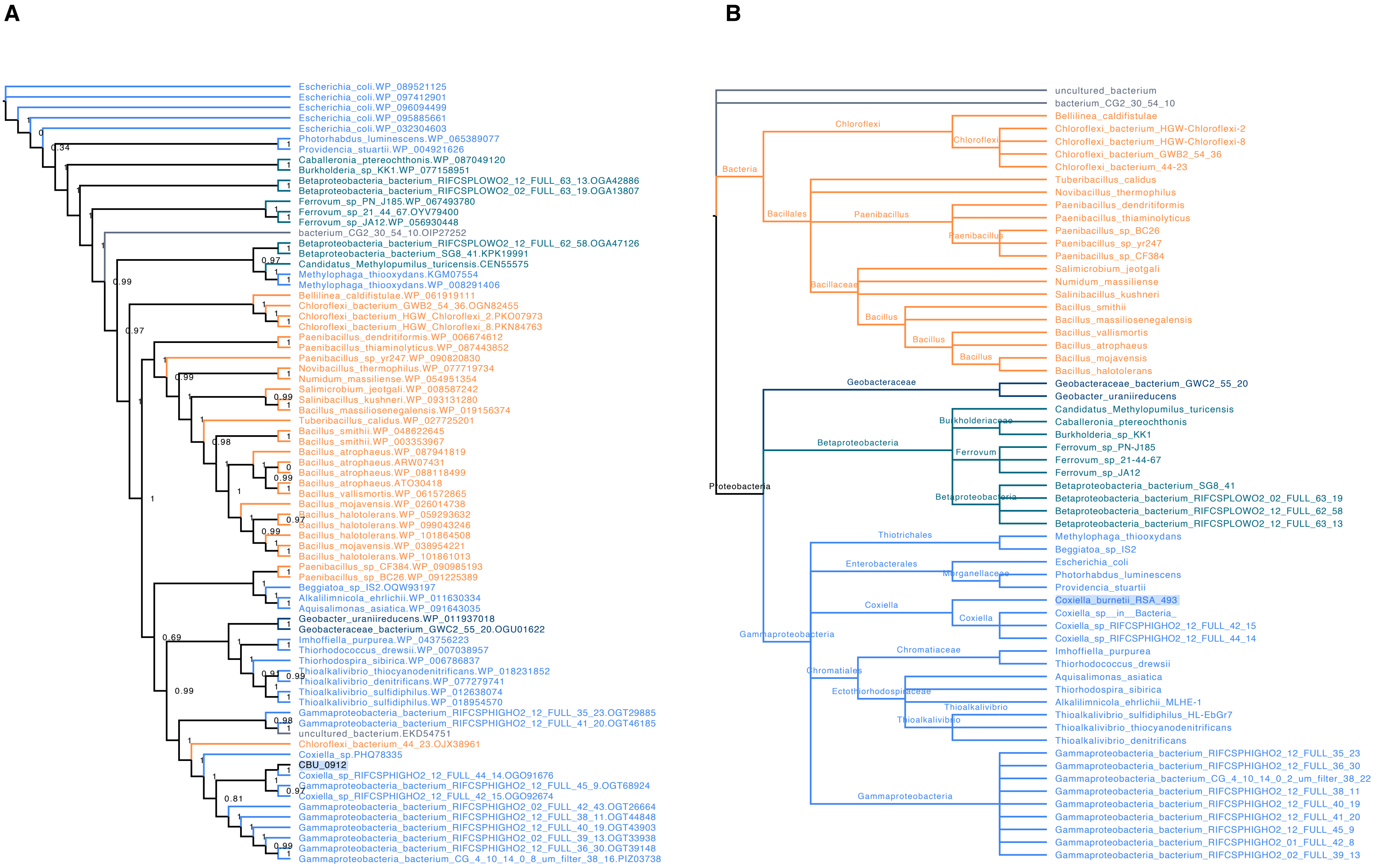
A
Escherichia_coli.WP_089521125
Escherichia_coli.WP_097412901
Escherichia_coli.WP_096094499
Escherichia_coli.WP_095885661
Escherichia_coli.WP_032304603
Photorhabdus_luminescens.WP_065389077
Providencia_stuartii.WP_004921626
Caballeronia_ptereochthonis.WP_087049120
Burkholderia_sp_KK1.WP_077158951
Betaproteobacteria_bacterium_RIFCSPLOWO2_12_FULL_63_13.OGA42886
Betaproteobacteria_bacterium_RIFCSPLOWO2_02_FULL_63_19.OGA13807
Ferrovum_sp_PN_J185.WP_067493780
Ferrovum_sp_21_44_67.OYV79400
Ferrovum_sp_JA12.WP_056930448
bacterium_CG2_30_54_10.OIP27252
Betaproteobacteria_bacterium_RIFCSPLOWO2_12_FULL_62_58.OGA47126
Betaproteobacteria_bacterium_SG8_41.KPK19991
Candidatus_Methylopumilus_turicensis.CEN55575
Methylophaga_thiooxydans.KGM07554
Methylophaga_thiooxydans.WP_008291406
Bellilinea_caldifistulae.WP_061919111
Chloroflexi_bacterium_GWB2_54_36.OGN82455
Chloroflexi_bacterium_HGW_Chloroflexi_2.PKO07973
Chloroflexi_bacterium_HGW_Chloroflexi_8.PKN84763
Paenibacillus_dendritiformis.WP_006674612
Paenibacillus_thiaminolyticus.WP_087443852
Paenibacillus_sp_yr247.WP_090820830
Novibacillus_thermophilus.WP_077719734
Numidum_massiliense.WP_054951354
Salimicrobium_jeotgali.WP_008587242
Salinibacillus_kushneri.WP_093131280
Bacillus_massiliosenegalensis.WP_019156374
Tuberibacillus_calidus.WP_027725201
Bacillus_smithii.WP_048622645
Bacillus_smithii.WP_003353967
Bacillus_atrophaeus.WP_087941819
Bacillus_atrophaeus.ARW07431
Bacillus_atrophaeus.WP_088118499
Bacillus_atrophaeus.ATO30418
Bacillus_vallismortis.WP_061572865
Bacillus_mojavensis.WP_026014738
Bacillus_halotolerans.WP_059293632
Bacillus_halotolerans.WP_099043246
Bacillus_halotolerans.WP_101864508
Bacillus_mojavensis.WP_038954221
Bacillus_halotolerans.WP_101861013
Paenibacillus_sp_CF384.WP_090985193
Paenibacillus_sp_BC26.WP_091225389
Beggiatoa_sp_IS2.OQW93197
Alkalilimnicola_ehrlichii.WP_011630334
Aquisalimonas_asiatica.WP_091643035
Geobacter_uraniireducens.WP_011937018
Geobacteraceae_bacterium_GWC2_55_20.OGU01622
Imhoffiella_purpurea.WP_043756223
Thiorhodococcus_drewsii.WP_007038957
Thiorhodospira_sibirica.WP_006786837
Thioalkalivibrio_thiocyanodenitrificans.WP_018231852
Thioalkalivibrio_denitrificans.WP_077279741
Thioalkalivibrio_sulfidiphilus.WP_012638074
Thioalkalivibrio_sulfidiphilus.WP_018954570
Gammaproteobacteria_bacterium_RIFCSPHIGHO2_12_FULL_35_23.OGT29885
Gammaproteobacteria_bacterium_RIFCSPHIGHO2_12_FULL_41_20.OGT46185
uncultured_bacterium.EKD54751
Chloroflexi_bacterium_44_23.OJX38961
Coxiella_sp.PHQ78335
CBU_0912
Coxiella_sp_RIFCSPHIGHO2_12_FULL_44_14.OGO91676
Gammaproteobacteria_bacterium_RIFCSPHIGHO2_12_FULL_45_9.OGT68924
Coxiella_sp_RIFCSPHIGHO2_12_FULL_42_15.OGO92674
Gammaproteobacteria_bacterium_RIFCSPHIGHO2_02_FULL_42_43.OGT26664
Gammaproteobacteria_bacterium_RIFCSPHIGHO2_12_FULL_38_11.OGT44848
Gammaproteobacteria_bacterium_RIFCSPHIGHO2_12_FULL_40_19.OGT43903
Gammaproteobacteria_bacterium_RIFCSPHIGHO2_02_FULL_39_13.OGT33938
Gammaproteobacteria_bacterium_RIFCSPHIGHO2_12_FULL_36_30.OGT39148
Gammaproteobacteria_bacterium_CG_4_10_14_0_8_um_filter_38_16.PIZ03738
B
uncultured_bacterium
bacterium_CG2_30_54_10
Bacteria
Chloroflexi
Bellilinea_caldifistulae
Chloroflexi_bacterium_HGW-Chloroflexi-2
Chloroflexi_bacterium_HGW-Chloroflexi-8
Chloroflexi_bacterium_GWB2_54_36
Chloroflexi_bacterium_44-23
Bacillales
Tuberibacillus_calidus
Novibacillus_thermophilus
Paenibacillus
Paenibacillus_dendritiformis
Paenibacillus_thiaminolyticus
Paenibacillus_sp_BC26
Paenibacillus_sp_yr247
Paenibacillus_sp_CF384
Bacillaceae
Salimicrobium_jeotgali
Numidum_massiliense
Salinibacillus_kushneri
Bacillus
Bacillus_smithii
Bacillus_massiliosenegalensis
Bacillus_vallismortis
Bacillus_atrophaeus
Bacillus_mojavensis
Bacillus_halotolerans
Proteobacteria
Geobacteraceae
Geobacteraceae_bacterium_GWC2_55_20
Geobacter_uraniireducens
Betaproteobacteria
Burkholderiaceae
Candidatus_Methylopumilus_turicensis
Caballeronia_ptereochthonis
Burkholderia_sp_KK1
Ferrovum
Ferrovum_sp_PN-J185
Ferrovum_sp_21-44-67
Ferrovum_sp_JA12
Betaproteobacteria_bacterium_SG8_41
Betaproteobacteria_bacterium_RIFCSPLOWO2_02_FULL_63_19
Betaproteobacteria_bacterium_RIFCSPLOWO2_12_FULL_62_58
Betaproteobacteria_bacterium_RIFCSPLOWO2_12_FULL_63_13
Gammaproteobacteria
Thiotrichales
Methylophaga_thiooxydans
Beggiatoa_sp_IS2
Enterobacterales
Morganellaceae
Escherichia_coli
Photorhabdus_luminescens
Providencia_stuartii
Coxiella
Coxiella_burnetii_RSA_493
Coxiella_sp__in__Bacteria_
Coxiella_sp_RIFCSPHIGHO2_12_FULL_42_15
Coxiella_sp_RIFCSPHIGHO2_12_FULL_44_14
Chromatiales
Chromatiaceae
Imhoffiella_purpurea
Thiorhodococcus_drewsii
Ectothiorhodospiraceae
Aquisalimonas_asiatica
Thiorhodospira_sibirica
Alkalilimnicola_ehrlichii_MLHE-1
Thioalkalivibrio
Thioalkalivibrio_sulfidiphilus_HL-EbGr7
Thioalkalivibrio_thiocyanodenitrificans
Thioalkalivibrio_denitrificans
Gammaproteobacteria_bacterium_RIFCSPHIGHO2_12_FULL_35_23
Gammaproteobacteria_bacterium_RIFCSPHIGHO2_12_FULL_36_30
Gammaproteobacteria_bacterium_CG_4_10_14_0_2_um_filter_38_22
Gammaproteobacteria_bacterium_RIFCSPHIGHO2_12_FULL_38_11
Gammaproteobacteria_bacterium_RIFCSPHIGHO2_12_FULL_40_19
Gammaproteobacteria_bacterium_RIFCSPHIGHO2_12_FULL_41_20
Gammaproteobacteria_bacterium_RIFCSPHIGHO2_12_FULL_45_9
Gammaproteobacteria_bacterium_RIFCSPHIGHO2_01_FULL_42_8
Gammaproteobacteria_bacterium_RIFCSPHIGHO2_02_FULL_39_13

CBU_0837

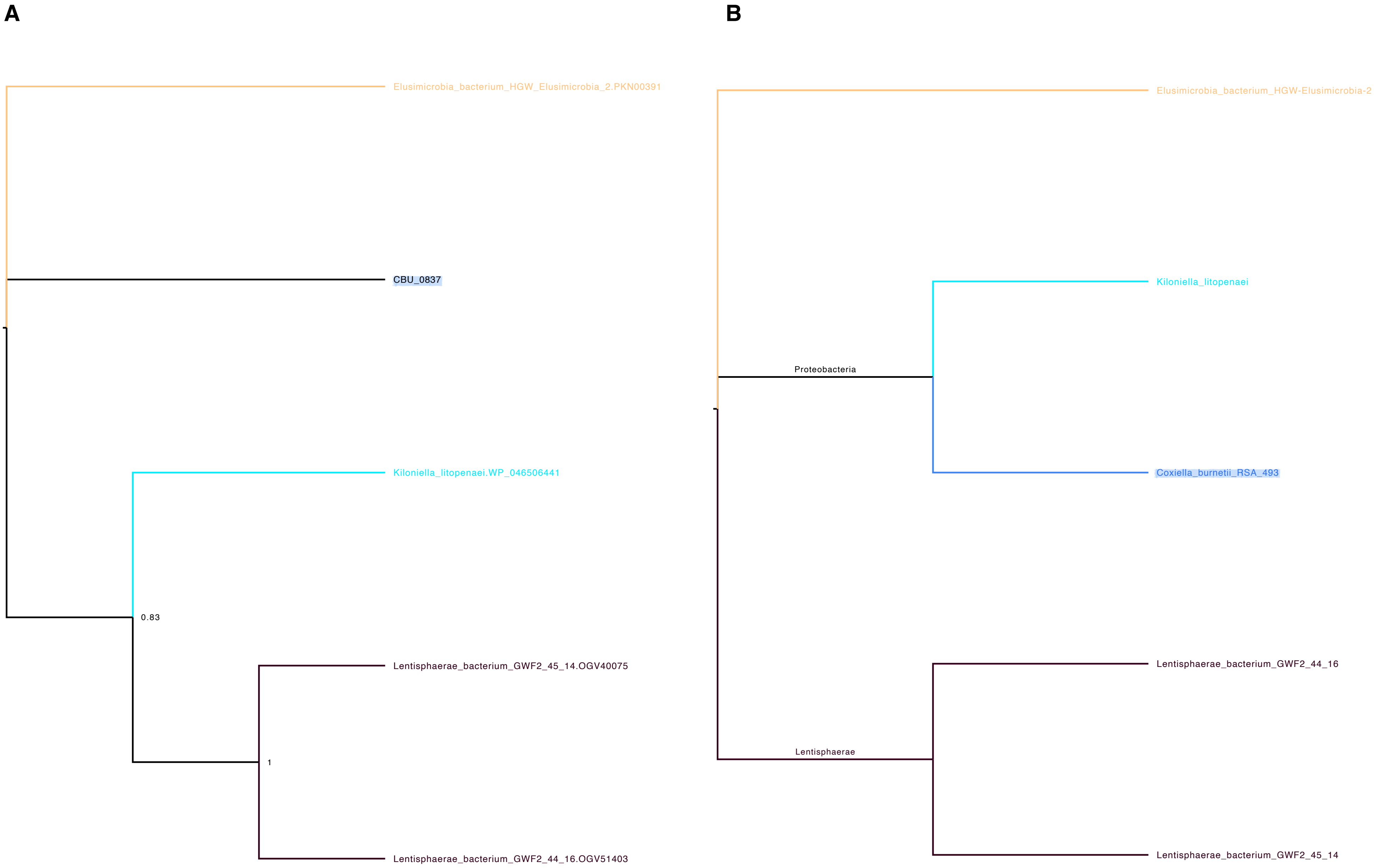
A
Elusimicrobia_bacterium_HGW_Elusimicrobia_2.PKN00391
CBU_0837
Kiloniella_litopenaei.WP_046506441
0.83
Lentisphaerae_bacterium_GWF2_45_14.OGV40075
1
Lentisphaerae_bacterium_GWF2_44_16.OGV51403
B
Elusimicrobia_bacterium_HGW-Elusimicrobia-2
Kiloniella_litopenaei
Proteobacteria
Coxiella_burnetii_RSA_493
Lentisphaerae_bacterium_GWF2_44_16
Lentisphaerae
Lentisphaerae_bacterium_GWF2_45_14

CBU_0819

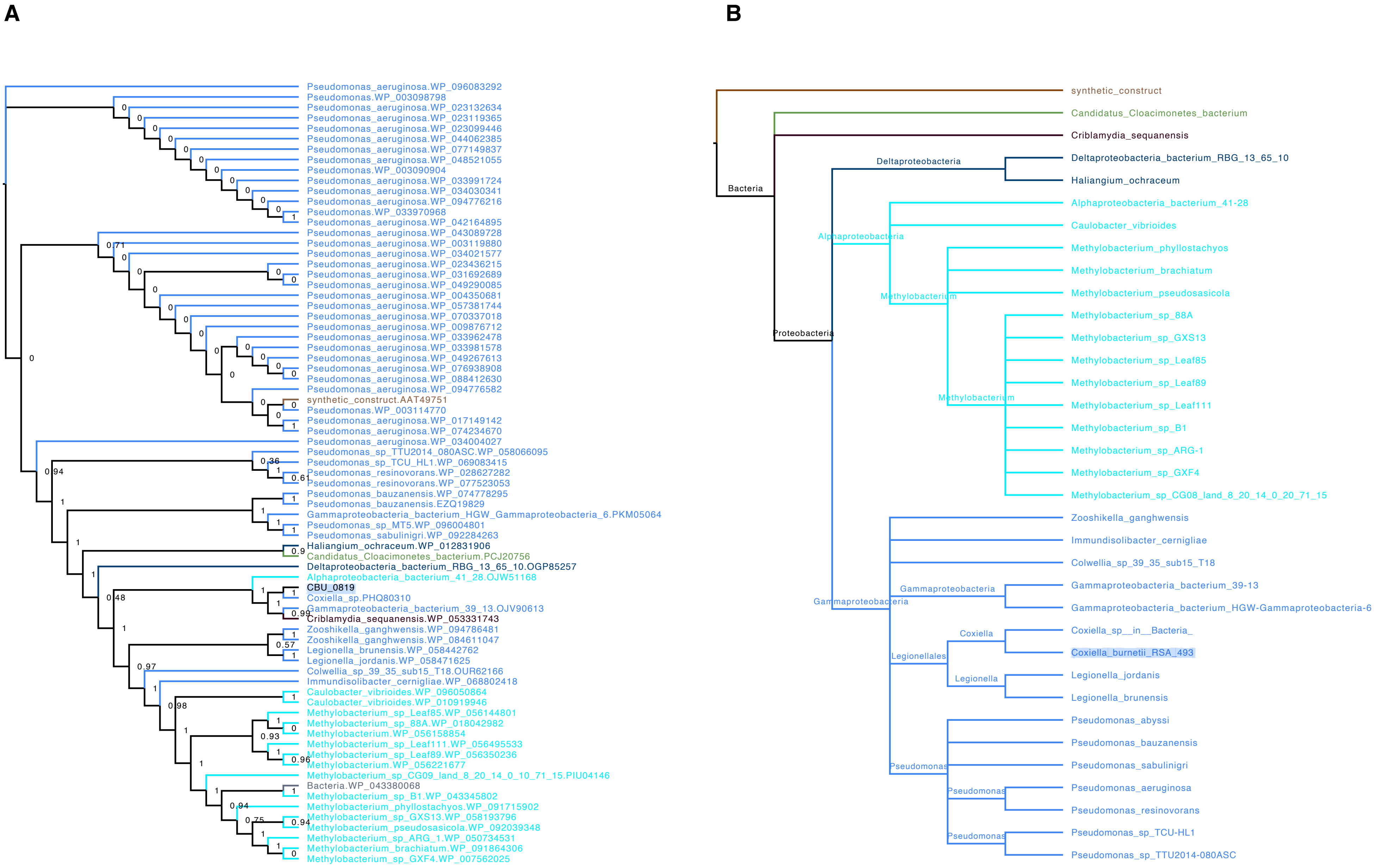
A
Pseudomonas_aeruginosa.WP_096083292
Pseudomonas.WP_003098798
Pseudomonas_aeruginosa.WP_023132634
Pseudomonas_aeruginosa.WP_023119365
Pseudomonas_aeruginosa.WP_023099446
Pseudomonas_aeruginosa.WP_044062385
Pseudomonas_aeruginosa.WP_077149837
Pseudomonas_aeruginosa.WP_048521055
Pseudomonas.WP_003090904
Pseudomonas_aeruginosa.WP_033991724
Pseudomonas_aeruginosa.WP_034030341
Pseudomonas_aeruginosa.WP_094776216
Pseudomonas.WP_033970968
Pseudomonas_aeruginosa.WP_042164895
Pseudomonas_aeruginosa.WP_043089728
Pseudomonas_aeruginosa.WP_003119880
Pseudomonas_aeruginosa.WP_034021577
Pseudomonas_aeruginosa.WP_023436215
Pseudomonas_aeruginosa.WP_031692689
Pseudomonas_aeruginosa.WP_049290085
Pseudomonas_aeruginosa.WP_004350681
Pseudomonas_aeruginosa.WP_057381744
Pseudomonas_aeruginosa.WP_070337018
Pseudomonas_aeruginosa.WP_009876712
Pseudomonas_aeruginosa.WP_033962478
Pseudomonas_aeruginosa.WP_033981578
Pseudomonas_aeruginosa.WP_049267613
Pseudomonas_aeruginosa.WP_076938908
Pseudomonas_aeruginosa.WP_088412630
Pseudomonas_aeruginosa.WP_094776582
synthetic_construct.AAT49751
Pseudomonas.WP_003114770
Pseudomonas_aeruginosa.WP_017149142
Pseudomonas_aeruginosa.WP_074234670
Pseudomonas_aeruginosa.WP_034004027
Pseudomonas_sp_TTU2014_080ASC.WP_058066095
Pseudomonas_sp_TCU_HL1.WP_069083415
Pseudomonas_resinovorans.WP_028627282
Pseudomonas_resinovorans.WP_077523053
Pseudomonas_bauzanensis.WP_074778295
Pseudomonas_bauzanensis.EZQ19829
Gammaproteobacteria_bacterium_HGW_Gammaproteobacteria_6.PKM05064
Pseudomonas_sp_MT5.WP_096004801
Pseudomonas_sabulinigri.WP_092284263
Haliangium_ochraceum.WP_012831906
Candidatus_Cloacimonetes_bacterium.PCJ20756
Deltaproteobacteria_bacterium_RBG_13_65_10.OGP85257
Alphaproteobacteria_bacterium_41_28.OJW51168
CBU_0819
Coxiella_sp.PHQ80310
Gammaproteobacteria_bacterium_39_13.OJV90613
Criblamydia_sequanensis.WP_053331743
Zooshikella_ganghwensis.WP_094786481
Zooshikella_ganghwensis.WP_084611047
Legionella_brunensis.WP_058442762
Legionella_jordanis.WP_058471625
Colwellia_sp_39_35_sub15_T18.OUR62166
Immundisolibacter_cernigliae.WP_068802418
Caulobacter_vibrioides.WP_096050864
Caulobacter_vibrioides.WP_010919946
Methylobacterium_sp_Leaf85.WP_056144801
Methylobacterium_sp_88A.WP_018042982
Methylobacterium.WP_056158854
Methylobacterium_sp_Leaf111.WP_056495533
Methylobacterium_sp_Leaf89.WP_056350236
Methylobacterium.WP_056221677
Methylobacterium_sp_CG09_land_8_20_14_0_10_71_15.PIU04146
Bacteria.WP_043380068
Methylobacterium_sp_B1.WP_043345802
Methylobacterium_phyllostachyos.WP_091715902
Methylobacterium_sp_GXS13.WP_058193796
Methylobacterium_pseudosasicola.WP_092039348
Methylobacterium_sp_ARG_1.WP_050734531
Methylobacterium_brachiatum.WP_091864306
Methylobacterium_sp_GXF4.WP_007562025
B
Bacteria
Proteobacteria
Deltaproteobacteria
Alphaproteobacteria
Methylobacterium
Methylobacterium
Gammaproteobacteria
Gammaproteobacteria
Legionellales
Coxiella
Legionella
Pseudomonas
Pseudomonas
Pseudomonas
synthetic_construct
Candidatus_Cloacimonetes_bacterium
Criblamydia_sequanensis
Deltaproteobacteria_bacterium_RBG_13_65_10
Haliangium_ochraceum
Alphaproteobacteria_bacterium_41-28
Caulobacter_vibrioides
Methylobacterium_phyllostachyos
Methylobacterium_brachiatum
Methylobacterium_pseudosasicola
Methylobacterium_sp_88A
Methylobacterium_sp_GXS13
Methylobacterium_sp_Leaf85
Methylobacterium_sp_Leaf89
Methylobacterium_sp_Leaf111
Methylobacterium_sp_B1
Methylobacterium_sp_ARG-1
Methylobacterium_sp_GXF4
Methylobacterium_sp_CG08_land_8_20_14_0_20_71_15
Zooshikella_ganghwensis
Immundisolibacter_cernigliae
Colwellia_sp_39_35_sub15_T18
Gammaproteobacteria_bacterium_39-13
Gammaproteobacteria_bacterium_HGW-Gammaproteobacteria-6
Coxiella_sp__in__Bacteria_
Coxiella_burnetii_RSA_493
Legionella_jordanis
Legionella_brunensis
Pseudomonas_abyssi
Pseudomonas_bauzanensis
Pseudomonas_sabulinigri
Pseudomonas_aeruginosa
Pseudomonas_resinovorans
Pseudomonas_sp_TCU-HL1
Pseudomonas_sp_TTU2014-080ASC

CBU_0792

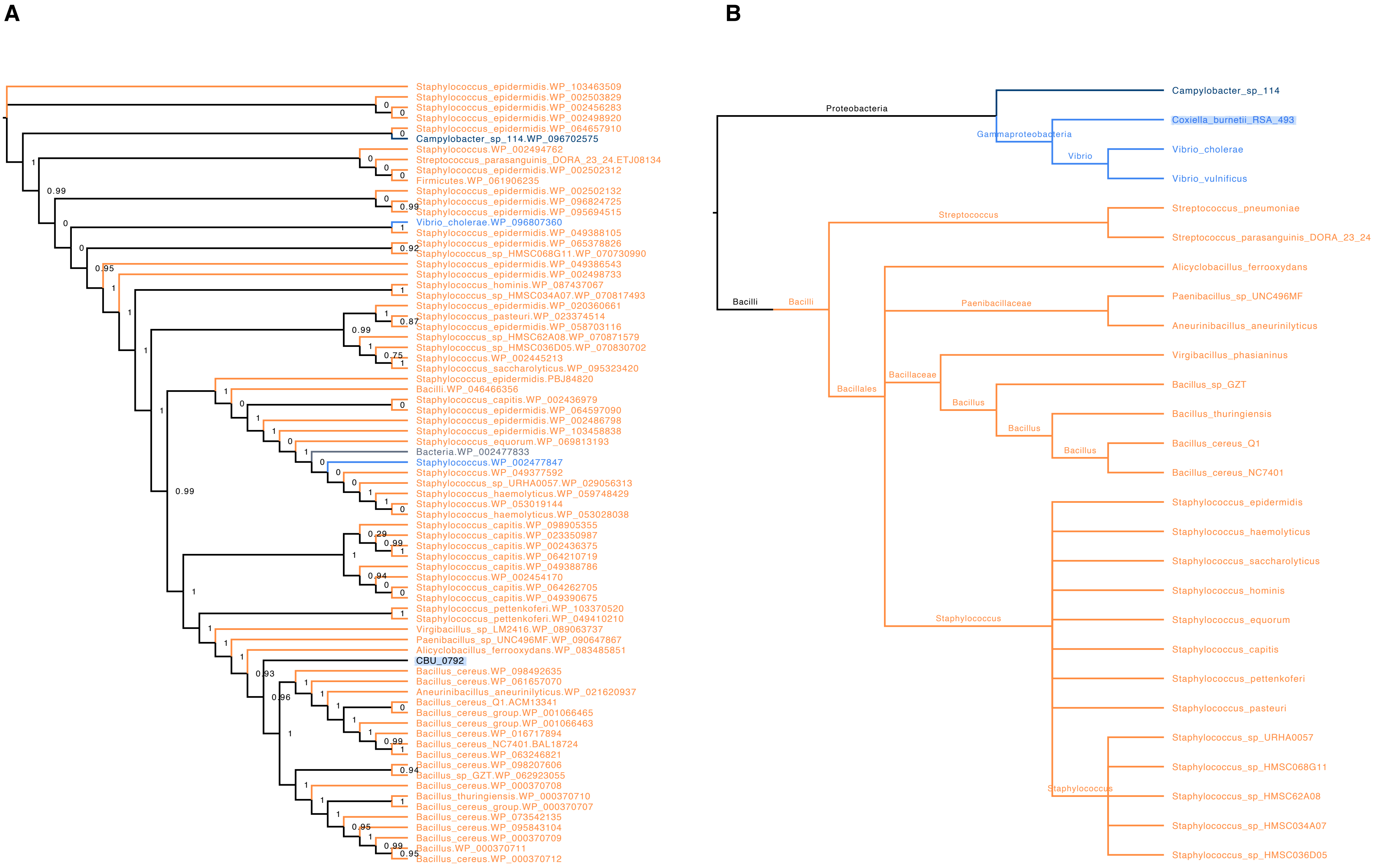
A
Staphylococcus_epidermidis.WP_103463509
Staphylococcus_epidermidis.WP_002503829
Staphylococcus_epidermidis.WP_002456283
Staphylococcus_epidermidis.WP_002498920
Staphylococcus_epidermidis.WP_064657910
Campylobacter_sp_114.WP_096702575
Staphylococcus.WP_002494762
Streptococcus_parasanguinis_DORA_23_24.ETJ08134
Staphylococcus_epidermidis.WP_002502312
Firmicutes.WP_061906235
Staphylococcus_epidermidis.WP_002502132
Staphylococcus_epidermidis.WP_096824725
Staphylococcus_epidermidis.WP_095694515
Vibrio_cholerae.WP_096807360
Staphylococcus_epidermidis.WP_049388105
Staphylococcus_epidermidis.WP_065378826
Staphylococcus_sp_HMSC068G11.WP_070730990
Staphylococcus_epidermidis.WP_049386543
Staphylococcus_epidermidis.WP_002498733
Staphylococcus_hominis.WP_087437067
Staphylococcus_sp_HMSC034A07.WP_070817493
Staphylococcus_epidermidis.WP_020360661
Staphylococcus_pasteuri.WP_023374514
Staphylococcus_epidermidis.WP_058703116
Staphylococcus_sp_HMSC62A08.WP_070871579
Staphylococcus_sp_HMSC036D05.WP_070830702
Staphylococcus.WP_002445213
Staphylococcus_saccharolyticus.WP_095323420
Staphylococcus_epidermidis.PBJ84820
Bacilli.WP_046466356
Staphylococcus_capitis.WP_002436979
Staphylococcus_epidermidis.WP_064597090
Staphylococcus_epidermidis.WP_002486798
Staphylococcus_epidermidis.WP_103458838
Staphylococcus_equorum.WP_069813193
Bacteria.WP_002477833
Staphylococcus.WP_002477847
Staphylococcus.WP_049377592
Staphylococcus_sp_URHA0057.WP_029056313
Staphylococcus_haemolyticus.WP_059748429
Staphylococcus.WP_053019144
Staphylococcus_haemolyticus.WP_053028038
Staphylococcus_capitis.WP_098905355
Staphylococcus_capitis.WP_023350987
Staphylococcus_capitis.WP_002436375
Staphylococcus_capitis.WP_064210719
Staphylococcus_capitis.WP_049388786
Staphylococcus.WP_002454170
Staphylococcus_capitis.WP_064262705
Staphylococcus_capitis.WP_049390675
Staphylococcus_pettenkoferi.WP_103370520
Staphylococcus_pettenkoferi.WP_049410210
Virgibacillus_sp_LM2416.WP_089063737
Paenibacillus_sp_UNC496MF.WP_090647867
Alicyclobacillus_ferrooxydans.WP_083485851
CBU_0792
Bacillus_cereus.WP_098492635
Bacillus_cereus.WP_061657070
Aneurinibacillus_aneurinilyticus.WP_021620937
Bacillus_cereus_Q1.ACM13341
Bacillus_cereus_group.WP_001066465
Bacillus_cereus_group.WP_001066463
Bacillus_cereus.WP_016717894
Bacillus_cereus_NC7401.BAL18724
Bacillus_cereus.WP_063246821
Bacillus_cereus.WP_098207606
Bacillus_sp_GZT.WP_062923055
Bacillus_cereus.WP_000370708
Bacillus_thuringiensis.WP_000370710
Bacillus_cereus_group.WP_000370707
Bacillus_cereus.WP_073542135
Bacillus_cereus.WP_095843104
Bacillus_cereus.WP_000370709
Bacillus.WP_000370711
Bacillus_cereus.WP_000370712
B
Proteobacteria
Campylobacter_sp_114
Gammaproteobacteria
Coxiella_burnetii_RSA_493
Vibrio
Vibrio_cholerae
Vibrio_vulnificus
Streptococcus
Streptococcus_pneumoniae
Streptococcus_parasanguinis_DORA_23_24
Bacilli
Alicyclobacillus_ferrooxydans
Paenibacillaceae
Paenibacillus_sp_UNC496MF
Aneurinibacillus_aneurinilyticus
Bacillaceae
Virgibacillus_phasianinus
Bacillales
Bacillus
Bacillus_sp_GZT
Bacillus_thuringiensis
Bacillus_cereus_Q1
Bacillus_cereus_NC7401
Staphylococcus
Staphylococcus_epidermidis
Staphylococcus_haemolyticus
Staphylococcus_saccharolyticus
Staphylococcus_hominis
Staphylococcus_equorum
Staphylococcus_capitis
Staphylococcus_pettenkoferi
Staphylococcus_pasteuri
Staphylococcus_sp_URHA0057
Staphylococcus_sp_HMSC068G11
Staphylococcus_sp_HMSC62A08
Staphylococcus_sp_HMSC034A07
Staphylococcus_sp_HMSC036D05

CBU_0788

A **B**

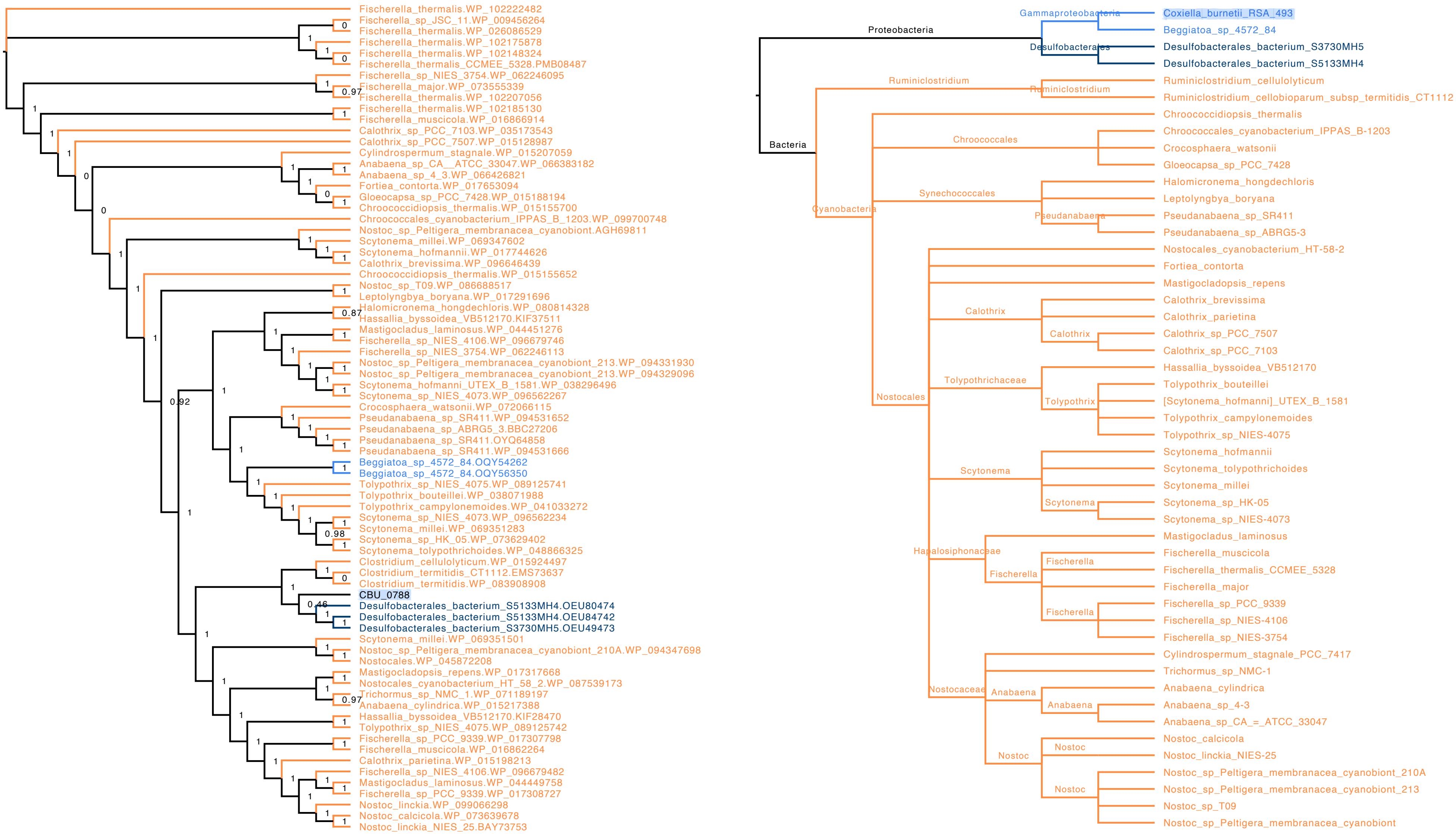
Fischerella_thermalis.WP_102222482
Fischerella_sp_JSC_11.WP_009456264
Fischerella_thermalis.WP_026086529
Fischerella_thermalis.WP_102175878
Fischerella_thermalis.WP_102148324
Fischerella_thermalis_CCMEE_5328.PMB08487
Fischerella_sp_NIES_3754.WP_062246095
Fischerella_major.WP_073555339
Fischerella_thermalis.WP_102207056
Fischerella_thermalis.WP_102185130
Fischerella_muscicola.WP_016866914
Calothrix_sp_PCC_7103.WP_035173543
Calothrix_sp_PCC_7507.WP_015128987
Cylindrospermum_stagnale.WP_015207059
Anabaena_sp_CA__ATCC_33047.WP_066383182
Anabaena_sp_4_3.WP_066426821
Fortiea_contorta.WP_017653094
Gloeocapsa_sp_PCC_7428.WP_015188194
Chroococcidiopsis_thermalis.WP_015155700
Chroococcales_cyanobacterium_IPPAS_B_1203.WP_099700748
Nostoc_sp_Peltigera_membranacea_cyanobiont.AGH69811
Scytonema_millei.WP_069347602
Scytonema_hofmannii.WP_017744626
Calothrix_brevissima.WP_096646439
Chroococcidiopsis_thermalis.WP_015155652
Nostoc_sp_T09.WP_086688517
Leptolyngbya_boryana.WP_017291696
Halomicronema_hongdechloris.WP_080814328
Hassallia_byssoidea_VB512170.KIF37511
Mastigocladus_laminosus.WP_044451276
Fischerella_sp_NIES_4106.WP_096679746
Fischerella_sp_NIES_3754.WP_062246113
Nostoc_sp_Peltigera_membranacea_cyanobiont_213.WP_094331930
Nostoc_sp_Peltigera_membranacea_cyanobiont_213.WP_094329096
Scytonema_hofmanni_UTEX_B_1581.WP_038296496
Scytonema_sp_NIES_4073.WP_096562267
Crocosphaera_watsonii.WP_072066115
Pseudanabaena_sp_SR411.WP_094531652
Pseudanabaena_sp_ABRG5_3.BBC27206
Pseudanabaena_sp_SR411.OYQ64858
Pseudanabaena_sp_SR411.WP_094531666
Beggiatoa_sp_4572_84.OQY54262
Beggiatoa_sp_4572_84.OQY56350
Tolypothrix_sp_NIES_4075.WP_089125741
Tolypothrix_bouteillei.WP_038071988
Tolypothrix_campylonemoides.WP_041033272
Scytonema_sp_NIES_4073.WP_096562234
Scytonema_millei.WP_069351283
Scytonema_sp_HK_05.WP_073629402
Scytonema_tolypothrichoides.WP_048866325
Clostridium_cellulolyticum.WP_015924497
Clostridium_termitidis_CT1112.EMS73637
Clostridium_termitidis.WP_083908908
CBU_0788
Desulfobacterales_bacterium_S5133MH4.OEU80474
Desulfobacterales_bacterium_S5133MH4.OEU84742
Desulfobacterales_bacterium_S3730MH5.OEU49473
Scytonema_millei.WP_069351501
Nostoc_sp_Peltigera_membranacea_cyanobiont_210A.WP_094347698
Nostocales.WP_045872208
Mastigocladopsis_repens.WP_017317668
Nostocales_cyanobacterium_HT_58_2.WP_087539173
Trichormus_sp_NMC_1.WP_071189197
Anabaena_cylindrica.WP_015217388
Hassallia_byssoidea_VB512170.KIF28470
Tolypothrix_sp_NIES_4075.WP_089125742
Fischerella_sp_PCC_9339.WP_017307798
Fischerella_muscicola.WP_016862264
Calothrix_parietina.WP_015198213
Fischerella_sp_NIES_4106.WP_096679482
Mastigocladus_laminosus.WP_044449758
Fischerella_sp_PCC_9339.WP_017308727
Nostoc_linckia.WP_099066298
Nostoc_calcicola.WP_073639678
Nostoc_linckia_NIES_25.BAY73753
Proteobacteria
Gammaproteobacteria
Desulfobacterales
Bacteria
Ruminiclostridium
Cyanobacteria
Chroococcales
Synechococcales
Pseudanabaena
Nostocales
Calothrix
Tolypothrichaceae
Tolypothrix
Scytonema
Hapalosiphonaceae
Fischerella
Nostocaceae
Anabaena
Nostoc
Coxiella_burnetii_RSA_493
Beggiatoa_sp_4572_84
Desulfobacterales_bacterium_S3730MH5
Desulfobacterales_bacterium_S5133MH4
Ruminiclostridium_cellulolyticum
Ruminiclostridium_cellobioparum_subsp_termitidis_CT1112
Chroococcidiopsis_thermalis
Chroococcales_cyanobacterium_IPPAS_B-1203
Crocosphaera_watsonii
Gloeocapsa_sp_PCC_7428
Halomicronema_hongdechloris
Leptolyngbya_boryana
Pseudanabaena_sp_SR411
Pseudanabaena_sp_ABRG5-3
Nostocales_cyanobacterium_HT-58-2
Fortiea_contorta
Mastigocladopsis_repens
Calothrix_brevissima
Calothrix_parietina
Calothrix_sp_PCC_7507
Calothrix_sp_PCC_7103
Hassallia_byssoidea_VB512170
Tolypothrix_bouteillei
[Scytonema_hofmanni]_UTEX_B_1581
Tolypothrix_campylonemoides
Tolypothrix_sp_NIES-4075
Scytonema_hofmannii
Scytonema_tolypothrichoides
Scytonema_millei
Scytonema_sp_HK-05
Scytonema_sp_NIES-4073
Mastigocladus_laminosus
Fischerella_muscicola
Fischerella_thermalis_CCMEE_5328
Fischerella_major
Fischerella_sp_PCC_9339
Fischerella_sp_NIES-4106
Fischerella_sp_NIES-3754
Cylindrospermum_stagnale_PCC_7417
Trichormus_sp_NMC-1
Anabaena_cylindrica
Anabaena_sp_4-3
Anabaena_sp_CA_=_ATCC_33047
Nostoc_calcicola
Nostoc_linckia_NIES-25
Nostoc_sp_Peltigera_membranacea_cyanobiont_210A
Nostoc_sp_Peltigera_membranacea_cyanobiont_213
Nostoc_sp_T09
Nostoc_sp_Peltigera_membranacea_cyanobiont

CBU_0786

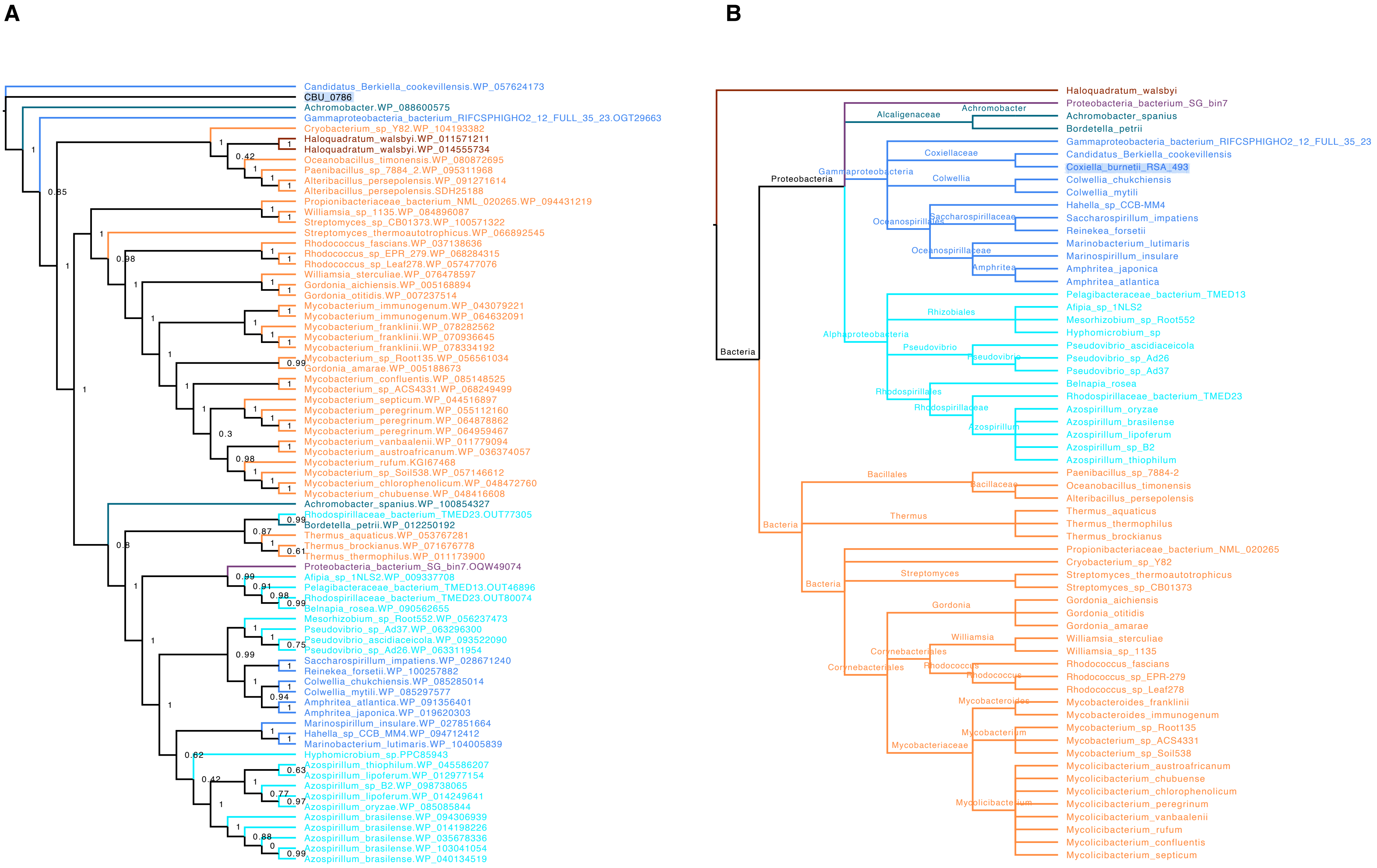

A
Candidatus_Berkiella_cookevillensis.WP_057624173
CBU_0786
Achromobacter.WP_088600575
Gammaproteobacteria_bacterium_RIFCSPHIGHO2_12_FULL_35_23.OGT29663
Cryobacterium_sp_Y82.WP_104193382
Haloquadratum_walsbyi.WP_011571211
Haloquadratum_walsbyi.WP_014555734
Oceanobacillus_timonensis.WP_080872695
Paenibacillus_sp_7884_2.WP_095311968
Alteribacillus_persepolensis.WP_091271614
Alteribacillus_persepolensis.SDH25188
Propionibacteriaceae_bacterium_NML_020265.WP_094431219
Williamsia_sp_1135.WP_084896087
Streptomyces_sp_CB01373.WP_100571322
Streptomyces_thermoautotrophicus.WP_066892545
Rhodococcus_fascians.WP_037138636
Rhodococcus_sp_EPR_279.WP_068284315
Rhodococcus_sp_Leaf278.WP_057477076
Williamsia_sterculiae.WP_076478597
Gordonia_aichiensis.WP_005168894
Gordonia_otitidis.WP_007237514
Mycobacterium_immunogenum.WP_043079221
Mycobacterium_immunogenum.WP_064632091
Mycobacterium_franklinii.WP_078282562
Mycobacterium_franklinii.WP_070936645
Mycobacterium_franklinii.WP_078334192
Mycobacterium_sp_Root135.WP_056561034
Gordonia_amarae.WP_005188673
Mycobacterium_confluentis.WP_085148525
Mycobacterium_sp_ACS4331.WP_068249499
Mycobacterium_septicum.WP_044516897
Mycobacterium_peregrinum.WP_055112160
Mycobacterium_peregrinum.WP_064878862
Mycobacterium_peregrinum.WP_064959467
Mycobacterium_vanbaalenii.WP_011779094
Mycobacterium_austroafricanum.WP_036374057
Mycobacterium_rufum.KGI67468
Mycobacterium_sp_Soil538.WP_057146612
Mycobacterium_chlorophenolicum.WP_048472760
Mycobacterium_chubuense.WP_048416608
Achromobacter_spanius.WP_100854327
Rhodospirillaceae_bacterium_TMED23.OUT77305
Bordetella_petrii.WP_012250192
Thermus_aquaticus.WP_053767281
Thermus_brockianus.WP_071676778
Thermus_thermophilus.WP_011173900
Proteobacteria_bacterium_SG_bin7.OQW49074
Afipia_sp_1NLS2.WP_009337708
Pelagibacteraceae_bacterium_TMED13.OUT46896
Rhodospirillaceae_bacterium_TMED23.OUT80074
Belnapia_rosea.WP_090562655
Mesorhizobium_sp_Root552.WP_056237473
Pseudovibrio_sp_Ad37.WP_063296300
Pseudovibrio_ascidiaceicola.WP_093522090
Pseudovibrio_sp_Ad26.WP_063311954
Saccharospirillum_impatiens.WP_028671240
Reinekea_forsetii.WP_100257882
Colwellia_chukchiensis.WP_085285014
Colwellia_mytili.WP_085297577
Amphritea_atlantica.WP_091356401
Amphritea_japonica.WP_019620303
Marinospirillum_insulare.WP_027851664
Hahella_sp_CCB_MM4.WP_094712412
Marinobacterium_lutimaris.WP_104005839
Hyphomicrobium_sp.PPC85943
Azospirillum_thiophilum.WP_045586207
Azospirillum_lipoferum.WP_012977154
Azospirillum_sp_B2.WP_098738065
Azospirillum_lipoferum.WP_014249641
Azospirillum_oryzae.WP_085085844
Azospirillum_brasilense.WP_094306939
Azospirillum_brasilense.WP_014198226
Azospirillum_brasilense.WP_035678336
Azospirillum_brasilense.WP_103041054
Azospirillum_brasilense.WP_040134519
B
Haloquadratum_walsbyi
Proteobacteria_bacterium_SG_bin7
Achromobacter_spanius
Bordetella_petrii
Gammaproteobacteria_bacterium_RIFCSPHIGHO2_12_FULL_35_23
Candidatus_Berkiella_cookevillensis
Coxiella_burnetii_RSA_493
Colwellia_chukchiensis
Colwellia_mytili
Hahella_sp_CCB-MM4
Saccharospirillum_impatiens
Reinekea_forsetii
Marinobacterium_lutimaris
Marinospirillum_insulare
Amphritea_japonica
Amphritea_atlantica
Pelagibacteraceae_bacterium_TMED13
Afipia_sp_1NLS2
Mesorhizobium_sp_Root552
Hyphomicrobium_sp
Pseudovibrio_ascidiaceicola
Pseudovibrio_sp_Ad26
Pseudovibrio_sp_Ad37
Belnapia_rosea
Rhodospirillaceae_bacterium_TMED23
Azospirillum_oryzae
Azospirillum_brasilense
Azospirillum_lipoferum
Azospirillum_sp_B2
Azospirillum_thiophilum
Paenibacillus_sp_7884-2
Oceanobacillus_timonensis
Alteribacillus_persepolensis
Thermus_aquaticus
Thermus_thermophilus
Thermus_brockianus
Propionibacteriaceae_bacterium_NML_020265
Cryobacterium_sp_Y82
Streptomyces_thermoautotrophicus
Streptomyces_sp_CB01373
Gordonia_aichiensis
Gordonia_otitidis
Gordonia_amarae
Williamsia_sterculiae
Williamsia_sp_1135
Rhodococcus_fascians
Rhodococcus_sp_EPR-279
Rhodococcus_sp_Leaf278
Mycobacteroides_franklinii
Mycobacteroides_immunogenum
Mycobacterium_sp_Root135
Mycobacterium_sp_ACS4331
Mycobacterium_sp_Soil538
Mycolicibacterium_austroafricanum
Mycolicibacterium_chubuense
Mycolicibacterium_chlorophenolicum
Mycolicibacterium_peregrinum
Mycolicibacterium_vanbaalenii
Mycolicibacterium_rufum
Mycolicibacterium_confluentis
Mycolicibacterium_septicum
Bacteria
Proteobacteria
Alcaligenaceae
Achromobacter
Gammaproteobacteria
Coxiellaceae
Colwellia
Oceanospirillales
Saccharospirillaceae
Oceanospirillaceae
Amphritea
Alphaproteobacteria
Rhizobiales
Pseudovibrio
Rhodospirillales
Rhodospirillaceae
Azospirillum
Bacillales
Bacillaceae
Thermus
Streptomyces
Gordonia
Williamsia
Corynebacteriales
Rhodococcus
Mycobacteroides
Mycobacterium
Mycobacteriaceae
Mycolicibacterium

CBU_0769

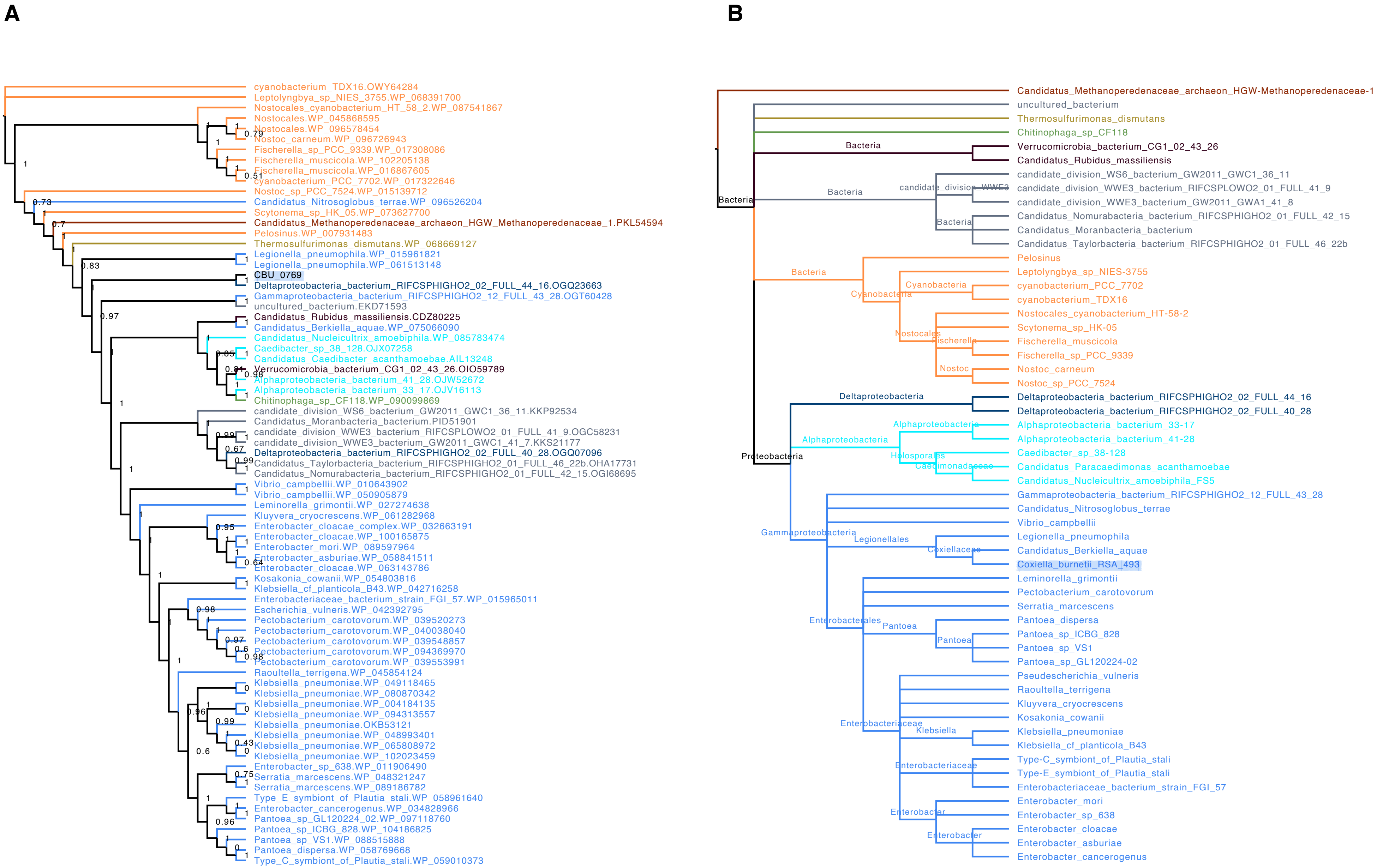

A
cyanobacterium_TDX16.OWY64284
Leptolyngbya_sp_NIES_3755.WP_068391700
Nostocales_cyanobacterium_HT_58_2.WP_087541867
Nostocales.WP_045868595
Nostocales.WP_096578454
Nostoc_carneum.WP_096726943
Fischerella_sp_PCC_9339.WP_017308086
Fischerella_muscicola.WP_102205138
Fischerella_muscicola.WP_016867605
cyanobacterium_PCC_7702.WP_017322646
Nostoc_sp_PCC_7524.WP_015139712
Candidatus_Nitrosoglobus_terrae.WP_096526204
Scytonema_sp_HK_05.WP_073627700
Candidatus_Methanoperedenaceae_archaeon_HGW_Methanoperedenaceae_1.PKL54594
Pelosinus.WP_007931483
Thermosulfurimonas_dismutans.WP_068669127
Legionella_pneumophila.WP_015961821
Legionella_pneumophila.WP_061513148
CBU_0769
Deltaproteobacteria_bacterium_RIFCSPHIGHO2_02_FULL_44_16.OGQ23663
Gammaproteobacteria_bacterium_RIFCSPHIGHO2_12_FULL_43_28.OGT60428
uncultured_bacterium.EKD71593
Candidatus_Rubidus_massiliensis.CDZ80225
Candidatus_Berkiella_aquae.WP_075066090
Candidatus_Nucleicultrix_amoebiphila.WP_085783474
Caedibacter_sp_38_128.OJX07258
Candidatus_Caedibacter_acanthamoebae.AIL13248
Verrucomicrobia_bacterium_CG1_02_43_26.OIO59789
Alphaproteobacteria_bacterium_41_28.OJW52672
Alphaproteobacteria_bacterium_33_17.OJV16113
Chitinophaga_sp_CF118.WP_090099869
candidate_division_WS6_bacterium_GW2011_GWC1_36_11.KKP92534
Candidatus_Moranbacteria_bacterium.PID51901
candidate_division_WWE3_bacterium_RIFCSPLOWO2_01_FULL_41_9.OGC58231
candidate_division_WWE3_bacterium_GW2011_GWC1_41_7.KKS21177
Deltaproteobacteria_bacterium_RIFCSPHIGHO2_02_FULL_40_28.OGQ07096
Candidatus_Taylorbacteria_bacterium_RIFCSPHIGHO2_01_FULL_46_22b.OHA17731
Candidatus_Nomurabacteria_bacterium_RIFCSPHIGHO2_01_FULL_42_15.OGI68695
Vibrio_campbellii.WP_010643902
Vibrio_campbellii.WP_050905879
Leminorella_grimontii.WP_027274638
Kluyvera_cryocrescens.WP_061282968
Enterobacter_cloacae_complex.WP_032663191
Enterobacter_cloacae.WP_100165875
Enterobacter_mori.WP_089597964
Enterobacter_asburiae.WP_058841511
Enterobacter_cloacae.WP_063143786
Kosakonia_cowanii.WP_054803816
Klebsiella_cf_planticola_B43.WP_042716258
Enterobacteriaceae_bacterium_strain_FGI_57.WP_015965011
Escherichia_vulneris.WP_042392795
Pectobacterium_carotovorum.WP_039520273
Pectobacterium_carotovorum.WP_040038040
Pectobacterium_carotovorum.WP_039548857
Pectobacterium_carotovorum.WP_094369970
Pectobacterium_carotovorum.WP_039553991
Raoultella_terrigena.WP_045854124
Klebsiella_pneumoniae.WP_049118465
Klebsiella_pneumoniae.WP_080870342
Klebsiella_pneumoniae.WP_004184135
Klebsiella_pneumoniae.WP_094313557
Klebsiella_pneumoniae.OKB53121
Klebsiella_pneumoniae.WP_048993401
Klebsiella_pneumoniae.WP_065808972
Klebsiella_pneumoniae.WP_102023459
Enterobacter_sp_638.WP_011906490
Serratia_marcescens.WP_048321247
Serratia_marcescens.WP_089186782
Type_E_symbiont_of_Plautia_stali.WP_058961640
Enterobacter_cancerogenus.WP_034828966
Pantoea_sp_GL120224_02.WP_097118760
Pantoea_sp_ICBG_828.WP_104186825
Pantoea_sp_VS1.WP_088515888
Pantoea_dispersa.WP_058769668
Type_C_symbiont_of_Plautia_stali.WP_059010373
B
Candidatus_Methanoperedenaceae_archaeon_HGW-Methanoperedenaceae-1
uncultured_bacterium
Thermosulfurimonas_dismutans
Chitinophaga_sp_CF118
Bacteria
Verrucomicrobia_bacterium_CG1_02_43_26
Candidatus_Rubidus_massiliensis
candidate_division_WS6_bacterium_GW2011_GWC1_36_11
candidate_division_WWE3
candidate_division_WWE3_bacterium_RIFCSPLOWO2_01_FULL_41_9
candidate_division_WWE3_bacterium_GW2011_GWA1_41_8
Candidatus_Nomurabacteria_bacterium_RIFCSPHIGHO2_01_FULL_42_15
Candidatus_Moranbacteria_bacterium
Candidatus_Taylorbacteria_bacterium_RIFCSPHIGHO2_01_FULL_46_22b
Pelosinus
Leptolyngbya_sp_NIES-3755
Cyanobacteria
cyanobacterium_PCC_7702
cyanobacterium_TDX16
Nostocales_cyanobacterium_HT-58-2
Nostocales
Scytonema_sp_HK-05
Fischerella
Fischerella_muscicola
Fischerella_sp_PCC_9339
Nostoc
Nostoc_carneum
Nostoc_sp_PCC_7524
Deltaproteobacteria
Deltaproteobacteria_bacterium_RIFCSPHIGHO2_02_FULL_44_16
Deltaproteobacteria_bacterium_RIFCSPHIGHO2_02_FULL_40_28
Alphaproteobacteria
Alphaproteobacteria_bacterium_33-17
Alphaproteobacteria_bacterium_41-28
Proteobacteria
Holosporales
Caedibacter_sp_38-128
Caedimonadaceae
Candidatus_Paracaedimonas_acanthamoebae
Candidatus_Nucleicultrix_amoebiphila_FS5
Gammaproteobacteria_bacterium_RIFCSPHIGHO2_12_FULL_43_28
Candidatus_Nitrosoglobus_terrae
Vibrio_campbellii
Gammaproteobacteria
Legionella_pneumophila
Legionellales
Coxiellaceae
Candidatus_Berkiella_aquae
Coxiella_burnetii_RSA_493
Leminorella_grimontii
Pectobacterium_carotovorum
Serratia_marcescens
Enterobacterales
Pantoea
Pantoea_dispersa
Pantoea_sp_ICBG_828
Pantoea_sp_VS1
Pantoea_sp_GL120224-02
Pseudescherichia_vulneris
Raoultella_terrigena
Kluyvera_cryocrescens
Kosakonia_cowanii
Enterobacteriaceae
Klebsiella
Klebsiella_pneumoniae
Klebsiella_cf_planticola_B43
Type-C_symbiont_of_Plautia_stali
Type-E_symbiont_of_Plautia_stali
Enterobacteriaceae_bacterium_strain_FGI_57
Enterobacter
Enterobacter_mori
Enterobacter_sp_638
Enterobacter_cloacae
Enterobacter_asburiae
Enterobacter_cancerogenus

CBU_0767

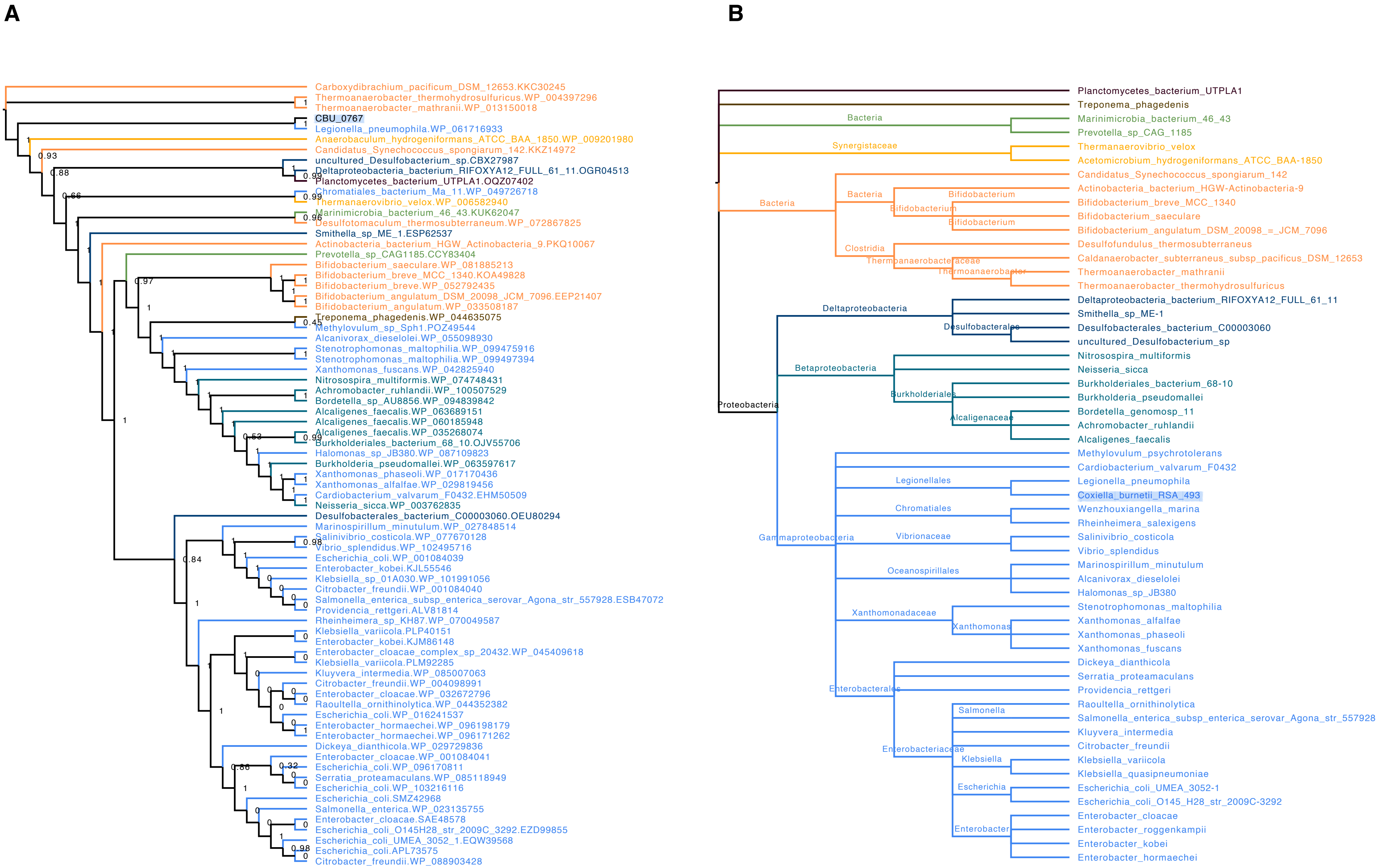
A
Carboxydibrachium_pacificum_DSM_12653.KKC30245
Thermoanaerobacter_thermohydrosulfuricus.WP_004397296
Thermoanaerobacter_mathranii.WP_013150018
CBU_0767
Legionella_pneumophila.WP_061716933
Anaerobaculum_hydrogeniformans_ATCC_BAA_1850.WP_009201980
Candidatus_Synechococcus_spongiarum_142.KKZ14972
uncultured_Desulfobacterium_sp.CBX27987
Deltaproteobacteria_bacterium_RIFOXYA12_FULL_61_11.OGR04513
Planctomycetes_bacterium_UTPLA1.OQZ07402
Chromatiales_bacterium_Ma_11.WP_049726718
Thermanaerovibrio_velox.WP_006582940
Marinimicrobia_bacterium_46_43.KUK62047
Desulfotomaculum_thermosubterraneum.WP_072867825
Smithella_sp_ME_1.ESP62537
Actinobacteria_bacterium_HGW_Actinobacteria_9.PKQ10067
Prevotella_sp_CAG1185.CCY83404
Bifidobacterium_saeculare.WP_081885213
Bifidobacterium_breve_MCC_1340.KOA49828
Bifidobacterium_breve.WP_052792435
Bifidobacterium_angulatum_DSM_20098_JCM_7096.EEP21407
Bifidobacterium_angulatum.WP_033508187
Treponema_phagedenis.WP_044635075
Methylovulum_sp_Sph1.POZ49544
Alcanivorax_dieselolei.WP_055098930
Stenotrophomonas_maltophilia.WP_099475916
Stenotrophomonas_maltophilia.WP_099497394
Xanthomonas_fuscans.WP_042825940
Nitrosospira_multiformis.WP_074748431
Achromobacter_ruhlandii.WP_100507529
Bordetella_sp_AU8856.WP_094839842
Alcaligenes_faecalis.WP_063689151
Alcaligenes_faecalis.WP_060185948
Alcaligenes_faecalis.WP_035268074
Burkholderiales_bacterium_68_10.OJV55706
Halomonas_sp_JB380.WP_087109823
Burkholderia_pseudomallei.WP_063597617
Xanthomonas_phaseoli.WP_017170436
Xanthomonas_alfalfae.WP_029819456
Cardiobacterium_valvarum_F0432.EHM50509
Neisseria_sicca.WP_003762835
Desulfobacterales_bacterium_C00003060.OEU80294
Marinospirillum_minutulum.WP_027848514
Salinivibrio_costicola.WP_077670128
Vibrio_splendidus.WP_102495716
Escherichia_coli.WP_001084039
Enterobacter_kobei.KJL55546
Klebsiella_sp_01A030.WP_101991056
Citrobacter_freundii.WP_001084040
Salmonella_enterica_subsp_enterica_serovar_Agona_str_557928.ESB47072
Providencia_rettgeri.ALV81814
Rheinheimera_sp_KH87.WP_070049587
Klebsiella_variicola.PLP40151
Enterobacter_kobei.KJM86148
Enterobacter_cloacae_complex_sp_20432.WP_045409618
Klebsiella_variicola.PLM92285
Kluyvera_intermedia.WP_085007063
Citrobacter_freundii.WP_004098991
Enterobacter_cloacae.WP_032672796
Raoultella_ornithinolytica.WP_044352382
Escherichia_coli.WP_016241537
Enterobacter_hormaechei.WP_096198179
Enterobacter_hormaechei.WP_096171262
Dickeya_dianthicola.WP_029729836
Enterobacter_cloacae.WP_001084041
Escherichia_coli.WP_096170811
Serratia_proteamaculans.WP_085118949
Escherichia_coli.WP_103216116
Escherichia_coli.SMZ42968
Salmonella_enterica.WP_023135755
Enterobacter_cloacae.SAE48578
Escherichia_coli_O145H28_str_2009C_3292.EZD99855
Escherichia_coli_UMEA_3052_1.EQW39568
Escherichia_coli.APL73575
Citrobacter_freundii.WP_088903428
B
Planctomycetes_bacterium_UTPLA1
Treponema_phagedenis
Bacteria
Marinimicrobia_bacterium_46_43
Prevotella_sp_CAG_1185
Synergistaceae
Thermanaerovibrio_velox
Acetomicrobium_hydrogeniformans_ATCC_BAA-1850
Bacteria
Candidatus_Synechococcus_spongiarum_142
Bacteria
Bifidobacterium
Actinobacteria_bacterium_HGW-Actinobacteria-9
Bifidobacterium
Bifidobacterium_breve_MCC_1340
Bifidobacterium_saeculare
Bifidobacterium
Bifidobacterium_angulatum_DSM_20098_=_JCM_7096
Clostridia
Desulfofundulus_thermosubterraneus
Thermoanaerobacteraceae
Caldanaerobacter_subterraneus_subsp_pacificus_DSM_12653
Thermoanaerobacter
Thermoanaerobacter_mathranii
Thermoanaerobacter_thermohydrosulfuricus
Deltaproteobacteria
Deltaproteobacteria_bacterium_RIFOXYA12_FULL_61_11
Smithella_sp_ME-1
Desulfobacterales
Desulfobacterales_bacterium_C00003060
uncultured_Desulfobacterium_sp
Betaproteobacteria
Nitrosospira_multiformis
Neisseria_sicca
Burkholderiales
Burkholderiales_bacterium_68-10
Burkholderia_pseudomallei
Proteobacteria
Alcaligenaceae
Bordetella_genomosp_11
Achromobacter_ruhlandii
Alcaligenes_faecalis
Methylovulum_psychrotolerans
Cardiobacterium_valvarum_F0432
Legionellales
Legionella_pneumophila
Coxiella_burnetii_RSA_493
Chromatiales
Wenzhouxiangella_marina
Rheinheimera_salexigens
Gammaproteobacteria
Vibrionaceae
Salinivibrio_costicola
Vibrio_splendidus
Oceanospirillales
Marinospirillum_minutulum
Alcanivorax_dieselolei
Halomonas_sp_JB380
Xanthomonadaceae
Stenotrophomonas_maltophilia
Xanthomonas
Xanthomonas_alfalfae
Xanthomonas_phaseoli
Xanthomonas_fuscans
Dickeya_dianthicola
Serratia_proteamaculans
Enterobacterales
Providencia_rettgeri
Raoultella_ornithinolytica
Salmonella
Salmonella_enterica_subsp_enterica_serovar_Agona_str_557928
Kluyvera_intermedia
Enterobacteriaceae
Citrobacter_freundii
Klebsiella
Klebsiella_variicola
Klebsiella_quasipneumoniae
Escherichia
Escherichia_coli_UMEA_3052-1
Escherichia_coli_O145_H28_str_2009C-3292
Enterobacter
Enterobacter_cloacae
Enterobacter_roggenkampii
Enterobacter_kobei
Enterobacter_hormaechei

CBU_0715, 0724, 1086, 1104, 1544

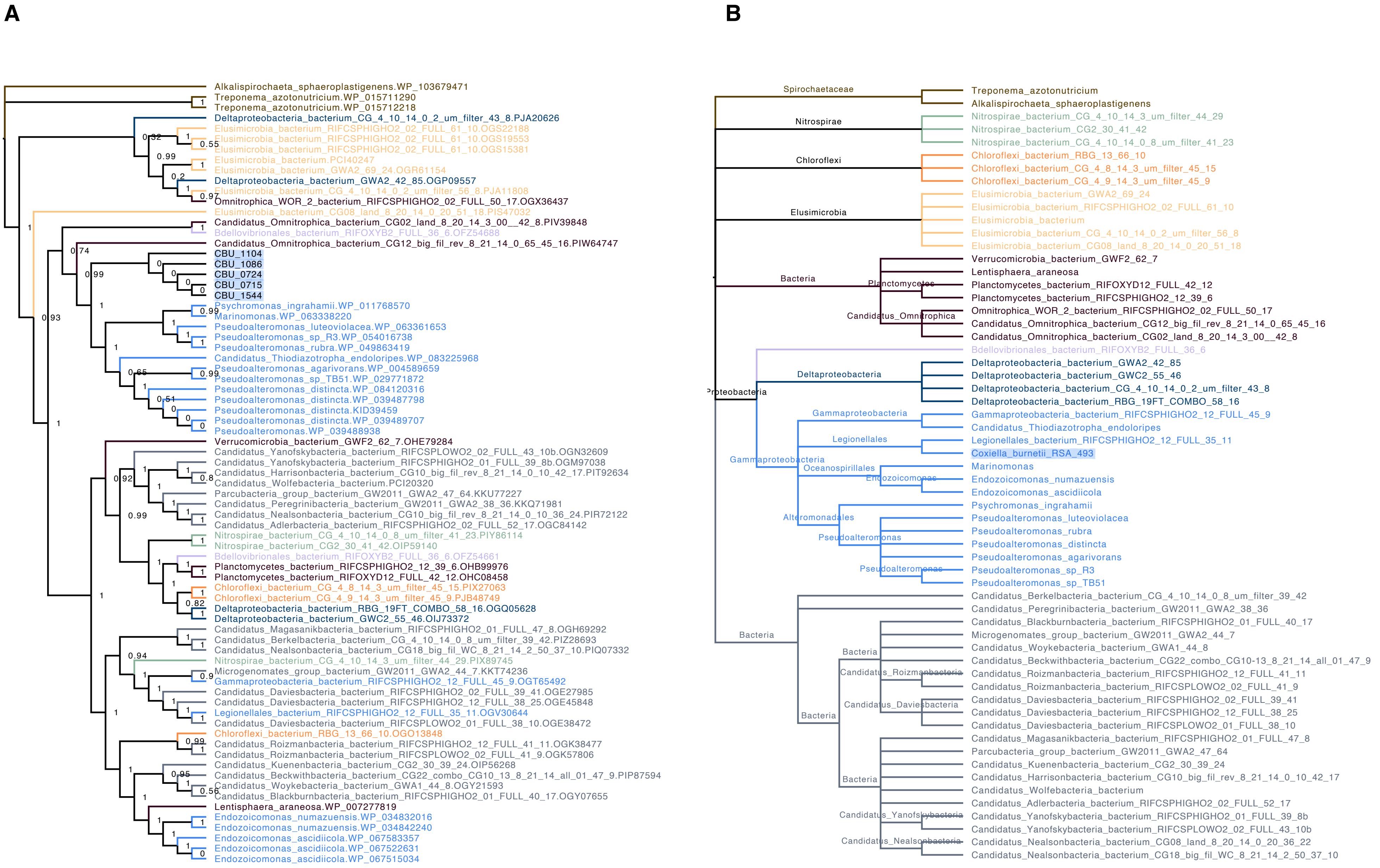

CBU_0514

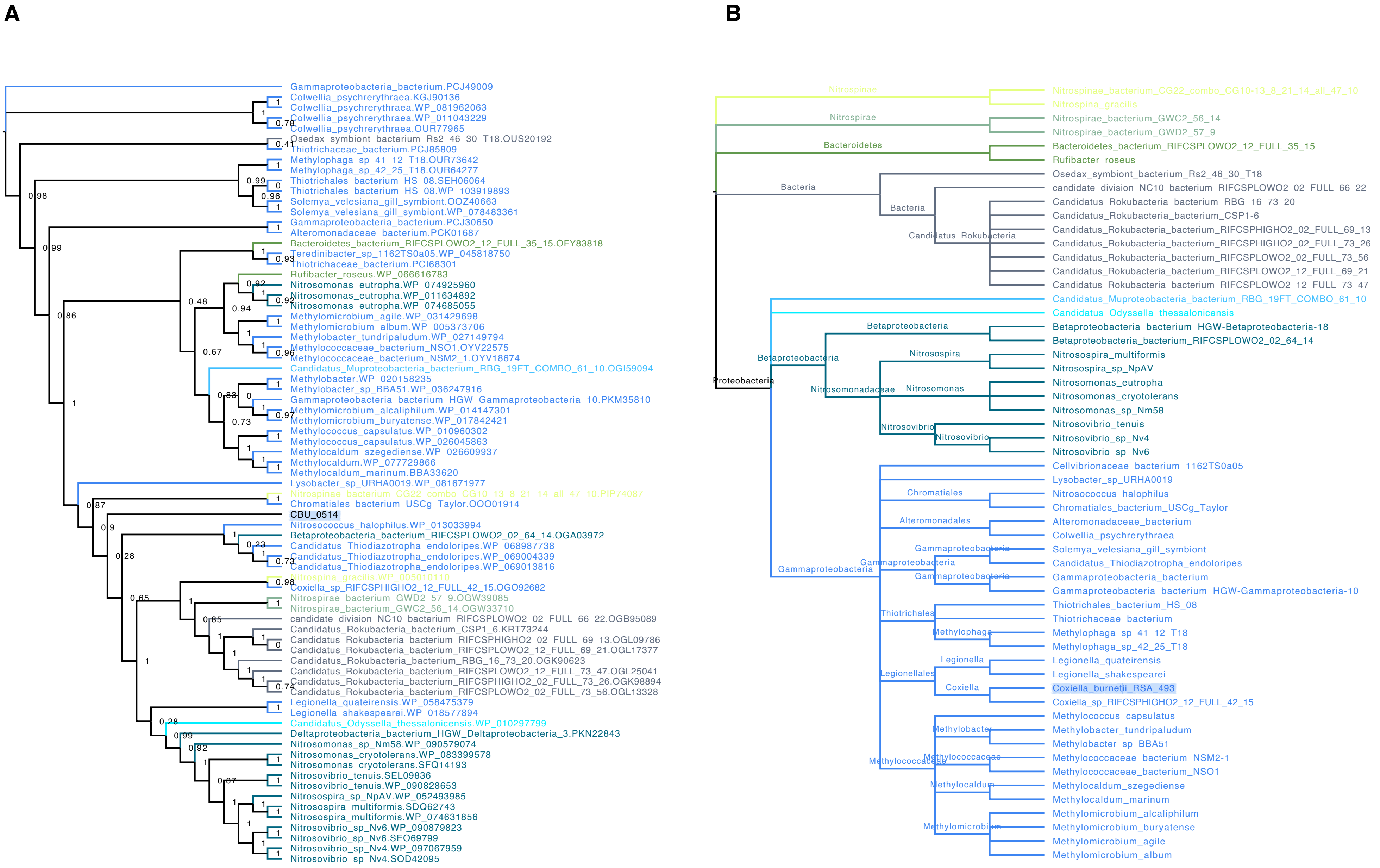
A
Gammaproteobacteria_bacterium.PCJ49009
Colwellia_psychrerythraea.KGJ90136
Colwellia_psychrerythraea.WP_081962063
Colwellia_psychrerythraea.WP_011043229
Colwellia_psychrerythraea.OUR77965
Osedax_symbiont_bacterium_Rs2_46_30_T18.OUS20192
Thiotrichaceae_bacterium.PCJ85809
Methylophaga_sp_41_12_T18.OUR73642
Methylophaga_sp_42_25_T18.OUR64277
Thiotrichales_bacterium_HS_08.SEH06064
Thiotrichales_bacterium_HS_08.WP_103919893
Solemya_velesiana_gill_symbiont.OOZ40663
Solemya_velesiana_gill_symbiont.WP_078483361
Gammaproteobacteria_bacterium.PCJ30650
Alteromonadaceae_bacterium.PCK01687
Bacteroidetes_bacterium_RIFCSPLOWO2_12_FULL_35_15.OFY83818
Teredinibacter_sp_1162TS0a05.WP_045818750
Thiotrichaceae_bacterium.PCI68301
Rufibacter_roseus.WP_066616783
Nitrosomonas_eutropha.WP_074925960
Nitrosomonas_eutropha.WP_011634892
Nitrosomonas_eutropha.WP_074685055
Methylomicrobium_agile.WP_031429698
Methylomicrobium_album.WP_005373706
Methylobacter_tundripaludum.WP_027149794
Methylococcaceae_bacterium_NSO1.OYV22575
Methylococcaceae_bacterium_NSM2_1.OYV18674
Candidatus_Muproteobacteria_bacterium_RBG_19FT_COMBO_61_10.OGI59094
Methylobacter.WP_020158235
Methylobacter_sp_BBA51.WP_036247916
Gammaproteobacteria_bacterium_HGW_Gammaproteobacteria_10.PKM35810
Methylomicrobium_alcaliphilum.WP_014147301
Methylomicrobium_buryatense.WP_017842421
Methylococcus_capsulatus.WP_010960302
Methylococcus_capsulatus.WP_026045863
Methylocaldum_szegediense.WP_026609937
Methylocaldum.WP_077729866
Methylocaldum_marinum.BBA33620
Lysobacter_sp_URHA0019.WP_081671977
Nitrospinae_bacterium_CG22_combo_CG10_13_8_21_14_all_47_10.PIP74087
Chromatiales_bacterium_USCg_Taylor.OOO01914
CBU_0514
Nitrosococcus_halophilus.WP_013033994
Betaproteobacteria_bacterium_RIFCSPLOWO2_02_64_14.OGA03972
Candidatus_Thiodiazotropha_endoloripes.WP_068987738
Candidatus_Thiodiazotropha_endoloripes.WP_069004339
Candidatus_Thiodiazotropha_endoloripes.WP_069013816
Nitrospina_gracilis.WP_005010110
Coxiella_sp_RIFCSPHIGHO2_12_FULL_42_15.OGO92682
Nitrospirae_bacterium_GWD2_57_9.OGW39085
Nitrospirae_bacterium_GWC2_56_14.OGW33710
candidate_division_NC10_bacterium_RIFCSPLOWO2_02_FULL_66_22.OGB95089
Candidatus_Rokubacteria_bacterium_CSP1_6.KRT73244
Candidatus_Rokubacteria_bacterium_RIFCSPHIGHO2_02_FULL_69_13.OGL09786
Candidatus_Rokubacteria_bacterium_RIFCSPLOWO2_12_FULL_69_21.OGL17377
Candidatus_Rokubacteria_bacterium_RBG_16_73_20.OGK90623
Candidatus_Rokubacteria_bacterium_RIFCSPLOWO2_12_FULL_73_47.OGL25041
Candidatus_Rokubacteria_bacterium_RIFCSPHIGHO2_02_FULL_73_26.OGK98894
Candidatus_Rokubacteria_bacterium_RIFCSPLOWO2_02_FULL_73_56.OGL13328
Legionella_quateirensis.WP_058475379
Legionella_shakespearei.WP_018577894
Candidatus_Odyssella_thessalonicensis.WP_010297799
Deltaproteobacteria_bacterium_HGW_Deltaproteobacteria_3.PKN22843
Nitrosomonas_sp_Nm58.WP_090579074
Nitrosomonas_cryotolerans.WP_083399578
Nitrosomonas_cryotolerans.SFQ14193
Nitrosovibrio_tenuis.SEL09836
Nitrosovibrio_tenuis.WP_090828653
Nitrosospira_sp_NpAV.WP_052493985
Nitrosospira_multiformis.SDQ62743
Nitrosospira_multiformis.WP_074631856
Nitrosovibrio_sp_Nv6.WP_090879823
Nitrosovibrio_sp_Nv6.SEO69799
Nitrosovibrio_sp_Nv4.WP_097067959
Nitrosovibrio_sp_Nv4.SOD42095
B
Nitrospinae
Nitrospirae
Bacteroidetes
Bacteria
Candidatus_Rokubacteria
Proteobacteria
Betaproteobacteria
Nitrosospira
Nitrosomonadaceae
Nitrosomonas
Nitrosovibrio
Gammaproteobacteria
Chromatiales
Alteromonadales
Thiotrichales
Methylophaga
Legionellales
Legionella
Coxiella
Methylococcaceae
Methylobacter
Methylocaldum
Methylomicrobium
Nitrospinae_bacterium_CG22_combo_CG10-13_8_21_14_all_47_10
Nitrospina_gracilis
Nitrospirae_bacterium_GWC2_56_14
Nitrospirae_bacterium_GWD2_57_9
Bacteroidetes_bacterium_RIFCSPLOWO2_12_FULL_35_15
Rufibacter_roseus
Osedax_symbiont_bacterium_Rs2_46_30_T18
candidate_division_NC10_bacterium_RIFCSPLOWO2_02_FULL_66_22
Candidatus_Rokubacteria_bacterium_RBG_16_73_20
Candidatus_Rokubacteria_bacterium_CSP1-6
Candidatus_Rokubacteria_bacterium_RIFCSPHIGHO2_02_FULL_69_13
Candidatus_Rokubacteria_bacterium_RIFCSPHIGHO2_02_FULL_73_26
Candidatus_Rokubacteria_bacterium_RIFCSPLOWO2_02_FULL_73_56
Candidatus_Rokubacteria_bacterium_RIFCSPLOWO2_12_FULL_69_21
Candidatus_Rokubacteria_bacterium_RIFCSPLOWO2_12_FULL_73_47
Candidatus_Muproteobacteria_bacterium_RBG_19FT_COMBO_61_10
Candidatus_Odyssella_thessalonicensis
Betaproteobacteria_bacterium_HGW-Betaproteobacteria-18
Betaproteobacteria_bacterium_RIFCSPLOWO2_02_64_14
Nitrosospira_multiformis
Nitrosospira_sp_NpAV
Nitrosomonas_eutropha
Nitrosomonas_cryotolerans
Nitrosomonas_sp_Nm58
Nitrosovibrio_tenuis
Nitrosovibrio_sp_Nv4
Nitrosovibrio_sp_Nv6
Cellvibrionaceae_bacterium_1162TS0a05
Lysobacter_sp_URHA0019
Nitrosococcus_halophilus
Chromatiales_bacterium_USCg_Taylor
Alteromonadaceae_bacterium
Colwellia_psychrerythraea
Solemya_velesiana_gill_symbiont
Candidatus_Thiodiazotropha_endoloripes
Gammaproteobacteria_bacterium
Gammaproteobacteria_bacterium_HGW-Gammaproteobacteria-10
Thiotrichales_bacterium_HS_08
Thiotrichaceae_bacterium
Methylophaga_sp_41_12_T18
Methylophaga_sp_42_25_T18
Legionella_quateirensis
Legionella_shakespearei
Coxiella_burnetii_RSA_493
Coxiella_sp_RIFCSPHIGHO2_12_FULL_42_15
Methylococcus_capsulatus
Methylobacter_tundripaludum
Methylobacter_sp_BBA51
Methylococcaceae_bacterium_NSM2-1
Methylococcaceae_bacterium_NSO1
Methylocaldum_szegediense
Methylocaldum_marinum
Methylomicrobium_alcaliphilum
Methylomicrobium_buryatense
Methylomicrobium_agile
Methylomicrobium_album

CBU_0513

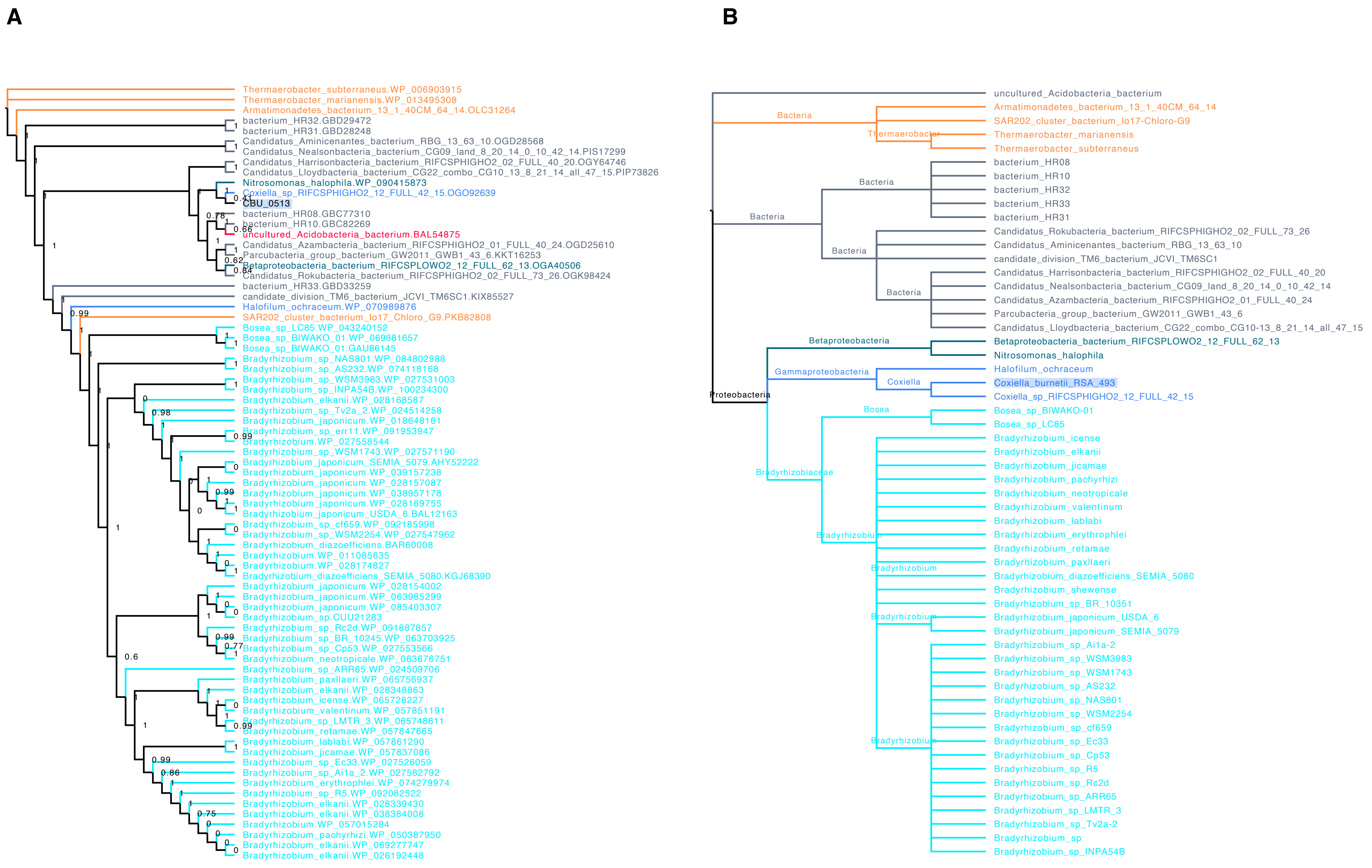

A
Thermaerobacter_subterraneus.WP_006903915
Thermaerobacter_marianensis.WP_013495308
Armatimonadetes_bacterium_13_1_40CM_64_14.OLC31264
bacterium_HR32.GBD29472
bacterium_HR31.GBD28248
Candidatus_Aminicenantes_bacterium_RBG_13_63_10.OGD28568
Candidatus_Nealsonbacteria_bacterium_CG09_land_8_20_14_0_10_42_14.PIS17299
Candidatus_Harrisonbacteria_bacterium_RIFCSPHIGHO2_02_FULL_40_20.OGY64746
Candidatus_Lloydbacteria_bacterium_CG22_combo_CG10_13_8_21_14_all_47_15.PIP73826
Nitrosomonas_halophila.WP_090415873
Coxiella_sp_RIFCSPHIGHO2_12_FULL_42_15.OGO92639
CBU_0513
bacterium_HR08.GBC77310
bacterium_HR10.GBC82269
uncultured_Acidobacteria_bacterium.BAL54875
Candidatus_Azambacteria_bacterium_RIFCSPHIGHO2_01_FULL_40_24.OGD25610
Parcubacteria_group_bacterium_GW2011_GWB1_43_6.KKT16253
Betaproteobacteria_bacterium_RIFCSPLOWO2_12_FULL_62_13.OGA40506
Candidatus_Rokubacteria_bacterium_RIFCSPHIGHO2_02_FULL_73_26.OGK98424
bacterium_HR33.GBD33259
candidate_division_TM6_bacterium_JCVI_TM6SC1.KIX85527
Halofilum_ochraceum.WP_070989876
SAR202_cluster_bacterium_Io17_Chloro_G9.PKB82808
Bosea_sp_LC85.WP_043240152
Bosea_sp_BIWAKO_01.WP_069881657
Bosea_sp_BIWAKO_01.GAU86145
Bradyrhizobium_sp_NAS801.WP_084802988
Bradyrhizobium_sp_AS232.WP_074118168
Bradyrhizobium_sp_WSM3983.WP_027531003
Bradyrhizobium_sp_INPA54B.WP_100234300
Bradyrhizobium_elkanii.WP_028168587
Bradyrhizobium_sp_Tv2a_2.WP_024514258
Bradyrhizobium_japonicum.WP_018648161
Bradyrhizobium_sp_err11.WP_091953947
Bradyrhizobium.WP_027558544
Bradyrhizobium_sp_WSM1743.WP_027571190
Bradyrhizobium_japonicum_SEMIA_5079.AHY52222
Bradyrhizobium_japonicum.WP_039157238
Bradyrhizobium_japonicum.WP_028157087
Bradyrhizobium_japonicum.WP_038957178
Bradyrhizobium_japonicum.WP_028169755
Bradyrhizobium_japonicum_USDA_6.BAL12163
Bradyrhizobium_sp_cf659.WP_092185998
Bradyrhizobium_sp_WSM2254.WP_027547962
Bradyrhizobium_diazoefficiens.BAR60008
Bradyrhizobium.WP_011085635
Bradyrhizobium.WP_028174827
Bradyrhizobium_diazoefficiens_SEMIA_5080.KGJ68390
Bradyrhizobium_japonicum.WP_028154002
Bradyrhizobium_japonicum.WP_063985299
Bradyrhizobium_japonicum.WP_085403307
Bradyrhizobium_sp.CUU21283
Bradyrhizobium_sp_Rc2d.WP_091887857
Bradyrhizobium_sp_BR_10245.WP_063703925
Bradyrhizobium_sp_Cp53.WP_027553566
Bradyrhizobium_neotropicale.WP_063676751
Bradyrhizobium_sp_ARR65.WP_024509706
Bradyrhizobium_paxllaeri.WP_065756937
Bradyrhizobium_elkanii.WP_028348863
Bradyrhizobium_icense.WP_065728227
Bradyrhizobium_valentinum.WP_057851191
Bradyrhizobium_sp_LMTR_3.WP_065748611
Bradyrhizobium_retamae.WP_057847665
Bradyrhizobium_lablabi.WP_057861290
Bradyrhizobium_jicamae.WP_057837086
Bradyrhizobium_sp_Ec33.WP_027526059
Bradyrhizobium_sp_Ai1a_2.WP_027582792
Bradyrhizobium_erythrophlei.WP_074279974
Bradyrhizobium_sp_R5.WP_092082522
Bradyrhizobium_elkanii.WP_028339430
Bradyrhizobium_elkanii.WP_038384008
Bradyrhizobium.WP_057015284
Bradyrhizobium_pachyrhizi.WP_050387950
Bradyrhizobium_elkanii.WP_069277747
Bradyrhizobium_elkanii.WP_026192448
B
uncultured_Acidobacteria_bacterium
Bacteria
Armatimonadetes_bacterium_13_1_40CM_64_14
SAR202_cluster_bacterium_Io17-Chloro-G9
Thermaerobacter
Thermaerobacter_marianensis
Thermaerobacter_subterraneus
bacterium_HR08
bacterium_HR10
bacterium_HR32
bacterium_HR33
bacterium_HR31
Candidatus_Rokubacteria_bacterium_RIFCSPHIGHO2_02_FULL_73_26
Candidatus_Aminicenantes_bacterium_RBG_13_63_10
candidate_division_TM6_bacterium_JCVI_TM6SC1
Candidatus_Harrisonbacteria_bacterium_RIFCSPHIGHO2_02_FULL_40_20
Candidatus_Nealsonbacteria_bacterium_CG09_land_8_20_14_0_10_42_14
Candidatus_Azambacteria_bacterium_RIFCSPHIGHO2_01_FULL_40_24
Parcubacteria_group_bacterium_GW2011_GWB1_43_6
Candidatus_Lloydbacteria_bacterium_CG22_combo_CG10-13_8_21_14_all_47_15
Betaproteobacteria
Betaproteobacteria_bacterium_RIFCSPLOWO2_12_FULL_62_13
Nitrosomonas_halophila
Gammaproteobacteria
Halofilum_ochraceum
Coxiella
Coxiella_burnetii_RSA_493
Coxiella_sp_RIFCSPHIGHO2_12_FULL_42_15
Proteobacteria
Bosea
Bosea_sp_BIWAKO-01
Bosea_sp_LC85
Bradyrhizobiaceae
Bradyrhizobium
Bradyrhizobium_icense
Bradyrhizobium_elkanii
Bradyrhizobium_jicamae
Bradyrhizobium_pachyrhizi
Bradyrhizobium_neotropicale
Bradyrhizobium_valentinum
Bradyrhizobium_lablabi
Bradyrhizobium_erythrophlei
Bradyrhizobium_retamae
Bradyrhizobium_paxllaeri
Bradyrhizobium_diazoefficiens_SEMIA_5080
Bradyrhizobium_shewense
Bradyrhizobium_sp_BR_10351
Bradyrhizobium_japonicum_USDA_6
Bradyrhizobium_japonicum_SEMIA_5079
Bradyrhizobium_sp_Ai1a-2
Bradyrhizobium_sp_WSM3983
Bradyrhizobium_sp_WSM1743
Bradyrhizobium_sp_AS232
Bradyrhizobium_sp_NAS801
Bradyrhizobium_sp_WSM2254
Bradyrhizobium_sp_cf659
Bradyrhizobium_sp_Ec33
Bradyrhizobium_sp_Cp53
Bradyrhizobium_sp_R5
Bradyrhizobium_sp_Rc2d
Bradyrhizobium_sp_ARR65
Bradyrhizobium_sp_LMTR_3
Bradyrhizobium_sp_Tv2a-2
Bradyrhizobium_sp
Bradyrhizobium_sp_INPA54B

CBU_0455

A

B

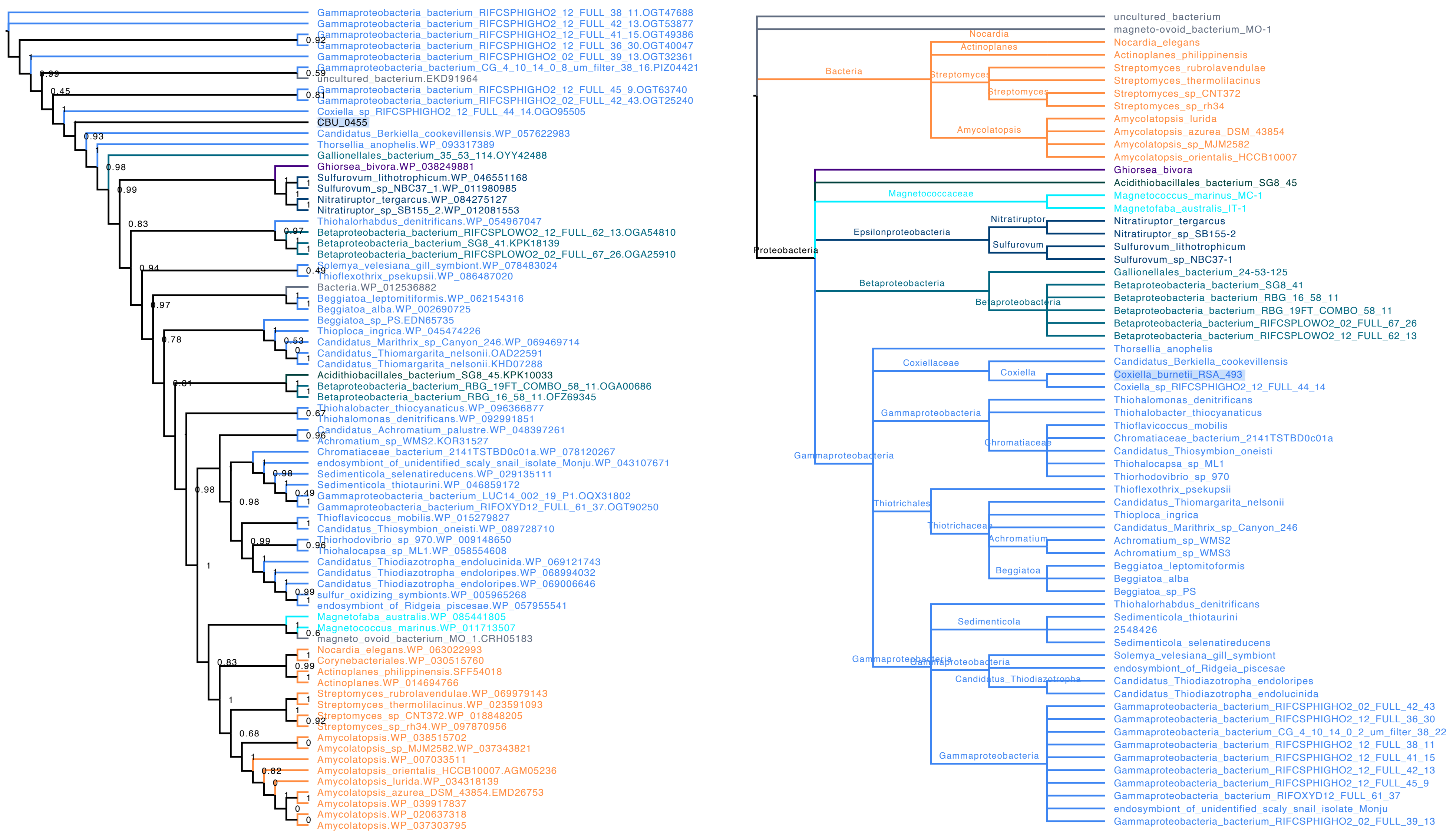

Gammaproteobacteria_bacterium_RIFCSPHIGHO2_12_FULL_38_11.OGT47688
Gammaproteobacteria_bacterium_RIFCSPHIGHO2_12_FULL_42_13.OGT53877
Gammaproteobacteria_bacterium_RIFCSPHIGHO2_12_FULL_41_15.OGT49386
Gammaproteobacteria_bacterium_RIFCSPHIGHO2_12_FULL_36_30.OGT40047
Gammaproteobacteria_bacterium_RIFCSPHIGHO2_02_FULL_39_13.OGT32361
Gammaproteobacteria_bacterium_CG_4_10_14_0_8_um_filter_38_16.PIZ04421
uncultured_bacterium.EKD91964
Gammaproteobacteria_bacterium_RIFCSPHIGHO2_12_FULL_45_9.OGT63740
Gammaproteobacteria_bacterium_RIFCSPHIGHO2_02_FULL_42_43.OGT25240
Coxiella_sp_RIFCSPHIGHO2_12_FULL_44_14.OGO95505
CBU_0455
Candidatus_Berkiella_cookevillensis.WP_057622983
Thorsellia_anophelis.WP_093317389
Gallionellales_bacterium_35_53_114.OYY42488
Ghiorsea_bivora.WP_038249881
Sulfurovum_lithotrophicum.WP_046551168
Sulfurovum_sp_NBC37_1.WP_011980985
Nitratiruptor_tergarcus.WP_084275127
Nitratiruptor_sp_SB155_2.WP_012081553
Thiohalorhabdus_denitrificans.WP_054967047
Betaproteobacteria_bacterium_RIFCSPLOWO2_12_FULL_62_13.OGA54810
Betaproteobacteria_bacterium_SG8_41.KPK18139
Betaproteobacteria_bacterium_RIFCSPLOWO2_02_FULL_67_26.OGA25910
Solemya_velesiana_gill_symbiont.WP_078483024
Thioflexothrix_psekupsii.WP_086487020
Bacteria.WP_012536882
Beggiatoa_leptomitiformis.WP_062154316
Beggiatoa_alba.WP_002690725
Beggiatoa_sp_PS.EDN65735
Thioploca_ingrica.WP_045474226
Candidatus_Marithrix_sp_Canyon_246.WP_069469714
Candidatus_Thiomargarita_nelsonii.OAD22591
Candidatus_Thiomargarita_nelsonii.KHD07288
Acidithiobacillales_bacterium_SG8_45.KPK10033
Betaproteobacteria_bacterium_RBG_19FT_COMBO_58_11.OGA00686
Betaproteobacteria_bacterium_RBG_16_58_11.OFZ69345
Thiohalobacter_thiocyanaticus.WP_096366877
Thiohalomonas_denitrificans.WP_092991851
Candidatus_Achromatium_palustre.WP_048397261
Achromatium_sp_WMS2.KOR31527
Chromatiaceae_bacterium_2141TSTBD0c01a.WP_078120267
endosymbiont_of_unidentified_scaly_snail_isolate_Monju.WP_043107671
Sedimenticola_selenatireducens.WP_029135111
Sedimenticola_thiotaurini.WP_046859172
Gammaproteobacteria_bacterium_LUC14_002_19_P1.OQX31802
Gammaproteobacteria_bacterium_RIFOXYD12_FULL_61_37.OGT90250
Thioflavicoccus_mobilis.WP_015279827
Candidatus_Thiosymbion_oneisti.WP_089728710
Thiorhodovibrio_sp_970.WP_009148650
Thiohalocapsa_sp_ML1.WP_058554608
Candidatus_Thiodiazotropha_endolucinida.WP_069121743
Candidatus_Thiodiazotropha_endoloripes.WP_068994032
Candidatus_Thiodiazotropha_endoloripes.WP_069006646
sulfur_oxidizing_symbionts.WP_005965268
endosymbiont_of_Ridgeia_piscesae.WP_057955541
Magnetofaba_australis.WP_085441805
Magnetococcus_marinus.WP_011713507
magneto_ovoid_bacterium_MO_1.CRH05183
Nocardia_elegans.WP_063022993
Corynebacteriales.WP_030515760
Actinoplanes_philippinensis.SFF54018
Actinoplanes.WP_014694766
Streptomyces_rubrolavendulae.WP_069979143
Streptomyces_thermolilacinus.WP_023591093
Streptomyces_sp_CNT372.WP_018848205
Streptomyces_sp_rh34.WP_097870956
Amycolatopsis.WP_038515702
Amycolatopsis_sp_MJM2582.WP_037343821
Amycolatopsis.WP_007033511
Amycolatopsis_orientalis_HCCB10007.AGM05236
Amycolatopsis_lurida.WP_034318139
Amycolatopsis_azurea_DSM_43854.EMD26753
Amycolatopsis.WP_039917837
Amycolatopsis.WP_020637318
Amycolatopsis.WP_037303795
uncultured_bacterium
magneto-ovoid_bacterium_MO-1
Bacteria
Nocardia
Actinoplanes
Streptomyces
Amycolatopsis
Nocardia_elegans
Actinoplanes_philippinensis
Streptomyces_rubrolavendulae
Streptomyces_thermolilacinus
Streptomyces_sp_CNT372
Streptomyces_sp_rh34
Amycolatopsis_lurida
Amycolatopsis_azurea_DSM_43854
Amycolatopsis_sp_MJM2582
Amycolatopsis_orientalis_HCCB10007
Proteobacteria
Ghiorsea_bivora
Acidithiobacillales_bacterium_SG8_45
Magnetococcaceae
Magnetococcus_marinus_MC-1
Magnetofaba_australis_IT-1
Epsilonproteobacteria
Nitratiruptor
Sulfurovum
Nitratiruptor_tergarcus
Nitratiruptor_sp_SB155-2
Sulfurovum_lithotrophicum
Sulfurovum_sp_NBC37-1
Betaproteobacteria
Gallionellales_bacterium_24-53-125
Betaproteobacteria_bacterium_SG8_41
Betaproteobacteria_bacterium_RBG_16_58_11
Betaproteobacteria_bacterium_RBG_19FT_COMBO_58_11
Betaproteobacteria_bacterium_RIFCSPLOWO2_02_FULL_67_26
Betaproteobacteria_bacterium_RIFCSPLOWO2_12_FULL_62_13
Gammaproteobacteria
Coxiellaceae
Coxiella
Chromatiaceae
Thiotrichales
Thiotrichaceae
Achromatium
Beggiatoa
Sedimenticola
Candidatus_Thiodiazotropha
Thorsellia_anophelis
Candidatus_Berkiella_cookevillensis
Coxiella_burnetii_RSA_493
Coxiella_sp_RIFCSPHIGHO2_12_FULL_44_14
Thiohalomonas_denitrificans
Thiohalobacter_thiocyanaticus
Thioflavicoccus_mobilis
Chromatiaceae_bacterium_2141TSTBD0c01a
Candidatus_Thiosymbion_oneisti
Thiohalocapsa_sp_ML1
Thiorhodovibrio_sp_970
Thioflexothrix_psekupsii
Candidatus_Thiomargarita_nelsonii
Thioploca_ingrica
Candidatus_Marithrix_sp_Canyon_246
Achromatium_sp_WMS2
Achromatium_sp_WMS3
Beggiatoa_leptomitoformis
Beggiatoa_alba
Beggiatoa_sp_PS
Thiohalorhabdus_denitrificans
Sedimenticola_thiotaurini
2548426
Sedimenticola_selenatireducens
Solemya_velesiana_gill_symbiont
endosymbiont_of_Ridgeia_piscesae
Candidatus_Thiodiazotropha_endoloripes
Candidatus_Thiodiazotropha_endolucinida
Gammaproteobacteria_bacterium_RIFCSPHIGHO2_02_FULL_42_43
Gammaproteobacteria_bacterium_RIFCSPHIGHO2_12_FULL_36_30
Gammaproteobacteria_bacterium_CG_4_10_14_0_2_um_filter_38_22
Gammaproteobacteria_bacterium_RIFCSPHIGHO2_12_FULL_38_11
Gammaproteobacteria_bacterium_RIFCSPHIGHO2_12_FULL_41_15
Gammaproteobacteria_bacterium_RIFCSPHIGHO2_12_FULL_42_13
Gammaproteobacteria_bacterium_RIFCSPHIGHO2_12_FULL_45_9
Gammaproteobacteria_bacterium_RIFOXYD12_FULL_61_37
endosymbiont_of_unidentified_scaly_snail_isolate_Monju
Gammaproteobacteria_bacterium_RIFCSPHIGHO2_02_FULL_39_13

CBU_0395

A

B

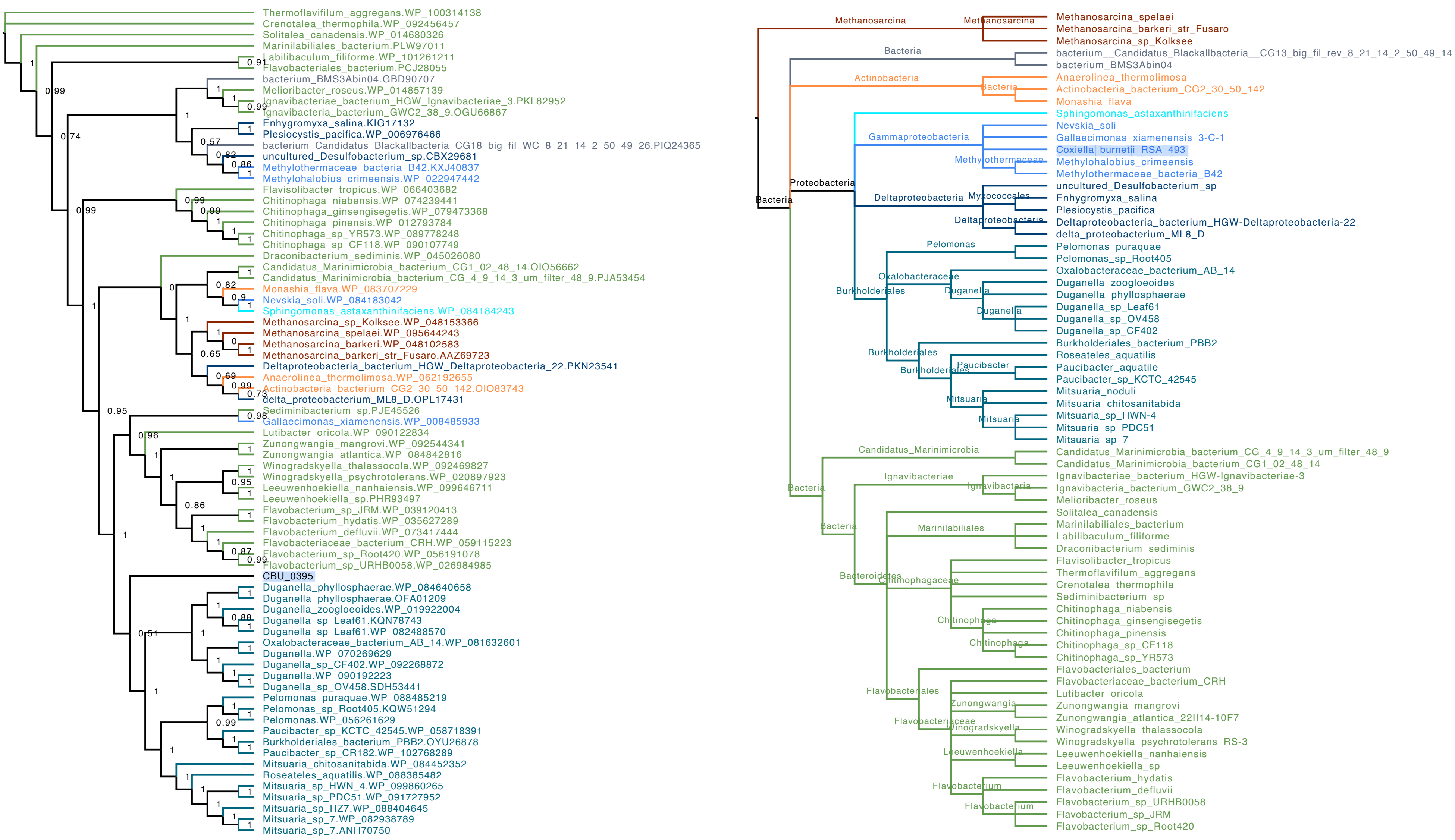
Thermoflavifilum_aggregans.WP_100314138
Crenotalea_thermophila.WP_092456457
Solitalea_canadensis.WP_014680326
Marinilabiliales_bacterium.PLW97011
Labilibaculum_filiforme.WP_101261211
Flavobacteriales_bacterium.PCJ28055
bacterium_BMS3Abin04.GBD90707
Melioribacter_roseus.WP_014857139
Ignavibacteriae_bacterium_HGW_Ignavibacteriae_3.PKL82952
Ignavibacteria_bacterium_GWC2_38_9.OGU66867
Enhygromyxa_salina.KIG17132
Plesiocystis_pacifica.WP_006976466
bacterium_Candidatus_Blackallbacteria_CG18_big_fil_WC_8_21_14_2_50_49_26.PIQ24365
uncultured_Desulfobacterium_sp.CBX29681
Methylothermaceae_bacteria_B42.KXJ40837
Methylohalobius_crimeensis.WP_022947442
Flavisolibacter_tropicus.WP_066403682
Chitinophaga_niabensis.WP_074239441
Chitinophaga_ginsengisegetis.WP_079473368
Chitinophaga_pinensis.WP_012793784
Chitinophaga_sp_YR573.WP_089778248
Chitinophaga_sp_CF118.WP_090107749
Draconibacterium_sediminis.WP_045026080
Candidatus_Marinimicrobia_bacterium_CG1_02_48_14.OIO56662
Candidatus_Marinimicrobia_bacterium_CG_4_9_14_3_um_filter_48_9.PJA53454
Monashia_flava.WP_083707229
Nevskia_soli.WP_084183042
Sphingomonas_astaxanthinifaciens.WP_084184243
Methanosarcina_sp_Kolksee.WP_048153366
Methanosarcina_spelaei.WP_095644243
Methanosarcina_barkeri.WP_048102583
Methanosarcina_barkeri_str_Fusaro.AAZ69723
Deltaproteobacteria_bacterium_HGW_Deltaproteobacteria_22.PKN23541
Anaerolinea_thermolimosa.WP_062192655
Actinobacteria_bacterium_CG2_30_50_142.OIO83743
delta_proteobacterium_ML8_D.OPL17431
Sediminibacterium_sp.PJE45526
Gallaecimonas_xiamenensis.WP_008485933
Lutibacter_oricola.WP_090122834
Zunongwangia_mangrovi.WP_092544341
Zunongwangia_atlantica.WP_084842816
Winogradskyella_thalassocola.WP_092469827
Winogradskyella_psychrotolerans.WP_020897923
Leeuwenhoekiella_nanhaiensis.WP_099646711
Leeuwenhoekiella_sp.PHR93497
Flavobacterium_sp_JRM.WP_039120413
Flavobacterium_hydatis.WP_035627289
Flavobacterium_defluvii.WP_073417444
Flavobacteriaceae_bacterium_CRH.WP_059115223
Flavobacterium_sp_Root420.WP_056191078
Flavobacterium_sp_URHB0058.WP_026984985
CBU_0395
Duganella_phyllosphaerae.WP_084640658
Duganella_phyllosphaerae.OFA01209
Duganella_zoogloeoides.WP_019922004
Duganella_sp_Leaf61.KQN78743
Duganella_sp_Leaf61.WP_082488570
Oxalobacteraceae_bacterium_AB_14.WP_081632601
Duganella.WP_070269629
Duganella_sp_CF402.WP_092268872
Duganella.WP_090192223
Duganella_sp_OV458.SDH53441
Pelomonas_puraquae.WP_088485219
Pelomonas_sp_Root405.KQW51294
Pelomonas.WP_056261629
Paucibacter_sp_KCTC_42545.WP_058718391
Burkholderiales_bacterium_PBB2.OYU26878
Paucibacter_sp_CR182.WP_102768289
Mitsuaria_chitosanitabida.WP_084452352
Roseateles_aquatilis.WP_088385482
Mitsuaria_sp_HWN_4.WP_099860265
Mitsuaria_sp_PDC51.WP_091727952
Mitsuaria_sp_HZ7.WP_088404645
Mitsuaria_sp_7.WP_082938789
Mitsuaria_sp_7.ANH70750
Methanosarcina_spelaei
Methanosarcina_barkeri_str_Fusaro
Methanosarcina_sp_Kolksee
bacterium__Candidatus_Blackallbacteria__CG13_big_fil_rev_8_21_14_2_50_49_14
bacterium_BMS3Abin04
Anaerolinea_thermolimosa
Actinobacteria_bacterium_CG2_30_50_142
Monashia_flava
Sphingomonas_astaxanthinifaciens
Nevskia_soli
Gallaecimonas_xiamenensis_3-C-1
Coxiella_burnetii_RSA_493
Methylohalobius_crimeensis
Methylothermaceae_bacteria_B42
uncultured_Desulfobacterium_sp
Enhygromyxa_salina
Plesiocystis_pacifica
Deltaproteobacteria_bacterium_HGW-Deltaproteobacteria-22
delta_proteobacterium_ML8_D
Pelomonas_puraquae
Pelomonas_sp_Root405
Oxalobacteraceae_bacterium_AB_14
Duganella_zoogloeoides
Duganella_phyllosphaerae
Duganella_sp_Leaf61
Duganella_sp_OV458
Duganella_sp_CF402
Burkholderiales_bacterium_PBB2
Roseateles_aquatilis
Paucibacter_aquatile
Paucibacter_sp_KCTC_42545
Mitsuaria_noduli
Mitsuaria_chitosanitabida
Mitsuaria_sp_HWN-4
Mitsuaria_sp_PDC51
Mitsuaria_sp_7
Candidatus_Marinimicrobia_bacterium_CG_4_9_14_3_um_filter_48_9
Candidatus_Marinimicrobia_bacterium_CG1_02_48_14
Ignavibacteriae_bacterium_HGW-Ignavibacteriae-3
Ignavibacteria_bacterium_GWC2_38_9
Melioribacter_roseus
Solitalea_canadensis
Marinilabiliales_bacterium
Labilibaculum_filiforme
Draconibacterium_sediminis
Flavisolibacter_tropicus
Thermoflavifilum_aggregans
Crenotalea_thermophila
Sediminibacterium_sp
Chitinophaga_niabensis
Chitinophaga_ginsengisegetis
Chitinophaga_pinensis
Chitinophaga_sp_CF118
Chitinophaga_sp_YR573
Flavobacteriales_bacterium
Flavobacteriaceae_bacterium_CRH
Lutibacter_oricola
Zunongwangia_mangrovi
Zunongwangia_atlantica_22II14-10F7
Winogradskyella_thalassocola
Winogradskyella_psychrotolerans_RS-3
Leeuwenhoekiella_nanhaiensis
Leeuwenhoekiella_sp
Flavobacterium_hydatis
Flavobacterium_defluvii
Flavobacterium_sp_URHB0058
Flavobacterium_sp_JRM
Flavobacterium_sp_Root420

CBU_0373

A B

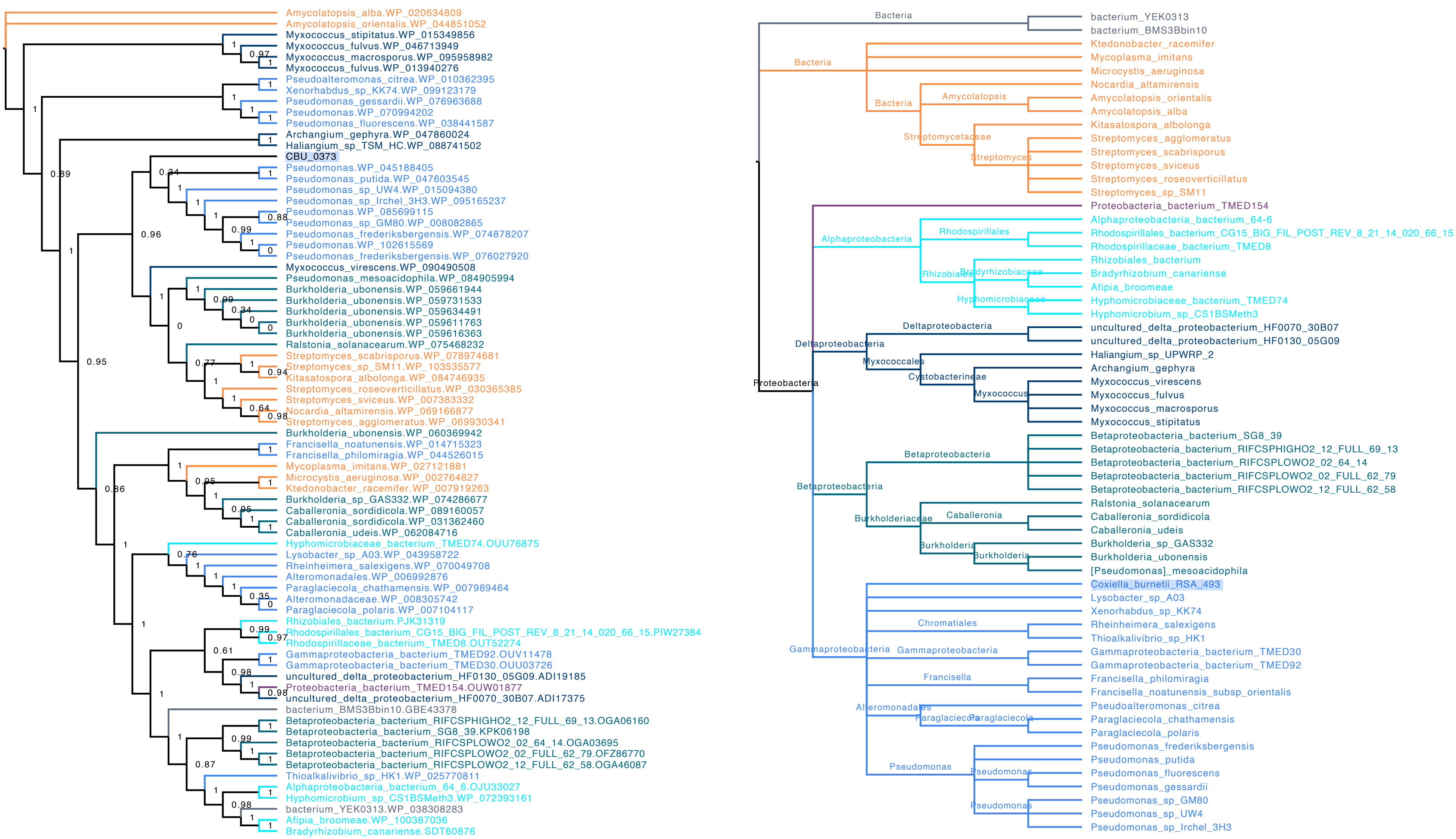

Amycolatopsis_alba.WP_020634809
Amycolatopsis_orientalis.WP_044851052
Myxococcus_stipitatus.WP_015349856
Myxococcus_fulvus.WP_046713949
Myxococcus_macrosporus.WP_095958982
Myxococcus_fulvus.WP_013940276
Pseudoalteromonas_citrea.WP_010362395
Xenorhabdus_sp_KK74.WP_099123179
Pseudomonas_gessardii.WP_076963688
Pseudomonas.WP_070994202
Pseudomonas_fluorescens.WP_038441587
Archangium_gephyra.WP_047860024
Haliangium_sp_TSM_HC.WP_088741502
CBU_0373
Pseudomonas.WP_045188405
Pseudomonas_putida.WP_047603545
Pseudomonas_sp_UW4.WP_015094380
Pseudomonas_sp_Irchel_3H3.WP_095165237
Pseudomonas.WP_085699115
Pseudomonas_sp_GM80.WP_008082865
Pseudomonas_frederiksbergensis.WP_074878207
Pseudomonas.WP_102615569
Pseudomonas_frederiksbergensis.WP_076027920
Myxococcus_virescens.WP_090490508
Pseudomonas_mesoacidophila.WP_084905994
Burkholderia_ubonensis.WP_059661944
Burkholderia_ubonensis.WP_059731533
Burkholderia_ubonensis.WP_059634491
Burkholderia_ubonensis.WP_059611763
Burkholderia_ubonensis.WP_059616363
Ralstonia_solanacearum.WP_075468232
Streptomyces_scabrisporus.WP_078974681
Streptomyces_sp_SM11.WP_103535577
Kitasatospora_albolonga.WP_084746935
Streptomyces_roseoverticillatus.WP_030365385
Streptomyces_sviceus.WP_007383332
Nocardia_altamirensis.WP_069166877
Streptomyces_agglomeratus.WP_069930341
Burkholderia_ubonensis.WP_060369942
Francisella_noatunensis.WP_014715323
Francisella_philomiragia.WP_044526015
Mycoplasma_imitans.WP_027121881
Microcystis_aeruginosa.WP_002764827
Ktedonobacter_racemifer.WP_007919263
Burkholderia_sp_GAS332.WP_074286677
Caballeronia_sordidicola.WP_089160057
Caballeronia_sordidicola.WP_031362460
Caballeronia_udeis.WP_062084716
Hyphomicrobiaceae_bacterium_TMED74.OUU76875
Lysobacter_sp_A03.WP_043958722
Rheinheimera_salexigens.WP_070049708
Alteromonadales.WP_006992876
Paraglaciecola_chathamensis.WP_007989464
Alteromonadaceae.WP_008305742
Paraglaciecola_polaris.WP_007104117
Rhizobiales_bacterium.PJK31319
Rhodospirillales_bacterium_CG15_BIG_FIL_POST_REV_8_21_14_020_66_15.PIW27384
Rhodospirillaceae_bacterium_TMED8.OUT52274
Gammaproteobacteria_bacterium_TMED92.OUV11478
Gammaproteobacteria_bacterium_TMED30.OUU03726
uncultured_delta_proteobacterium_HF0130_05G09.ADI19185
Proteobacteria_bacterium_TMED154.OUW01877
uncultured_delta_proteobacterium_HF0070_30B07.ADI17375
bacterium_BMS3Bbin10.GBE43378
Betaproteobacteria_bacterium_RIFCSPHIGHO2_12_FULL_69_13.OGA06160
Betaproteobacteria_bacterium_SG8_39.KPK06198
Betaproteobacteria_bacterium_RIFCSPLOWO2_02_64_14.OGA03695
Betaproteobacteria_bacterium_RIFCSPLOWO2_02_FULL_62_79.OFZ86770
Betaproteobacteria_bacterium_RIFCSPLOWO2_12_FULL_62_58.OGA46087
Thioalkalivibrio_sp_HK1.WP_025770811
Alphaproteobacteria_bacterium_64_6.OJU33027
Hyphomicrobium_sp_CS1BSMeth3.WP_072393161
bacterium_YEK0313.WP_038308283
Afipia_broomeae.WP_100387036
Bradyrhizobium_canariense.SDT60876
Bacteria
bacterium_YEK0313
bacterium_BMS3Bbin10
Ktedonobacter_racemifer
Mycoplasma_imitans
Microcystis_aeruginosa
Nocardia_altamirensis
Amycolatopsis
Amycolatopsis_orientalis
Amycolatopsis_alba
Kitasatospora_albolonga
Streptomycetaceae
Streptomyces_agglomeratus
Streptomyces
Streptomyces_scabrisporus
Streptomyces_sviceus
Streptomyces_roseoverticillatus
Streptomyces_sp_SM11
Proteobacteria_bacterium_TMED154
Alphaproteobacteria
Alphaproteobacteria_bacterium_64-6
Rhodospirillales
Rhodospirillales_bacterium_CG15_BIG_FIL_POST_REV_8_21_14_020_66_15
Rhodospirillaceae_bacterium_TMED8
Rhizobiales
Rhizobiales_bacterium
Bradyrhizobiaceae
Bradyrhizobium_canariense
Afipia_broomeae
Hyphomicrobiaceae
Hyphomicrobiaceae_bacterium_TMED74
Hyphomicrobium_sp_CS1BSMeth3
Deltaproteobacteria
uncultured_delta_proteobacterium_HF0070_30B07
uncultured_delta_proteobacterium_HF0130_05G09
Myxococcales
Haliangium_sp_UPWRP_2
Proteobacteria
Cystobacterineae
Archangium_gephyra
Myxococcus
Myxococcus_virescens
Myxococcus_fulvus
Myxococcus_macrosporus
Myxococcus_stipitatus
Betaproteobacteria
Betaproteobacteria_bacterium_SG8_39
Betaproteobacteria_bacterium_RIFCSPHIGHO2_12_FULL_69_13
Betaproteobacteria_bacterium_RIFCSPLOWO2_02_64_14
Betaproteobacteria_bacterium_RIFCSPLOWO2_02_FULL_62_79
Betaproteobacteria_bacterium_RIFCSPLOWO2_12_FULL_62_58
Ralstonia_solanacearum
Burkholderiaceae
Caballeronia
Caballeronia_sordidicola
Caballeronia_udeis
Burkholderia
Burkholderia_sp_GAS332
Burkholderia_ubonensis
[Pseudomonas]_mesoacidophila
Coxiella_burnetii_RSA_493
Lysobacter_sp_A03
Xenorhabdus_sp_KK74
Chromatiales
Rheinheimera_salexigens
Thioalkalivibrio_sp_HK1
Gammaproteobacteria
Gammaproteobacteria_bacterium_TMED30
Gammaproteobacteria_bacterium_TMED92
Francisella
Francisella_philomiragia
Francisella_noatunensis_subsp_orientalis
Alteromonadales
Pseudoalteromonas_citrea
Paraglaciecola
Paraglaciecola_chathamensis
Paraglaciecola_polaris
Pseudomonas
Pseudomonas_frederiksbergensis
Pseudomonas_putida
Pseudomonas_fluorescens
Pseudomonas_gessardii
Pseudomonas_sp_GM80
Pseudomonas_sp_UW4
Pseudomonas_sp_Irchel_3H3

CBU_0284

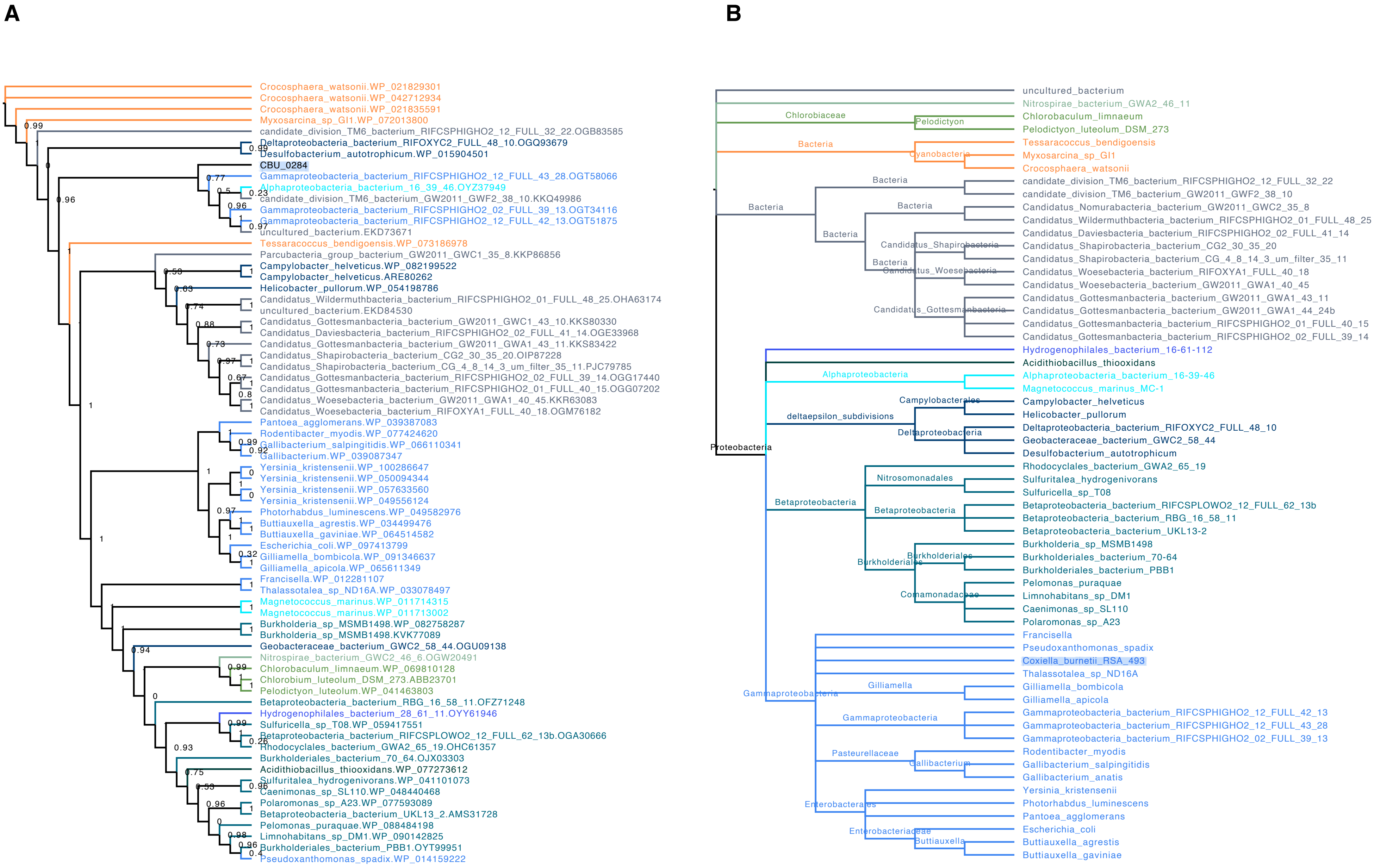

A
B
Crocosphaera_watsonii.WP_021829301
Crocosphaera_watsonii.WP_042712934
Crocosphaera_watsonii.WP_021835591
Myxosarcina_sp_GI1.WP_072013800
candidate_division_TM6_bacterium_RIFCSPHIGHO2_12_FULL_32_22.OGB83585
Deltaproteobacteria_bacterium_RIFOXYC2_FULL_48_10.OGQ93679
Desulfobacterium_autotrophicum.WP_015904501
CBU_0284
Gammaproteobacteria_bacterium_RIFCSPHIGHO2_12_FULL_43_28.OGT58066
Alphaproteobacteria_bacterium_16_39_46.OYZ37949
candidate_division_TM6_bacterium_GW2011_GWF2_38_10.KKQ49986
Gammaproteobacteria_bacterium_RIFCSPHIGHO2_02_FULL_39_13.OGT34116
Gammaproteobacteria_bacterium_RIFCSPHIGHO2_12_FULL_42_13.OGT51875
uncultured_bacterium.EKD73671
Tessaracoccus_bendigoensis.WP_073186978
Parcubacteria_group_bacterium_GW2011_GWC1_35_8.KKP86856
Campylobacter_helveticus.WP_082199522
Campylobacter_helveticus.ARE80262
Helicobacter_pullorum.WP_054198786
Candidatus_Wildermuthbacteria_bacterium_RIFCSPHIGHO2_01_FULL_48_25.OHA63174
uncultured_bacterium.EKD84530
Candidatus_Gottesmanbacteria_bacterium_GW2011_GWC1_43_10.KKS80330
Candidatus_Daviesbacteria_bacterium_RIFCSPHIGHO2_02_FULL_41_14.OGE33968
Candidatus_Gottesmanbacteria_bacterium_GW2011_GWA1_43_11.KKS83422
Candidatus_Shapirobacteria_bacterium_CG2_30_35_20.OIP87228
Candidatus_Shapirobacteria_bacterium_CG_4_8_14_3_um_filter_35_11.PJC79785
Candidatus_Gottesmanbacteria_bacterium_RIFCSPHIGHO2_02_FULL_39_14.OGG17440
Candidatus_Gottesmanbacteria_bacterium_RIFCSPHIGHO2_01_FULL_40_15.OGG07202
Candidatus_Woesebacteria_bacterium_GW2011_GWA1_40_45.KKR63083
Candidatus_Woesebacteria_bacterium_RIFOXYA1_FULL_40_18.OGM76182
Pantoea_agglomerans.WP_039387083
Rodentibacter_myodis.WP_077424620
Gallibacterium_salpingitidis.WP_066110341
Gallibacterium.WP_039087347
Yersinia_kristensenii.WP_100286647
Yersinia_kristensenii.WP_050094344
Yersinia_kristensenii.WP_057633560
Yersinia_kristensenii.WP_049556124
Photorhabdus_luminescens.WP_049582976
Buttiauxella_agrestis.WP_034499476
Buttiauxella_gaviniae.WP_064514582
Escherichia_coli.WP_097413799
Gilliamella_bombicola.WP_091346637
Gilliamella_apicola.WP_065611349
Francisella.WP_012281107
Thalassotalea_sp_ND16A.WP_033078497
Magnetococcus_marinus.WP_011714315
Magnetococcus_marinus.WP_011713002
Burkholderia_sp_MSMB1498.WP_082758287
Burkholderia_sp_MSMB1498.KVK77089
Geobacteraceae_bacterium_GWC2_58_44.OGU09138
Nitrospirae_bacterium_GWC2_46_6.OGW20491
Chlorobaculum_limnaeum.WP_069810128
Chlorobium_luteolum_DSM_273.ABB23701
Pelodictyon_luteolum.WP_041463803
Betaproteobacteria_bacterium_RBG_16_58_11.OFZ71248
Hydrogenophilales_bacterium_28_61_11.OYY61946
Sulfuricella_sp_T08.WP_059417551
Betaproteobacteria_bacterium_RIFCSPLOWO2_12_FULL_62_13b.OGA30666
Rhodocyclales_bacterium_GWA2_65_19.OHC61357
Burkholderiales_bacterium_70_64.OJX03303
Acidithiobacillus_thiooxidans.WP_077273612
Sulfuritalea_hydrogenivorans.WP_041101073
Caenimonas_sp_SL110.WP_048440468
Polaromonas_sp_A23.WP_077593089
Betaproteobacteria_bacterium_UKL13_2.AMS31728
Pelomonas_puraquae.WP_088484198
Limnohabitans_sp_DM1.WP_090142825
Burkholderiales_bacterium_PBB1.OYT99951
Pseudoxanthomonas_spadix.WP_014159222
uncultured_bacterium
Nitrospirae_bacterium_GWA2_46_11
Chlorobiaceae
Pelodictyon
Chlorobaculum_limnaeum
Pelodictyon_luteolum_DSM_273
Bacteria
Cyanobacteria
Tessaracoccus_bendigoensis
Myxosarcina_sp_GI1
Crocosphaera_watsonii
candidate_division_TM6_bacterium_RIFCSPHIGHO2_12_FULL_32_22
candidate_division_TM6_bacterium_GW2011_GWF2_38_10
Candidatus_Nomurabacteria_bacterium_GW2011_GWC2_35_8
Candidatus_Wildermuthbacteria_bacterium_RIFCSPHIGHO2_01_FULL_48_25
Candidatus_Daviesbacteria_bacterium_RIFCSPHIGHO2_02_FULL_41_14
Candidatus_Shapirobacteria
Candidatus_Shapirobacteria_bacterium_CG2_30_35_20
Candidatus_Shapirobacteria_bacterium_CG_4_8_14_3_um_filter_35_11
Candidatus_Woesebacteria
Candidatus_Woesebacteria_bacterium_RIFOXYA1_FULL_40_18
Candidatus_Woesebacteria_bacterium_GW2011_GWA1_40_45
Candidatus_Gottesmanbacteria
Candidatus_Gottesmanbacteria_bacterium_GW2011_GWA1_43_11
Candidatus_Gottesmanbacteria_bacterium_GW2011_GWA1_44_24b
Candidatus_Gottesmanbacteria_bacterium_RIFCSPHIGHO2_01_FULL_40_15
Candidatus_Gottesmanbacteria_bacterium_RIFCSPHIGHO2_02_FULL_39_14
Proteobacteria
Hydrogenophilales_bacterium_16-61-112
Acidithiobacillus_thiooxidans
Alphaproteobacteria
Alphaproteobacteria_bacterium_16-39-46
Magnetococcus_marinus_MC-1
deltaepsilon_subdivisions
Campylobacterales
Campylobacter_helveticus
Helicobacter_pullorum
Deltaproteobacteria
Deltaproteobacteria_bacterium_RIFOXYC2_FULL_48_10
Geobacteraceae_bacterium_GWC2_58_44
Desulfobacterium_autotrophicum
Betaproteobacteria
Nitrosomonadales
Rhodocyclales_bacterium_GWA2_65_19
Sulfuritalea_hydrogenivorans
Sulfuricella_sp_T08
Betaproteobacteria_bacterium_RIFCSPLOWO2_12_FULL_62_13b
Betaproteobacteria_bacterium_RBG_16_58_11
Betaproteobacteria_bacterium_UKL13-2
Burkholderiales
Burkholderia_sp_MSMB1498
Burkholderiales_bacterium_70-64
Burkholderiales_bacterium_PBB1
Comamonadaceae
Pelomonas_puraquae
Limnohabitans_sp_DM1
Caenimonas_sp_SL110
Polaromonas_sp_A23
Gammaproteobacteria
Francisella
Pseudoxanthomonas_spadix
Coxiella_burnetii_RSA_493
Thalassotalea_sp_ND16A
Gilliamella
Gilliamella_bombicola
Gilliamella_apicola
Gammaproteobacteria_bacterium_RIFCSPHIGHO2_12_FULL_42_13
Gammaproteobacteria_bacterium_RIFCSPHIGHO2_12_FULL_43_28
Gammaproteobacteria_bacterium_RIFCSPHIGHO2_02_FULL_39_13
Pasteurellaceae
Gallibacterium
Rodentibacter_myodis
Gallibacterium_salpingitidis
Gallibacterium_anatis
Enterobacterales
Enterobacteriaceae
Buttiauxella
Yersinia_kristensenii
Photorhabdus_luminescens
Pantoea_agglomerans
Escherichia_coli
Buttiauxella_agrestis
Buttiauxella_gaviniae

CBU_0184, CBU_0193

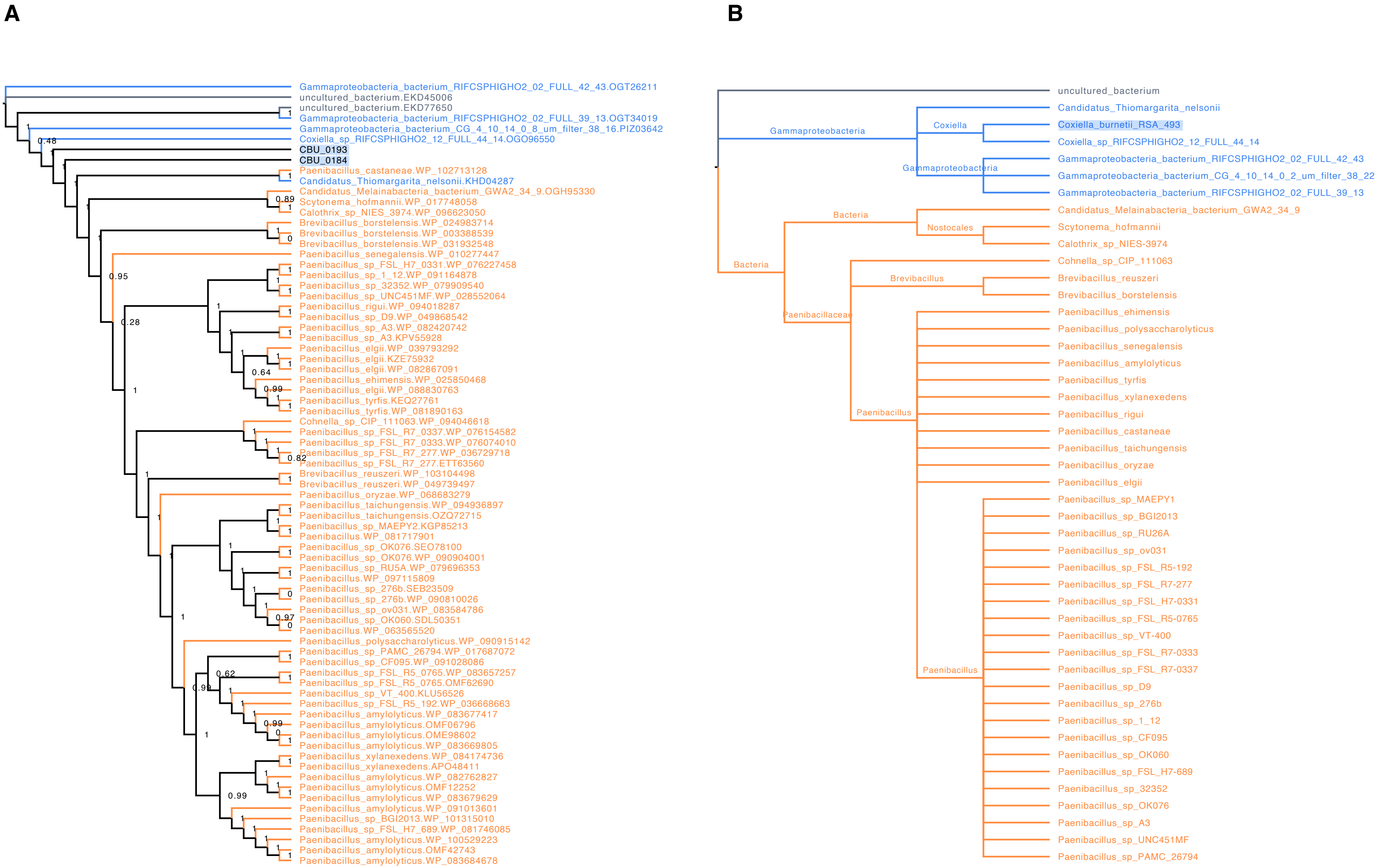

CBU_0182

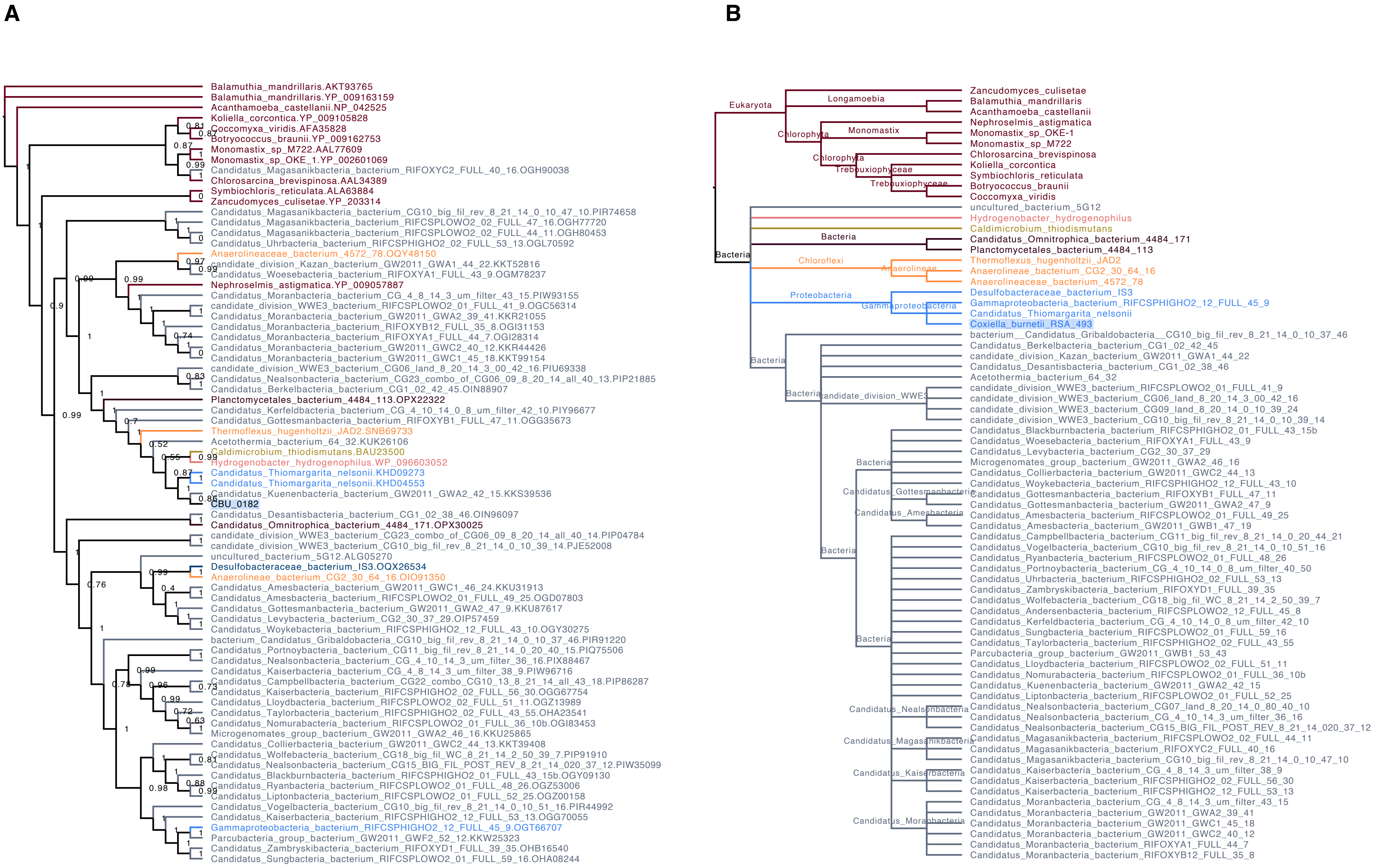

A
Balamuthia_mandrillaris.AKT93765
Balamuthia_mandrillaris.YP_009163159
Acanthamoeba_castellanii.NP_042525
Koliella_corcontica.YP_009105828
Coccomyxa_viridis.AFA35828
Botryococcus_braunii.YP_009162753
Monomastix_sp_M722.AAL77609
Monomastix_sp_OKE_1.YP_002601069
Candidatus_Magasanikbacteria_bacterium_RIFOXYC2_FULL_40_16.OGH90038
Chlorosarcina_brevispinosa.AAL34389
Symbiochloris_reticulata.ALA63884
Zancudomyces_culisetae.YP_203314
Candidatus_Magasanikbacteria_bacterium_CG10_big_fil_rev_8_21_14_0_10_47_10.PIR74658
Candidatus_Magasanikbacteria_bacterium_RIFCSPLOWO2_02_FULL_47_16.OGH77720
Candidatus_Magasanikbacteria_bacterium_RIFCSPLOWO2_02_FULL_44_11.OGH80453
Candidatus_Uhrbacteria_bacterium_RIFCSPHIGHO2_02_FULL_53_13.OGL70592
Anaerolineaceae_bacterium_4572_78.OQY48150
candidate_division_Kazan_bacterium_GW2011_GWA1_44_22.KKT52816
Candidatus_Woesebacteria_bacterium_RIFOXYA1_FULL_43_9.OGM78237
Nephroselmis_astigmatica.YP_009057887
Candidatus_Moranbacteria_bacterium_CG_4_8_14_3_um_filter_43_15.PIW93155
candidate_division_WWE3_bacterium_RIFCSPLOWO2_01_FULL_41_9.OGC56314
Candidatus_Moranbacteria_bacterium_GW2011_GWA2_39_41.KKR21055
Candidatus_Moranbacteria_bacterium_RIFOXYB12_FULL_35_8.OGI31153
Candidatus_Moranbacteria_bacterium_RIFOXYA1_FULL_44_7.OGI28314
Candidatus_Moranbacteria_bacterium_GW2011_GWC2_40_12.KKR44426
Candidatus_Moranbacteria_bacterium_GW2011_GWC1_45_18.KKT99154
candidate_division_WWE3_bacterium_CG06_land_8_20_14_3_00_42_16.PIU69338
Candidatus_Nealsonbacteria_bacterium_CG23_combo_of_CG06_09_8_20_14_all_40_13.PIP21885
Candidatus_Berkelbacteria_bacterium_CG1_02_42_45.OIN88907
Planctomycetales_bacterium_4484_113.OPX22322
Candidatus_Kerfeldbacteria_bacterium_CG_4_10_14_0_8_um_filter_42_10.PIY96677
Candidatus_Gottesmanbacteria_bacterium_RIFOXYB1_FULL_47_11.OGG35673
Thermoflexus_hugenholtzii_JAD2.SNB69733
Acetothermia_bacterium_64_32.KUK26106
Caldimicrobium_thiodismutans.BAU23500
Hydrogenobacter_hydrogenophilus.WP_096603052
Candidatus_Thiomargarita_nelsonii.KHD09273
Candidatus_Thiomargarita_nelsonii.KHD04553
Candidatus_Kuenenbacteria_bacterium_GW2011_GWA2_42_15.KKS39536
CBU_0182
Candidatus_Desantisbacteria_bacterium_CG1_02_38_46.OIN96097
Candidatus_Omnitrophica_bacterium_4484_171.OPX30025
candidate_division_WWE3_bacterium_CG23_combo_of_CG06_09_8_20_14_all_40_14.PIP04784
candidate_division_WWE3_bacterium_CG10_big_fil_rev_8_21_14_0_10_39_14.PJE52008
uncultured_bacterium_5G12.ALG05270
Desulfobacteraceae_bacterium_IS3.OQX26534
Anaerolineae_bacterium_CG2_30_64_16.OIO91350
Candidatus_Amesbacteria_bacterium_GW2011_GWC1_46_24.KKU31913
Candidatus_Amesbacteria_bacterium_RIFCSPLOWO2_01_FULL_49_25.OGD07803
Candidatus_Gottesmanbacteria_bacterium_GW2011_GWA2_47_9.KKU87617
Candidatus_Levybacteria_bacterium_CG2_30_37_29.OIP57459
Candidatus_Woykebacteria_bacterium_RIFCSPHIGHO2_12_FULL_43_10.OGY30275
bacterium_Candidatus_Gribaldobacteria_CG10_big_fil_rev_8_21_14_0_10_37_46.PIR91220
Candidatus_Portnoybacteria_bacterium_CG11_big_fil_rev_8_21_14_0_20_40_15.PIQ75506
Candidatus_Nealsonbacteria_bacterium_CG_4_10_14_3_um_filter_36_16.PIX88467
Candidatus_Kaiserbacteria_bacterium_CG_4_8_14_3_um_filter_38_9.PIW96716
Candidatus_Campbellbacteria_bacterium_CG22_combo_CG10_13_8_21_14_all_43_18.PIP86287
Candidatus_Kaiserbacteria_bacterium_RIFCSPHIGHO2_02_FULL_56_30.OGG67754
Candidatus_Lloydbacteria_bacterium_RIFCSPLOWO2_02_FULL_51_11.OGZ13989
Candidatus_Taylorbacteria_bacterium_RIFCSPHIGHO2_02_FULL_43_55.OHA23541
Candidatus_Nomurabacteria_bacterium_RIFCSPLOWO2_01_FULL_36_10b.OGI83453
Microgenomates_group_bacterium_GW2011_GWA2_46_16.KKU25865
Candidatus_Collierbacteria_bacterium_GW2011_GWC2_44_13.KKT39408
Candidatus_Wolfebacteria_bacterium_CG18_big_fil_WC_8_21_14_2_50_39_7.PIP91910
Candidatus_Nealsonbacteria_bacterium_CG15_BIG_FIL_POST_REV_8_21_14_020_37_12.PIW35099
Candidatus_Blackburnbacteria_bacterium_RIFCSPHIGHO2_01_FULL_43_15b.OGY09130
Candidatus_Ryanbacteria_bacterium_RIFCSPLOWO2_01_FULL_48_26.OGZ53006
Candidatus_Liptonbacteria_bacterium_RIFCSPLOWO2_01_FULL_52_25.OGZ00158
Candidatus_Vogelbacteria_bacterium_CG10_big_fil_rev_8_21_14_0_10_51_16.PIR44992
Candidatus_Kaiserbacteria_bacterium_RIFCSPHIGHO2_12_FULL_53_13.OGG70055
Gammaproteobacteria_bacterium_RIFCSPHIGHO2_12_FULL_45_9.OGT66707
Parcubacteria_group_bacterium_GW2011_GWF2_52_12.KKW25323
Candidatus_Zambryskibacteria_bacterium_RIFOXYD1_FULL_39_35.OHB16540
Candidatus_Sungbacteria_bacterium_RIFCSPLOWO2_01_FULL_59_16.OHA08244
B
Eukaryota
Longamoebia
Chlorophyta
Monomastix
Trebouxiophyceae
Bacteria
Chloroflexi
Anaerolineae
Proteobacteria
Gammaproteobacteria
candidate_division_WWE3
Candidatus_Gottesmanbacteria
Candidatus_Amesbacteria
Candidatus_Nealsonbacteria
Candidatus_Magasanikbacteria
Candidatus_Kaiserbacteria
Candidatus_Moranbacteria
Zancudomyces_culisetae
Balamuthia_mandrillaris
Acanthamoeba_castellanii
Nephroselmis_astigmatica
Monomastix_sp_OKE-1
Monomastix_sp_M722
Chlorosarcina_brevispinosa
Koliella_corcontica
Symbiochloris_reticulata
Botryococcus_braunii
Coccomyxa_viridis
uncultured_bacterium_5G12
Hydrogenobacter_hydrogenophilus
Caldimicrobium_thiodismutans
Candidatus_Omnitrophica_bacterium_4484_171
Planctomycetales_bacterium_4484_113
Thermoflexus_hugenholtzii_JAD2
Anaerolineae_bacterium_CG2_30_64_16
Anaerolineaceae_bacterium_4572_78
Desulfobacteraceae_bacterium_IS3
Gammaproteobacteria_bacterium_RIFCSPHIGHO2_12_FULL_45_9
Candidatus_Thiomargarita_nelsonii
Coxiella_burnetii_RSA_493
bacterium__Candidatus_Gribaldobacteria__CG10_big_fil_rev_8_21_14_0_10_37_46
Candidatus_Berkelbacteria_bacterium_CG1_02_42_45
candidate_division_Kazan_bacterium_GW2011_GWA1_44_22
Candidatus_Desantisbacteria_bacterium_CG1_02_38_46
Acetothermia_bacterium_64_32
candidate_division_WWE3_bacterium_RIFCSPLOWO2_01_FULL_41_9
candidate_division_WWE3_bacterium_CG06_land_8_20_14_3_00_42_16
candidate_division_WWE3_bacterium_CG09_land_8_20_14_0_10_39_24
candidate_division_WWE3_bacterium_CG10_big_fil_rev_8_21_14_0_10_39_14
Candidatus_Blackburnbacteria_bacterium_RIFCSPHIGHO2_01_FULL_43_15b
Candidatus_Woesebacteria_bacterium_RIFOXYA1_FULL_43_9
Candidatus_Levybacteria_bacterium_CG2_30_37_29
Microgenomates_group_bacterium_GW2011_GWA2_46_16
Candidatus_Collierbacteria_bacterium_GW2011_GWC2_44_13
Candidatus_Woykebacteria_bacterium_RIFCSPHIGHO2_12_FULL_43_10
Candidatus_Gottesmanbacteria_bacterium_RIFOXYB1_FULL_47_11
Candidatus_Gottesmanbacteria_bacterium_GW2011_GWA2_47_9
Candidatus_Amesbacteria_bacterium_RIFCSPLOWO2_01_FULL_49_25
Candidatus_Amesbacteria_bacterium_GW2011_GWB1_47_19
Candidatus_Campbellbacteria_bacterium_CG11_big_fil_rev_8_21_14_0_20_44_21
Candidatus_Vogelbacteria_bacterium_CG10_big_fil_rev_8_21_14_0_10_51_16
Candidatus_Ryanbacteria_bacterium_RIFCSPLOWO2_01_FULL_48_26
Candidatus_Portnoybacteria_bacterium_CG_4_10_14_0_8_um_filter_40_50
Candidatus_Uhrbacteria_bacterium_RIFCSPHIGHO2_02_FULL_53_13
Candidatus_Zambryskibacteria_bacterium_RIFOXYD1_FULL_39_35
Candidatus_Wolfebacteria_bacterium_CG18_big_fil_WC_8_21_14_2_50_39_7
Candidatus_Andersenbacteria_bacterium_RIFCSPHIGHO2_12_FULL_45_8
Candidatus_Kerfeldbacteria_bacterium_CG_4_10_14_0_8_um_filter_42_10
Candidatus_Sungbacteria_bacterium_RIFCSPLOWO2_01_FULL_59_16
Candidatus_Taylorbacteria_bacterium_RIFCSPHIGHO2_02_FULL_43_55
Parcubacteria_group_bacterium_GW2011_GWB1_53_43
Candidatus_Lloydbacteria_bacterium_RIFCSPLOWO2_02_FULL_51_11
Candidatus_Nomurabacteria_bacterium_RIFCSPLOWO2_01_FULL_36_10b
Candidatus_Kuenenbacteria_bacterium_GW2011_GWA2_42_15
Candidatus_Liptonbacteria_bacterium_RIFCSPLOWO2_01_FULL_52_25
Candidatus_Nealsonbacteria_bacterium_CG07_land_8_20_14_0_80_40_10
Candidatus_Nealsonbacteria_bacterium_CG_4_10_14_3_um_filter_36_16
Candidatus_Nealsonbacteria_bacterium_CG15_BIG_FIL_POST_REV_8_21_14_020_37_12
Candidatus_Magasanikbacteria_bacterium_RIFCSPLOWO2_02_FULL_44_11
Candidatus_Magasanikbacteria_bacterium_RIFOXYC2_FULL_40_16
Candidatus_Magasanikbacteria_bacterium_CG10_big_fil_rev_8_21_14_0_10_47_10
Candidatus_Kaiserbacteria_bacterium_CG_4_8_14_3_um_filter_38_9
Candidatus_Kaiserbacteria_bacterium_RIFCSPHIGHO2_02_FULL_56_30
Candidatus_Kaiserbacteria_bacterium_RIFCSPHIGHO2_12_FULL_53_13
Candidatus_Moranbacteria_bacterium_CG_4_8_14_3_um_filter_43_15
Candidatus_Moranbacteria_bacterium_GW2011_GWA2_39_41
Candidatus_Moranbacteria_bacterium_GW2011_GWC1_45_18
Candidatus_Moranbacteria_bacterium_GW2011_GWC2_40_12
Candidatus_Moranbacteria_bacterium_RIFOXYA1_FULL_44_7
Candidatus_Moranbacteria_bacterium_RIFOXYB12_FULL_35_8

CBU_0175

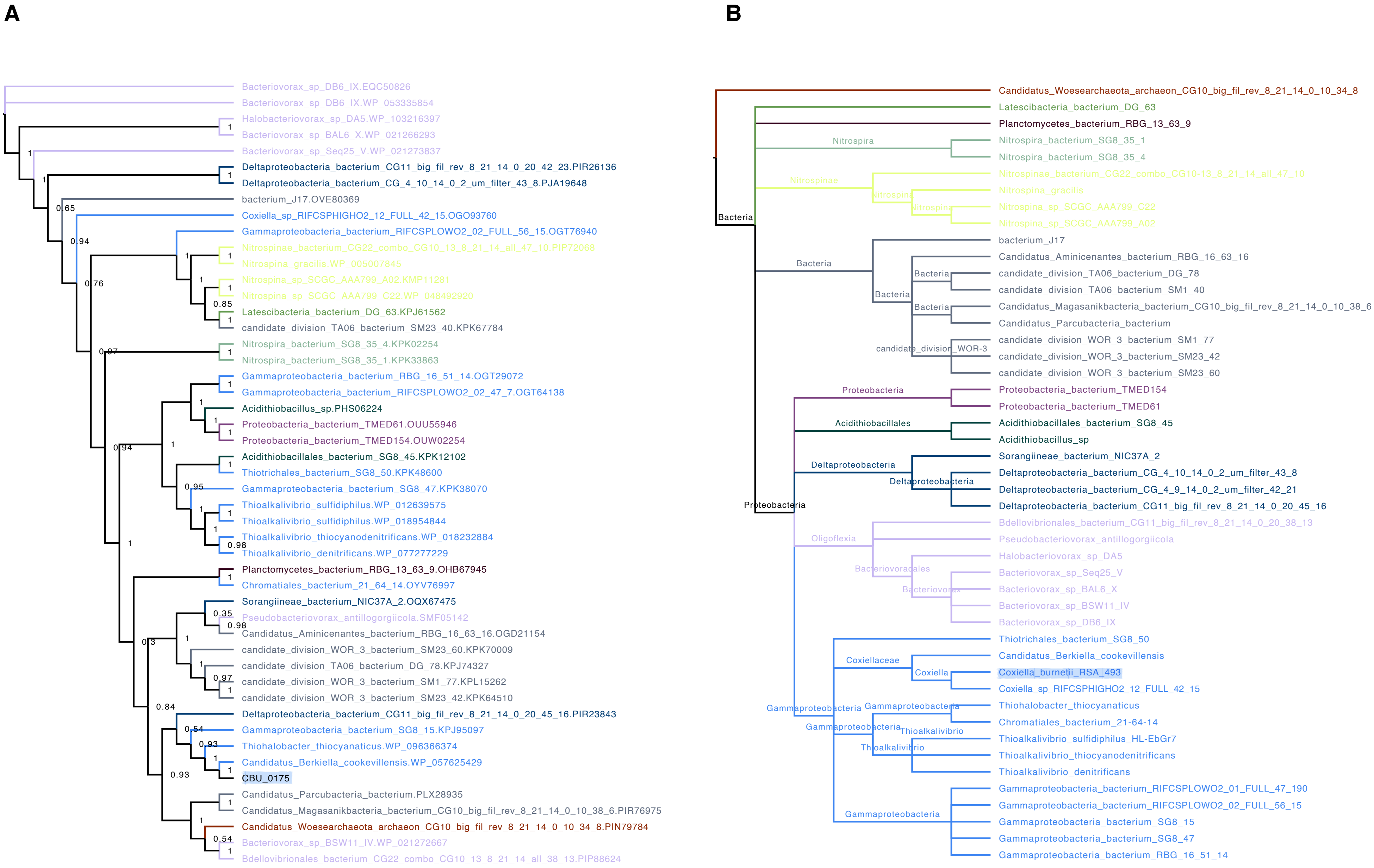
A
Bacteriovorax_sp_DB6_IX.EQC50826
Bacteriovorax_sp_DB6_IX.WP_053335854
Halobacteriovorax_sp_DA5.WP_103216397
Bacteriovorax_sp_BAL6_X.WP_021266293
Bacteriovorax_sp_Seq25_V.WP_021273837
Deltaproteobacteria_bacterium_CG11_big_fil_rev_8_21_14_0_20_42_23.PIR26136
Deltaproteobacteria_bacterium_CG_4_10_14_0_2_um_filter_43_8.PJA19648
bacterium_J17.OVE80369
Coxiella_sp_RIFCSPHIGHO2_12_FULL_42_15.OGO93760
Gammaproteobacteria_bacterium_RIFCSPLOWO2_02_FULL_56_15.OGT76940
Nitrospinae_bacterium_CG22_combo_CG10_13_8_21_14_all_47_10.PIP72068
Nitrospina_gracilis.WP_005007845
Nitrospina_sp_SCGC_AAA799_A02.KMP11281
Nitrospina_sp_SCGC_AAA799_C22.WP_048492920
Latescibacteria_bacterium_DG_63.KPJ61562
candidate_division_TA06_bacterium_SM23_40.KPK67784
Nitrospira_bacterium_SG8_35_4.KPK02254
Nitrospira_bacterium_SG8_35_1.KPK33863
Gammaproteobacteria_bacterium_RBG_16_51_14.OGT29072
Gammaproteobacteria_bacterium_RIFCSPLOWO2_02_47_7.OGT64138
Acidithiobacillus_sp.PHS06224
Proteobacteria_bacterium_TMED61.OUU55946
Proteobacteria_bacterium_TMED154.OUW02254
Acidithiobacillales_bacterium_SG8_45.KPK12102
Thiotrichales_bacterium_SG8_50.KPK48600
Gammaproteobacteria_bacterium_SG8_47.KPK38070
Thioalkalivibrio_sulfidiphilus.WP_012639575
Thioalkalivibrio_sulfidiphilus.WP_018954844
Thioalkalivibrio_thiocyanodenitrificans.WP_018232884
Thioalkalivibrio_denitrificans.WP_077277229
Planctomycetes_bacterium_RBG_13_63_9.OHB67945
Chromatiales_bacterium_21_64_14.OYV76997
Sorangiineae_bacterium_NIC37A_2.OQX67475
Pseudobacteriovorax_antillogorgiicola.SMF05142
Candidatus_Aminicenantes_bacterium_RBG_16_63_16.OGD21154
candidate_division_WOR_3_bacterium_SM23_60.KPK70009
candidate_division_TA06_bacterium_DG_78.KPJ74327
candidate_division_WOR_3_bacterium_SM1_77.KPL15262
candidate_division_WOR_3_bacterium_SM23_42.KPK64510
Deltaproteobacteria_bacterium_CG11_big_fil_rev_8_21_14_0_20_45_16.PIR23843
Gammaproteobacteria_bacterium_SG8_15.KPJ95097
Thiohalobacter_thiocyanaticus.WP_096366374
Candidatus_Berkiella_cookevillensis.WP_057625429
CBU_0175
Candidatus_Parcubacteria_bacterium.PLX28935
Candidatus_Magasanikbacteria_bacterium_CG10_big_fil_rev_8_21_14_0_10_38_6.PIR76975
Candidatus_Woesearchaeota_archaeon_CG10_big_fil_rev_8_21_14_0_10_34_8.PIN79784
Bacteriovorax_sp_BSW11_IV.WP_021272667
Bdellovibrionales_bacterium_CG22_combo_CG10_13_8_21_14_all_38_13.PIP88624
B
Candidatus_Woesearchaeota_archaeon_CG10_big_fil_rev_8_21_14_0_10_34_8
Latescibacteria_bacterium_DG_63
Planctomycetes_bacterium_RBG_13_63_9
Nitrospira
Nitrospira_bacterium_SG8_35_1
Nitrospira_bacterium_SG8_35_4
Nitrospinae
Nitrospina
Nitrospinae_bacterium_CG22_combo_CG10-13_8_21_14_all_47_10
Nitrospina_gracilis
Nitrospina_sp_SCGC_AAA799_C22
Nitrospina_sp_SCGC_AAA799_A02
Bacteria
bacterium_J17
Candidatus_Aminicenantes_bacterium_RBG_16_63_16
candidate_division_TA06_bacterium_DG_78
candidate_division_TA06_bacterium_SM1_40
Candidatus_Magasanikbacteria_bacterium_CG10_big_fil_rev_8_21_14_0_10_38_6
Candidatus_Parcubacteria_bacterium
candidate_division_WOR-3
candidate_division_WOR_3_bacterium_SM1_77
candidate_division_WOR_3_bacterium_SM23_42
candidate_division_WOR_3_bacterium_SM23_60
Proteobacteria
Proteobacteria_bacterium_TMED154
Proteobacteria_bacterium_TMED61
Acidithiobacillales
Acidithiobacillales_bacterium_SG8_45
Acidithiobacillus_sp
Deltaproteobacteria
Sorangiineae_bacterium_NIC37A_2
Deltaproteobacteria_bacterium_CG_4_10_14_0_2_um_filter_43_8
Deltaproteobacteria_bacterium_CG_4_9_14_0_2_um_filter_42_21
Deltaproteobacteria_bacterium_CG11_big_fil_rev_8_21_14_0_20_45_16
Oligoflexia
Bdellovibrionales_bacterium_CG11_big_fil_rev_8_21_14_0_20_38_13
Pseudobacteriovorax_antillogorgiicola
Bacteriovoracales
Bacteriovorax
Halobacteriovorax_sp_DA5
Bacteriovorax_sp_Seq25_V
Bacteriovorax_sp_BAL6_X
Bacteriovorax_sp_BSW11_IV
Bacteriovorax_sp_DB6_IX
Gammaproteobacteria
Coxiellaceae
Coxiella
Thiotrichales_bacterium_SG8_50
Candidatus_Berkiella_cookevillensis
Coxiella_burnetii_RSA_493
Coxiella_sp_RIFCSPHIGHO2_12_FULL_42_15
Thiohalobacter_thiocyanaticus
Chromatiales_bacterium_21-64-14
Thioalkalivibrio
Thioalkalivibrio_sulfidiphilus_HL-EbGr7
Thioalkalivibrio_thiocyanodenitrificans
Thioalkalivibrio_denitrificans
Gammaproteobacteria_bacterium_RIFCSPLOWO2_01_FULL_47_190
Gammaproteobacteria_bacterium_RIFCSPLOWO2_02_FULL_56_15
Gammaproteobacteria_bacterium_SG8_15
Gammaproteobacteria_bacterium_SG8_47
Gammaproteobacteria_bacterium_RBG_16_51_14

CBU_0168

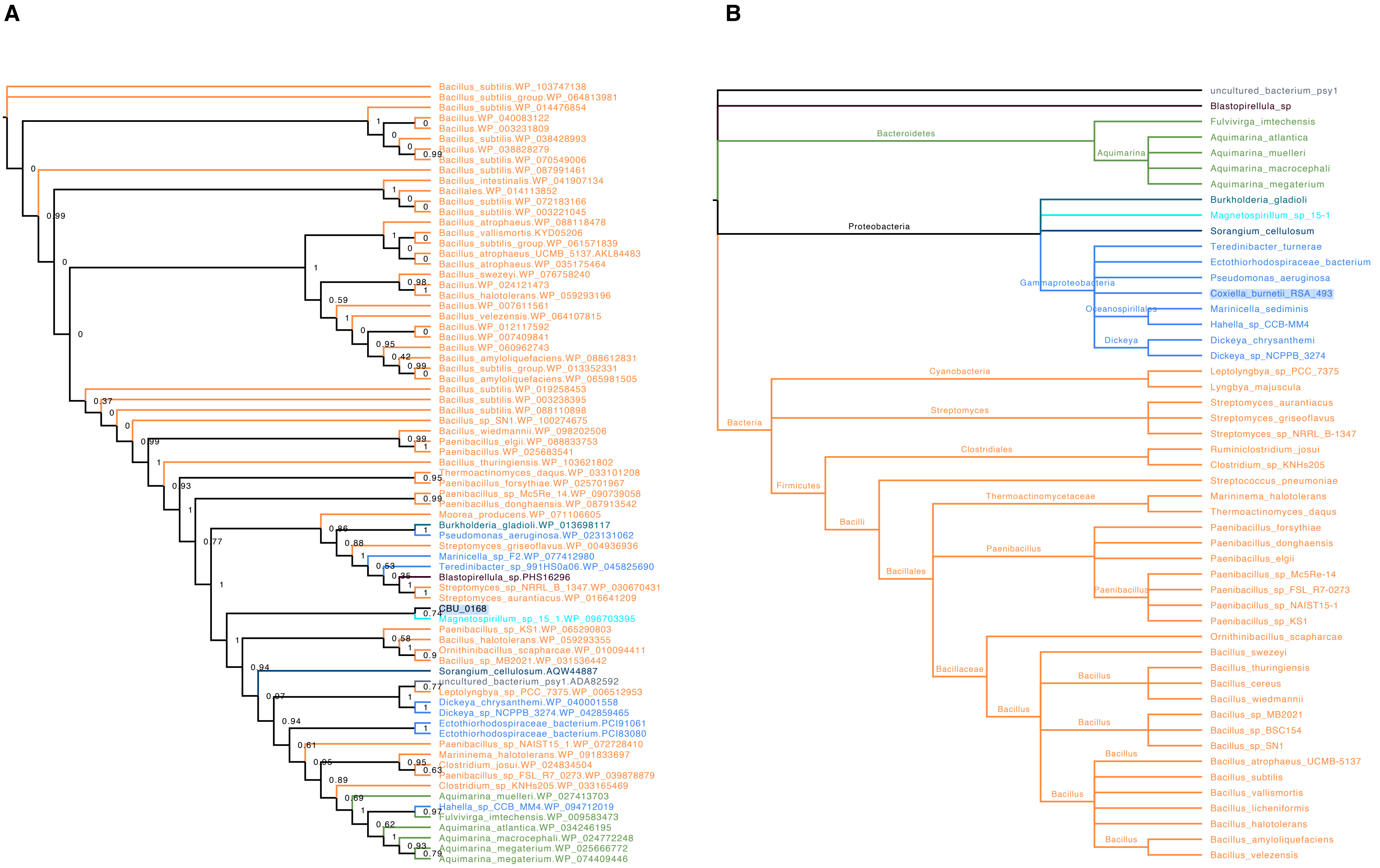

A
Bacillus_subtilis.WP_103747138
Bacillus_subtilis_group.WP_064813981
Bacillus_subtilis.WP_014476854
Bacillus.WP_040083122
Bacillus.WP_003231809
Bacillus_subtilis.WP_038428993
Bacillus.WP_038828279
Bacillus_subtilis.WP_070549006
Bacillus_subtilis.WP_087991461
Bacillus_intestinalis.WP_041907134
Bacillales.WP_014113852
Bacillus_subtilis.WP_072183166
Bacillus_subtilis.WP_003221045
Bacillus_atrophaeus.WP_088118478
Bacillus_vallismortis.KYD05206
Bacillus_subtilis_group.WP_061571839
Bacillus_atrophaeus_UCMB_5137.AKL84483
Bacillus_atrophaeus.WP_035175464
Bacillus_swezeyi.WP_076758240
Bacillus.WP_024121473
Bacillus_halotolerans.WP_059293196
Bacillus.WP_007611561
Bacillus_velezensis.WP_064107815
Bacillus.WP_012117592
Bacillus.WP_007409841
Bacillus.WP_060962743
Bacillus_amyloliquefaciens.WP_088612831
Bacillus_subtilis_group.WP_013352331
Bacillus_amyloliquefaciens.WP_065981505
Bacillus_subtilis.WP_019258453
Bacillus_subtilis.WP_003238395
Bacillus_subtilis.WP_088110898
Bacillus_sp_SN1.WP_100274675
Bacillus_wiedmannii.WP_098202506
Paenibacillus_elgii.WP_088833753
Paenibacillus.WP_025683541
Bacillus_thuringiensis.WP_103621802
Thermoactinomyces_daqus.WP_033101208
Paenibacillus_forsythiae.WP_025701967
Paenibacillus_sp_Mc5Re_14.WP_090739058
Paenibacillus_donghaensis.WP_087913542
Moorea_producens.WP_071106605
Burkholderia_gladioli.WP_013698117
Pseudomonas_aeruginosa.WP_023131062
Streptomyces_griseoflavus.WP_004936936
Marinicella_sp_F2.WP_077412980
Teredinibacter_sp_991HS0a06.WP_045825690
Blastopirellula_sp.PHS16296
Streptomyces_sp_NRRL_B_1347.WP_030670431
Streptomyces_aurantiacus.WP_016641209
CBU_0168
Magnetospirillum_sp_15_1.WP_096703395
Paenibacillus_sp_KS1.WP_065290803
Bacillus_halotolerans.WP_059293355
Ornithinibacillus_scapharcae.WP_010094411
Bacillus_sp_MB2021.WP_031536442
Sorangium_cellulosum.AQW44887
uncultured_bacterium_psy1.ADA82592
Leptolyngbya_sp_PCC_7375.WP_006512953
Dickeya_chrysanthemi.WP_040001558
Dickeya_sp_NCPPB_3274.WP_042859465
Ectothiorhodospiraceae_bacterium.PCI91061
Ectothiorhodospiraceae_bacterium.PCI83080
Paenibacillus_sp_NAIST15_1.WP_072728410
Marininema_halotolerans.WP_091833697
Clostridium_josui.WP_024834504
Paenibacillus_sp_FSL_R7_0273.WP_039878879
Clostridium_sp_KNHs205.WP_033165469
Aquimarina_muelleri.WP_027413703
Hahella_sp_CCB_MM4.WP_094712019
Fulvivirga_imtechensis.WP_009583473
Aquimarina_atlantica.WP_034246195
Aquimarina_macrocephali.WP_024772248
Aquimarina_megaterium.WP_025666772
Aquimarina_megaterium.WP_074409446
B
uncultured_bacterium_psy1
Blastopirellula_sp
Bacteroidetes
Fulvivirga_imtechensis
Aquimarina
Aquimarina_atlantica
Aquimarina_muelleri
Aquimarina_macrocephali
Aquimarina_megaterium
Burkholderia_gladioli
Magnetospirillum_sp_15-1
Proteobacteria
Sorangium_cellulosum
Teredinibacter_turnerae
Ectothiorhodospiraceae_bacterium
Gammaproteobacteria
Pseudomonas_aeruginosa
Coxiella_burnetii_RSA_493
Oceanospirillales
Marinicella_sediminis
Hahella_sp_CCB-MM4
Dickeya
Dickeya_chrysanthemi
Dickeya_sp_NCPPB_3274
Cyanobacteria
Leptolyngbya_sp_PCC_7375
Lyngbya_majuscula
Streptomyces
Streptomyces_aurantiacus
Bacteria
Streptomyces_griseoflavus
Streptomyces_sp_NRRL_B-1347
Clostridiales
Ruminiclostridium_josui
Clostridium_sp_KNHs205
Firmicutes
Streptococcus_pneumoniae
Thermoactinomycetaceae
Marininema_halotolerans
Thermoactinomyces_daqus
Bacilli
Paenibacillus_forsythiae
Paenibacillus
Paenibacillus_donghaensis
Paenibacillus_elgii
Bacillales
Paenibacillus_sp_Mc5Re-14
Paenibacillus_sp_FSL_R7-0273
Paenibacillus_sp_NAIST15-1
Paenibacillus_sp_KS1
Ornithinibacillus_scapharcae
Bacillus_swezeyi
Bacillaceae
Bacillus
Bacillus_thuringiensis
Bacillus_cereus
Bacillus_wiedmannii
Bacillus_sp_MB2021
Bacillus_sp_BSC154
Bacillus_sp_SN1
Bacillus_atrophaeus_UCMB-5137
Bacillus_subtilis
Bacillus_vallismortis
Bacillus_licheniformis
Bacillus_halotolerans
Bacillus_amyloliquefaciens
Bacillus_velezensis

CBU_0159

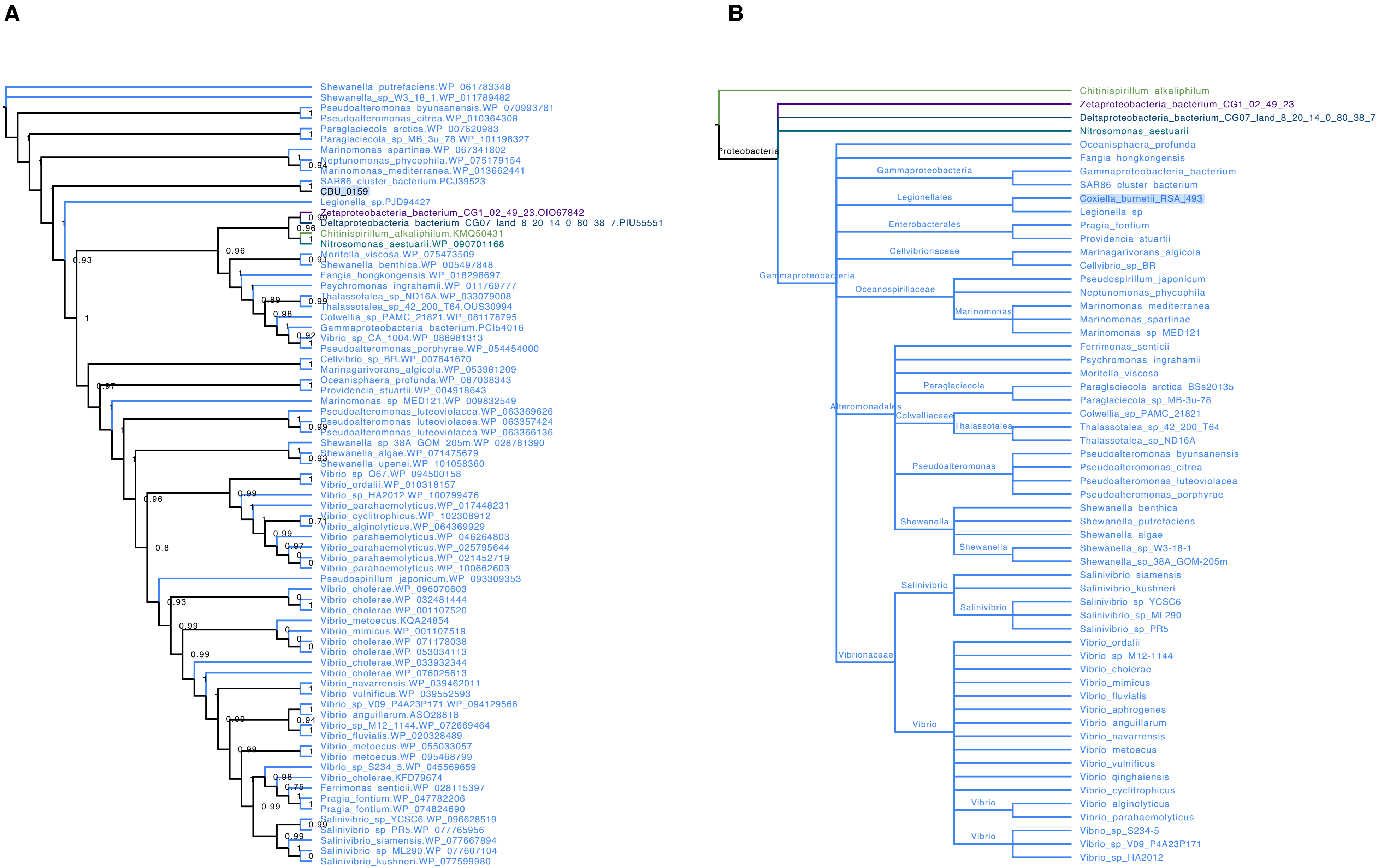
A
Shewanella_putrefaciens.WP_061783348
Shewanella_sp_W3_18_1.WP_011789482
Pseudoalteromonas_byunsanensis.WP_070993781
Pseudoalteromonas_citrea.WP_010364308
Paraglaciecola_arctica.WP_007620983
Paraglaciecola_sp_MB_3u_78.WP_101198327
Marinomonas_spartinae.WP_067341802
Neptunomonas_phycophila.WP_075179154
Marinomonas_mediterranea.WP_013662441
SAR86_cluster_bacterium.PCJ39523
CBU_0159
Legionella_sp.PJD94427
Zetaproteobacteria_bacterium_CG1_02_49_23.OIO67842
Deltaproteobacteria_bacterium_CG07_land_8_20_14_0_80_38_7.PIU55551
Chitinispirillum_alkaliphilum.KMQ50431
Nitrosomonas_aestuarii.WP_090701168
Moritella_viscosa.WP_075473509
Shewanella_benthica.WP_005497848
Fangia_hongkongensis.WP_018298697
Psychromonas_ingrahamii.WP_011769777
Thalassotalea_sp_ND16A.WP_033079008
Thalassotalea_sp_42_200_T64.OUS30994
Colwellia_sp_PAMC_21821.WP_081178795
Gammaproteobacteria_bacterium.PCI54016
Vibrio_sp_CA_1004.WP_086981313
Pseudoalteromonas_porphyrae.WP_054454000
Cellvibrio_sp_BR.WP_007641670
Marinagarivorans_algicola.WP_053981209
Oceanisphaera_profunda.WP_087038343
Providencia_stuartii.WP_004918643
Marinomonas_sp_MED121.WP_009832549
Pseudoalteromonas_luteoviolacea.WP_063369626
Pseudoalteromonas_luteoviolacea.WP_063357424
Pseudoalteromonas_luteoviolacea.WP_063366136
Shewanella_sp_38A_GOM_205m.WP_028781390
Shewanella_algae.WP_071475679
Shewanella_upenei.WP_101058360
Vibrio_sp_Q67.WP_094500158
Vibrio_ordalii.WP_010318157
Vibrio_sp_HA2012.WP_100799476
Vibrio_parahaemolyticus.WP_017448231
Vibrio_cyclitrophicus.WP_102308912
Vibrio_alginolyticus.WP_064369929
Vibrio_parahaemolyticus.WP_046264803
Vibrio_parahaemolyticus.WP_025795644
Vibrio_parahaemolyticus.WP_021452719
Vibrio_parahaemolyticus.WP_100662603
Pseudospirillum_japonicum.WP_093309353
Vibrio_cholerae.WP_096070603
Vibrio_cholerae.WP_032481444
Vibrio_cholerae.WP_001107520
Vibrio_metoecus.KQA24854
Vibrio_mimicus.WP_001107519
Vibrio_cholerae.WP_071178038
Vibrio_cholerae.WP_053034113
Vibrio_cholerae.WP_033932344
Vibrio_cholerae.WP_076025613
Vibrio_navarrensis.WP_039462011
Vibrio_vulnificus.WP_039552593
Vibrio_sp_V09_P4A23P171.WP_094129566
Vibrio_anguillarum.ASO28818
Vibrio_sp_M12_1144.WP_072669464
Vibrio_fluvialis.WP_020328489
Vibrio_metoecus.WP_055033057
Vibrio_metoecus.WP_095468799
Vibrio_sp_S234_5.WP_045569659
Vibrio_cholerae.KFD79674
Ferrimonas_senticii.WP_028115397
Pragia_fontium.WP_047782206
Pragia_fontium.WP_074824690
Salinivibrio_sp_YCSC6.WP_096628519
Salinivibrio_sp_PR5.WP_077765956
Salinivibrio_siamensis.WP_077667894
Salinivibrio_sp_ML290.WP_077607104
Salinivibrio_kushneri.WP_077599980
B
Proteobacteria
Gammaproteobacteria
Gammaproteobacteria
Legionellales
Enterobacterales
Cellvibrionaceae
Oceanospirillaceae
Marinomonas
Alteromonadales
Paraglaciecola
Colwelliaceae
Thalassotalea
Pseudoalteromonas
Shewanella
Shewanella
Vibrionaceae
Salinivibrio
Salinivibrio
Vibrio
Vibrio
Vibrio
Chitinispirillum_alkaliphilum
Zetaproteobacteria_bacterium_CG1_02_49_23
Deltaproteobacteria_bacterium_CG07_land_8_20_14_0_80_38_7
Nitrosomonas_aestuarii
Oceanisphaera_profunda
Fangia_hongkongensis
Gammaproteobacteria_bacterium
SAR86_cluster_bacterium
Coxiella_burnetii_RSA_493
Legionella_sp
Pragia_fontium
Providencia_stuartii
Marinagarivorans_algicola
Cellvibrio_sp_BR
Pseudospirillum_japonicum
Neptunomonas_phycophila
Marinomonas_mediterranea
Marinomonas_spartinae
Marinomonas_sp_MED121
Ferrimonas_senticii
Psychromonas_ingrahamii
Moritella_viscosa
Paraglaciecola_arctica_BSs20135
Paraglaciecola_sp_MB-3u-78
Colwellia_sp_PAMC_21821
Thalassotalea_sp_42_200_T64
Thalassotalea_sp_ND16A
Pseudoalteromonas_byunsanensis
Pseudoalteromonas_citrea
Pseudoalteromonas_luteoviolacea
Pseudoalteromonas_porphyrae
Shewanella_benthica
Shewanella_putrefaciens
Shewanella_algae
Shewanella_sp_W3-18-1
Shewanella_sp_38A_GOM-205m
Salinivibrio_siamensis
Salinivibrio_kushneri
Salinivibrio_sp_YCSC6
Salinivibrio_sp_ML290
Salinivibrio_sp_PR5
Vibrio_ordalii
Vibrio_sp_M12-1144
Vibrio_cholerae
Vibrio_mimicus
Vibrio_fluvialis
Vibrio_aphrogenes
Vibrio_anguillarum
Vibrio_navarrensis
Vibrio_metoecus
Vibrio_vulnificus
Vibrio_qinghaiensis
Vibrio_cyclitrophicus
Vibrio_alginolyticus
Vibrio_parahaemolyticus
Vibrio_sp_S234-5
Vibrio_sp_V09_P4A23P171
Vibrio_sp_HA2012

CBU_0006, 0040, 0384 0523, 0554, 1076, 1090, 1186, 1217a, 1270, 1570b, 1590b, 1639c, 1699a, 1716a, 1758a, 1785, 1896a, 1959b, 1987b

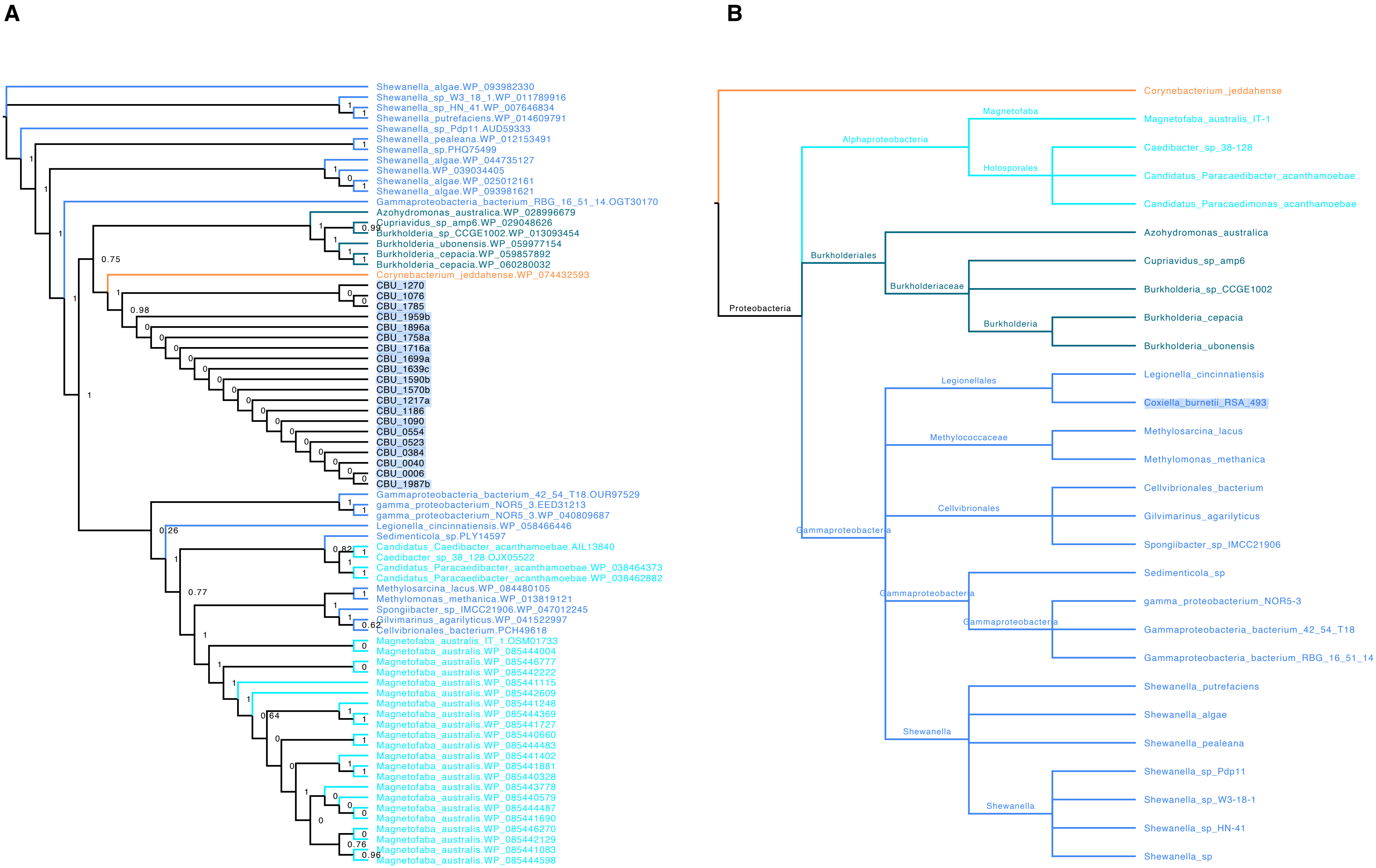

Supplement: evab108_Supplementary_Data [file evab108_supplementary_data.zip › FigS1_rev_v1.pdf]
