# Supplementary figures and images for: Coxiella burnetii and Related Tick Endosymbionts Evolved from Pathogenic Ancestors
Source: Genome Biol Evol. 2021 May 19;13(7):evab108. doi: 10.1093/gbe/evab108 (PMC8290121; doi:10.1093/gbe/evab108)

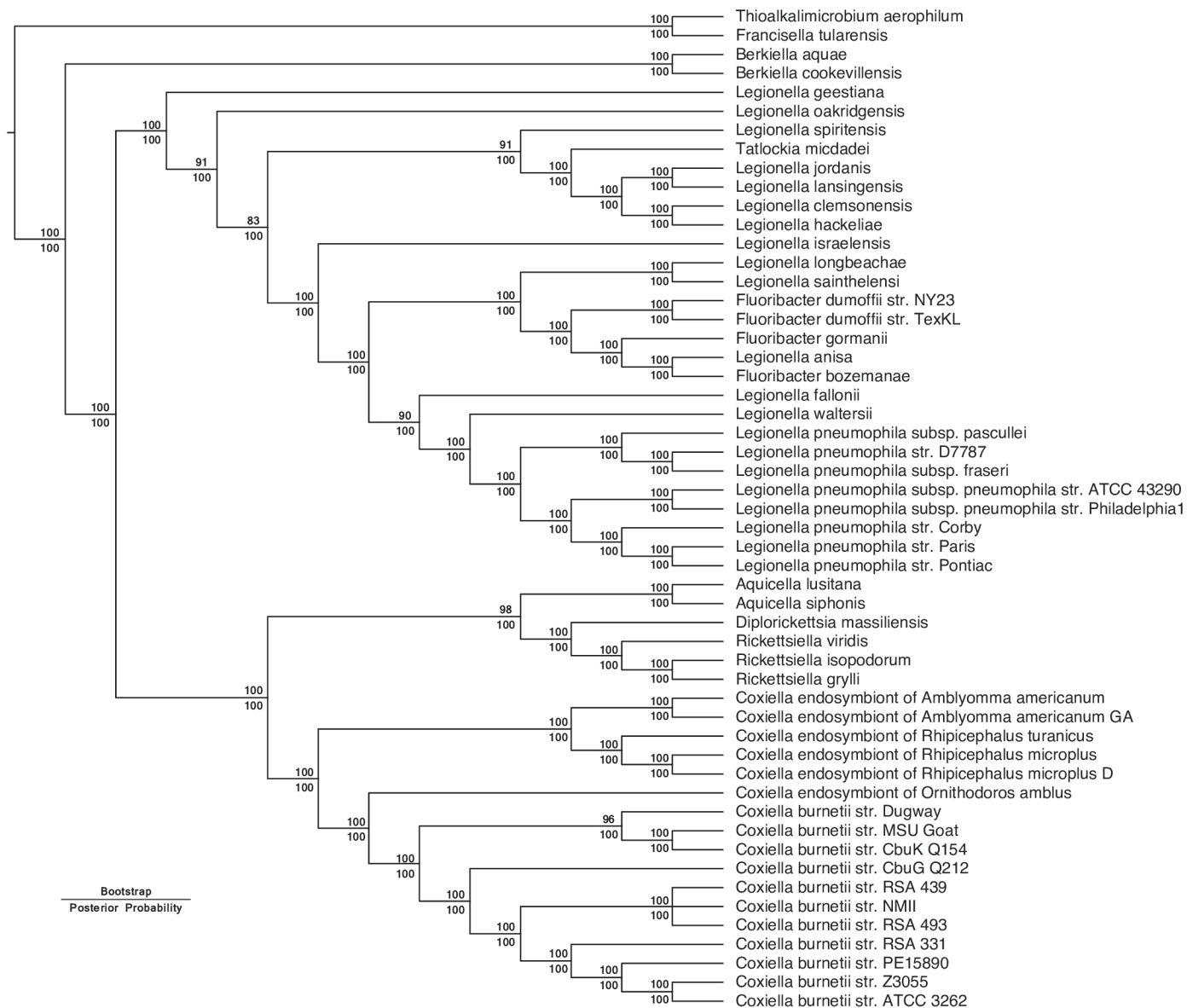

Supplement: evab108_Supplementary_Data [file evab108_supplementary_data.zip › FigS2_rev.pdf]
